# Supplementary material for: Facile and general electrochemical deuteration of unactivated alkyl halides
Source: Nat Commun. 2022 Jun 30;13:3774. doi: 10.1038/s41467-022-31435-9 (PMC9247074; doi:10.1038/s41467-022-31435-9)
Supplement: Supplementary file 1 — Supplementary Information [file 41467_2022_31435_MOESM1_ESM.pdf]

Supplementary Information

Facile and General Electrochemical Deuteration of Unactivated Alkyl Halides

Pengfei Li[1], Chengcheng Guo[1], Siyi Wang[1], Dengke Ma[1], Tian Feng[1], Yanwei Wang[1] and Youai Qiu[1]*

[1]State Key Laboratory and Institute of Elemento-Organic Chemistry, Frontiers Science Center for New Organic Matter, College of Chemistry, Nankai University, 94 Weijin Road, Tianjin, 300071, China

***qiuyouai@nankai.edu.cn**

Table of Contents

Supplementary Methods 2

General Remarks 2

Optimization of the of Reaction Conditions 3

Mechanistic Investigations 5

Graphical Guide 11

Characterization Data of Products 12

Cyclic Voltammetry 40

GC-MS Date 43

NMR Spectrum 44

Supplementary References 112

Supplementary Methods

General Remarks

Catalytic reactions were carried out in undivided electrochemical cells (15 mL) using pre-dried glassware, if not noted otherwise. Solvents were obtained from commercial sources. All the starting materials were obtained from commercial sources or synthesized according to literature methods (>95% purity).[1-4] Commercially available chemicals were obtained from *Bide Pharmatech Ltd, Tianjin Heowns OPDE Technologies and Shanghai Macklin Biochemical Co* used as received unless otherwise stated. Lead plate electrodes (10 mm × 15 mm × 0.3 mm, 99.9%; obtained from Shengshida, Hebei, China), carbon felt electrode and graphite felt electrodes (10 mm × 15 mm × 5 mm, Jinglong company, Beijing, China) were connected using stainless steel adapters. Electrocatalysis was conducted using an HSPY-36-03 potentiostat in constant current mode. Cyclic Voltammetry studies were performed using a Shanghai Chenhua CHI630E workstation and Nova 2.0 software. Yields refer to isolated compounds, estimated to be >95% purity as determined by ^{1}H-NMR. GCMS analysis was performed on an Aligent 7890/5975C-GS/MSD. Flash chromatography was performed using Silica gel (200-300 mesh) purchased from Qingdao Haiyang Chemical Co., China. NMR spectra were recorded on Bruker AVANCE AV 400 in the solvent indicated; chemical shifts are given in ppm relative to the residual solvent peak. The HRMS data were collected on a MicrOTOF mass spectrometer with ESI mass analyzer.

Optimization of the of Reaction Conditions

Supplementary Table 1: Optimization for electrocatalytic deuteration of alkyl bromides

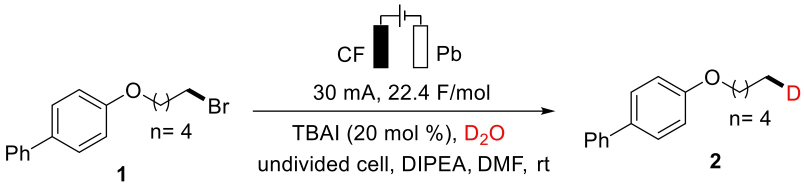

Entry	Variation	Yield %[b]	D - inc %[c]
**1**	**None**	**96**	**99**
2	10 eq D_2O	94	84
3	30 eq D_2O	91	95
4	Fe (-)	trace	0
5	Cu (-)	97	97
6	Nb (-)	trace	0
7	Zn (+)	38	63
8	Mg (+)	99	89
9	DBU	89	99
10	Et_3N	94	99
11	PPh_3	52	95
12	NPh_3	94	99
13	1 eq DIPEA	66	99
14	2 eq DIPEA	93	99
15	50% TBAI	91	99
16	100% TBAI	84	99
17	NaI	87	99
18	TBAB	90	99
19	$TBAClO_4$	94	99

[a] Reaction conditions: undivided cell, carbon felt anode, lead plate cathode constant current = 30 mA, alkyl halide **1** (0.5 mmol), D_2O (25.0 mmol), TBAI (20 mol %), DIPEA (1.5 mmol), DMF (5.0 mL), rt, 10 h. [b] Yield of isolated product. [c] Deuterium incorporation determined by ^{1}H NMR. DIPEA = *N*, *N*-Diisopropylethylamine, TBAI = ^{n}Bu$_4$NI, N.D. = not detected, rt = room temperature.

Supplementary Table 2: Optimization for electrocatalytic deuteration of alkyl chlorides.[a]

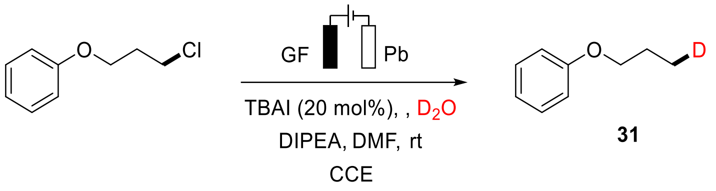

Entry	Current	Anode	Yield %[b]	D-inc %[c]
1	30 mA	CF	70	90
2	50 mA	CF	68	90
3	50 mA	GF[d]	70	99

[a] Reaction conditions: undivided cell, carbon felt anode, lead plate cathode, constant current, (3-chloropropoxy)benzene (0.5 mmol), D_2O (50.0 equiv), TBAI (20 mol%), DIPEA (3.0 equiv), DMF (5.0 mL), rt, 10 h. [b] Yield of isolated product. [c] Deuterium incorporation determined by ^{1}H NMR. DIPEA = *N*, *N*-Diisopropylethylamine, TBAI = ^{n}Bu$_4$NI. [d] GF = graphite felt anode. rt = room temperature.

Mechanistic Investigations

General procedure for D-DMF as the solvent:

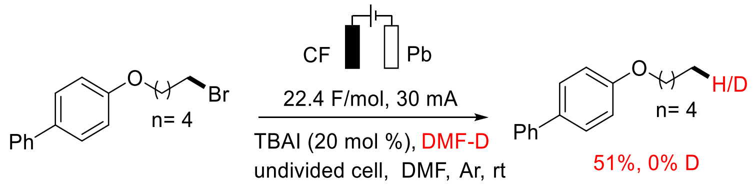

The electrocatalysis was carried out in an undivided cell with a carbon felt anode (10 mm × 15 mm × 5 mm) and a lead cathode (10 mm × 15 mm × 0.3 mm). To a 15 mL pre-dried undivided electrochemical cell (15 mL) equipped with a magnetic bar were added alkyl bromide (0.3 mmol, 1 equiv), TBAI (0.06 mmol, 20 mol%), and D-DMF (3.0 mL). Then D_2O (15 mmol, 50 equiv) and DIPEA (0.9 mmol, 3.0 equiv) were added via a syringe. The electrocatalysis was performed at room temperature with a constant current of 30 mA maintained for 10 h. The carbon felt anode was washed with EtOAc (3 × 5 mL) in an ultrasonic bath. H_2O (20 mL) was added to the system, and the resulting mixture was extracted with EtOAc (3 x 20 mL). The combined organic phase was dried with anhydrous Na_2SO_4, filtered, and concentrated in vacuo. The crude product was purified by column chromatography to furnish the desired product.

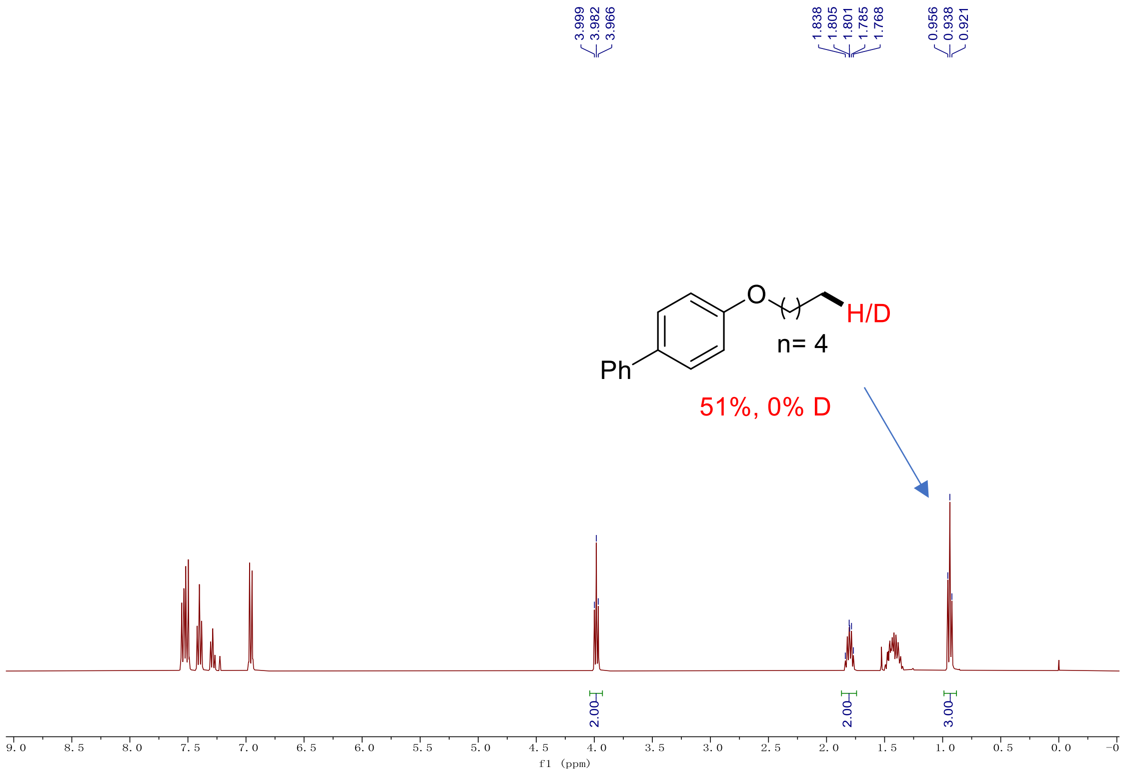

Supplementary Figure 1. ^{1}H NMR (400 MHz, Chloroform-*d*)

Effect of iodine:

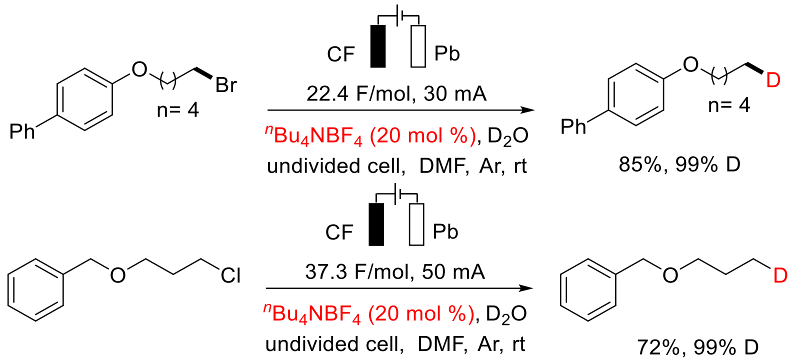

The electrocatalysis was carried out in an undivided cell with a graphite felt anode (10 mm × 15 mm × 5 mm) and a lead cathode (10 mm × 15 mm × 0.3 mm). To a 15 mL pre-dried undivided electrochemical cell (15 mL) equipped with a magnetic bar were added alkyl bromide or chloride (0.5 mmol, 1 equiv), ^{n}Bu$_4$NBF$_4$ (32.9 mg, 0.1 mmol, 20 mol%) and DMF (5.0 mL). Then D_2O (25 mmol, 50 equiv) and DIPEA (1.5 mmol, 3.0 equiv) were added via a syringe. The electrocatalysis was performed at room temperature with a constant current of 50 mA maintained for 10 h. The carbon felt anode was washed with EtOAc (3 × 5 mL) in an ultrasonic bath. H_2O (20 mL) was added to the system, and the resulting mixture was extracted with EtOAc (3 x 20 mL). The combined organic phase was dried with anhydrous Na_2SO_4, filtered, and concentrated in vacuo. The crude product was purified by column chromatography to furnish the desired product.

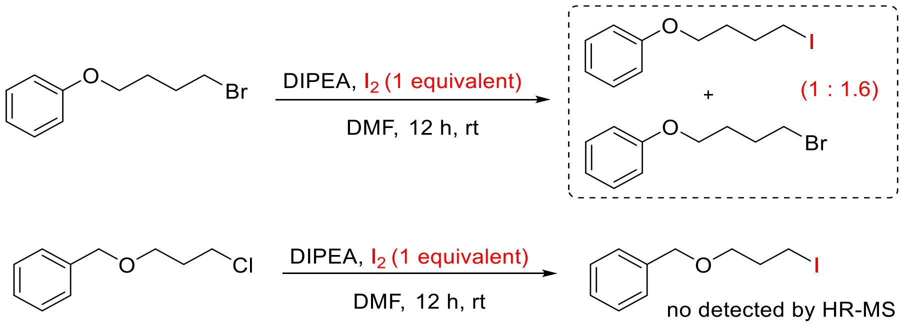

The mixture of alkyl bromide or chloride (0.5 mmol, 1 equiv), I_2 (0.5 mmol 1 equiv), DIPEA (1.5 mmol, 3.0 equiv), and DMF (5.0 mL) were charged into a reaction tube. The reaction mixture was stirred at room temperature for 10 h. H_2O (20 mL) was added to the system, and the resulting mixture was extracted with EtOAc (3 x 20 mL). The combined organic phase was dried with anhydrous Na_2SO_4, filtered, and concentrated

in vacuo. Determined by ^{1}H NMR (Supplementary Figure 2 and 3).

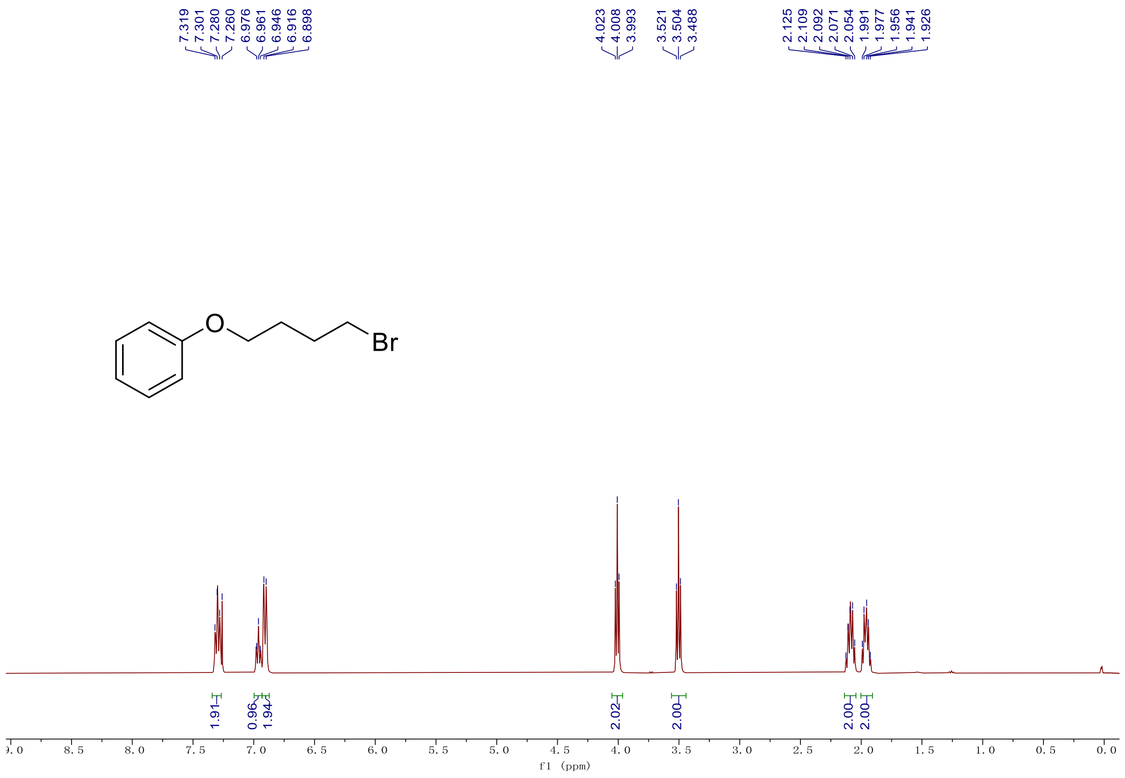

Supplementary Figure 2. ^{1}H NMR (400 MHz, Chloroform-*d*)

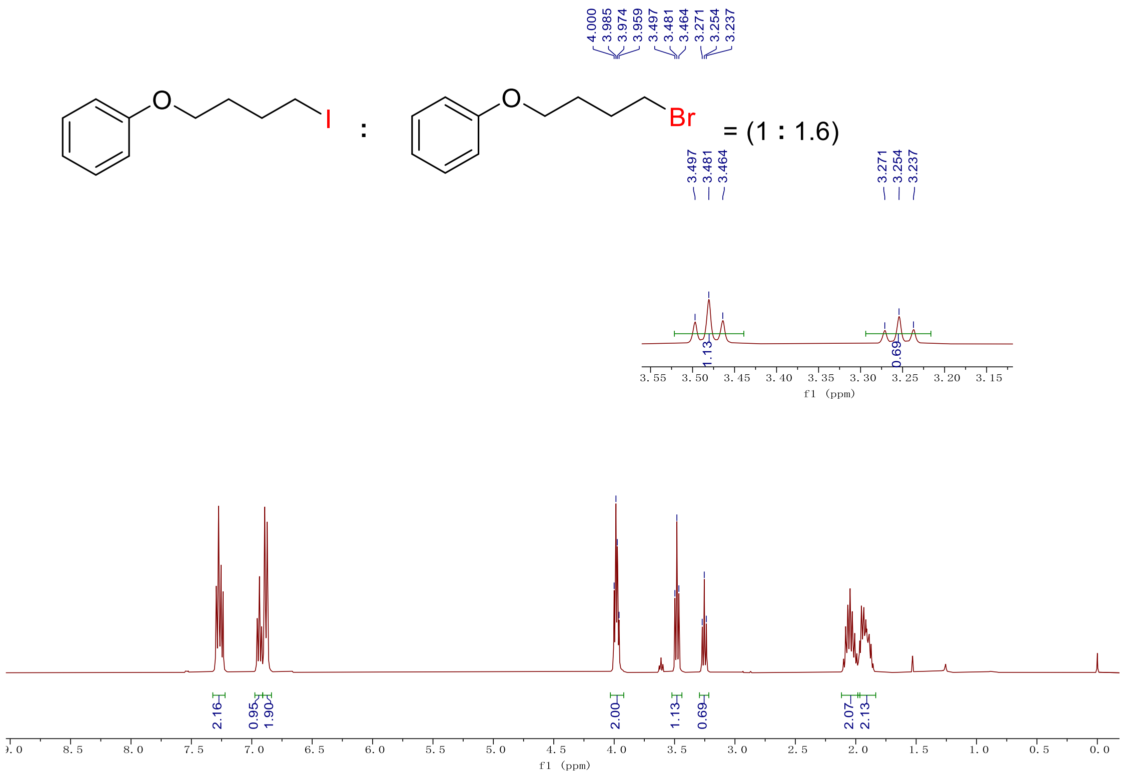

Supplementary Figure 3. ^{1}H NMR (400 MHz, Chloroform-*d*)

Consumed charge studies:

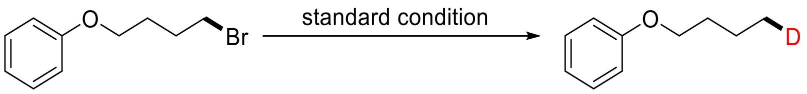

The electrocatalysis was carried out in an undivided cell with a carbon felt anode (10 mm × 15 mm × 5 mm) and a lead cathode (10 mm × 15 mm × 0.3 mm). To a 15 mL pre-dried undivided electrochemical cell (15 mL) equipped with a magnetic bar were added alkyl bromide (0.5 mmol, 1 equiv), and DMF (5.0 mL). Then D_2O (25 mmol, 50 equiv) and DIPEA (1.5 mmol, 3.0 equiv) were added via a syringe. The electrocatalysis was performed at room temperature with a constant current of 30 mA maintained for 10 h. The carbon felt anode was washed with EtOAc (3 × 5 mL) in an ultrasonic bath. H_2O (20 mL) was added to the system, and the resulting mixture was extracted with EtOAc (3 x 20 mL). The combined organic phase was dried with anhydrous Na_2SO_4, filtered, and concentrated in vacuo. Determined by ^1H NMR using CH_2Br_2 (0.5 mmol) as an internal standard.

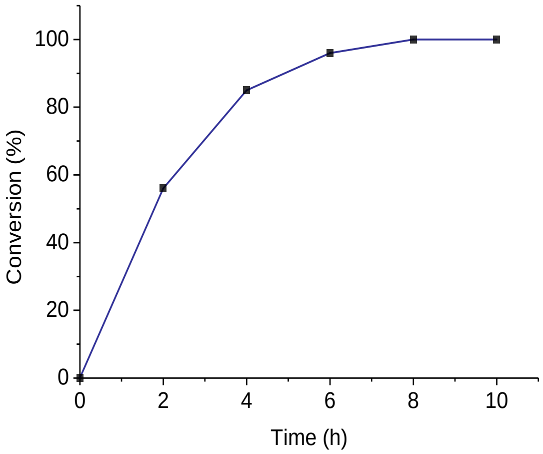

Supplementary Figure 4. Conversion rate

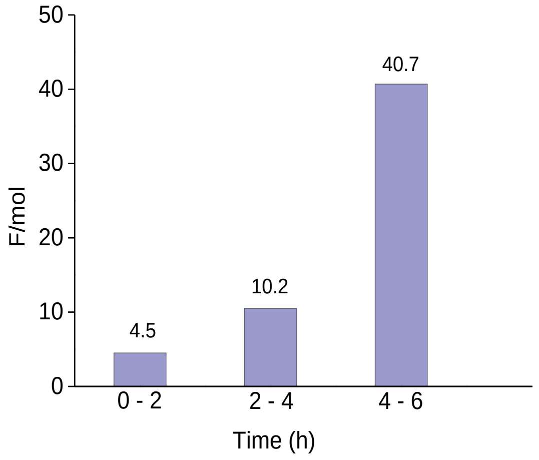

Supplementary Figure 5. consumed charge

The consumed charge for each substrate is calculated by the following formula.

$$\mathrm{F/mol} = \frac{i(\mathrm{mA}) \times t(\mathrm{S})}{(0.5 - \mathrm{x}) \times 10^{-3} \times 96500}$$

x = raw materials consumed

Radical inhibition reaction:

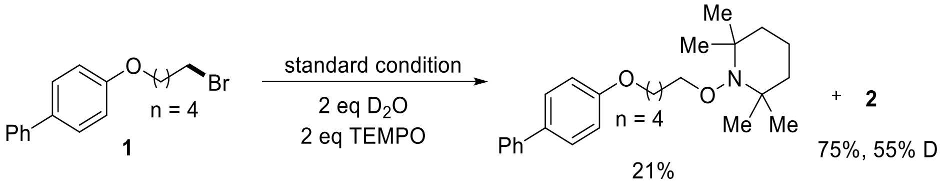

The electrocatalysis was carried out in an undivided cell with a carbon felt anode (10 mm × 15 mm × 5 mm) and a lead cathode (10 mm × 15 mm × 0.3 mm). To a 15 mL pre-dried undivided electrochemical cell (15 mL) equipped with a magnetic bar were added **1** (0.5 mmol, 1 equiv), TBAI (36.9 mg, 0.1 mmol, 20 mol%), TEMPO (1.0 mmol, 2 equiv) and DMF (5.0 mL). Then D_2O (1.0 mmol, 2 equiv) and DIPEA (1.5 mmol, 3.0

equiv) were added via a syringe. The electrocatalysis was performed at room temperature with a constant current of 30 mA maintained for 10 h. The carbon felt anode was washed with EtOAc (3 × 5 mL) in an ultrasonic bath. After that, the system was added H_2O (20 mL) and extracted with EtOAc (3 x 20 mL), the combined organic phase was dried by anhydrous Na_2SO_4, filtered, and concentrated in vacuo. The crude product was purified by column chromatography to furnish the desired product.

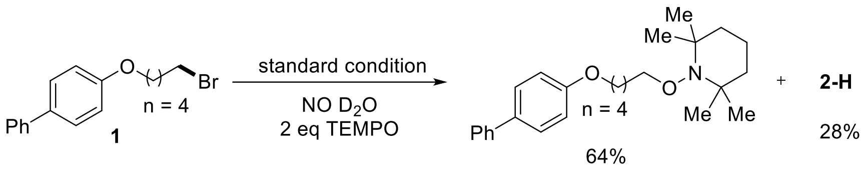

The electrocatalysis was carried out in an undivided cell with a carbon felt anode (10 mm × 15 mm × 5 mm) and a lead cathode (10 mm × 15 mm × 0.3 mm). To a 15 mL pre-dried undivided electrochemical cell (15 mL) equipped with a magnetic bar was added **1** (0.5 mmol, 1 equiv), TBAI (36.9 mg, 0.1 mmol, 20 mol%), TEMPO (1.0 mmol, 2 equiv) and DMF (5.0 mL). Then DIPEA (1.5 mmol, 3.0 equiv) was added via a syringe. The electrocatalysis was performed at room temperature with a constant current of 30 mA maintained for 10 h. The carbon felt anode was washed with EtOAc (3 × 5 mL) in an ultrasonic bath. After that, the system was added H_2O (20 mL) and extracted with EtOAc (3 x 20 mL), the combined organic phase was dried by anhydrous Na_2SO_4, filtered, and concentrated in vacuo. The crude product was purified by column chromatography to furnish the desired product.

Graphical Guide

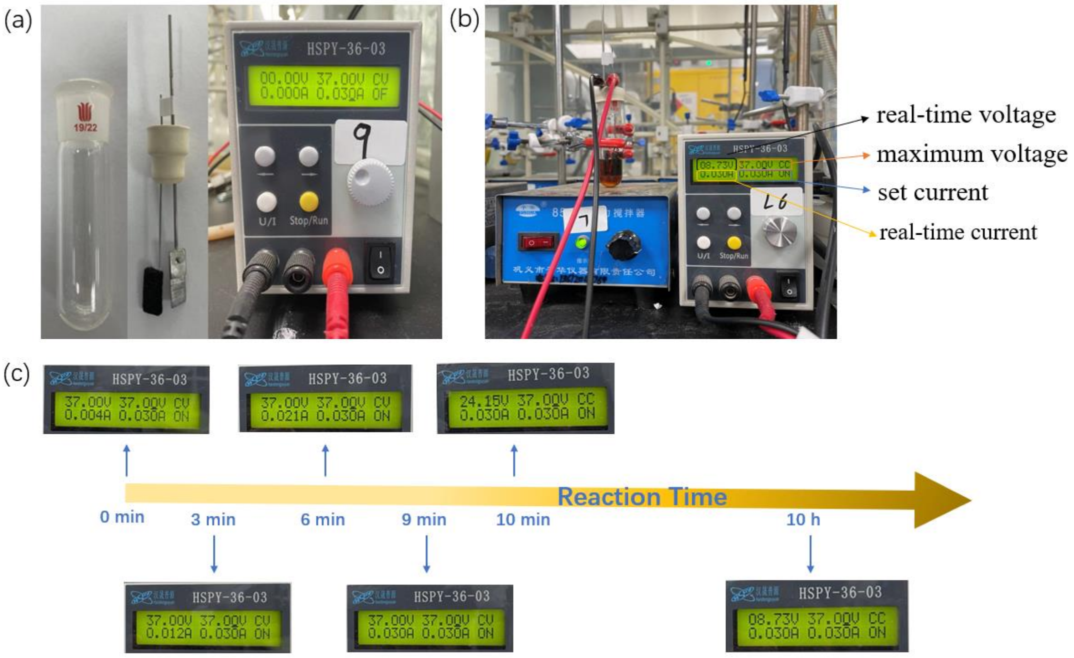

Supplementary Figure 6. (a) and (b): Reaction system; (c): Without TBAI system

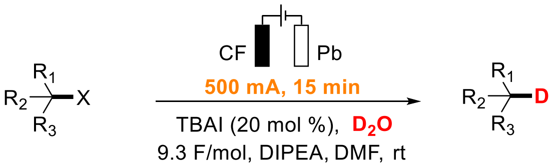

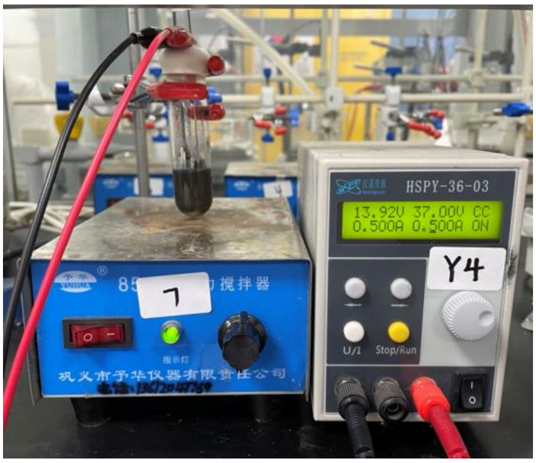

Supplementary Figure 7. 500 mA Condition

Characterization Data of Products

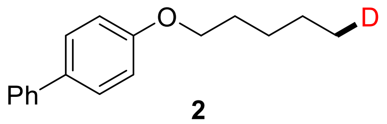

2

Compound **2** was prepared following the general procedure, purification by column chromatography on silica gel (petroleum ether/EtOAc = 100:1) yielded **2** (115.7 mg, 96%, 99% D) as a white solid. M.p.: 59–60 °C. ^{1}H NMR (400 MHz, Chloroform-d) δ 7.63 – 7.58 (m, 4H), 7.50 – 7.46 (m, 2H), 7.37 (t, J = 7.2 Hz, 1H), 7.06 – 7.02 (m, 2H), 4.06 (t, J = 6.4 Hz, 2H), 1.92 – 1.85 (m, 2H), 1.57 – 1.43 (m, 4H), 1.04 – 0.99 (m, 2H). ^{13}C NMR (100 MHz, Chloroform-*d*) δ 158.8, 140.9, 133.6, 128.7, 128.1, 126.7, 126.6, 114.8, 68.1, 29.0, 28.2, 22.4, 13.8 (t, J = 20.0 Hz). HR-MS (ESI) m/z calc. for $C_{17}H_{20}DO$ $[M+H]^+$: 242.1650, found: 242.1653.

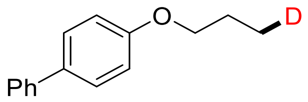

3

4-(Propoxy-3-*d*)-1,1'-biphenyl

Compound **3** was prepared following the general procedure, purification by column chromatography on silica gel (petroleum ether/EtOAc = 100:1) yielded **3** (99.6 mg, 94%, 99% D) as a white solid. M.p.: 65–66 °C. ^{1}H NMR (400 MHz, Chloroform-*d*) δ 7.60 – 7.54 (m, 4H), 7.46 – 7.43 (m, 2H), 7.35 – 7.31 (m, 1H), 7.02 – 6.99 (m, 2H), 3.99 (t, J = 6.4 Hz, 2H), 1.90 – 1.83 (m, 2H), 1.11 – 1.05 (m, 2H). ^{13}C NMR (100 MHz, Chloroform-*d*) δ 158.8, 140.9, 133.6, 128.7, 128.1, 126.8, 126.6, 114.8, 69.6, 22.6, 10.3 (t, J = 20.0 Hz). HR-MS (ESI) m/z calc. for $C_{15}H_{16}DO$ $[M+H]^+$: 214.1337, found: 214.1330. Spectroscopic data match those previously reported in the literature.[3]

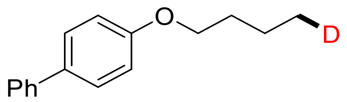

4

4-(Butoxy-4-*d*)-1,1'-biphenyl

Compound **4** was prepared following the general procedure, purification by column chromatography on silica gel (petroleum ether/EtOAc = 100:1) yielded **4** (102.1 mg, 90%, 99% D) as a white solid. M.p.: 68–71 °C. ^{1}H NMR (400 MHz, Chloroform-*d*) δ

7.63 – 7.57 (m, 4H), 7.49 – 7.45 (m, 2H), 7.38 – 7.33 (m, 1H), 7.05 – 7.01 (m, 2H), 3.97 (t, J = 6.4 Hz, 2H), 1.89 – 1.82 (m, 2H), 1.61 – 1.53 (m, 2H), 1.08 – 1.02 (m, 2H). ^{13}C NMR (100 MHz, Chloroform-*d*) δ 158.8, 141.0, 133.6, 128.8, 128.2, 126.8, 126.7, 114.8, 67.8, 31.4, 19.3, 13.7 (t, J = 20.0 Hz). HR-MS (ESI) m/z calc. for $C_{16}H_{18}DO$ $[M+H]^+$: 228.1493, found: 228.1499. Spectroscopic data match those previously reported in the literature.[3]

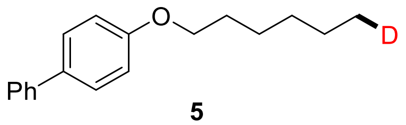

5

4-((Hexyl-6-*d*)oxy)-1,1'-biphenyl

Compound **5** was prepared following the general procedure, purification by column chromatography on silica gel (petroleum ether/EtOAc = 100:1) yielded **5** (102.0 mg, 80%, 99% D) as a white solid. M.p.: 61–62 °C. ^{1}H NMR (400 MHz, Chloroform-*d*) δ 7.60 – 7.54 (m, 4H), 7.47 – 7.43 (m, 2H), 7.35 – 7.31 (m, 1H), 7.02 – 6.99 (m, 2H), 4.02 (t, J = 6.4 Hz, 2H), 1.88 – 1.80 (m, 2H), 1.55 – 1.48 (m, 2H), 1.41 – 1.37 (m, 4H), 0.96 – 0.92 (m, 2H). ^{13}C NMR (100 MHz, Chloroform-*d*) δ 158.8, 140.9, 133.6, 128.7, 128.1, 126.7, 126.6, 114.8, 68.1, 31.6, 29.3, 25.8, 22.6, 13.8 (t, J = 20.0 Hz). HR-MS (ESI) m/z calc. for $C_{18}H_{22}DO$ $[M+H]^+$: 256.1806, found: 256.1806.

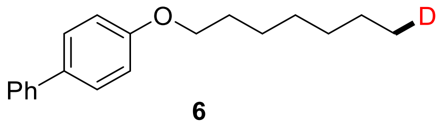

6

Compound **6** was prepared following the general procedure, purification by column chromatography on silica gel (petroleum ether/EtOAc = 100:1) yielded **6** (84.6 mg, 63%, 97% D) as a white solid. M.p.: 67–68 °C. ^{1}H NMR (400 MHz, Chloroform-*d*) δ 7.59 – 7.53 (m, 4H), 7.45 – 7.41 (m, 2H), 7.34 – 7.30 (m, 1H), 7.01 – 6.97 (m, 2H), 4.02 (t, J = 6.4 Hz, 2H), 1.87 – 1.79 (m, 2H), 1.55 – 1.46 (m, 2H), 1.42 – 1.33 (m, 6H), 0.93 – 0.91 (m, 2H). ^{13}C NMR (100 MHz, Chloroform-*d*) δ 158.8, 140.9, 133.6, 128.7, 128.1, 126.7, 126.6, 114.8, 68.1, 31.8, 29.4, 29.1, 26.1, 22.6, 13.8 (t, J = 20.0 Hz). HR-MS (ESI) m/z calc. for $C_{19}H_{24}DO$ $[M+H]^+$: 270.1963, found: 270.1963.

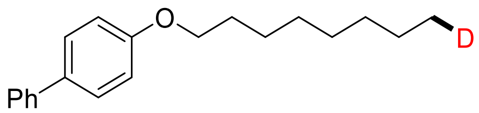

7

4-((Octyl-8-*d*)oxy)-1,1'-biphenyl

Compound **7** was prepared following the general procedure, purification by column chromatography on silica gel (petroleum ether/EtOAc = 100:1) yielded **7** (92.1 mg, 65%, 99% D) as a white solid. M.p.: 69–70 °C. ^{1}H NMR (400 MHz, Chloroform-*d*) δ 7.59 – 7.53 (m, 4H), 7.46 – 7.42 (m, 2H), 7.34 – 7.31 (m, 1H), 7.01 – 6.98 (m, 2H), 4.02 (d, *J* = 6.4 Hz, 2H), 1.87 – 1.80 (m, 2H), 1.54 – 1.46 (m, 2H), 1.38 – 1.33 (m, 8H), 0.93 – 0.89 (m, 2H). ^{13}C NMR (100 MHz, Chloroform-*d*) δ 158.8, 140.9, 133.6, 128.7, 128.1, 126.7, 126.6, 114.8, 68.1, 31.8, 29.4, 29.35, 29.3, 26.1, 22.6, 13.9 (t, *J* = 20.0 Hz). HR-MS (ESI) m/z calc. for $C_{20}H_{26}DO$ $[M+H]^+$: 284.2119, found: 284.2110.

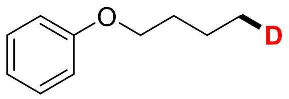

8

(Butoxy-4-*d*)benzene

Compound **8** was prepared following the general procedure, purification by column chromatography on silica gel (petroleum ether/EtOAc = 100:1) yielded **8** (46.0 mg, 61%, 99% D) as a colorless oil. ^{1}H NMR (400 MHz, Chloroform-*d*) δ 7.33 – 7.28 (m, 2H), 6.98 – 6.92 (m, 3H), 3.99 (t, *J* = 6.4 Hz, 2H), 1.83 – 1.76 (m, 2H), 1.56 – 1.48 (m, 2H), 1.03 – 0.97 (m, 2H).^{13}C NMR (100 MHz, Chloroform-*d*) δ 159.2, 129.4, 120.5, 114.5, 67.6, 31.4, 19.2, 13.6 (t, *J* = 20.0 Hz). HR-MS (ESI) m/z calc. for $C_{10}H_{14}DO$ $[M+H]^+$: 152.1180, found: 152.1180.

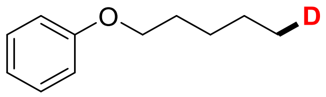

9

((pentyl-5-d)oxy)benzene

Compound **9** was prepared following the general procedure, purification by column chromatography on silica gel (petroleum ether/EtOAc = 100:1) yielded **9** (59.8 mg, 72%, 98% D) as a colorless oil. ^{1}H NMR (400 MHz, Chloroform-*d*) δ 7.32 – 7.28 (m, 2H), 6.97 – 6.91 (m, 3H), 3.98 (t, *J* = 6.4 Hz, 2H), 1.85 – 1.78 (m, 2H), 1.51 – 1.37 (m,

4H), 0.98 – 0.93 (m, 2H). ^{13}C NMR (100 MHz, Chloroform-*d*) δ 159.2, 129.4, 120.5, 114.5, 67.9, 29.0, 28.2, 22.4, 13.8 (t, *J* = 20 Hz). HR-MS (ESI) m/z calc. for $C_{10}H_{16}DO$ $[M+H]^+$: 166.1337, found: 166.1334.

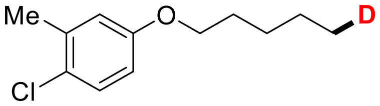

10

1-Chloro-2-methyl-4-((pentyl-5-*d*)oxy)benzene

Compound **10** was prepared following the general procedure, purification by column chromatography on silica gel (petroleum ether/EtOAc = 100:1) yielded **10** (80.9 mg, 76%, 99% D) as a colorless oil. ^{1}H NMR (400 MHz, Chloroform-*d*) δ 7.22 (d, *J* = 8.8 Hz, 1H), 6.78 (d, *J* = 3.2 Hz, 1H), 6.67 (dd, *J* = 8.8, 3.2 Hz, 1H), 3.92 (t, *J* = 6.4 Hz, 2H), 2.35 (s, 3H), 1.82 – 1.75 (m, 2H), 1.48 – 1.35 (m, 4H), 0.97 – 0.92 (m, 2H). ^{13}C NMR (100 MHz, Chloroform-*d*) δ 157.7, 136.9, 129.5, 125.5, 117.1, 113.1, 68.2, 29.0, 28.2, 22.4, 20.3, 13.8 (t, *J* = 20.0 Hz). HR-MS (ESI) m/z calc. for $C_{12}H_{17}DClO$ $[M+H]^+$: 214.1103, found: 214.1101.

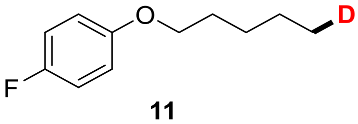

11

1-Fluoro-4-((pentyl-5-*d*)oxy)benzene

Compound **11** was prepared following the general procedure, purification by column chromatography on silica gel (petroleum ether/EtOAc = 100:1) yielded **11** (66.8 mg, 73%, 98% D) as a colorless oil. ^{1}H NMR (400 MHz, Chloroform-*d*) δ 6.99 – 6.93 (m, 2H), 6.86 – 6.80 (m, 2H), 3.91 (t, *J* = 6.4 Hz, 2H), 1.81 – 1.74 (m, 2H), 1.48 – 1.35 (m, 4H), 0.96 – 0.90 (m, 2H). ^{13}C NMR (100 MHz, Chloroform-*d*) δ 157.1 (d, $^{1}J_{C\text{-}F}$ = 240.0 Hz), 155.3 (d, $^{4}J_{C\text{-}F}$ = 2.0 Hz), 155.7 (d, $^{2}J_{C\text{-}F}$ = 22.9 Hz), 115.4 (d, $^{3}J_{C\text{-}F}$ = 8.0 Hz), 68.6, 29.01, 28.2, 22.4, 13.7 (t, *J* = 20 Hz). ^{19}F NMR (375 MHz, Chloroform-*d*) δ -124.5. HR-MS (ESI) m/z calc. for $C_{11}H_{15}DFO$ $[M+H]^+$: 184.1242, found: 184.1244.

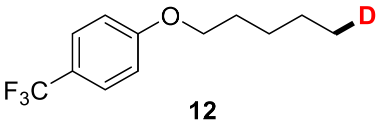

12

1-((Pentyl-5-*d*)oxy)-4-(trifluoromethyl)benzene

Compound **12** was prepared following the general procedure, purification by column chromatography on silica gel (petroleum ether/EtOAc = 100:1) yielded **12** (95.7 mg, 82%, 99% D) as a colorless oil. ^{1}H NMR (400 MHz, Chloroform-*d*) δ 7.53 (d, *J* = 8.8 Hz, 2H), 6.95 (d, *J* = 8.8 Hz, 2H), 3.99 (t, *J* = 6.4 Hz, 2H), 1.85 – 1.78 (m, 2H), 1.49 – 1.35 (m, 4H), 0.96 – 0.91 (m, 2H). ^{13}C NMR (100 MHz, Chloroform-*d*) δ 161.6 (d, $^{4}J_{C-F}$ = 1.3 Hz), 126.8 (q, $^{3}J_{C-F}$ = 3.6 Hz), 124.5 (q, $^{1}J_{C-F}$ = 269.7 Hz), 122.6 (q, $^{2}J_{C-F}$ = 32.5 Hz),114.4, 68.2, 28.8, 28.1, 22.3, 13.7 (t, *J* = 20 Hz). ^{19}F NMR (375 MHz, Chloroform-*d*) δ -61.4. HR-MS (ESI) m/z calc. for $C_{12}H_{15}DF_3O$ $[M+H]^+$: 234.1211, found: 234.1217.

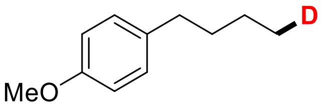

13

1-(Butyl-4-*d*)-4-methoxybenzene

Compound **13** was prepared following the general procedure, purification by column chromatography on silica gel (petroleum ether/EtOAc = 100:1) yielded **13** (57.72 mg, 70%, 99% D) as a colorless oil. ^{1}H NMR (400 MHz, Chloroform-*d*) δ 7.14 – 7.11 (m, 2H), 6.87 – 6.84 (m, 2H), 3.81 (s, 3H), 2.58 (d, *J* = 8.0 Hz, 2 H), 1.63 – 1.56 (m, 2H), 1.40 – 1.33 (m, 2H), 0.97 – 0.91 (m, 2H). ^{13}C NMR (100 MHz, Chloroform-*d*) δ 157.6, 135.0, 129.3, 113.7, 55.3, 34.8, 33.9, 22.3, 13.7 (t, *J* = 20.0 Hz). HR-MS (ESI) m/z calc. for $C_{11}H_{16}DO$ $[M+H]^+$: 166.1337, found: 166.1335.

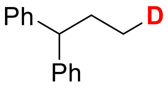

14

(Propane-1,1-diyl-3-*d*)dibenzene

Compound **14** was prepared following the general procedure, purification by column chromatography on silica gel (petroleum ether/EtOAc = 100:1) yielded **14** (75.9 mg, 77%, 98% D) as a colorless oil. ^{1}H NMR (400 MHz, Chloroform-*d*) δ 7.31 – 7.23 (m, 8H), 7.20 – 7.16 (m, 2H), 3.81 (t, *J* = 7.8 Hz, 1H), 2.12 – 2.06 (m, 2H), 0.94 – 0.88 (m, 2H). ^{13}C NMR (100 MHz, Chloroform-*d*) δ 145.2, 128.4, 128.0, 126.1, 53.3, 28.6, 12.6

(t, J = 20.0 Hz). HR-MS (ESI) m/z calc. for $C_{15}H_{16}D$ $[M+H]^+$: 198.1388, found: 198.1385.

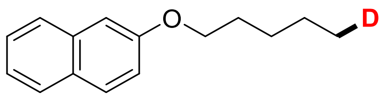

15

2-((Pentyl-5-*d*)oxy)naphthalene

Compound **15** was prepared following the general procedure, purification by column chromatography on silica gel (petroleum ether/EtOAc = 100:1) yielded **15** (87.1 mg, 81%, 99% D) as a colorless oil. ^{1}H NMR (400 MHz, Chloroform-*d*) δ 7.82 – 7.76 (m, 3H), 7.50 – 7.46 (m, 1H), 7.40 – 7.35 (m, 1H), 7.23 – 7.18 (m, 2H), 4.11 (t, J = 6.4 Hz, 2H), 1.94 – 1.87 (m, 2H), 1.58 – 1.43 (m, 4H), 1.04 – 0.98 (m, 2H). ^{13}C NMR (100 MHz, Chloroform-*d*) δ 157.1, 134.6, 129.3, 128.9, 127.7, 126.7, 126.3, 123.5, 119.1, 106.5, 68.0, 29.0, 28.3, 22.5, 13.8 (t, J = 20.0 Hz). HR-MS (ESI) m/z calc. for $C_{15}H_{18}DO$ $[M+H]^+$: 216.1493, found: 216.1497.

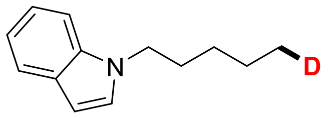

16

1-(Pentyl-5-*d*)-1*H*-indole

Compound **16** was prepared following the general procedure, purification by column chromatography on silica gel (petroleum ether/EtOAc = 100:1) yielded **16** (70.5 mg, 75%, 98% D) as a colorless oil. ^{1}H NMR (400 MHz, Chloroform-*d*) δ 7.69 (d, J = 7.6 Hz, 1H), 7.40 (d, J = 8.0 Hz, 1H), 7.28 – 7.24 (m, 1H), 7.17 – 7.14 (m, 2H), 6.54 (d, J = 3.2 Hz, 1H), 4.16 (t, J = 7.2 Hz, 2H), 1.93 – 1.85 (m, 2H), 1.41 – 1.34 (m, 4H), 0.96 – 0.90 (m, 2H). ^{13}C NMR (100 MHz, Chloroform-*d*) δ 136.0, 128.6, 127.8, 121.3, 121.0, 119.2, 109.4, 100.8, 46.4, 30.0, 29.2, 22.3, 13.7 (t, J = 20.0 Hz). HR-MS (ESI) m/z calc. for $C_{13}H_{17}DN$ $[M+H]^+$: 189.1497, found: 189.1497.

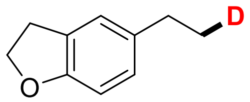

17

5-(Ethyl-2-*d*)-2,3-dihydrobenzofuran

Compound **17** was prepared following the general procedure, purification by column chromatography on silica gel (petroleum ether/EtOAc = 100:1) yielded **17** (52.9 mg, 71%, 99% D) as a colorless oil. ^{1}H NMR (400 MHz, Chloroform-*d*) δ 7.03 (s, 1H), 6.92 (d, *J* = 8.4 Hz, 1H), 6.70 (d, *J* = 8.4 Hz, 1H), 4.53 (t, *J* = 8.4 Hz, 2H), 3.16 (t, *J* = 8.8 Hz, 2H), 2.56 (t, *J* = 7.6 Hz, 2H), 1.22 – 1.16 (m, 2H). ^{13}C NMR (100 MHz, Chloroform-*d*) δ 158.1, 136.4, 127.2, 126.9, 124.4, 108.9, 71.1, 29.9, 28.3, 16.0 (t, *J* = 20.0 Hz). HR-MS (ESI) m/z calc. for $C_{10}H_{12}DO$ [M+H]$^+$: 150.1024, found: 150.1028.

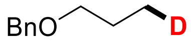

18

((Propoxy-3-*d*)methyl)benzene

Compound **18** was prepared following the general procedure, purification by column chromatography on silica gel (petroleum ether/EtOAc = 100:1) yielded **18** (49.1 mg, 65%, 99% D) as a colorless oil. ^{1}H NMR (400 MHz, Chloroform-*d*) δ 7.27 – 7.26 (m, 4H), 7.22 – 7.17 (m, 1H), 4.43 (s, 2H), 3.36 (t, *J* = 6.8 Hz, 2H), 1.60 – 1.52 (m, 2H), 0.89 – 0.83 (m, 2H). ^{13}C NMR (100 MHz, Chloroform-*d*) δ 138.7, 128.4, 127.6, 127.5, 72.8, 72.1, 22.9, 10.4 (t, *J* = 20.0 Hz). HR-MS (ESI) m/z calc. for $C_{10}H_{14}DO$ [M+H]$^+$: 152.1180, found: 152.1183.

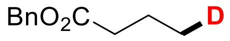

19

Benzyl butanoate-4-*d*

Compound **19** was prepared following the general procedure, purification by column chromatography on silica gel (petroleum ether/EtOAc = 100:1) yielded **19** (75.2 mg, 84%, 99% D) as a colorless oil. ^{1}H NMR (400 MHz, Chloroform-*d*) δ 7.27 – 7.22 (m, 5H), 5.03 (s, 2H), 2.25 (t, *J* = 7.6 Hz, 2H), 1.62 – 1.55 (m, 2H), 0.88 – 0.82 (m, 2H). ^{13}C NMR (100 MHz, Chloroform-*d*) δ 173.5, 136.2, 128.6, 128.2, 66.1, 36.2, 18.4, 13.4 (t, *J* = 20.0 Hz). HR-MS (ESI) m/z calc. for $C_{11}H_{14}DO_2$ [M+H]$^+$: 180.1129, found: 180.1130.

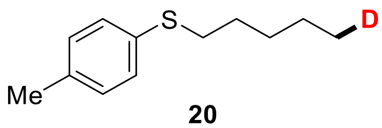

20

(Pentyl-5-*d*)(*p*-tolyl)sulfane

Compound **20** was prepared following the general procedure, purification by column chromatography on silica gel (petroleum ether/EtOAc = 100:1) yielded **20** (85.8 mg, 88%, 99% D) as a colorless oil. ^{1}H NMR (400 MHz, Chloroform-d) δ 7.28 – 7.26 (m, 2H), 7.13 – 7.11 (m, 2H), 2.90 (t, *J* = 7.4 Hz, 2H), 2.34 (s, 3H), 1.69 – 1.61 (m, 2H), 1.45 – 1.30 (m, 4H), 0.93 – 0.88 (m, 2H). ^{13}C NMR (100 MHz, Chloroform-*d*) δ 135.8, 133.2, 129.8, 129.6, 34.3, 31.0, 29.0, 22.2, 21.0, 13.7 (t, *J* = 20.0 Hz). HR-MS (ESI) m/z calc. for $C_{12}H_{18}DS$ $[M+H]^+$: 196.1265, found: 196.1266.

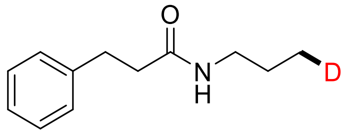

21

3-phenyl-N-(propyl-3-d)propanamide

Compound **21** was prepared following the general procedure, purification by column chromatography on silica gel (petroleum ether/EtOAc = 10:1) yielded **21** (83.5 mg, 87%, 96% D) as a colorless oil. ^{1}H NMR (400 MHz, Chloroform-*d*) δ 7.30 – 7.26 (m, 2H), 7.23 – 7.15 (m, 3H), 5.72 (s, 1H), 3.19 – 3.14 (m, 2H), 3.02 – 2.88 (m, 2H), 2.52 – 2.38 (m, 2H), 1.51 – 1.38 (m, 2H), 0.84 (m, 2H). ^{13}C NMR (150 MHz, Chloroform-*d*) δ 172.1, 141.0, 128.5, 128.4, 126.2, 41.2, 38.53, 31.8, 22.7, 11.2, 11.0, 10.9. HR-MS (ESI) m/z calc. for $C_{12}H_{17}DNO$ $[M+H]^+$: 193.1446, found: 192.1439.

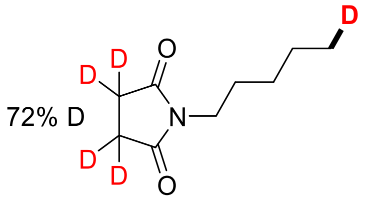

22

1-(butyl-4-d)pyrrolidine-2,5-dione

Compound **22** was prepared following the general procedure, purification by column chromatography on silica gel (petroleum ether/EtOAc = 10:1) yielded **22** (67.2 mg, 79%, 95% D) as a colorless oil. ^{1}H NMR (400 MHz, Chloroform-*d*) δ 3.49 – 3.36 (m, 2H), 2.64 – 2.63 (m, 4H), 1.50 (m 2H), 1.30 – 1.17 (m, 4H), 0.81 (t, *J* = 7.3 Hz, 2H). ^{13}C NMR (150 MHz, Chloroform-*d*) δ 177.3, 38.8, 28.9, 28.1, 27.4, 22.1, 13.7, 13.6,

13.4. HR-MS (ESI) m/z calc. for $C_9H_{15}DNO_2$ $[M+H]^+$: 171.1238, found: 171.1235.

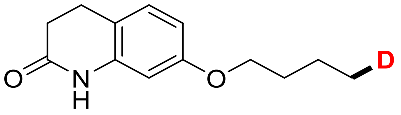

23

7-(Butoxy-4-*d*)-3,4-dihydroquinolin-2(1*H*)-one

Compound **23** was prepared following the general procedure, purification by column chromatography on silica gel (petroleum ether/EtOAc = 2:1) yielded **23** (93.5 mg, 85%, 97% D) as a colorless oil. ^{1}H NMR (400 MHz, Chloroform-d) δ 9.15 (s, 1H), 7.02 (d, J = 8.3 Hz, 1H), 6.52 – 6.51 (m, 1H), 6.40 (d, J = 2.4 Hz, 1H), 3.92 (t, J = 6.5 Hz, 2H), 2.99 – 2.83 (m, 2H), 2.67 – 2.55 (m, 2H), 1.82 – 1.63 (m, 2H), 1.5= – 1.43 (m, 2H), 0.98 – 0.92 (m, 2H). ^{13}C NMR (100 MHz, Chloroform-d) δ 172.5, 158.8, 138.2, 128.5, 115.5, 108.8, 102.3, 67.9, 31.3, 31.1, 24.6, 19.2, 13.6 (t, J = 20.0 Hz). HR-MS (ESI) m/z calc. for $C_{13}H_{17}DNO_2$ $[M+H]^+$: 221.1395, found: 221.1399.

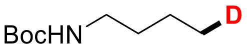

24

***tert*-Butyl (butyl-4-*d*)carbamate**

Compound **24** was prepared following the general procedure, purification by column chromatography on silica gel (petroleum ether/EtOAc = 50:1) yielded **24** (48.7 mg, 83%, 99% D) as a colorless oil. ^{1}H NMR (400 MHz, Chloroform-*d*) δ 4.52 (s, 1H), 3.12 – 3.07 (m, 2H), 1.47 – 1.46 (m, 2H), 1.43 (s, 9H), 1.35 – 1.27 (m, 2H), 0.92 – 0.86 (m, 2H). ^{13}C NMR (100 MHz, Chloroform-*d*) δ 156.0, 78.9, 42.3, 28.4, 23.2, 10.9 (t, J = 20.0 Hz). HR-MS (ESI) m/z calc. for $C_9H_{19}DNO_2$ $[M+H]^+$: 175.1551, found: 175.1549.

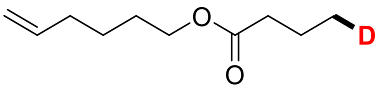

25

Pent-4-en-1-yl butanoate-4-*d*

Compound **25** was prepared following the general procedure, purification by column chromatography on silica gel (petroleum ether/EtOAc = 100:1) yielded **25** (59.0 mg, 69%, 99% D) as a colorless oil. ^{1}H NMR (400 MHz, Chloroform-*d*) δ 5.83 – 5.72 (m, 1H), 5.02 – 4.93 (m, 2H), 4.05 (t, J = 6.8 Hz, 2H), 2.26 (t, J = 7.2 Hz, 2H), 2.09 (q, J =

7.2 Hz, 2H), 1.60 – 1.59 (m, 4H), 1.47 – 1.40 (m, 2H), 0.93 – 0.89 (m, 2H). ^{13}C NMR (100 MHz, Chloroform-*d*) δ 173.7, 138.3, 114.8, 64.1, 36.2, 33.3, 28.1, 25.2, 18.4, 13.4 (t, *J* = 20.0 Hz). HR-MS (ESI) m/z calc. for $C_{10}H_{18}DO_2$ $[M+H]^+$: 172.1442, found: 172.1444.

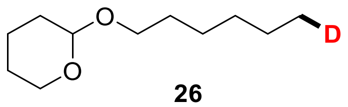

26

2-((Hexyl-6-*d*)oxy)tetrahydro-2*H*-pyran

Compound **26** was prepared following the general procedure, purification by column chromatography on silica gel (petroleum ether/EtOAc = 100:1) yielded **26** (65.5 mg, 70%, 99% D) as a colorless oil. ^{1}H NMR (400 MHz, Chloroform-*d*) δ 4.56 – 4.55 (m, 1H), 3.88 – 3.82 (m, 1H), 3.74 – 3.68 (m, 1H), 3.50 – 3.45 (m, 1H), 3.39 – 3.33 (m, 1H), 1.85 – 1.77 (m, 1H), 1.73 – 1.66 (m, 1H), 1.61 – 1.48 (m, 6H), 1.37 – 1.23 (m, 6H), 0.88 – 0.83 (m, 2H). ^{13}C NMR (100 MHz, Chloroform-*d*) δ 98.8, 67.7, 62.3, 31.7, 30.8, 29.7, 25.9, 25.5, 22.5, 19.7, 13.7 (t, *J* = 20.0 Hz). HR-MS (ESI) m/z calc. for $C_{11}H_{21}DNaO_2$ $[M+Na]^+$: 210.1575, found: 210.1572.

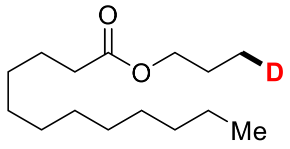

27

Propyl-3-*d* dodecanoate

Compound **27** was prepared following the general procedure, purification by column chromatography on silica gel (petroleum ether/EtOAc = 100:1) yielded **27** (109.3 mg, 90%, 99% D) as a colorless oil. ^{1}H NMR (400 MHz, Chloroform-*d*) δ 4.00 (t, *J* = 6.8 Hz, 2H), 2.27 (t, *J* = 7.2 Hz, 2H), 1.66 – 1.56 (m, 4H), 1.27 – 1.24 (m, 16H), 0.94 – 0.88 (m, 2H), 0.86 (t, *J* = 6.8 Hz, 3H).^{13}C NMR (100 MHz, Chloroform-*d*) δ 173.9, 65.8, 34.4, 31.9, 29.6, 29.5, 29.3, 29.3, 29.26, 29.2, 25.0, 22.7, 21.9, 14.1, 10.1 (t, *J* = 20.0 Hz). HR-MS (ESI) m/z calc. for $C_{15}H_{30}DO_2$ $[M+H]^+$: 244.2381, found: 244.2388.

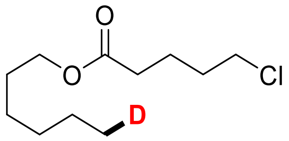

28

Hexyl-6-*d* 5-chloropentanoate

Compound **28** was prepared following the general procedure, purification by column chromatography on silica gel (petroleum ether/EtOAc = 100:1) yielded **28** (78.5 mg, 71%, 98% D) as a colorless oil. ^{1}H NMR (400 MHz, Chloroform-*d*) δ 4.07 (t, *J* = 6.8 Hz, 2H), 3.55 (t, *J* = 6.0 Hz, 2H), 2.34 (t, *J* = 6.8 Hz, 2H), 1.86 – 1.77 (m, 4H), 1.65 – 1.60 (m, 2H), 1.38 – 1.28 (m, 6H), 0.89 – 0.85 (m, 2H).^{13}C NMR (100 MHz, Chloroform-*d*) δ 173.3, 64.6, 44.5, 33.5, 31.9, 31.4, 28.6, 25.6, 22.4, 22.3, 13.7 (t, *J* = 20.0 Hz). HR-MS (ESI) m/z calc. for $C_{11}H_{21}DClO_2$ [M+H]$^+$: 222.1366, found: 222.1366.

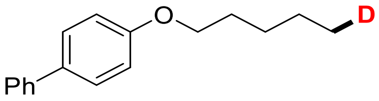

29

4-((pentyl-5-d)oxy)-1,1'-biphenyl

Compound **29** was prepared following the general procedure, purification by column chromatography on silica gel (petroleum ether/EtOAc = 100:1) yielded **29** (104.5 mg, 86%, 98% D) as a white solid. M.p.: 59–60 °C. ^{1}H NMR (400 MHz, Chloroform-*d*) δ 7.60 – 7.53 (m, 4H), 7.46 – 7.43 (m, 2H), 7.35 – 7.31 (m, 1H), 7.02 – 6.98 (m, 2H), 4.03 (t, *J* = 6.4 Hz, 2H), 1.88 – 1.81 (m, 2H), 1.54 – 1.39 (m, 4H), 1.00 – 0.94 (m, 2H). ^{13}C NMR (100 MHz, Chloroform-*d*) δ 158.8, 140.9, 133.6, 128.7, 128.1, 126.7, 126.6, 114.8, 68.1, 29.0, 28.2, 22.4, 13.8 (t, *J* = 20.0 Hz). HR-MS (ESI) m/z calc. for $C_{17}H_{20}DO$ [M+H]$^+$: 242.1650, found: 242.1657.

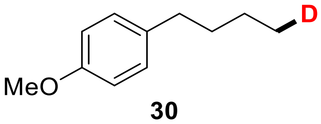

30

1-(Butyl-4-*d*)-4-methoxybenzene

Compound **30** was prepared following the general procedure, purification by column chromatography on silica gel (petroleum ether/EtOAc = 100:1) yielded **30** (54.5 mg, 66%, 97% D) as a colorless oil. ^{1}H NMR (400 MHz, Chloroform-*d*) δ 7.15 – 7.12 (m, 2H), 6.89 – 6.85 (m, 2H), 3.83 (s, 3H), 2.59 (d, *J* = 7.6 Hz, 2H), 1.65 – 1.57 (m, 2H), 1.42 – 1.35 (m, 2H), 0.98 – 0.92 (m, 2H).^{13}C NMR (100 MHz, Chloroform-*d*) δ 157.6, 135.0, 129.3, 113.7, 55.3, 34.8, 33.9, 22.3, 13.7 (t, *J* = 20.0 Hz). HR-MS (ESI) m/z calc. for $C_{11}H_{16}DO$ $[M+H]^+$: 166.1337, found: 166.1337.

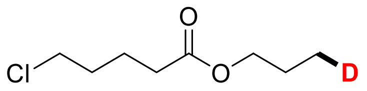

31

propyl-3-d 5-chloropentanoate

Compound **31** was prepared following the general procedure, purification by column chromatography on silica gel (petroleum ether/EtOAc = 100:1) yielded **31** (60.9 mg, 68%, 97% D) as a colorless oil. ^{1}H NMR (400 MHz, Chloroform-*d*) δ 4.04 (t, *J* = 6.4 Hz, 2H), 3.55 (t, *J* = 6.0 Hz, 2H), 2.35 (t, *J* = 7.2 Hz, 2H), 1.83 – 1.77 (m, 4H), 1.68 – 1.61 (m, 2H), 0.96 – 0.90 (m, 2H). ^{13}C NMR (100 MHz, Chloroform-d) δ 173.3, 66.0, 44.5, 33.5, 31.9, 22.3, 21.9, 10.1 (t, *J* = 20.0 Hz). HR-MS (ESI) m/z calc. for $C_8H_{15}DClO_2$ $[M+H]^+$: 180.0896, found: 180.0899.

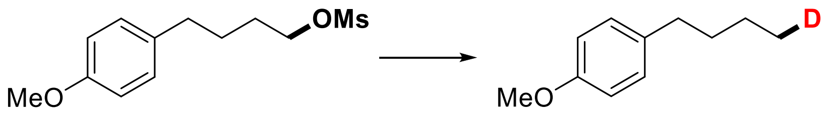

32

1-(butyl-4-d)-4-methoxybenzene

Compound **32** was prepared following the general procedure, purification by column chromatography on silica gel (petroleum ether/EtOAc = 100:1) yielded **32** (52.9 mg, 64%, 99% D) as a colorless oil. ^{1}H NMR (400 MHz, Chloroform-*d*) δ 7.19 – 7.03 (m, 2H), 6.90 – 6.76 (m, 2H), 3.80 (s, 3H), 2.64 – 2.51 (m, 2H), 1.67 – 1.50 (m, 2H), 1.43 – 1.30 (m, 2H), 0.96 – 0.90 (m, 2H). ^{13}C NMR (150 MHz, Chloroform-*d*) δ 157.7, 135.1, 129.4, 113.8, 55.4, 34.9, 34.0, 22.4, 13.9, 13.8, 13.7. HR-MS (ESI) m/z calc. for $C_{11}H_{16}DO$ $[M+H]^+$: 166.1337, found: 166.1339.

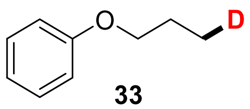

33

(Propoxy-3-*d*)benzene

Compound **33** was prepared following the general procedure, purification by column chromatography on silica gel (petroleum ether/EtOAc = 100:1) yielded **33** (48.0 mg, 70%, 99% D) as a colorless oil. ^{1}H NMR (400 MHz, Chloroform-*d*) δ 7.30 – 7.26 (m, 2H), 6.95 – 6.89 (m, 3H), 3.92 (t, *J* = 6.4 Hz, 2H), 1.84 – 1.77 (m, 2H), 1.06 – 1.00 (m, 2H). ^{13}C NMR (100 MHz, Chloroform-*d*) δ 159.1, 129.4, 120.5, 114.5, 69.4, 22.5, 10.3 (t, *J* = 20.0 Hz). HR-MS (ESI) m/z calc. for $C_9H_{12}DO$ $[M+H]^+$: 138.1024, found: 138.1024.

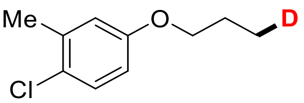

34

1-Chloro-2-methyl-4-(propoxy-3-*d*)benzene

Compound **34** was prepared following the general procedure, purification by column chromatography on silica gel (petroleum ether/EtOAc = 100:1) yielded **34** (68.5 mg, 74%, 99% D) as a colorless oil. ^{1}H NMR (400 MHz, Chloroform-*d*) δ 7.22 (d, *J* = 8.8 Hz, 1H), 6.78 (d, *J* = 2.8 Hz, 1H), 6.69 – 6.66 (m, 1H), 3.88 (t, *J* = 6.4 Hz, 2H), 2.35 (s, 3H), 1.83 – 1.76 (m, 2H), 1.06 – 1.01 (m, 2H). ^{13}C NMR (100 MHz, Chloroform-d) δ 157.7, 136.9, 129.5, 125.6, 117.1 (d, *J* = 6.6 Hz), 113.1 (d, *J* = 3.7 Hz), 69.7, 22.5, 20.3, 10.2 (t, *J* = 20.0 Hz). HR-MS (ESI) m/z calc. for $C_{10}H_{13}DClO$ $[M+H]^+$: 186.0790, found: 186.0797.

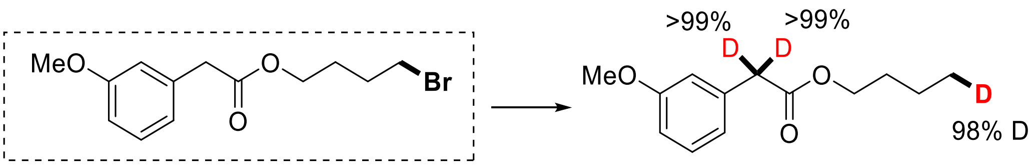

35

Butyl-4-*d* 2-(3-methoxyphenyl)acetate

Compound **35** was prepared following the general procedure, purification by column chromatography on silica gel (petroleum ether/EtOAc = 100:1) yielded **35** (94.5 mg, 84%, 98% D) as a colorless oil. ^{1}H NMR (400 MHz, Chloroform-*d*) δ 7.28 – 7.24 (m,

1H), 6.91 – 6.82 (m, 3H), 4.12 (t, *J* = 6.4 Hz, 2H), 3.83 (s, 3H), 1.67 – 1.60 (m, 2H), 1.41 – 1.34 (m, 2H), 0.96 – 0.90 (m, 2H). ^{13}C NMR (100 MHz, Chloroform-*d*) δ 171.6, 159.7, 135.6, 129.5, 121.6, 114.8, 112.7, 64.7, 55.2, 41.9 – 41.2 (m), 30.6, 19.0, 13.4 (t, *J* = 20.0 Hz). HR-MS (ESI) m/z calc. for $C_{13}H_{16}D_3O_3$ $[M+H]^+$: 226.1517, found: 226.1519.

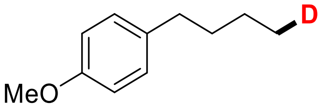

36

1-(Butyl-4-*d*)-4-methoxybenzene

Compound **36** was prepared following the general procedure, purification by column chromatography on silica gel (petroleum ether/EtOAc = 100:1) yielded **36** (58.6 mg, 71%, 98% D) as a colorless oil. ^{1}H NMR (400 MHz, Chloroform-*d*) δ 7.13 (d, *J* = 8.4 Hz, 2H), 6.86 (d, *J* = 8.4 Hz, 2H), 3.82 (s, 3H), 2.59 (t, *J* = 7.6 Hz, 2H), 1.64 – 1.57 (m, 2H), 1.42 – 1.34 (m, 2H), 0.98 – 0.92 (m, 2H). ^{13}C NMR (100 MHz, Chloroform-*d*) δ 157.6, 135.0, 129.3, 113.7, 55.3, 34.7, 33.9, 22.2, 13.7 (t, *J* = 20.0 Hz). HR-MS (ESI) m/z calc. for $C_{11}H_{16}DO$ $[M+H]^+$: 166.1337, found: 166.1338.

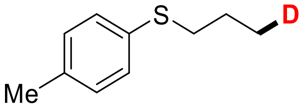

37

(Propyl-3-*d*)(*p*-tolyl)sulfane

Compound **37** was prepared following the general procedure, purification by column chromatography on silica gel (petroleum ether/EtOAc = 100:1) yielded **37** (55.9 mg, 67%, 97% D) as a colorless oil. ^{1}H NMR (400 MHz, Chloroform-*d*) δ 7.28 – 7.25 (m, 2H), 7.12 – 7.10 (m, 2H), 2.87 (d, *J* = 7.2 Hz, 2H), 2.33 (s, 3H), 1.69 – 1.62 (m, 2H), 1.04 – 0.98 (m, 2H). ^{13}C NMR (100 MHz, Chloroform-*d*) δ 135.9, 133.0, 129.9, 129.6, 36.4, 22.5, 21.0, 13.1 (t, *J* = 20.0 Hz). HR-MS (ESI) m/z calc. for $C_{10}H_{14}DS$ $[M+H]^+$: 168.0952, found: 168.0953.

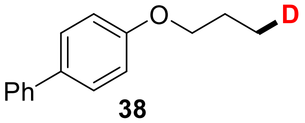

38

4-(Propoxy-3-*d*)-1,1'-biphenyl

Compound **38** was prepared following the general procedure, purification by column chromatography on silica gel (petroleum ether/EtOAc = 100:1) yielded **38** (85.7 mg, 80%, 99% D) as a white solid. M.p.: 65–66 °C. ^{1}H NMR (400 MHz, Chloroform-*d*) δ 7.60 – 7.54 (m, 4H), 7.47 – 7.43 (m, 2H), 7.35 – 7.31 (m, 1H), 7.03 – 6.99 (m, 2H), 4.00 (t, *J* = 6.4 Hz, 2H), 1.90 – 1.83 (m, 2H), 1.11 – 1.05 (m, 2H). ^{13}C NMR (100 MHz, Chloroform-*d*) δ 158.8, 140.9, 133.6, 128.8, 128.2, 126.8, 126.6, 114.8, 69.6, 22.6, 10.3 (t, *J* = 20.0 Hz). HR-MS (ESI) m/z calc. for $C_{15}H_{16}DO$ $[M+H]^{+}$: 214.1337, found: 214.1330. Spectroscopic data match those previously reported in the literature.[3]

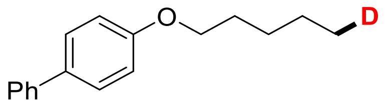

39

4-((pentyl-5-d)oxy)-1,1'-biphenyl

Compound **39** was prepared following the general procedure, purification by column chromatography on silica gel (petroleum ether/EtOAc = 100:1) yielded **39** (93.2 mg, 77%, 99% D) as a white solid. M.p.: 59–60 °C. ^{1}H NMR (400 MHz, Chloroform-*d*) δ 7.59 – 7.52 (m, 4H), 7.45 – 7.42 (m, 2H), 7.34 – 7.30 (m, 1H), 7.01 – 6.98 (m, 2H), 4.02 (t, *J* = 6.4 Hz, 2H), 1.87 – 1.80 (m, 2H), 1.53 – 1.40 (m, 4H), 0.99 – 0.93 (m, 2H). ^{13}C NMR (100 MHz, Chloroform-*d*) δ 158.8, 140.9, 133.6, 128.7, 128.1, 126.7, 126.6, 114.8, 68.1, 29.0, 28.2, 22.4, 13.8 (t, *J* = 20.0 Hz). HR-MS (ESI) m/z calc. for $C_{17}H_{20}DO$ $[M+H]^{+}$: 242.1650, found: 242.1653.

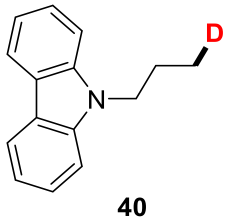

40

9-(Propyl-3-*d*)-9*H*-carbazole

Compound **40** was prepared following the general procedure, purification by column chromatography on silica gel (petroleum ether/EtOAc = 100:1) yielded **40** (98.1 mg, 93%, 99% D) as a yellow solid. M.p.: 55–56 °C. ^{1}H NMR (400 MHz, Chloroform-*d*) δ 8.21 (d, *J* = 7.6 Hz, 2H), 7.58 – 7.54 (m, 2H), 7.50 – 7.48 (m, 2H), 7.35 – 7.31 (m, 2H), 4.33 (t, *J* = 7.2 Hz, 2H), 2.02 – 1.95 (m, 2H), 1.077 – 1.02 (m, 2H). ^{13}C NMR (100

MHz, Chloroform-*d*) δ 140.6, 125.7, 122.9, 120.4, 118.8, 108.8, 44.6, 22.3, 11.6 (t, *J* = 20.0 Hz). HR-MS (ESI) m/z calc. for $C_{15}H_{15}DN$ $[M+H]^+$: 211.1340, found: 211.1340.

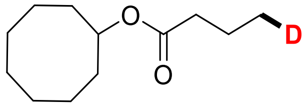

41

Cyclooctyl pentanoate-5-*d*

Compound **41** was prepared following the general procedure, purification by column chromatography on silica gel (petroleum ether/EtOAc = 100:1) yielded **41** (72.1 mg, 72%, 99% D) as a colorless oil. ^{1}H NMR (400 MHz, Chloroform-d) δ 4.90 – 4.84 (m, 1H), 2.17 (t, *J* = 7.4 Hz, 2H), 1.77 – 1.59 (m, 6H), 1.58 – 1.55 (m, 2H), 1.50 – 1.43 (m, 8H), 0.89 – 0.83 (m, 2H). ^{13}C NMR (100 MHz, Chloroform-*d*) δ 173.0, 74.7, 36.7, 31.5, 27.1, 25.4, 22.9, 18.5, 13.3 (t, *J* = 20.0 Hz). HR-MS (ESI) m/z calc. for $C_{12}H_{22}DO_2$ $[M+H]^+$: 200.1755, found: 200.1757.

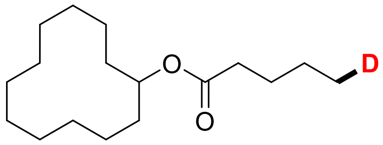

42

Cyclododecyl pentanoate-5-*d*

Compound **42** was prepared following the general procedure, purification by column chromatography on silica gel (petroleum ether/EtOAc = 100:1) yielded **42** (122.5 mg, 91%, 99% D) as a colorless oil. ^{1}H NMR (400 MHz, Chloroform-*d*) δ 5.03 – 4.97 (m, 1H), 2.25 (t, *J* = 7.6 Hz, 2H), 1.72 – 1.64 (m, 2H), 1.62 – 1.54 (m, 2H), 1.51 – 1.43 (m, 2H), 1.37 – 1.28 (m, 20H), 0.92 – 0.86 (m, 2H). ^{13}C NMR (100 MHz, Chloroform-*d*) δ 173.6, 71.8, 34.4, 29.1, 27.2, 24.1, 23.8, 23.4, 23.2, 22.2, 20.9, 13.4 (t, *J* = 20.0 Hz). HR-MS (ESI) m/z calc. for $C_{17}H_{32}DO_2$ $[M+H]^+$: 270.2538, found: 270.2535.

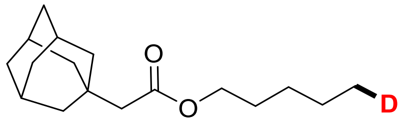

43

Pentyl-5-*d* 2-((1*s*,3*s*)-adamantan-1-yl)acetate

Compound **43** was prepared following the general procedure, purification by column chromatography on silica gel (petroleum ether/EtOAc = 100:1) yielded **43** (106.1 mg, 80%, 97% D) as a colorless oil. ^{1}H NMR (400 MHz, Chloroform-*d*) δ 4.02 (t, *J* = 6.4 Hz, 2H), 2.04 (s, 2H), 1.95 (s, 3H), 1.70 – 1.59 (m, 14H), 1.34 – 1.30 (m, 4H), 0.89 – 0.85 (m, 2H). ^{13}C NMR (100 MHz, Chloroform-*d*) δ 171.9, 64.0, 49.0, 42.4, 36.7, 32.7, 28.6, 28.4, 28.1, 22.2, 13.7 (t, *J* = 20.0 Hz). HR-MS (ESI) m/z calc. for $C_{17}H_{28}DO_2$ [M+H]$^+$: 266.2225, found: 266.2225.

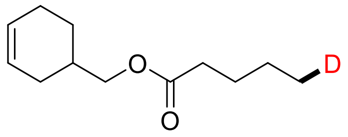

44

Cyclohex-3-en-1-yl pentanoate-5-*d*

Compound **44** was prepared following the general procedure, purification by column chromatography on silica gel (petroleum ether/EtOAc = 100:1) yielded **44** (73.5 mg, 75%, 99% D) as a colorless oil. ^{1}H NMR (400 MHz, Chloroform-*d*) δ 5.71 – 5.63 (m, 2H), 3.97 (d, *J* = 6.8 Hz, 2H), 2.32 (t, *J* = 7.6 Hz, 2H), 2.14 – 2.05 (m, 3H), 1.98 – 1.90 (m, 1H), 1.81 – 1.73 (m, 2H), 1.65 – 1.58 (m, 2H), 1.38 – 1.26 (m, 3H), 0.94 – 0.88 (m, 2H). ^{13}C NMR (100 MHz, Chloroform-*d*) δ 174.0, 127.0, 125.5, 68.5, 34.1, 33.0, 28.2, 27.1, 25.3, 24.4, 22.2, 13.4 (t, *J* = 20.0 Hz). HR-MS (ESI) m/z calc. for $C_{12}H_{20}DO_2$ [M+H]$^+$: 198.1599, found: 198.1598.

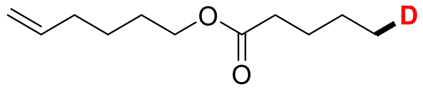

45

Hex-5-en-1-yl pentanoate-5-*d*

Compound **45** was prepared following the general procedure, purification by column chromatography on silica gel (petroleum ether/EtOAc = 100:1) yielded **45** (73.1 mg, 79%, 99% D) as a colorless oil. ^{1}H NMR (400 MHz, Chloroform-*d*) δ 5.84 – 5.74 (m, 1H), 5.03 – 4.94 (m, 2H), 4.06 (t, *J* = 6.4 Hz, 2H), 2.29 (t, *J* = 7.2 Hz, 2H), 2.10 – 2.05 (m, 2H), 1.65 – 1.56 (m, 4H), 1.48 – 1.41 (m, 2H), 1.36 – 1.29 (m, 2H), 0.92 – 0.86 (m, 2H). ^{13}C NMR (100 MHz, Chloroform-*d*) δ 174.0, 138.4, 114.8, 64.1, 34.1, 33.3, 28.1,

27.1, 25.2, 22.2, 13.4 (t, J = 20.0 Hz). HR-MS (ESI) m/z calc. for $C_{11}H_{20}DO_2$ $[M+H]^+$: 186.1599, found: 186.1598.

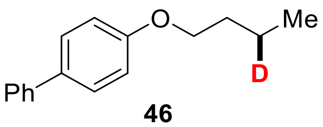

46

4-(Butoxy-3-*d*)-1,1'-biphenyl

Compound **46** was prepared following the general procedure, purification by column chromatography on silica gel (petroleum ether/EtOAc = 100:1) yielded **46** (68.1 mg, 60%, 91% D) as a white solid. M.p.: 59–60 °C. ^{1}H NMR (400 MHz, Chloroform-*d*) δ 7.59 – 7.53 (m, 4H), 7.45 – 7.41 (m, 2H), 7.32 (t, J = 7.2 Hz, 1H), 7.01 – 6.97 (m, 2H), 4.03 (t, J = 6.4 Hz, 2H), 1.84 – 1.78 (m, 2H), 1.57 – 1.50 (m, 1H), 1.04 – 1.00 (m, 3H). ^{13}C NMR (100 MHz, Chloroform-*d*) δ 158.8, 140.9, 133.6, 128.7, 128.1, 126.7, 126.6, 114.8, 67.8, 31.3, 19.1, 18.7 (t, J = 20.0 Hz), 13.8. HR-MS (ESI) m/z calc. for $C_{16}H_{18}DO$ $[M+H]^+$: 228.1493, found: 228.1494.

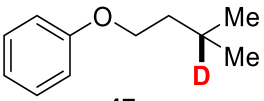

47

(3-Methylbutoxy-3-*d*)benzene

Compound **47** was prepared following the general procedure, purification by column chromatography on silica gel (petroleum ether/EtOAc = 100:1) yielded **47** (50.4 mg, 61%, 93% D) as a colorless oil. ^{1}H NMR (400 MHz, Chloroform-*d*) δ 7.32 – 7.28 (m, 2H), 6.97 – 6.91 (m, 3H), 4.00 (t, J = 6.8 Hz, 2H), 1.70 (t, J = 6.8 Hz, 2H), 0.98 (s, 6H). ^{13}C NMR (100 MHz, Chloroform-*d*) δ 159.2, 129.4, 120.5, 114.5, 66.2, 38.0, 24.6 (t, J = 20.0 Hz), 22.5. HR-MS (ESI) m/z calc. for $C_{11}H_{16}DO$ $[M+H]^+$: 166.1337, found: 166.1330.

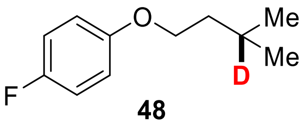

48

1-Fluoro-4-(3-methylbutoxy-3-*d*)benzene

Compound **48** was prepared following the general procedure, purification by column chromatography on silica gel (petroleum ether/EtOAc = 100:1) yielded **48** (65.9 mg,

72%, 93% D) as a colorless oil. ^{1}H NMR (400 MHz, Chloroform-*d*) δ 7.00 – 6.94 (m, 2H), 6.86 – 6.81 (m, 2H), 3.94 (t, *J* = 6.4 Hz, 2H), 1.66 (t, *J* = 6.4 Hz, 2H), 0.96 (s, 6H). ^{13}C NMR (100 MHz, Chloroform-*d*) δ157.1 (d, $^{1}J_{C-F}$ = 236.2 Hz), 155.3 (d, $^{4}J_{C-F}$ = 2.3 Hz), 115.7 (d, $^{2}J_{C-F}$ = 22.8 Hz), 115.4 (d, $^{3}J_{C-F}$ = 7.8 Hz), 67.0, 37.9, 24.8, 24.4 (t, *J* = 20 Hz). ^{19}F NMR (375 MHz, Chloroform-*d*) δ -124.5. HR-MS (ESI) m/z calc. for $C_{11}H_{15}DFO$ $[M+H]^+$: 184.1242, found: 184.1242.

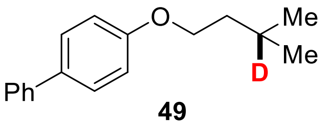

49

4-(3-Methylbutoxy-3-*d*)-1,1'-biphenyl

Compound **49** was prepared following the general procedure, purification by column chromatography on silica gel (petroleum ether/EtOAc = 100:1) yielded **49** (84.4 mg, 70%, 89% D) as a white solid. M.p.: 68–69 °C. ^{1}H NMR (400 MHz, Chloroform-d) δ 7.58 – 7.53 (m, 4H), 7.45 – 7.41 (m, 2H), 7.32 (t, J = 7.3 Hz, 1H), 6.99 (d, J = 8.7 Hz, 2H), 4.05 (t, J = 6.7 Hz, 2H), 1.72 (t, J = 6.6 Hz, 2H), 1.00 (s, 6H). ^{13}C NMR (100 MHz, Chloroform-*d*) δ 158.7, 140.9, 133.6, 128.7, 128.1, 126.7, 126.6, 114.8, 66.4, 37.9, 24.5 (t, *J* = 20.0 Hz), 22.5. HR-MS (ESI) m/z calc. for $C_{17}H_{20}DO$ $[M+H]^+$: 242.1650, found: 242.1655. Spectroscopic data match those previously reported in the literature.[3]

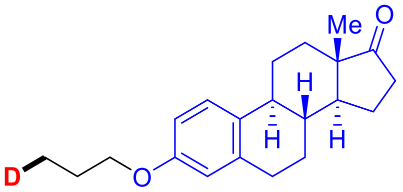

50

(8*R*,9*S*,13*S*,14*S*)-13-methyl-3-(propoxy-3-*d*)-6,7,8,9,11,12,13,14,15,16-decahydro-17*H*-cyclopenta[*a*]phenanthren-17-one

Compound **50** was prepared following the general procedure, purification by column chromatography on silica gel (petroleum ether/EtOAc = 20:1) yielded **50** (128.4 mg, 82%, 98% D) as a white solid. M.p.: 92–93 °C. ^{1}H NMR (400 MHz, Chloroform-*d*) δ 7.20 (d, *J* = 7.6 Hz, 1H), 6.73 (dd, *J* = 8.8, 2.8 Hz, 1H), 6.66 (d, *J* = 2.8 Hz, 1H), 3.90 (t, *J* = 6.4 Hz, 2H), 2.93 – 2.89 (m, 2H), 2.54 – 2.48 (m, 1H), 2.42 – 2.39 (m, 1H), 2.27

– 2.22 (m, 1H), 2.19 – 1.95 (m, 4H), 1.83 – 1.76 (m, 2H), 1.68 – 1.41 (m, 6H), 1.06 – 1.00 (m, 2H), 0.92 (s, 3H). ^{13}C NMR (100 MHz, Chloroform-d) δ 221.0, 157.2, 137.7, 131.8, 126.3, 114.5 (d, *J* = 6.4 Hz), 112.2 –112.0 (m), 69.4, 50.4, 48.0, 44.0, 38.4, 35.9, 31.6, 29.7, 26.6, 26.0, 22.6, 21.6, 13.9, 10.4 (t, *J* = 20.0 Hz). HR-MS (ESI) m/z calc. for $C_{21}H_{28}DO_2$ [M+H]$^+$: 314.2225, found: 314.2225.

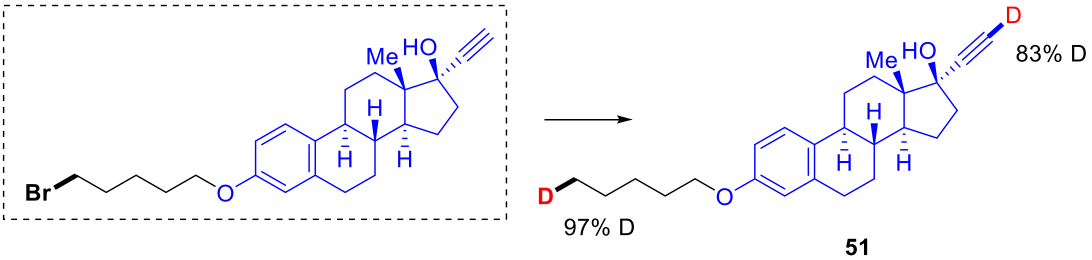

51

(8*R*,9*S*,13*S*,14*S*,17*R*)-17-(ethynyl-*d*)-13-methyl-3-((pentyl-5-*d*)oxy)-7,8,9,11,12,13,14,15,16,17-decahydro-6*H*-cyclopenta[*a*]phenanthren-17-ol

Compound **51** was prepared following the general procedure, purification by column chromatography on silica gel (petroleum ether/EtOAc = 20:1) yielded **51** (138.2 mg, 75%, 97% D) as a white solid. M.p.: 101–102 °C. ^{1}H NMR (400 MHz, Chloroform-*d*) δ 7.21 (d, *J* = 8.4 Hz, 1H), 6.72 (dd, *J* = 8.4, 2.8 Hz, 1H), 6.64 (d, *J* = 2.8 Hz, 1H), 3.93 (t, *J* = 6.4 Hz, 2H), 2.88 – 2.84 (m, 2H), 2.40 – 2.31 (m, 2H), 2.27 – 2.21 (m, 1H), 2.06 – 1.96 (m, 2H), 1.93 – 1.86 (m, 2H), 1.82 – 1.70 (m, 5H), 1.55 – 1.35 (m, 8H), 0.95 – 0.89 (m, 5H). ^{13}C NMR (100 MHz, Chloroform-*d*) δ 157.0, 137.9, 132.3, 126.3, 114.5 (d, *J* = 7.3, 2.8 Hz), 112.04 (d, *J* = 3.0), 79.9, 74.0 (m), 67.9, 49.5, 47.2, 43.6, 39.5, 39.0, 32.8, 29.9, 29.1, 28.2, 27.3, 26.4, 22.8, 22.4, 13.8 (t, *J* = 20.0 Hz), 12.7. HR-MS (ESI) m/z calc. for $C_{25}H_{33}D_2O_2$ [M+H]$^+$: 369.2757, found: 369.2757.

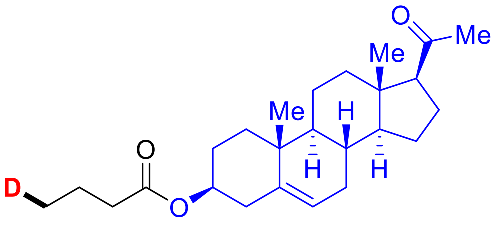

52

(3*S*,8*S*,9*S*,10*R*,13*S*,14*S*,17*S*)-17-acetyl-10,13-dimethyl 2,3,4,7,8,9,10,11,12,13,14,15,16,17-tetradecahydro-1*H*-cyclopenta[*a*]phenanthren-3-yl butanoate-4-*d*

Compound **52** was prepared following the general procedure, purification by column chromatography on silica gel (petroleum ether/EtOAc = 20:1) yielded **52** (160.7 mg, 83%, 98% D) as a white solid. M.p.: 81–82 °C. ^{1}H NMR (400 MHz, Chloroform-*d*) δ 5.34 – 5.32 (m, 1H), 4.62 – 4.54 (m, 1H), 2.50 (t, *J* = 8.8 Hz, 1H), 2.29 – 2.27 (m, 2H), 2.22 (t, *J* = 7.2 Hz, 2H), 2.17 – 2.12 (m, 1H), 2.10 –2.07 (m, 3H), 2.02 – 1.89 (m, 2H), 1.86 – 1.80 (m, 2H), 1.69 – 1.49 (m, 7H), 1.46 – 1.38 (m, 3H), 1.24 – 1.07 (m, 3H), 1.01 – 0.95 (m, 4H), 0.92 – 0.86 (m, 2H), 0.59 (s, 3H).^{13}C NMR (100 MHz, Chloroform-*d*) δ 209.5, 173.1, 139.7, 122.3, 73.5, 63.7, 56.8, 49.9, 44.0, 38.8, 38.1, 37.0, 36.6, 36.5, 31.8, 31.75, 31.6, 27.8, 24.5, 22.8, 21.0, 19.3, 18.4, 13.3 (t, *J* = 20.0 Hz), 13.21. HR-MS (ESI) m/z calc. for $C_{25}H_{38}DO_3$ [M+H]$^+$: 388.2956, found: 388.2955.

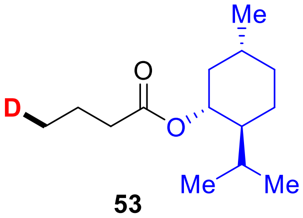

53

(1*R*,2*S*,5*R*)-2-Isopropyl-5-methylcyclohexyl butanoate-4-*d*

Compound **53** was prepared following the general procedure, purification by column chromatography on silica gel (petroleum ether/EtOAc = 100:1) yielded **53** (92.0 mg, 81%, 98% D) as a colorless oil. ^{1}H NMR (400 MHz, Chloroform-*d*) δ 4.72 – 4.65 (m, 1H), 2.27 (t, *J* = 7.2 Hz, 2H), 2.02 – 1.96 (m, 1H), 1.91 – 1.84 (m, 1H), 1.70 – 1.62 (m, 4H), 1.56 – 1.43 (m, 1H), 1.41 – 1.34 (m, 1H), 1.11 – 1.04 (m, 1H), 1.00 – 0.93 (m, 3H), 0.91 – 0.89 (m, 6H), 0.86 – 0.82 (m, 1H), 0.76 (d, *J* = 7.2 Hz, 3H). ^{13}C NMR (100 MHz, Chloroform-*d*) δ 173.3, 73.8, 47.0, 41.0, 36.6, 34.3, 31.4, 26.2, 23.4, 22.0, 20.7, 18.5, 16.3, 13.3 (t, *J* = 20.0 Hz). HR-MS (ESI) m/z calc. for $C_{14}H_{25}DNaO_2$ [M+Na]$^+$: 250.1888, found: 250.1878.

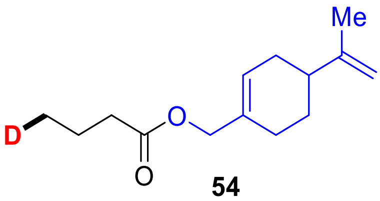

54

(4-(Prop-1-en-2-yl)cyclohex-1-en-1-yl)methyl butanoate-4-*d*

Compound **54** was prepared following the general procedure, purification by column

chromatography on silica gel (petroleum ether/EtOAc = 100:1) yielded **54** (78.1 mg, 70%, 97% D) as a colorless oil. ^{1}H NMR (400 MHz, Chloroform-d) δ 5.79 – 5.64 (m, 1H), 4.70 (d, *J* = 6.2 Hz, 2H), 4.45 (s, 2H), 2.29 (t, *J* = 7.4 Hz, 2H), 2.21 – 2.02 (m, 4H), 1.96 – 1.92 (m, 1H), 1.88 – 1.78 (m, 1H), 1.72 (s, 3H), 1.68 – 1.58 (m, 2H), 1.55 – 1.40 (m, 1H), 0.93 – 0.90 (m, 2H). ^{13}C NMR (100 MHz, Chloroform-d) δ 173.6, 149.6, 132.7, 125.6, 108.8, 68.2, 40.8, 36.2, 30.5, 27.3, 26.4, 20.7, 18.4, 13.4 (t, *J* = 20.0 Hz). HR-MS (ESI) m/z calc. for $C_{14}H_{22}DO_2$ $[M+H]^+$: 224.1755, found: 224.1755.

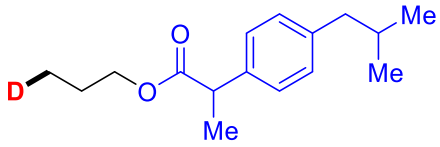

55

Propyl-3-*d* 2-(4-isobutylphenyl)propanoate

Compound **55** was prepared following the general procedure, purification by column chromatography on silica gel (petroleum ether/EtOAc = 100:1) yielded **55** (83.8 mg, 67%, 98% D) as a colorless oil. ^{1}H NMR (400 MHz, Chloroform-*d*) δ 7.26 – 7.24 (m, 2H), 7.14 – 7.12 (m, 2H), 4.06 (t, *J* = 6.4 Hz, 2H), 3.76 – 3.69 (m, 1H), 2.48 (d, *J* = 7.2 Hz, 2H), 1.93 – 1.83 (m, 1H), 1.66 – 1.59 (m, 2H), 1.53 (d, *J* = 7.2 Hz, 3H), 0.93 (d, *J* = 6.8 Hz, 6H), 0.90 – 0.84 (m, 2H). ^{13}C NMR (100 MHz, Chloroform-*d*) δ 174.8, 140.4, 137.9, 129.3, 127.2, 127.2, 66.2, 45.2, 45.1, 30.2, 22.4, 21.9, 18.5, 10.0 (t, *J* = 20.0 Hz). HR-MS (ESI) m/z calc. for $C_{16}H_{24}DO_2$ $[M+H]^+$: 250.1912, found: 250.1921.

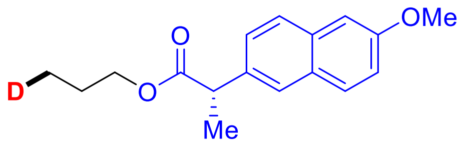

56

Propyl-3-*d* (*S*)-2-(6-methoxynaphthalen-2-yl)propanoate

Compound **56** was prepared following the general procedure, purification by column chromatography on silica gel (petroleum ether/EtOAc = 50:1) yielded **56** (120.3 mg, 88%, 98% D) as a white solid. M.p.: 53–54 °C. ^{1}H NMR (400 MHz, Chloroform-*d*) δ 7.73 – 7.69 (m, 3H), 7.44 (dd, *J* = 8.4, 1.6 Hz, 1H), 7.18 – 7.12 (m, 2H), 4.08 – 4.04 (m, 2H), 3.91 (s, 3H), 3.90 – 3.85 (m, 1H), 1.64 – 1.57 (m, 5H), 0.89 – 0.83 (m, 2H). ^{13}C NMR (100 MHz, Chloroform-*d*) δ 174.8, 157.6, 135.9, 133.7, 129.3, 129.0, 127.1,

126.3, 125.9, 119.0, 105.6, 66.3, 55.3, 45.6, 21.9, 18.6, 10.1 (t, J = 20.0 Hz). HR-MS (ESI) m/z calc. for $C_{17}H_{20}DO_3$ $[M+H]^+$: 274.1548, found: 274.1546.

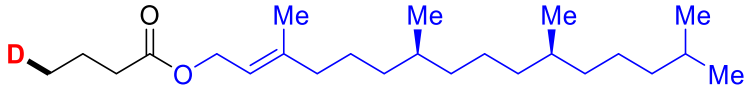

57

(7*S*,11*S*,*E*)-3,7,11,15-Tetramethylhexadec-2-en-1-yl butanoate-4-*d*

Compound **57** was prepared following the general procedure, purification by column chromatography on silica gel (petroleum ether/EtOAc = 50:1) yielded **57** (141.5 mg, 77%, 97% D) as a colorless oil. ^{1}H NMR (400 MHz, Chloroform-*d*) δ 5.35 – 5.31 (m, 1H), 4.59 (d, J = 7.2 Hz, 2H), 2.28 (t, J = 7.2 Hz, 2H), 2.00 (t, J = 8.0 Hz, 2H), 1.69 (s, 3H), 1.67 – 1.61 (m, 2H), 1.54 – 1.51 (m, 1H), 1.42 – 1.37 (m, 4H), 1.30 – 1.23 (m, 8H), 1.17 – 1.12 (m, 2H), 1.09 – 1.04 (m, 4H), 0.96 – 0.91 (m, 2H), 0.87 – 0.83 (m, 12H). ^{13}C NMR (100 MHz, Chloroform-*d*) δ 173.8, 142.6, 118.2, 61.2, 39.9, 39.4, 37.4, 37.36, 37.3, 36.6, 36.3, 32.8, 32.7, 28.0, 25.0, 24.8, 24.5, 22.7, 22.6, 19.75, 19.71, 18.4, 16.4, 13.4 (t, J = 20.0 Hz). HR-MS (ESI) m/z calc. for $C_{24}H_{46}DO_2$ $[M+H]^+$: 368.3633, found: 368.3634.

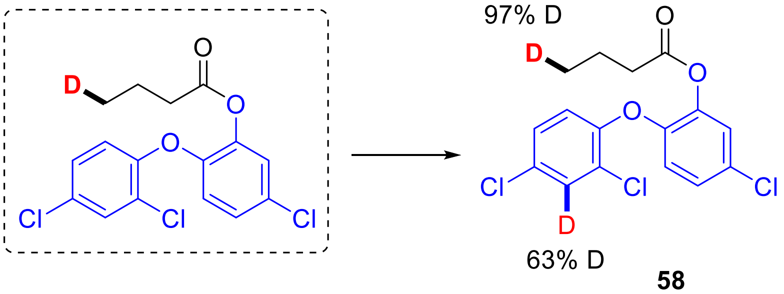

58

5-Chloro-2-(2,4-dichlorophenoxy-3-*d*)phenyl butanoate-4-*d*

Compound **58** was prepared following the general procedure, purification by column chromatography on silica gel (petroleum ether/EtOAc = 50:1) yielded **58** (127.8 mg, 71%, 97% D) as a colorless oil. ^{1}H NMR (400 MHz, Chloroform-*d*) δ 7.28 – 7.26 (m, 1H), 7.20 – 7.15 (m, 2H), 6.92 – 6.89 (m, 1H), 6.87 – 6.83 (m, 1H), 2.48 – 2.40 (m, 2H), 1.72 – 1.62 (m, 2H), 0.96 – 0.90 (m, 2H). HR-MS (ESI) m/z calc. for $C_{16}H_{12}D_2Cl_3O_3$ $[M+H]^+$: 361.0129, found: 361.0124.

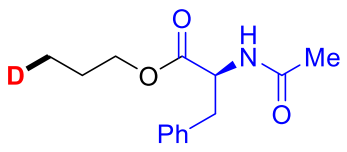

59

Propyl-3-*d* acetyl-*L*-phenylalaninate

Compound **59** was prepared following the general procedure, purification by column chromatography on silica gel (petroleum ether/EtOAc = 4:1) yielded **59** (92.6 mg, 74%, 99% D) as a colorless oil. ^{1}H NMR (400 MHz, Chloroform-*d*) δ 7.26 – 7.17 (m, 3H), 7.09 – 7.06 (m, 2H), 6.10 (d, *J* = 7.6 Hz, 1H), 4.86 – 4.81 (m, 1H), 4.07 – 3.96 (m, 2H), 3.12 – 3.02 (m, 2H), 1.93 (s, 3H), 1.62 – 1.55 (m, 2H), 0.88 – 0.83 (m, 2H). ^{13}C NMR (100 MHz, Chloroform-*d*) δ 171.8, 169.7, 136.0, 129.3, 128.5, 127.0, 67.0, 53.2, 38.0, 23.1, 21.7, 10.0 (t, *J* = 20.0 Hz). HR-MS (ESI) m/z calc. for $C_{14}H_{19}DNO_3$ $[M+H]^+$: 251.1500, found: 251.1503.

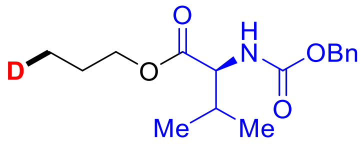

60

Propyl-3-*d* ((benzyloxy)carbonyl)-*L*-valinate

Compound **60** was prepared following the general procedure, purification by column chromatography on silica gel (petroleum ether/EtOAc = 4:1) yielded **60** (122.2 mg, 83%, 97% D) as a colorless oil. ^{1}H NMR (400 MHz, Chloroform-*d*) δ 7.37 – 7.31 (m, 5H), 5.32 (d, *J* = 8.8 Hz, 1H), 5.11 (s, 2H), 4.32 – 4.29 (m, 1H), 4.14 – 4.06 (m, 2H), 2.21 – 2.14 (m, 1H), 1.69 – 1.62 (m, 2H), 0.97 (d, *J* = 6.8 Hz, 3H), 0.95 – 0.92 (m, 2H), 0.90 (d, *J* = 7.2 Hz, 3H). ^{13}C NMR (100 MHz, Chloroform-*d*) δ 172.1, 156.3, 136.3, 128.5, 128.2, 128.1, 67.0, 66.9, 59.1, 31.4, 21.9, 19.0, 17.5, 10.1 (t, *J* = 20.0 Hz). HR-MS (ESI) m/z calc. for $C_{16}H_{23}DNO_4$ $[M+H]^+$: 295.1763, found: 295.1767.

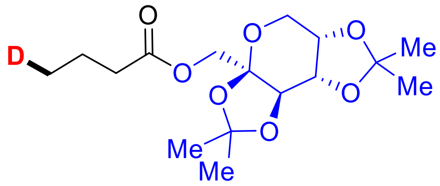

61

((3a*R*,5a*S*,8a*S*,8b*R*)-2,2,7,7-Tetramethyltetrahydro-3a*H*-bis([1,3]dioxolo)[4,5-*b*:4',5'-*d*]pyran-3a-yl)methyl butanoate-4-*d*

Compound **61** was prepared following the general procedure, purification by column chromatography on silica gel (petroleum ether/EtOAc = 20:1) yielded **61** (125.9 mg, 76%, 99% D) as a colorless oil. ^{1}H NMR (400 MHz, Chloroform-*d*) δ 4.58 (dd, *J* = 8.0, 2.4 Hz, 1H), 4.37 (d, *J* = 12.0 Hz, 1H), 4.28 (d, *J* = 2.4 Hz, 1H), 4.23 – 4.20 (m, 1H), 4.01 (d, *J* = 11.6 Hz, 1H), 3.88 (dd, *J* = 13.2, 2.0 Hz, 1H), 3.74 (dd, *J* = 13.2, 1.2 Hz, 1H), 2.31 (t, *J* = 7.2 Hz, 2H), 1.68 – 1.60 (m, 2H), 1.52 (s, 3H), 1.45 (s, 3H), 1.38 (s, 3H), 1.31 (s, 3H), 0.94 – 0.88 (m, 2H). ^{13}C NMR (100 MHz, Chloroform-*d*) δ 172.8, 109.1, 108.7, 101.6, 70.8, 70.5, 70.1, 65.0, 61.2, 35.9, 26.5, 25.9, 25.2, 24.0, 18.1, 13.4 (t, *J* = 20.0 Hz). HR-MS (ESI) m/z calc. for $C_{16}H_{26}DO_7$ $[M+H]^+$: 332.1814, found: 332.1817.

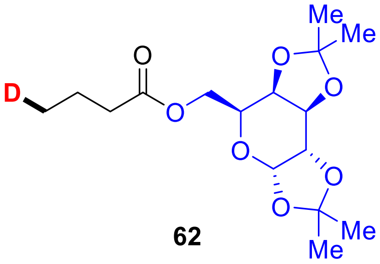

62

((3a*S*,5*S*,5a*R*,8a*R*,8b*S*)-2,2,7,7-Tetramethyltetrahydro-5*H*-bis([1,3]dioxolo)[4,5-*b*:4',5'-*d*]pyran-5-yl)methyl butanoate-4-*d*

Compound **62** was prepared following the general procedure, purification by column chromatography on silica gel (petroleum ether/EtOAc = 20:1) yielded **62** (139.1 mg, 84%, 98% D) as a colorless oil. ^{1}H NMR (400 MHz, Chloroform-*d*) δ 5.51 (d, *J* = 5.0 Hz, 1H), 4.59 (dd, *J* = 8.0, 2.4 Hz, 1H), 4.32 – 4.28 (m, 2H), 4.21 (dd, *J* = 8.0, 2.0 Hz, 1H), 4.16 – 4.11 (m, 1H), 4.01 – 3.98 (m, 1H), 2.30 (t, *J* = 7.2 Hz, 2H), 1.66 – 1.59 (m, 2H), 1.48 (s, 3H), 1.42 (s, 3H), 1.31 (d, *J* = 3.2 Hz, 6H), 0.94 – 0.88 (m, 2H). ^{13}C NMR (100 MHz, Chloroform-*d*) δ 173.6, 109.6, 108.7, 96.3, 71.1, 70.7, 70.4, 66.0, 63.2, 36.0, 26.0, 25.9, 24.9, 24.5, 18.4, 13.3 (t, *J* = 20.0 Hz). HR-MS (ESI) m/z calc. for $C_{16}H_{25}DNaO_7$ $[M+Na]^+$: 354.1634, found: 354.1635.

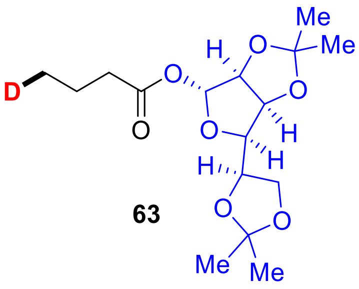

63

(3a*S*,4*R*,6*R*,6a*S*)-6-((*R*)-2,2-Dimethyl-1,3-dioxolan-4-yl)-2,2-dimethyltetrahydrofuro[3,4-*d*][1,3]dioxol-4-yl butanoate-4-*d*

Compound **63** was prepared following the general procedure, purification by column chromatography on silica gel (petroleum ether/EtOAc = 20:1) yielded **63** (134.1 mg, 81%, 99% D) as a colorless oil. ^{1}H NMR (400 MHz, Chloroform-*d*) δ 6.14 (s, 1H), 4.86 (dd, *J* = 5.6, 3.6 Hz, 1H), 4.69 (d, *J* = 6.0 Hz, 1H), 4.42 – 4.38 (m, 1H), 4.12 – 4.08 (m, 1H), 4.04 – 3.99 (m, 2H), 2.35 – 2.23 (m, 2H), 1.68 – 1.61 (m, 2H), 1.49 (s, 3H), 1.46 (s, 3H), 1.38 (s, 3H), 1.34 (s, 3H), 0.97 – 0.91 (m, 2H). ^{13}C NMR (100 MHz, Chloroform-*d*) δ 172.0, 113.2, 109.3, 100.5, 85.1, 82.2, 79.3, 72.9, 66.9, 36.1, 27.0, 25.9, 25.1, 24.7, 18.1, 13.2 (t, *J* = 20.0 Hz). HR-MS (ESI) m/z calc. for $C_{16}H_{26}DO_7$ $[M+H]^+$: 332.1814, found: 332.1817.

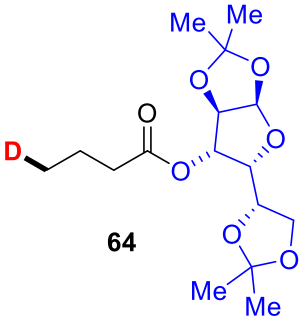

64

(3a*R*,5*R*,6*S*,6a*R*)-5-((*S*)-2,2-Dimethyl-1,3-dioxolan-4-yl)-2,2-dimethyltetrahydrofuro[2,3-*d*][1,3]dioxol-6-yl butanoate-4-*d*

Compound **64** was prepared following the general procedure, purification by column chromatography on silica gel (petroleum ether/EtOAc = 20:1) yielded **64** (124.2 mg, 75%, 99% D) as a colorless oil. ^{1}H NMR (400 MHz, Chloroform-*d*) δ 5.83 (d, *J* = 3.6 Hz, 1H), 5.23 (d, *J* = 2.0 Hz, 1H), 4.44 (d, *J* = 3.6 Hz, 1H), 4.20 – 4.15 (m, 2H), 4.06 – 4.03 (m, 1H), 3.98 – 3.95 (m, 1H), 2.35 – 2.23 (m, 2H), 1.66 – 1.59 (m, 2H), 1.48 (s, 3H), 1.36 (s, 3H), 1.27 (s, 6H), 0.94 – 0.88 (m, 2H). ^{13}C NMR (100 MHz, Chloroform-*d*) δ 172.1, 112.2, 109.3, 105.0, 83.4, 79.9, 75.8, 72.4, 67.3, 36.1, 26.8, 26.7, 26.2, 25.2,

18.3, 13.3 (t, J = 20.0 Hz). HR-MS (ESI) m/z calc. for $C_{16}H_{26}DO_7$ $[M+H]^+$: 332.1814, found: 332.1814.

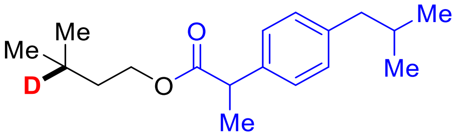

65

3-methylbutyl-3-d 2-(4-isobutylphenyl)propanoate

Compound **65** was prepared following the general procedure, purification by column chromatography on silica gel (petroleum ether/EtOAc = 100:1) yielded **65** (112.2 mg, 81%, 90% D) as a colorless oil. ^{1}H NMR (400 MHz, Chloroform-d) δ 7.21 (d, J = 8.1 Hz, 2H), 7.09 (d, J = 8.1 Hz, 2H), 4.10 (t, J = 6.8 Hz, 2H), 3.68 (q, J = 7.2 Hz, 1H), 2.46 (d, J = 7.2 Hz, 2H), 1.90 – 1.80 (m, 1H), 1.50 (s, 2H), 1.48 – 1.41 (m, 3H), 0.90 (d, J = 6.6 Hz, 6H), 0.85 (d, J = 5.3 Hz, 6H).^{13}C NMR (101 MHz, Chloroform-d) δ 174.8, 140.4, 137.9, 129.3, 127.2, 63.30, 45.2, 45.1, 37.1, 30.2, 24.7, 24.5, 24.3, 22.4, 22.3, 22.3, 18.5. HR-MS (ESI) m/z calc. for $C_{18}H_{28}DO_2$ $[M+H]^+$: 278.2225, found: 278.2229.

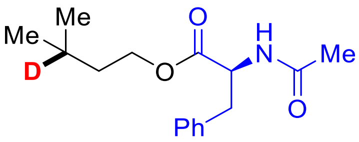

66

3-methylbutyl-3-d acetyl-L-phenylalaninate

Compound **66** was prepared following the general procedure, purification by column chromatography on silica gel (petroleum ether/EtOAc = 4:1) yielded **66** (97.4 mg, 70%, 90% D) as a colorless oil. ^{1}H NMR (400 MHz, Chloroform-d) δ 7.30 – 7.23 (m, 3H), 7.11 – 7.09 (m, 2H), 5.98 (d, J = 8.0 Hz, 1H), 4.89 – 4.84 (m, 1H), 4.16 – 4.06 (m, 2H), 3.15 – 3.06 (m, 2H), 1.97 (s, 3H), 1.48 (t, J = 6.8 Hz, 2H), 0.89 (s, 6H). ^{13}C NMR (100 MHz, Chloroform-d) δ 171.8, 169.6, 135.9, 129.3, 128.5, 127.1, 64.1, 53.2, 38.0, 37.0, 24.5 (t, J = 20.0 Hz), 23.2, 22.3, 22.2. HR-MS (ESI) m/z calc. for $C_{16}H_{23}DNO_3$ $[M+H]^+$: 279.1813, found: 279.1805.

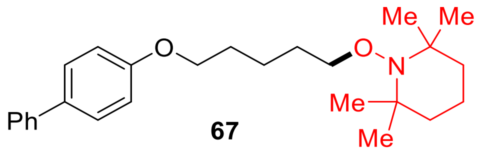

67

1-((5-([1,1'-Biphenyl]-4-yloxy)pentyl)oxy)-2,2,6,6-tetramethylpiperidine

Compound **67** was prepared following the general procedure, purification by column chromatography on silica gel (petroleum ether/EtOAc = 100:1). White solid. M.p.: 75–77 °C. ^{1}H NMR (400 MHz, Chloroform-*d*) δ 7.57 – 7.51 (m, 4H), 7.44 – 7.40 (m, 2H), 7.33 – 7.28 (m, 1H), 7.00 – 6.96 (m, 2H), 4.02 (t, *J* = 6.4 Hz, 2H), 3.78 (t, *J* = 6.0 Hz, 2H), 1.89 – 1.82 (m, 2H), 1.64 – 1.56 (m, 4H), 1.50 – 1.32 (m, 6H), 1.17 (s, 6H), 1.11 (s, 6H). ^{13}C NMR (100 MHz, Chloroform-*d*) δ 158.7, 140.9, 133.6, 128.7, 128.1, 126.7, 126.6, 114.8, 68.0, 59.7, 39.6, 33.1, 29.4, 28.6, 23.1, 20.2, 17.2. HR-MS (ESI) m/z calc. for $C_{26}H_{38}NO_2$ [M+H]$^{+}$: 396.2897, found: 396.2894.

Cyclic Voltammetry

The cyclic voltammetry was carried out with a Shanghai Chenhua CHI630E workstation. A glassy-carbon (GC) electrode (3mm-diameter, disk-electrode) and a Pt plate were used as the working electrode (or counter electrode), a Pb plate was used as the working electrode and an Ag/Ag^+ electrode was used as the reference electrode in acetonitrile, respectively. The measurements were carried out at a scan rate of 100 mV s^{-1}, if not indicated otherwise. The operation temperature was 298 K.

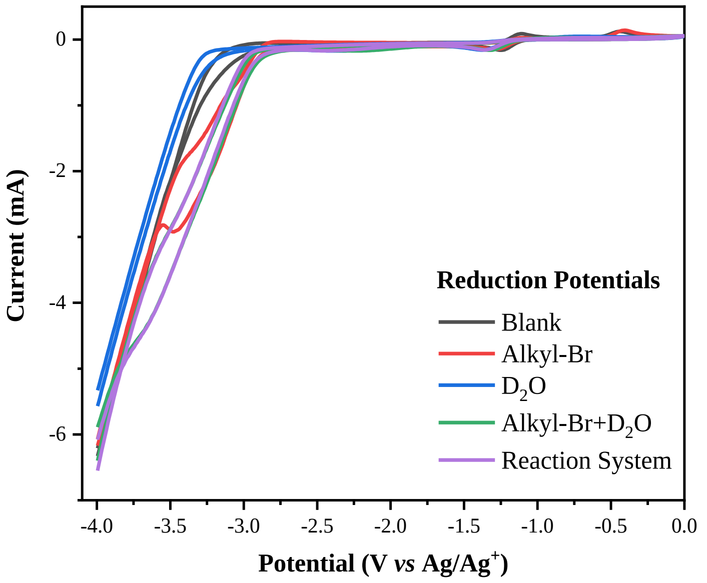

Supplementary Figure 8. Cyclic voltammograms of Alkyl-Br, D_2O and reaction system at 100 mVs^{-1} in MeCN. ^{n}Bu$_4$NBF$_4$ (0.1 M in MeCN), blank (black), Alkyl-Br (5 mM, red), D_2O (250 mM, blue), Alkyl-Br + D_2O (green), reaction system (purple).

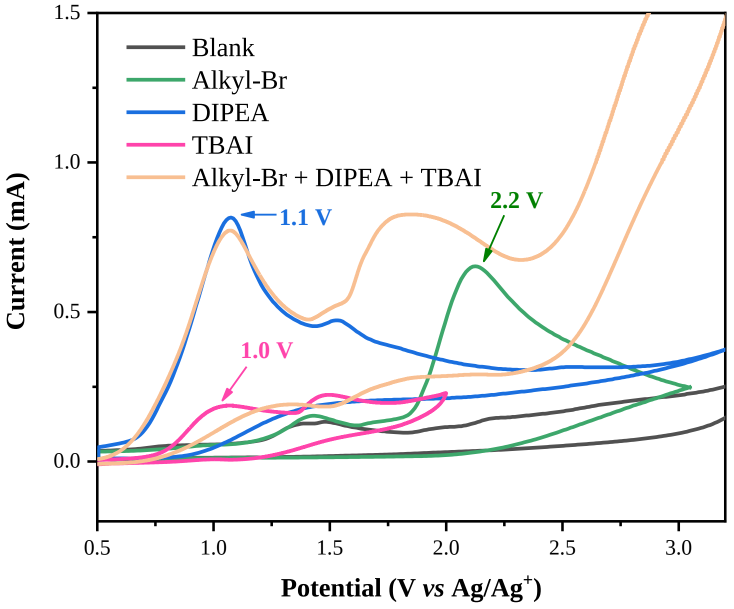

Supplementary Figure 9. Cyclic voltammograms of Alkyl-Br, DIPEA and reaction system at 100 mVs^{-1} in MeCN. ${}^{n}Bu_4NBF_4$ (0.1 M in MeCN), blank (black), Alkyl-Br (5 mM, green), TBAI (1 Mm, pink), DIPEA (15 mM, blue), reaction system (yellow).

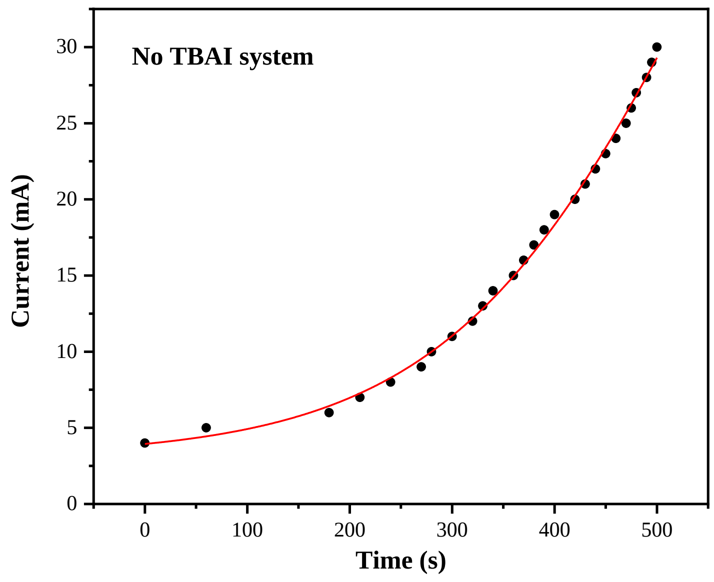

Supplementary Figure 10. Current-time curve of the system without TBAI.

Reaction conditions: undivided cell, carbon felt anode, lead plate cathode constant current = 30 mA, alkyl halides (0.5 mmol), D_2O (50.0 equiv), TBAI (20 mol%),

DMF (5.0 mL), rt.

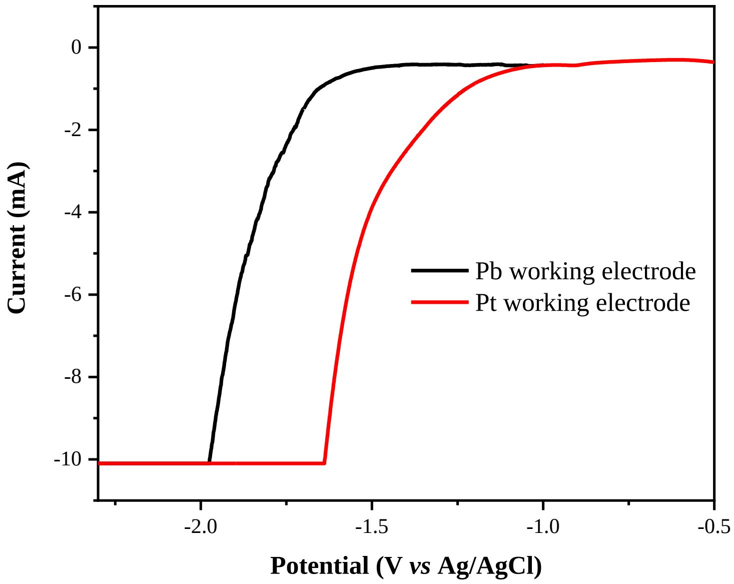

Supplementary Figure 11. HER performance of Pb working electrode and Pt working electrode in 1M NaOH solution.

GC-MS Date

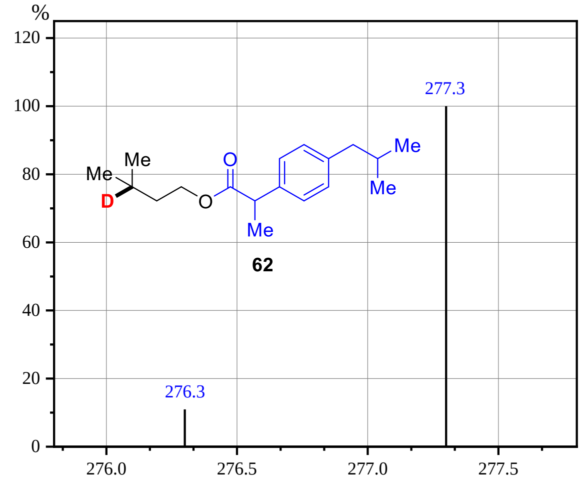

Supplementary Figure 12. GC-MS date of compound **63**

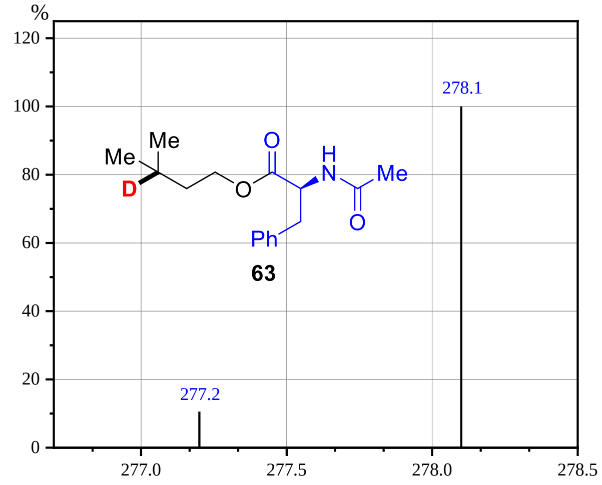

Supplementary Figure 13. GC-MS date of compound **63**

$$\%\ deuteration = 100 - \left(\frac{residual\ interal}{number\ of\ labelling\ sites} \times 100\right)$$

NMR Spectrum

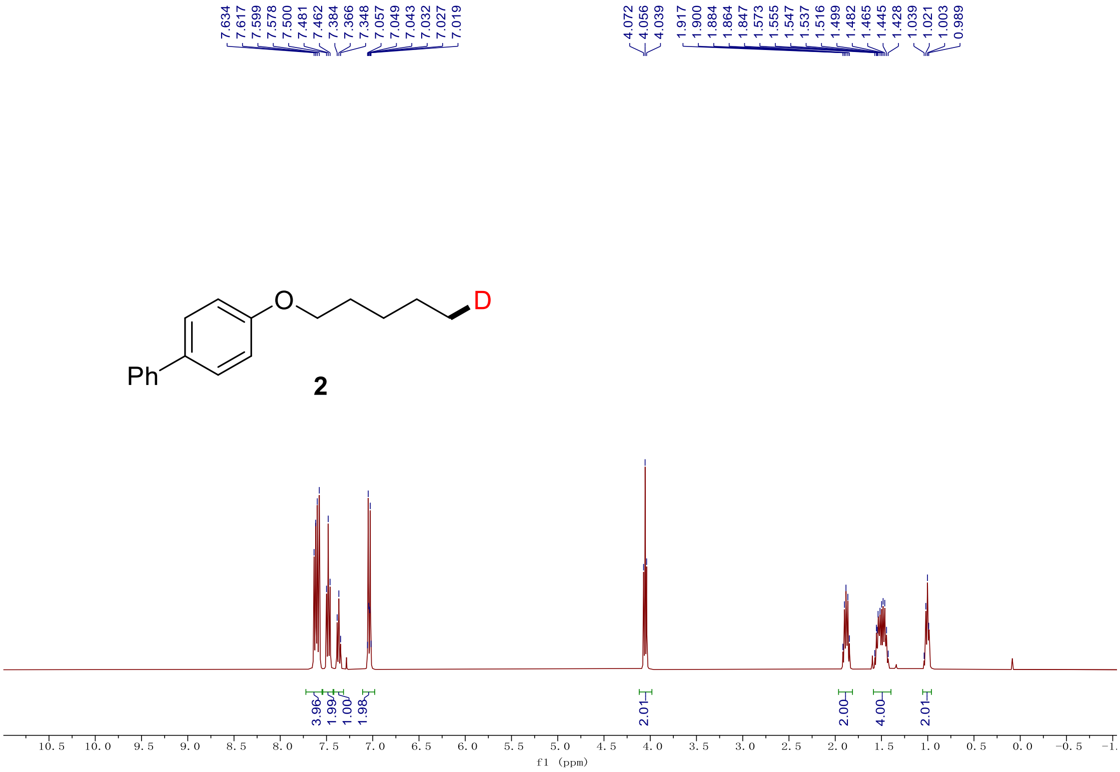

Supplementary Figure 14. ^{1}H NMR of compound **2** (400 MHz, Chloroform-*d*)

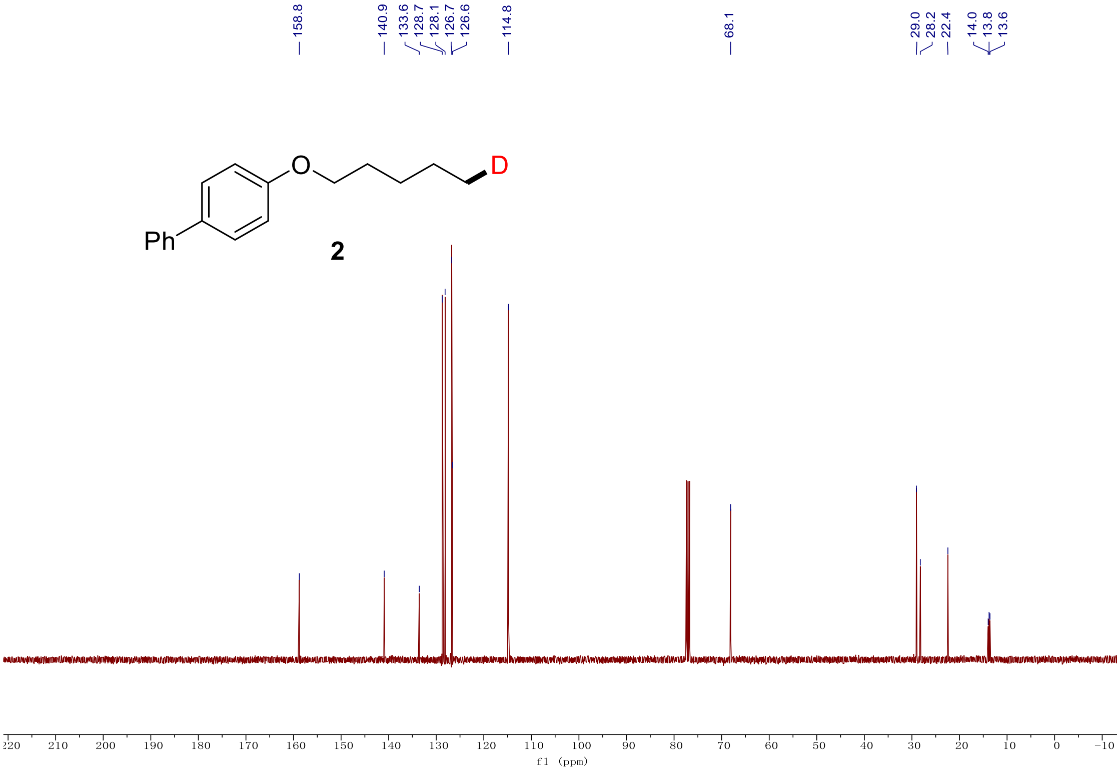

Supplementary Figure 15. ^{13}C NMR of compound **2** (100 MHz, Chloroform-*d*)

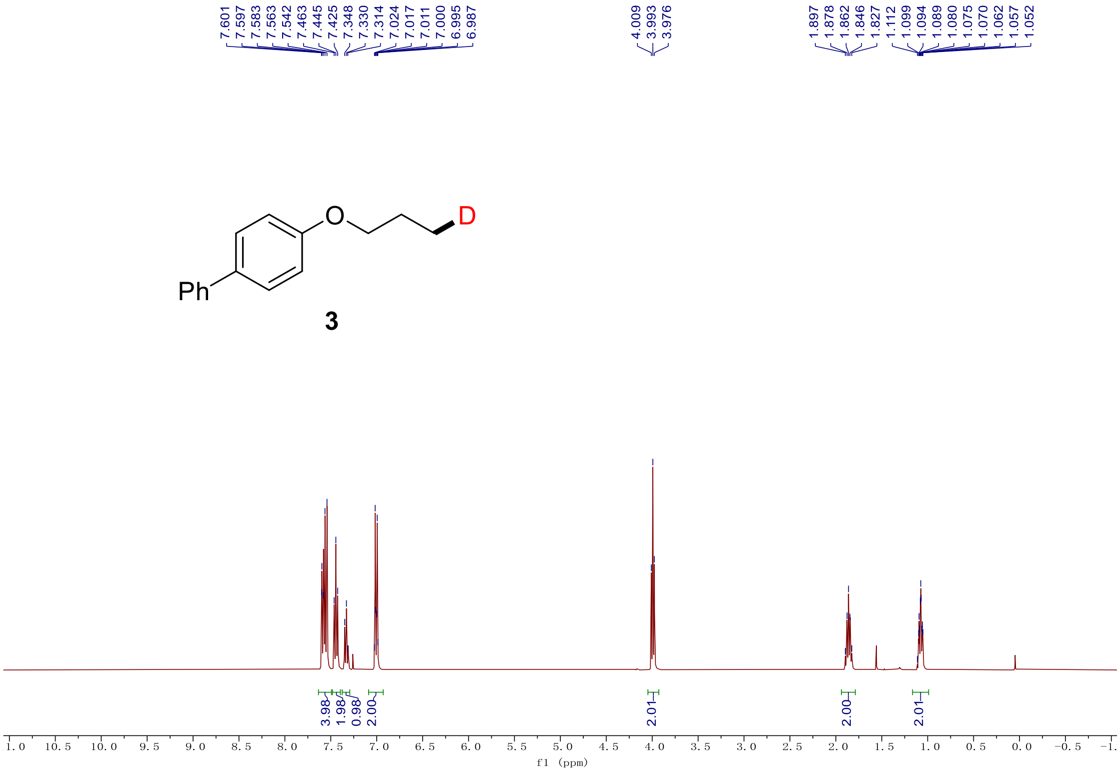

Supplementary Figure 16. ^{1}H NMR of compound **3** (400 MHz, Chloroform-*d*)

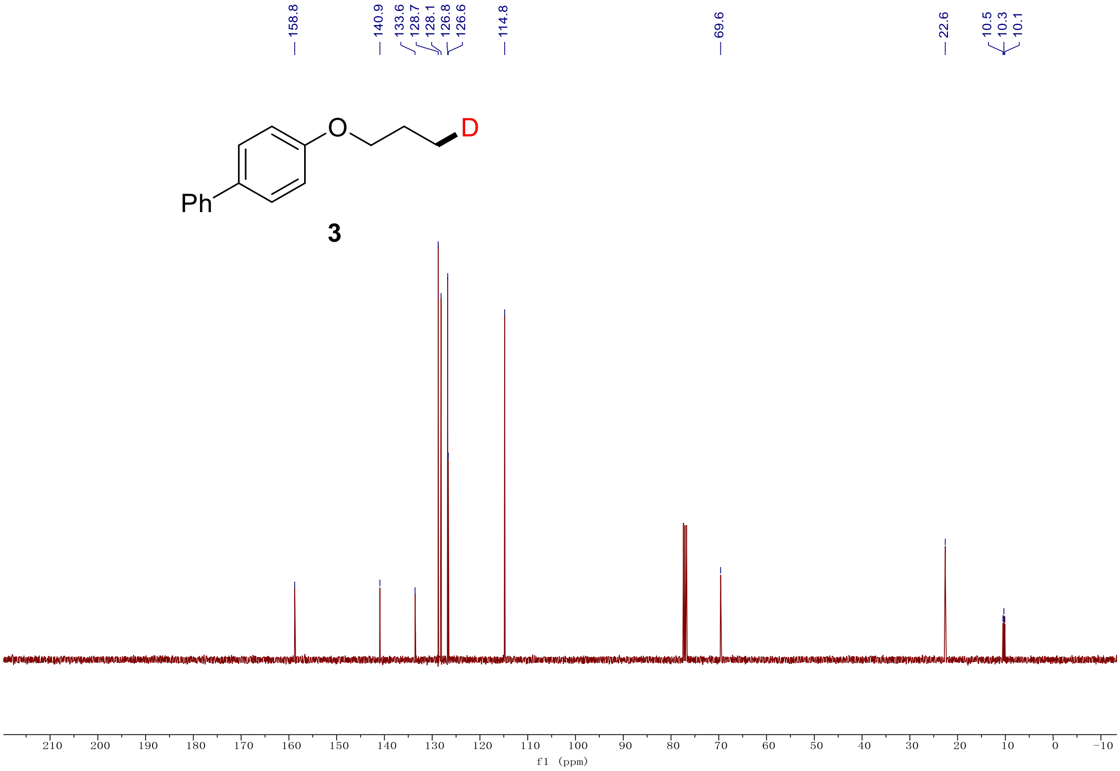

Supplementary Figure 17. ^{13}C NMR of compound **3** (100 MHz, Chloroform-*d*)

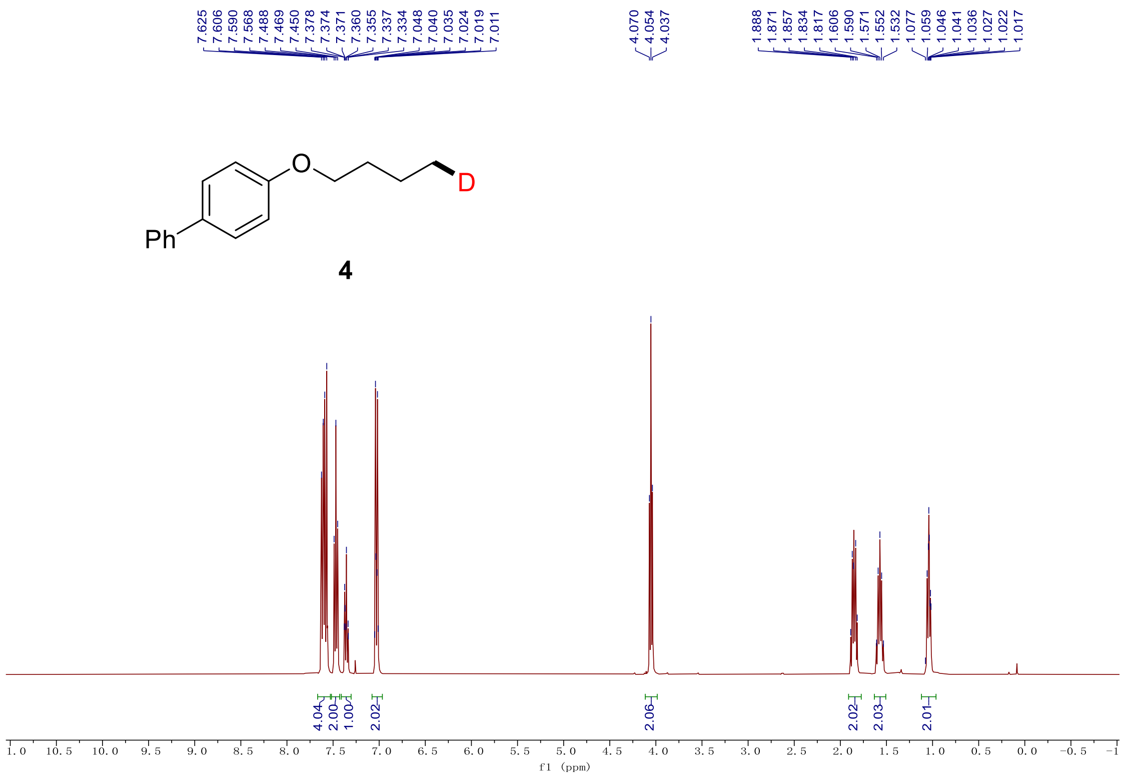

Supplementary Figure 18. ^{1}H NMR of compound **4** (400 MHz, Chloroform-*d*)

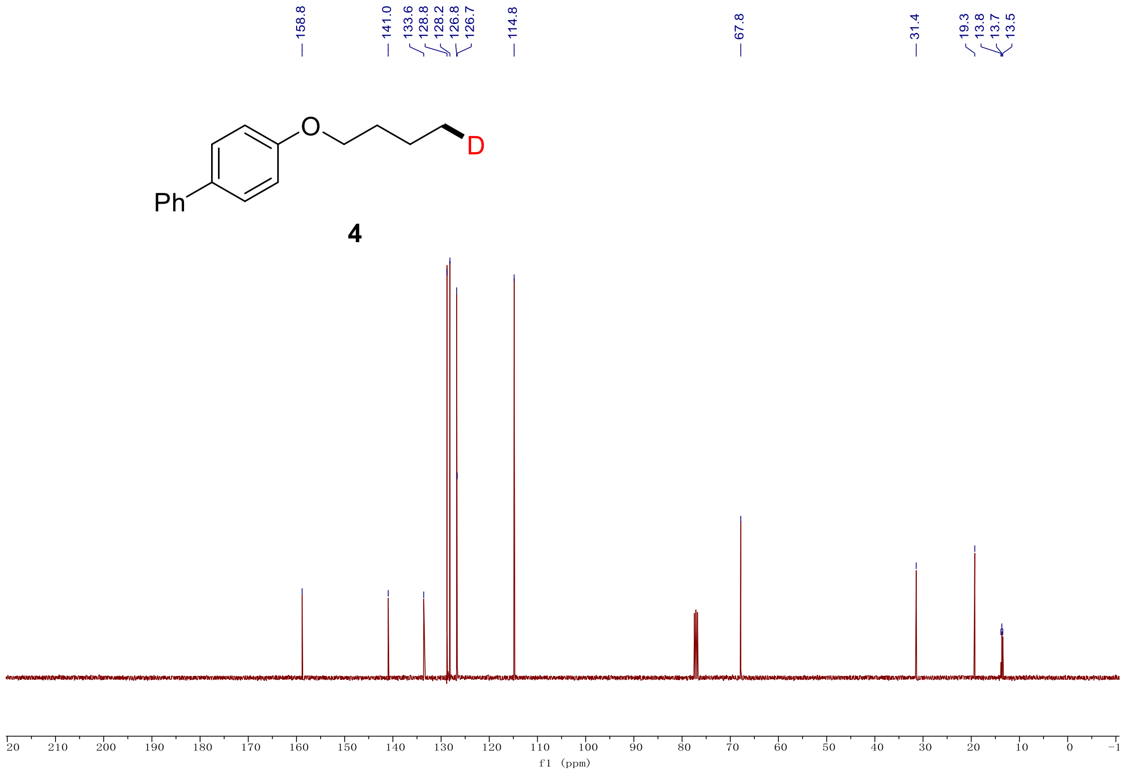

Supplementary Figure 19. ^{13}C NMR of compound **4** (100 MHz, Chloroform-*d*)

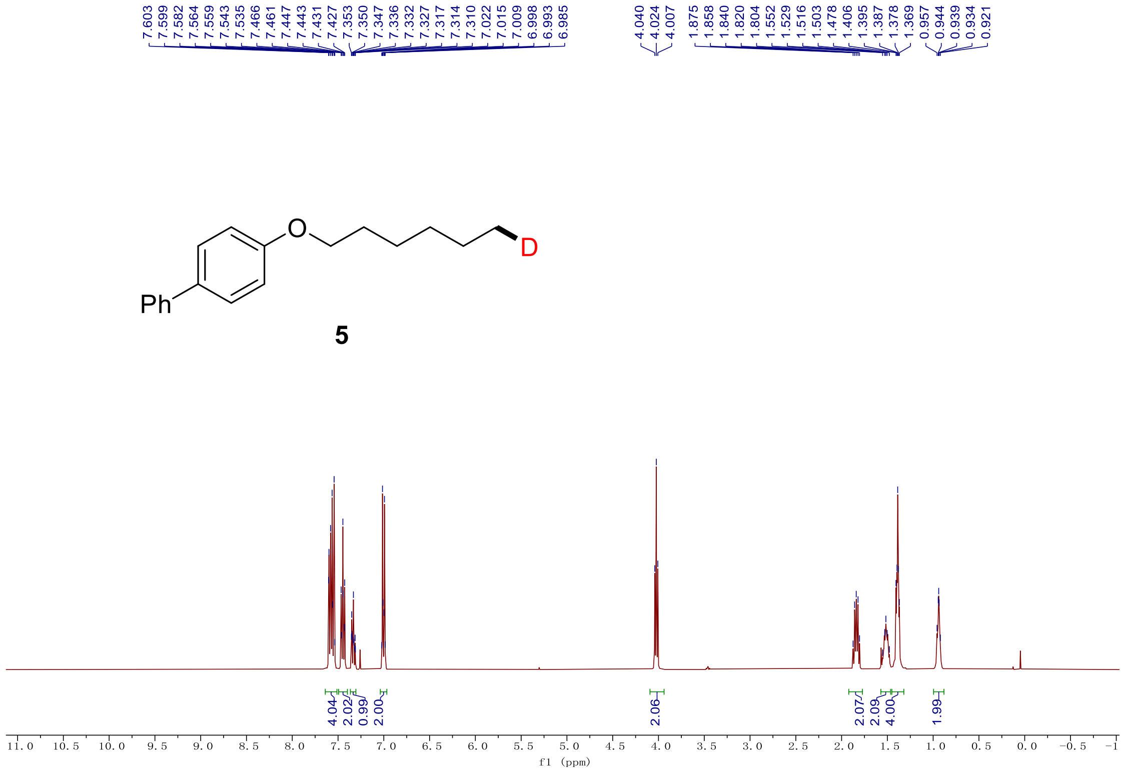

Supplementary Figure 20. ^{1}H NMR of compound **5** (400 MHz, Chloroform-*d*)

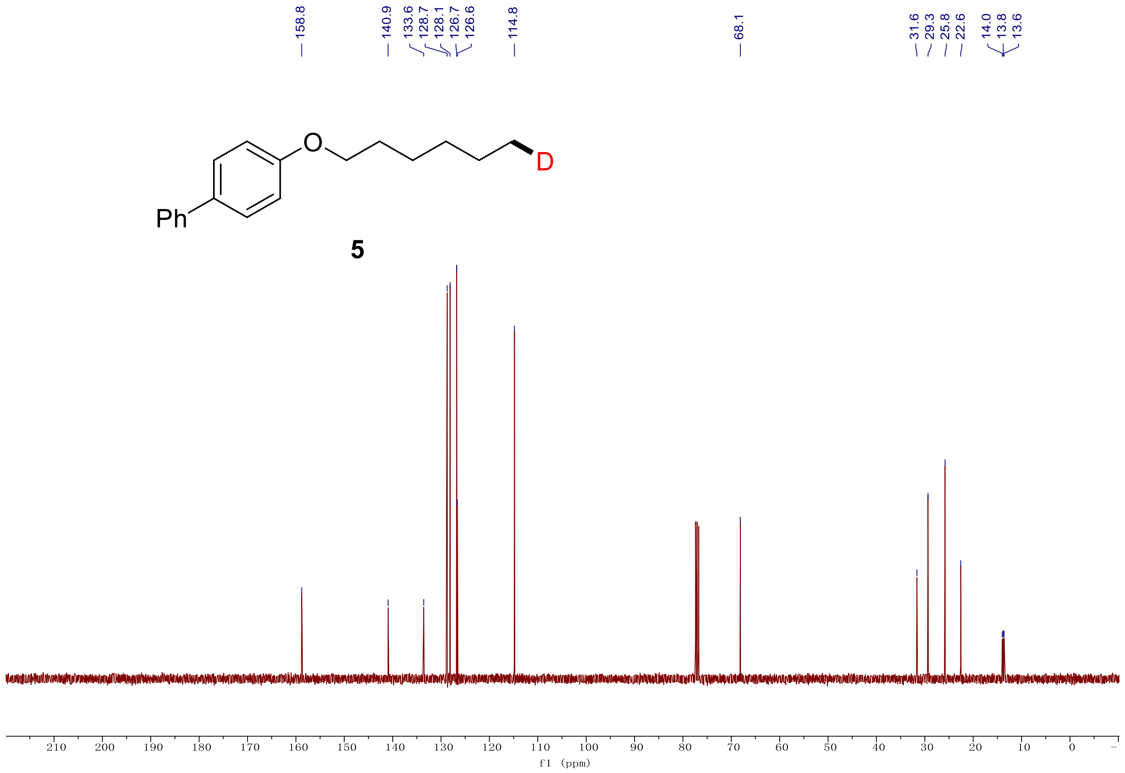

Supplementary Figure 21. ^{13}C NMR of compound **5** (100 MHz, Chloroform-*d*)

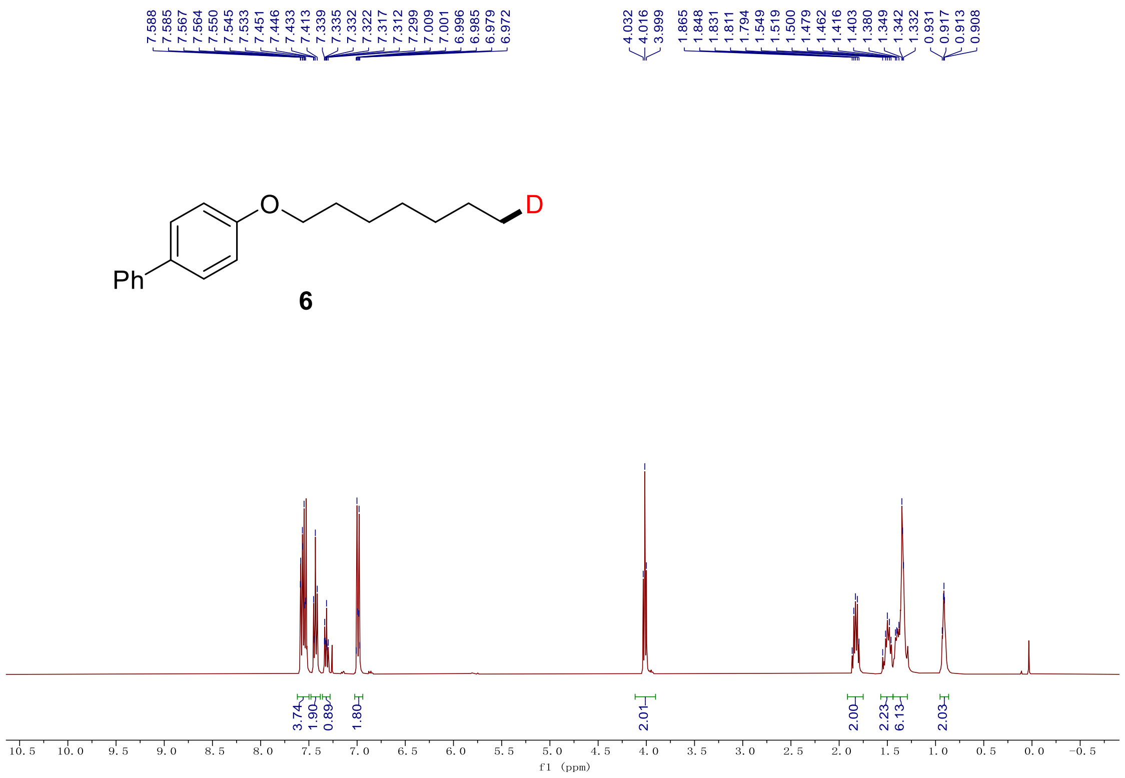

Supplementary Figure 22. ^{1}H NMR of compound **6** (400 MHz, Chloroform-*d*)

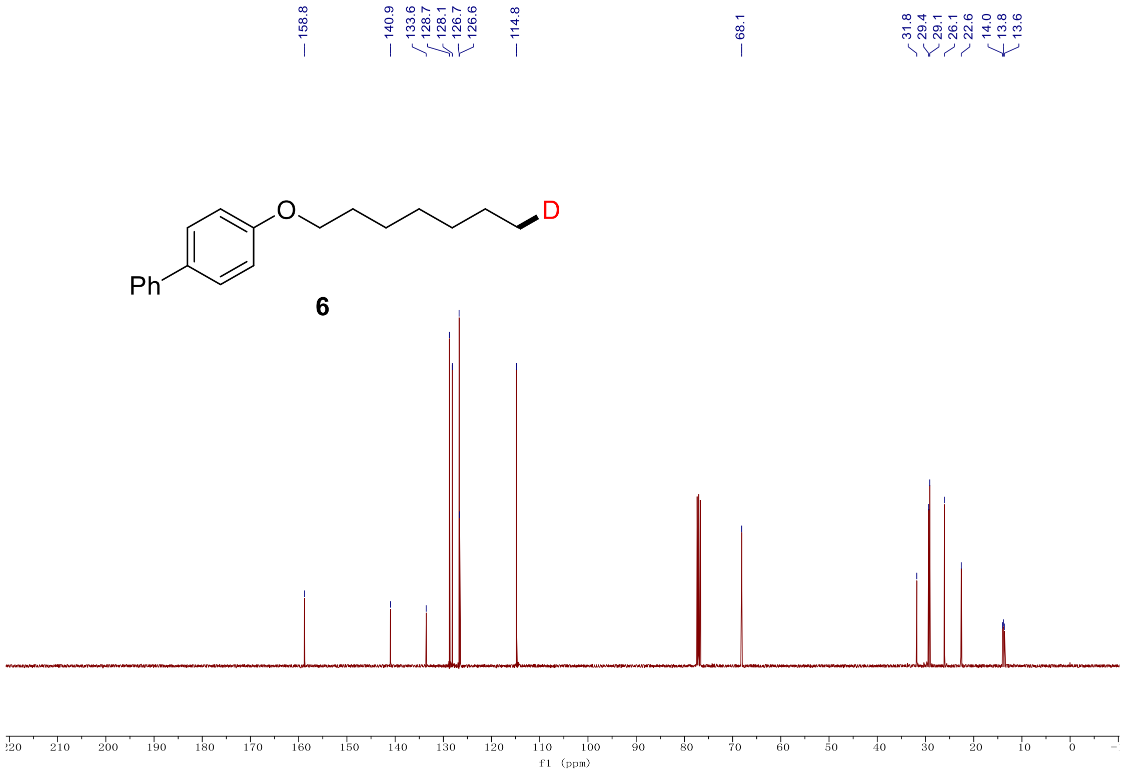

Supplementary Figure 23. ^{13}C NMR of compound **6** (100 MHz, Chloroform-*d*)

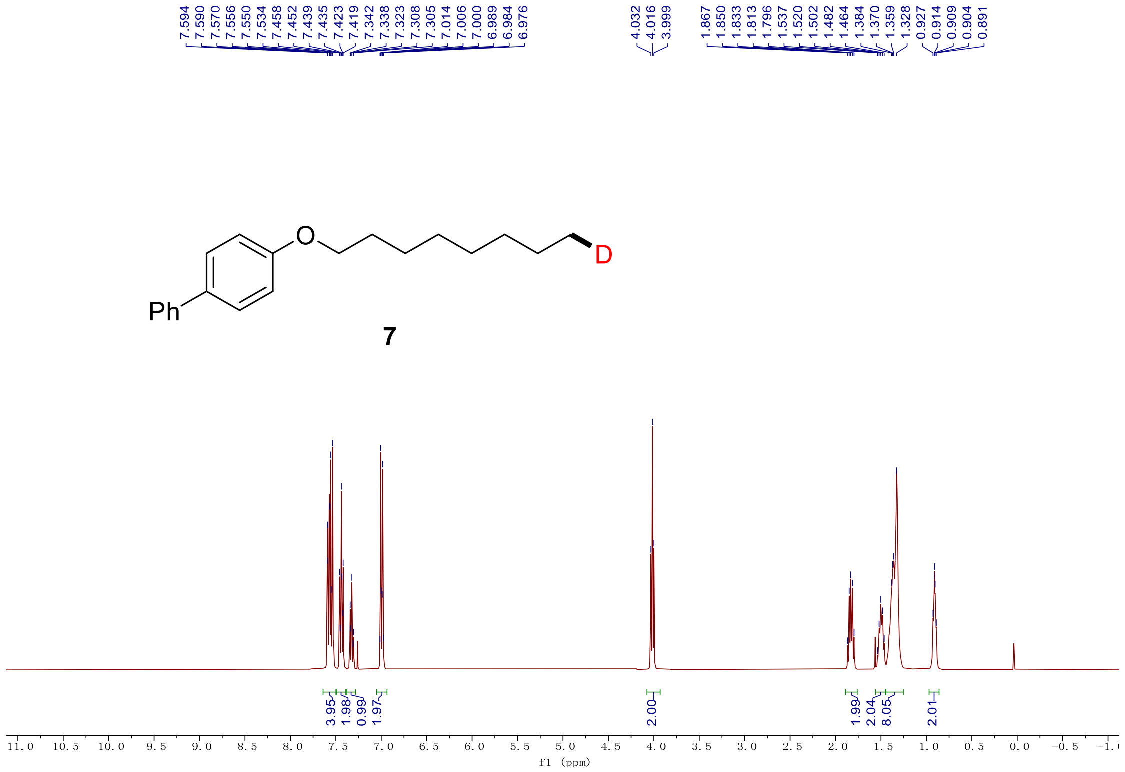

Supplementary Figure 24. ^{1}H NMR of compound **7** (400 MHz, Chloroform-*d*)

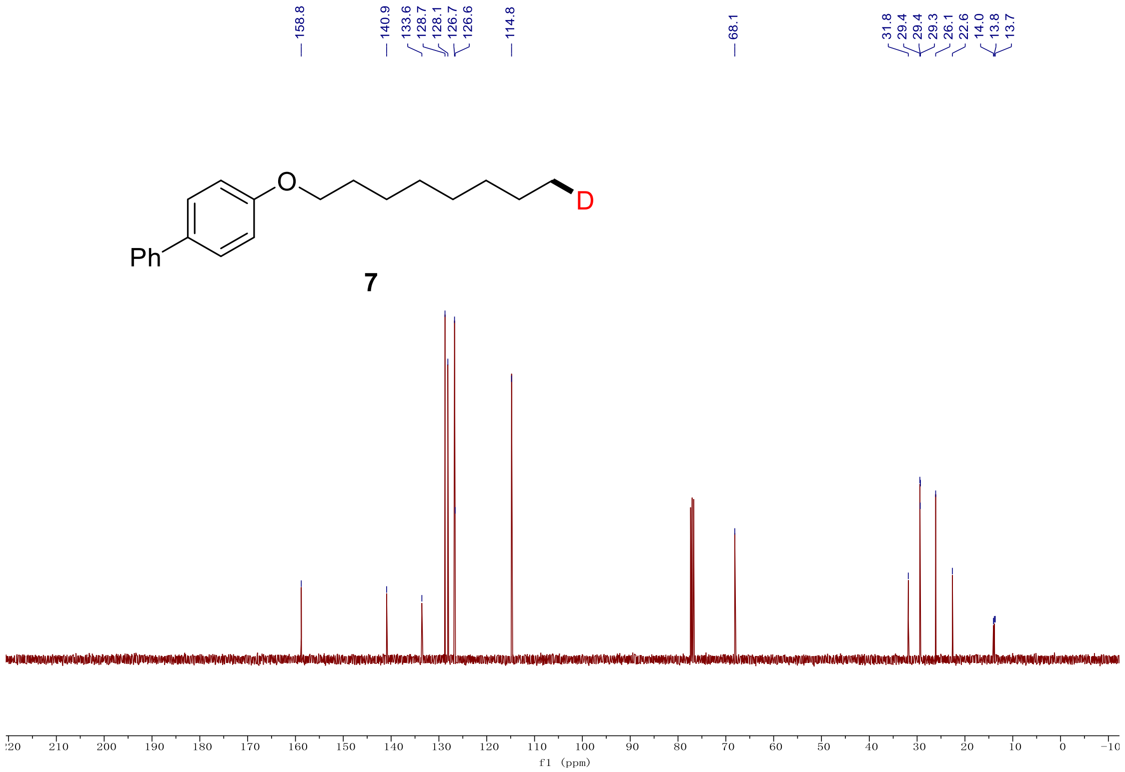

Supplementary Figure 25. ^{13}C NMR of compound **7** (100 MHz, Chloroform-*d*)

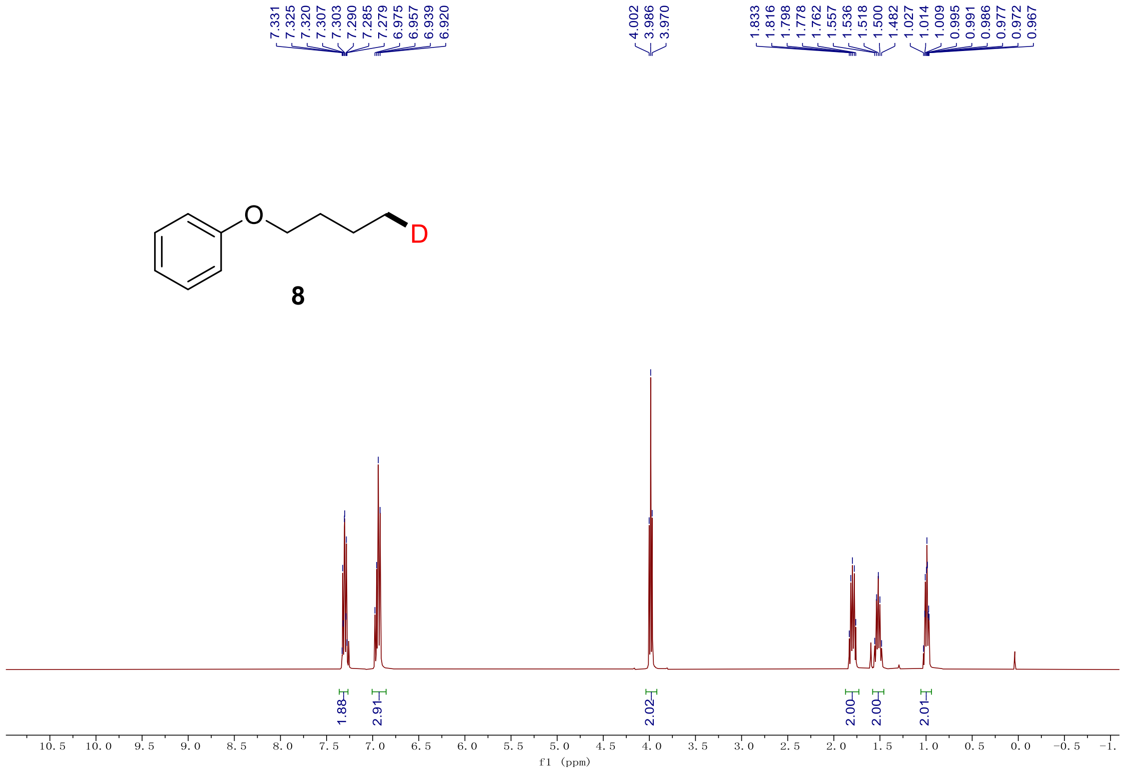

Supplementary Figure 26. ^{1}H NMR of compound **8** (400 MHz, Chloroform-*d*)

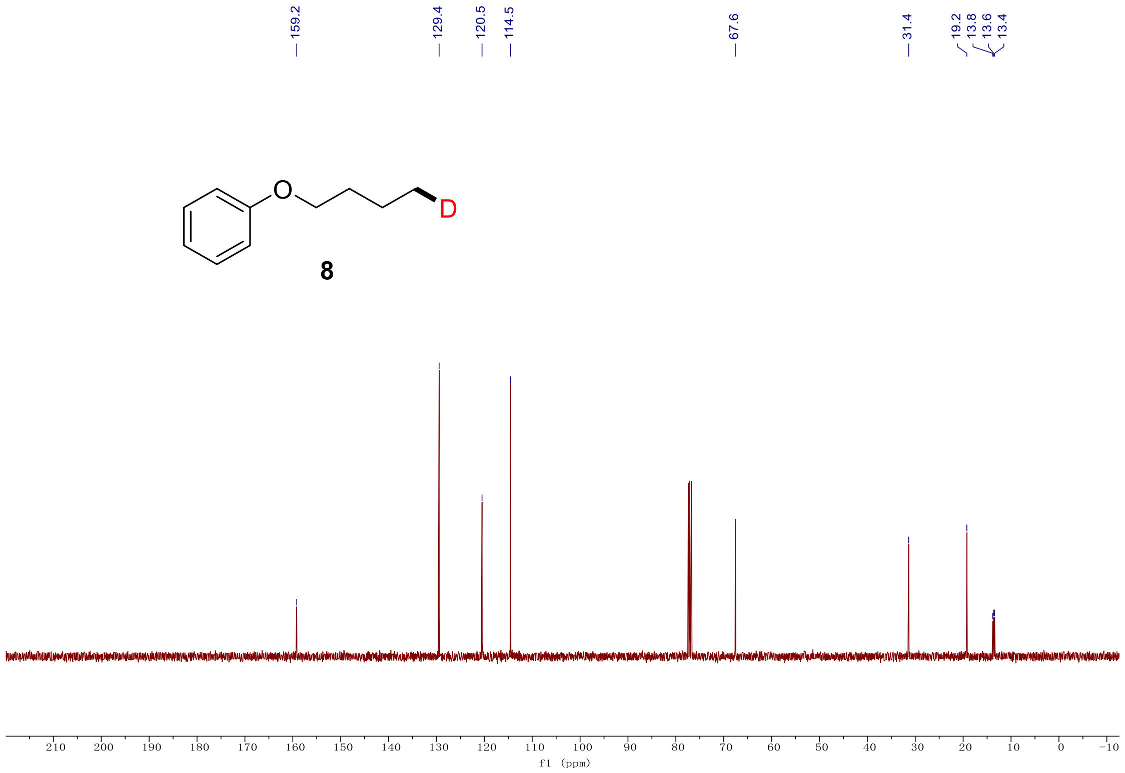

Supplementary Figure 27. ^{13}C NMR of compound **8** (100 MHz, Chloroform-*d*)

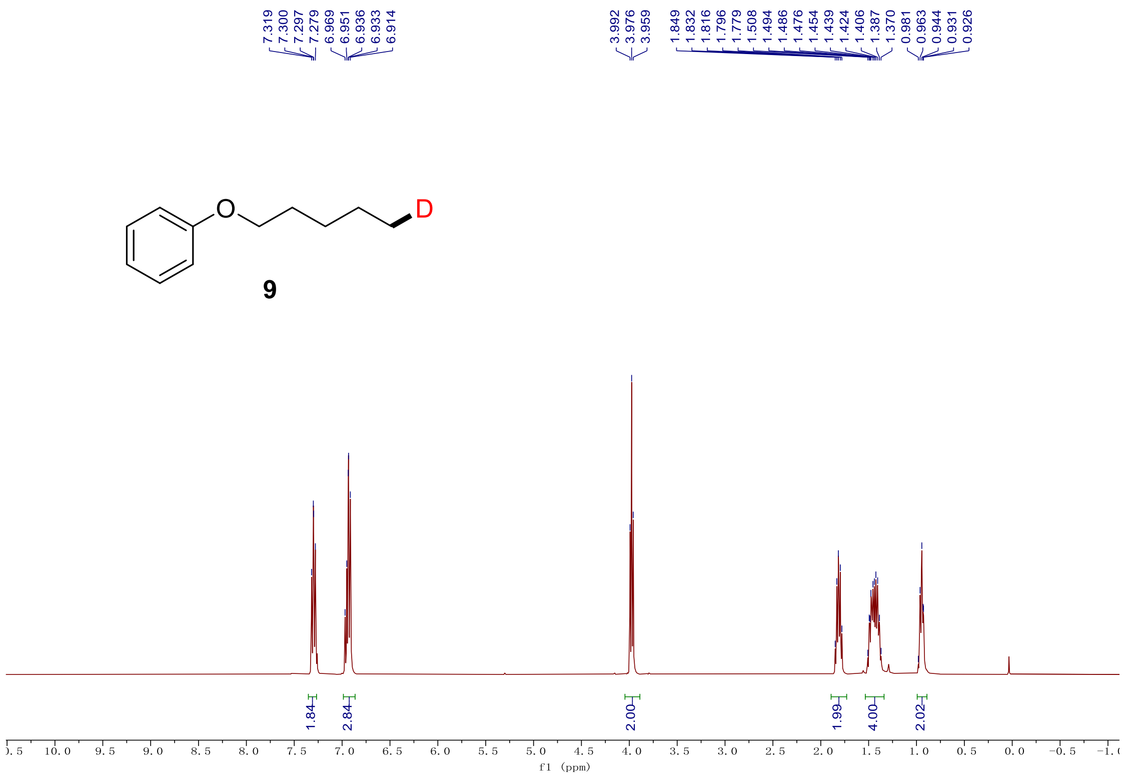

Supplementary Figure 28. ^{1}H NMR of compound **9** (400 MHz, Chloroform-*d*)

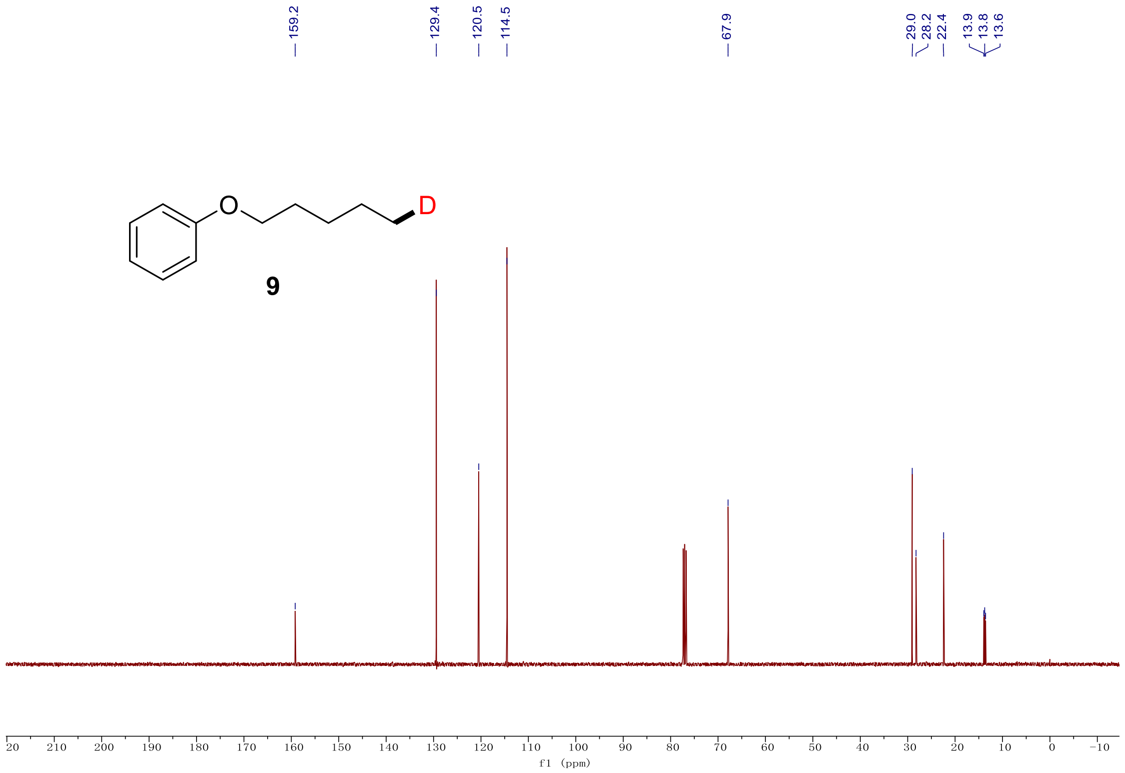

Supplementary Figure 29. ^{13}C NMR of compound **9** (100 MHz, Chloroform-*d*)

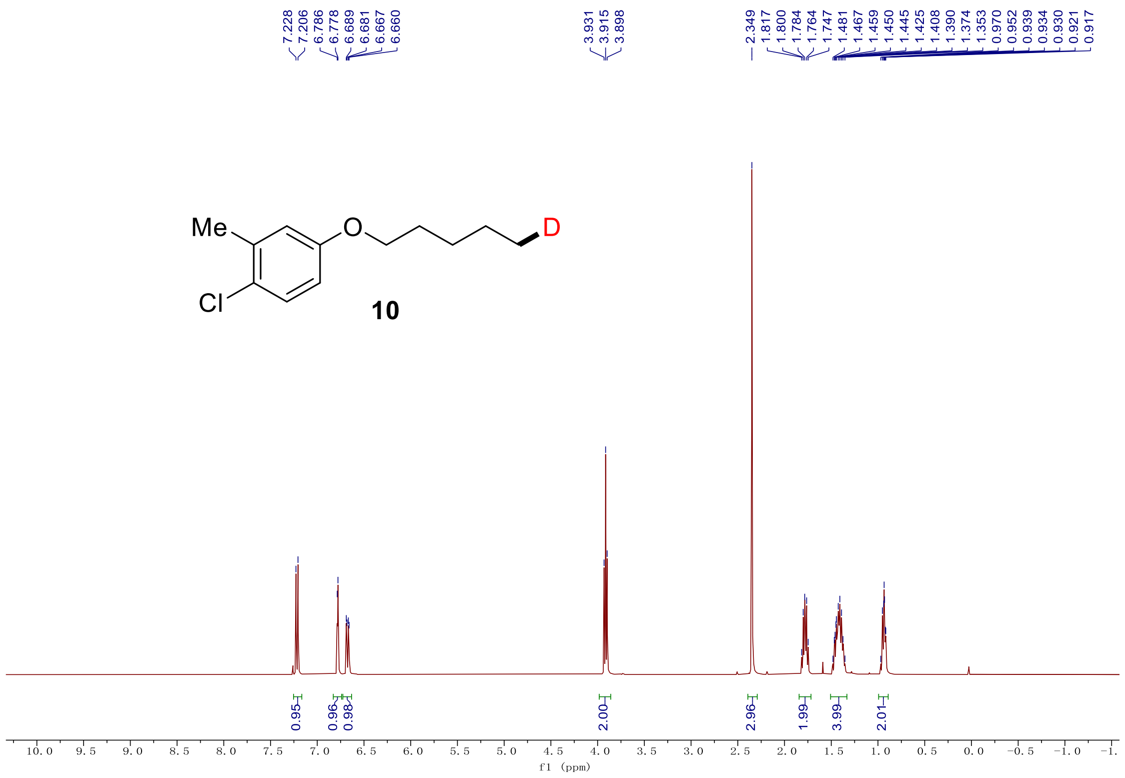

Supplementary Figure 30. ^{1}H NMR of compound **10** (400 MHz, Chloroform-*d*)

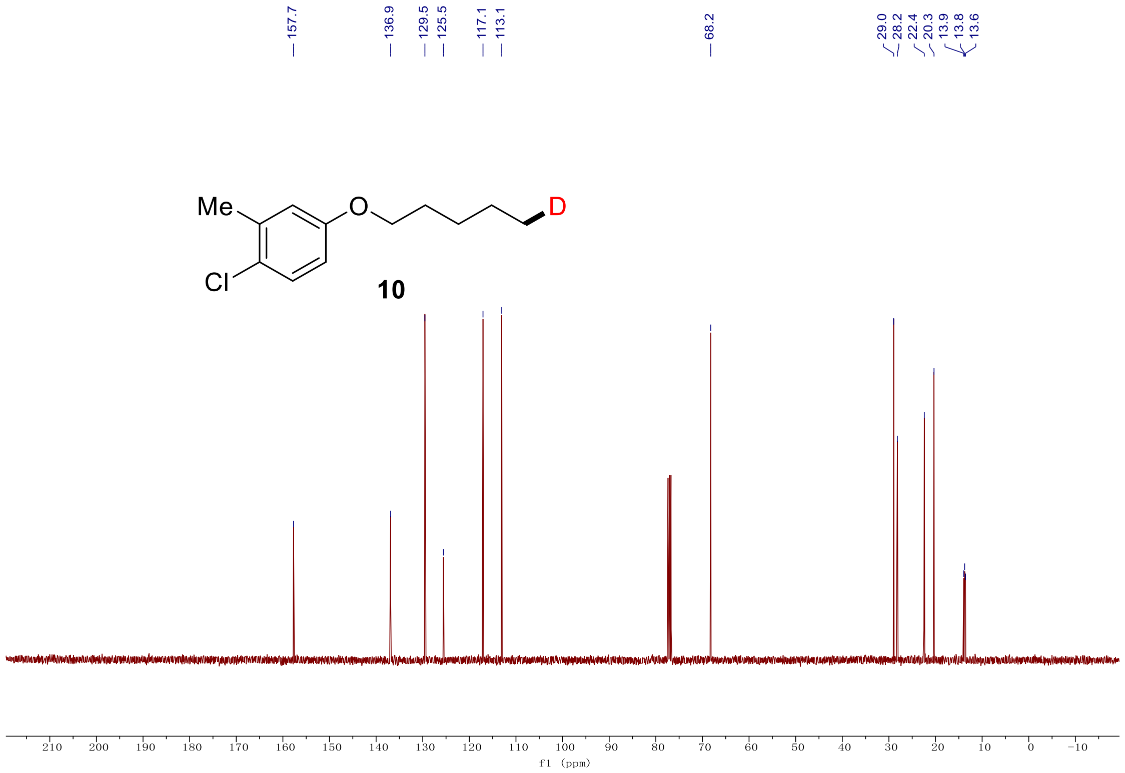

Supplementary Figure 31. ^{13}C NMR of compound **10** (100 MHz, Chloroform-*d*)

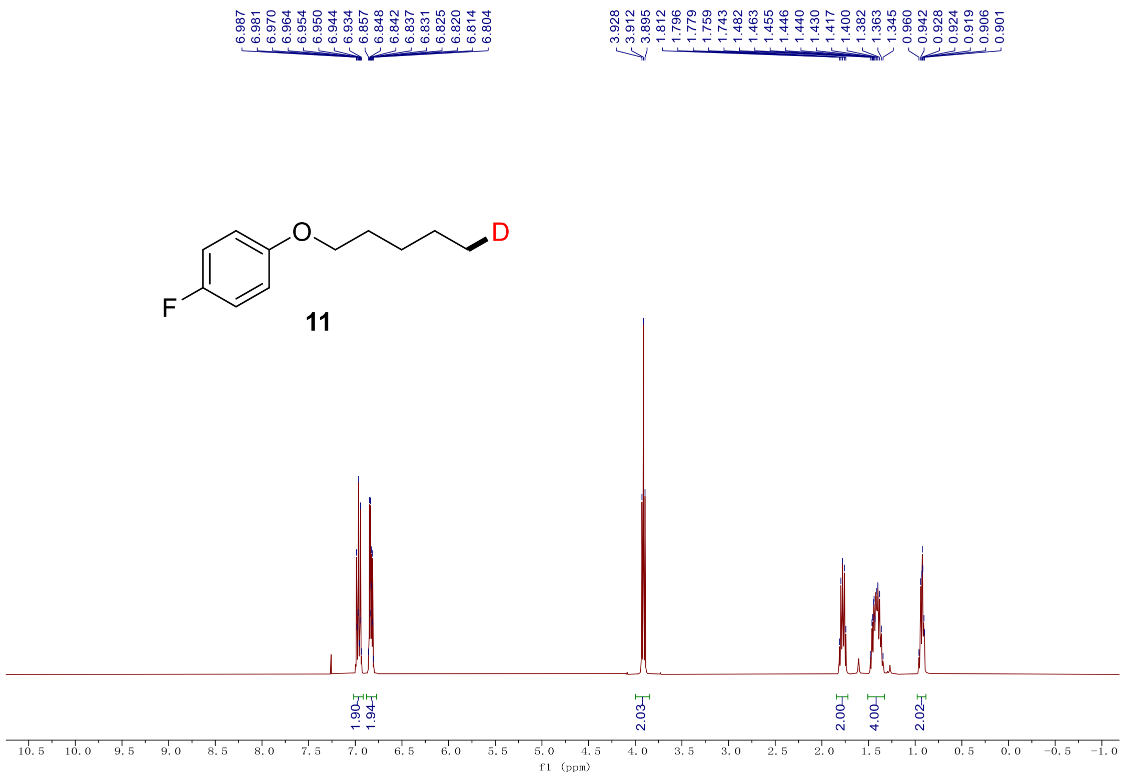

Supplementary Figure 32. ^{1}H NMR of compound **11** (400 MHz, Chloroform-*d*)

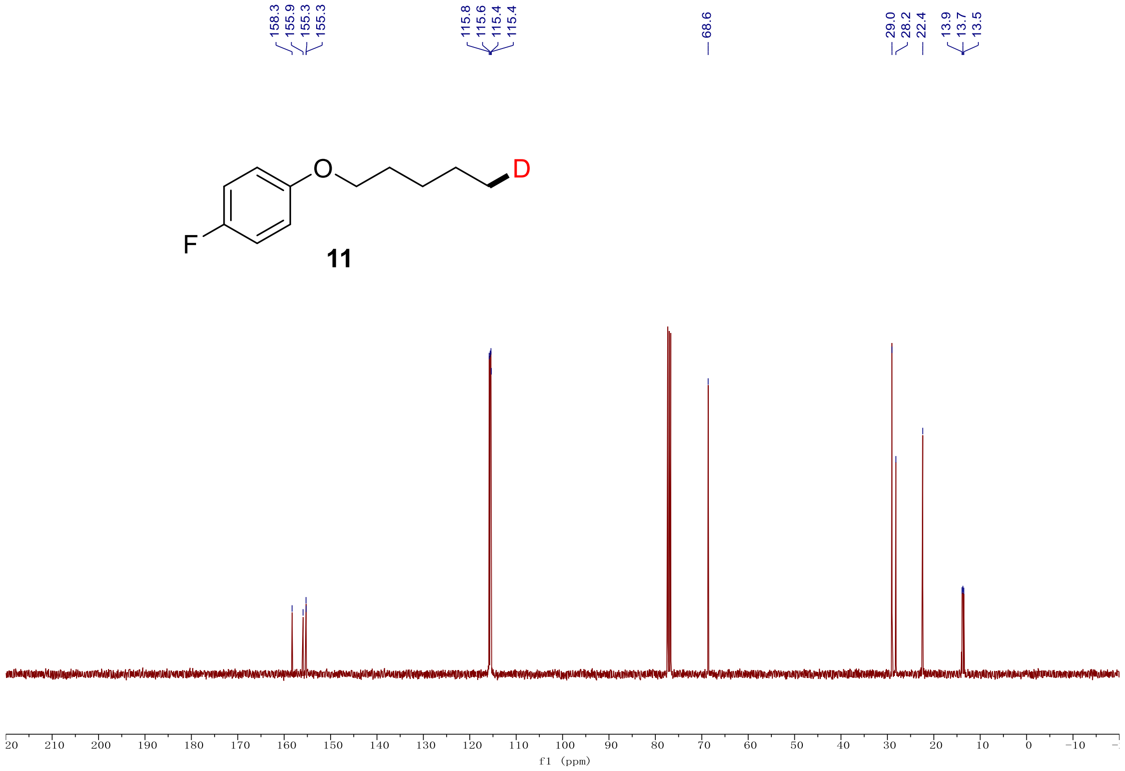

Supplementary Figure 33. ^{13}C NMR of compound **11** (100 MHz, Chloroform-*d*)

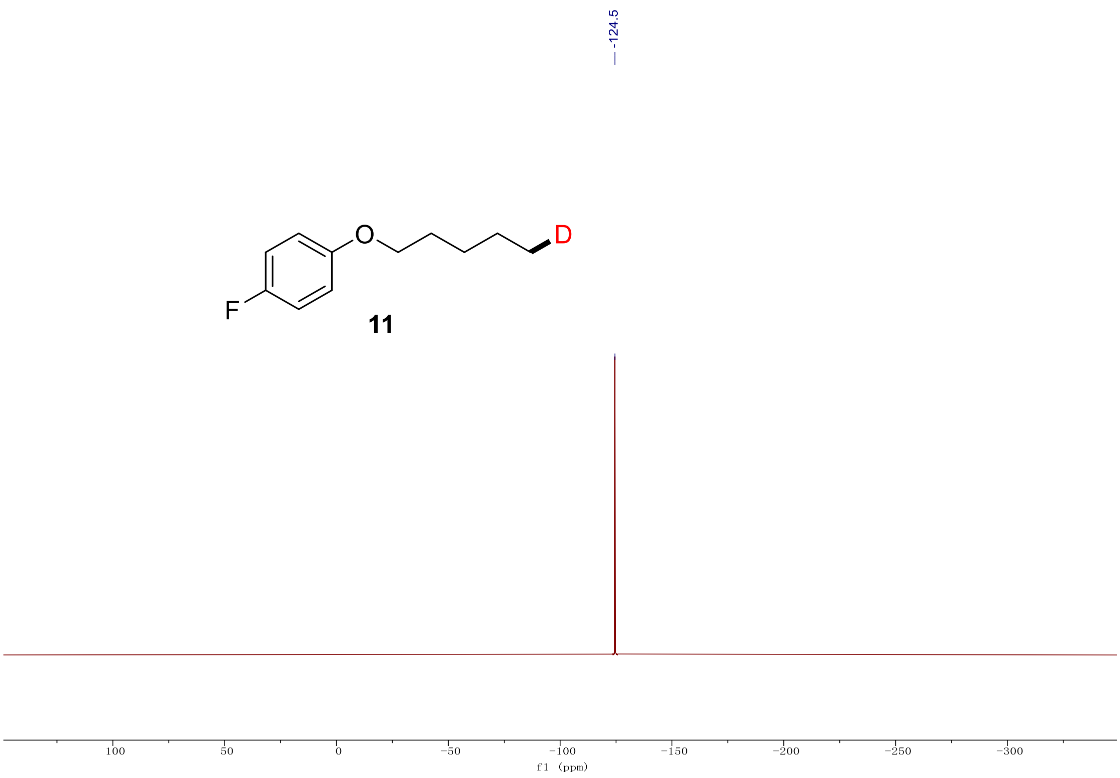

Supplementary Figure 34. ^{19}F NMR of compound **11** (375 MHz, Chloroform-*d*)

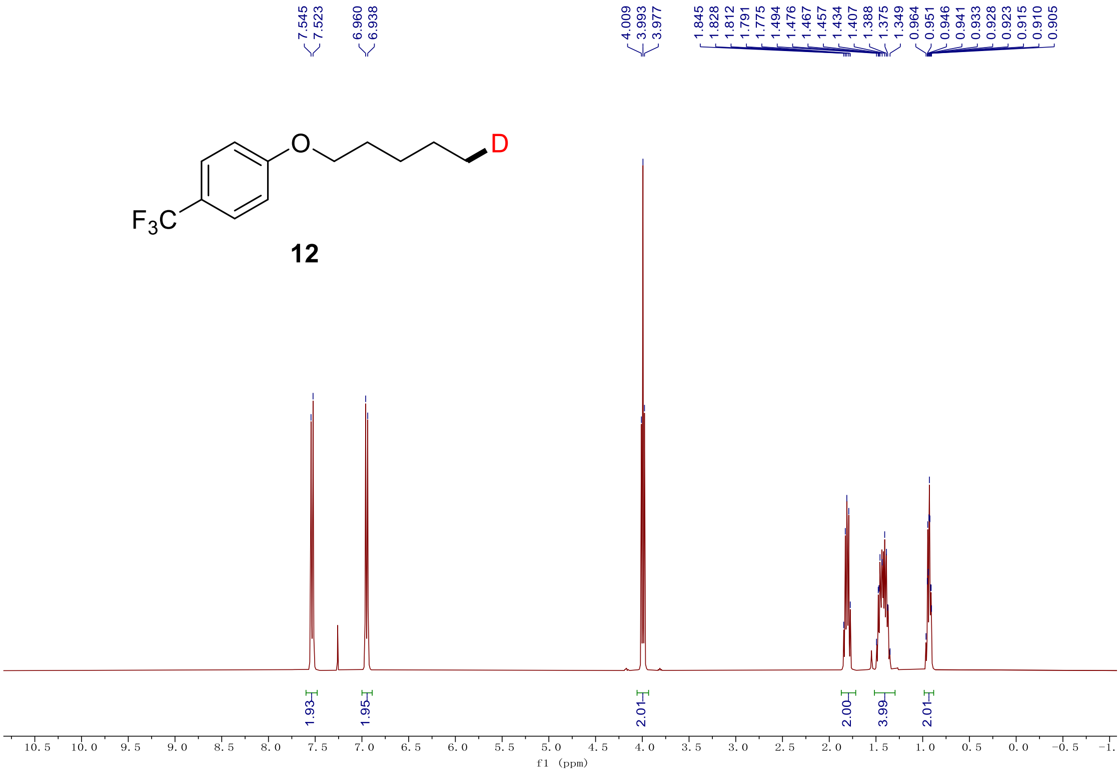

Supplementary Figure 35. ^{1}H NMR of compound **12** (400 MHz, Chloroform-*d*)

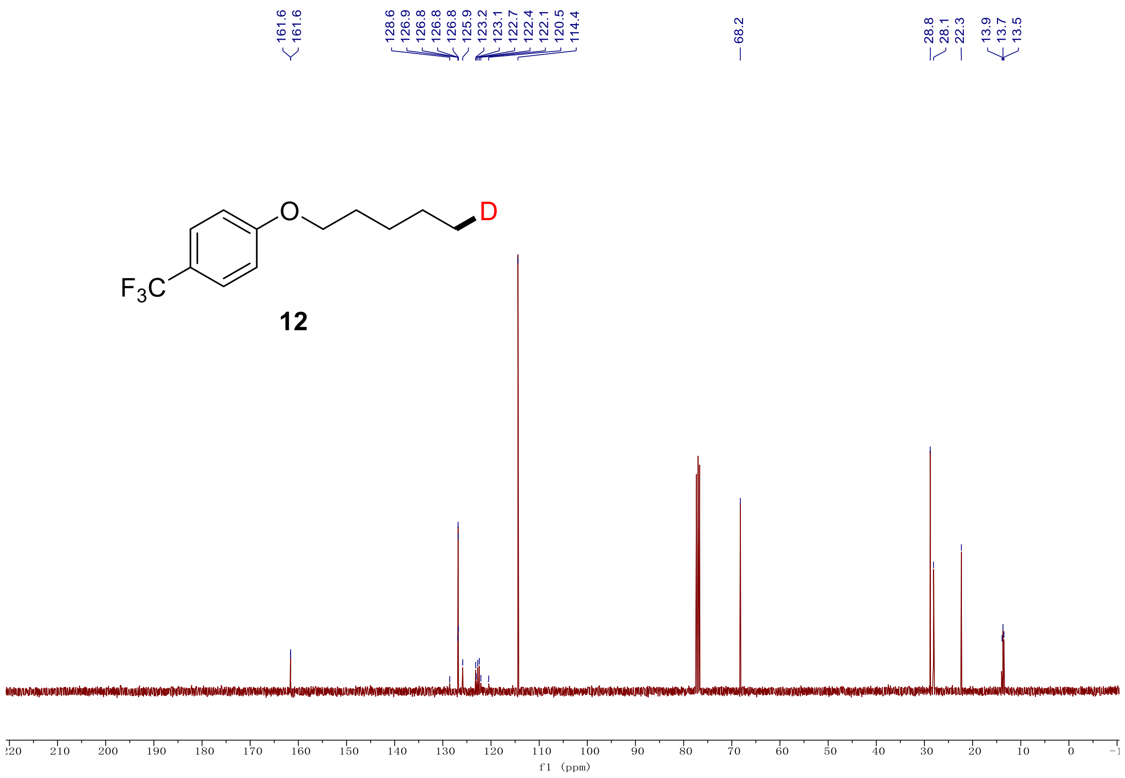

Supplementary Figure 36. ^{13}C NMR of compound 1**2** (100 MHz, Chloroform-*d*)

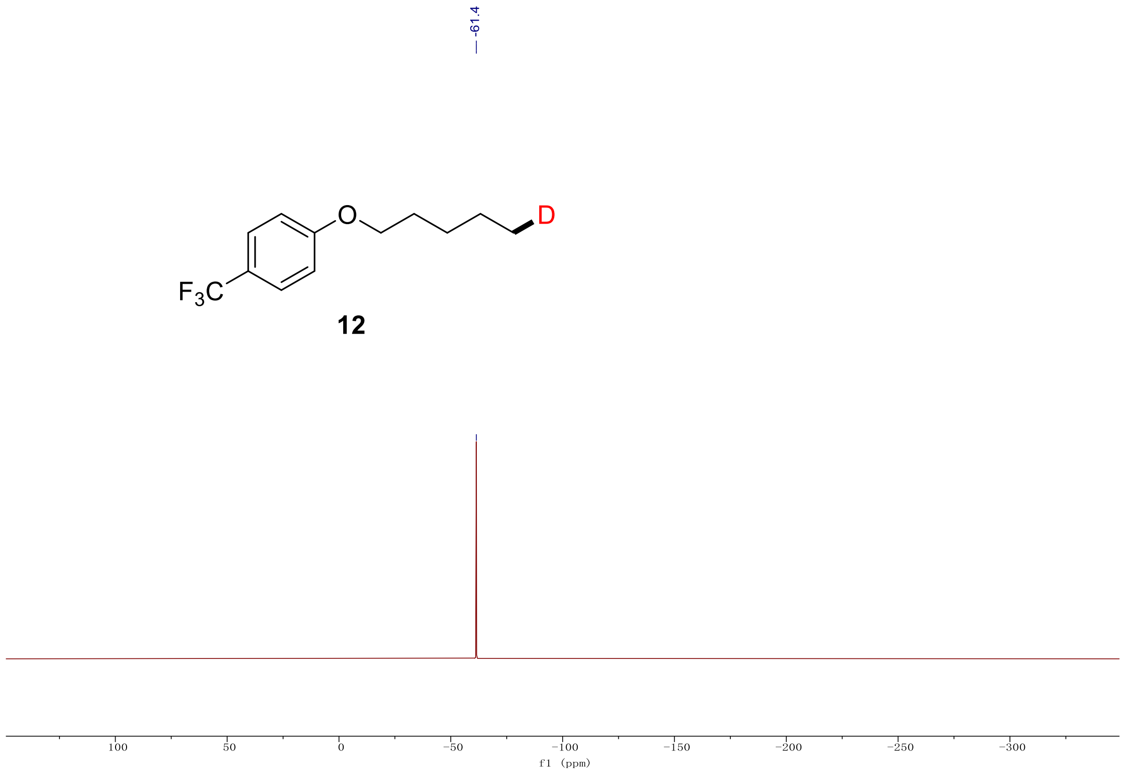

Supplementary Figure 37. ^{19}F NMR of compound 1**2** (375 MHz, Chloroform-*d*)

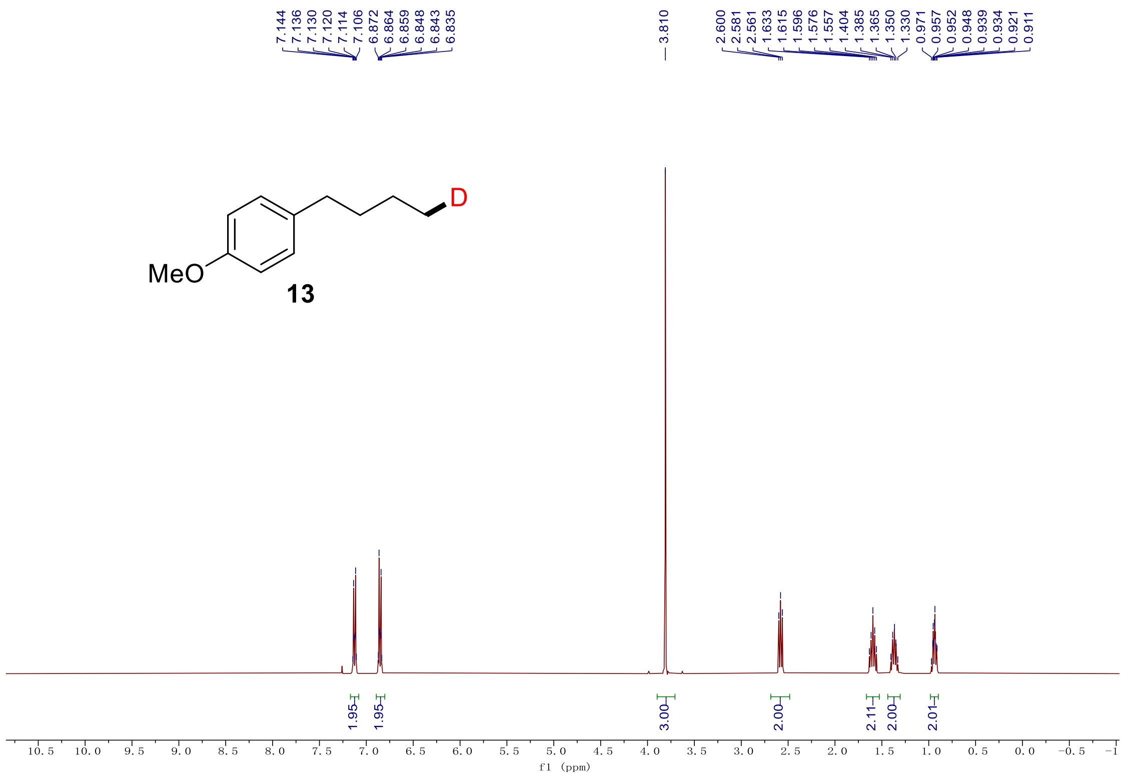

Supplementary Figure 38. ^{1}H NMR of compound **13** (400 MHz, Chloroform-*d*)

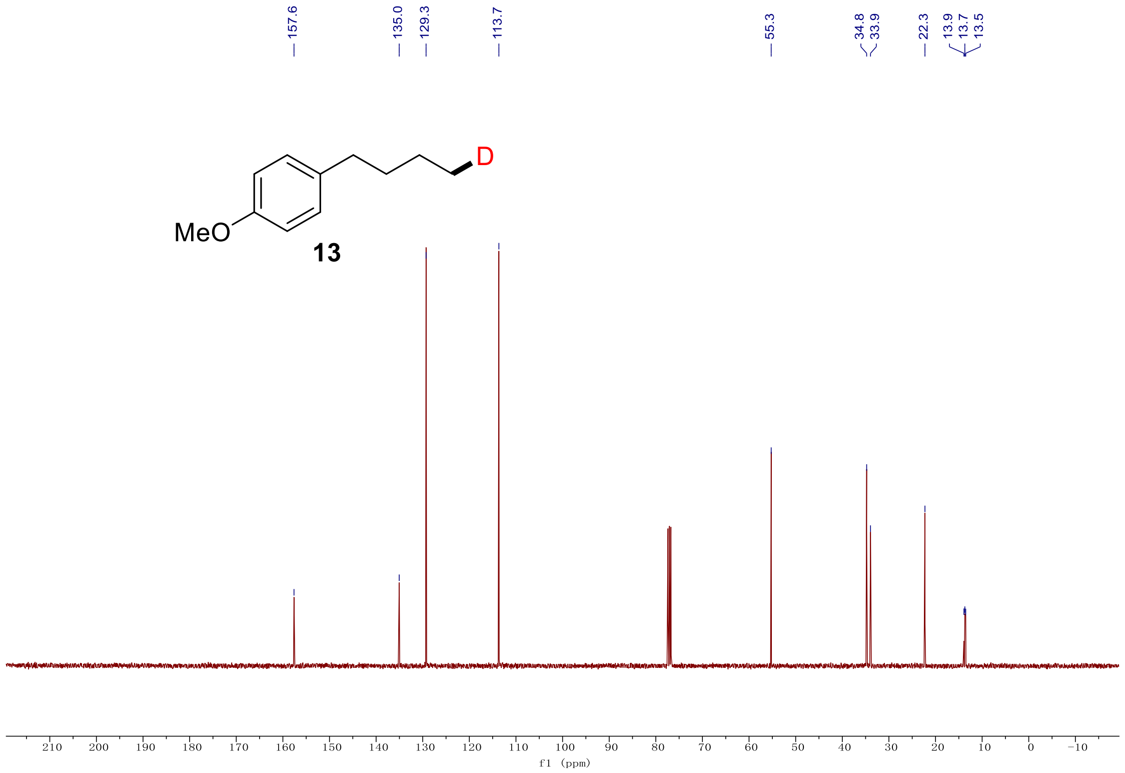

Supplementary Figure 39. ^{13}C NMR of compound **13** (100 MHz, Chloroform-*d*)

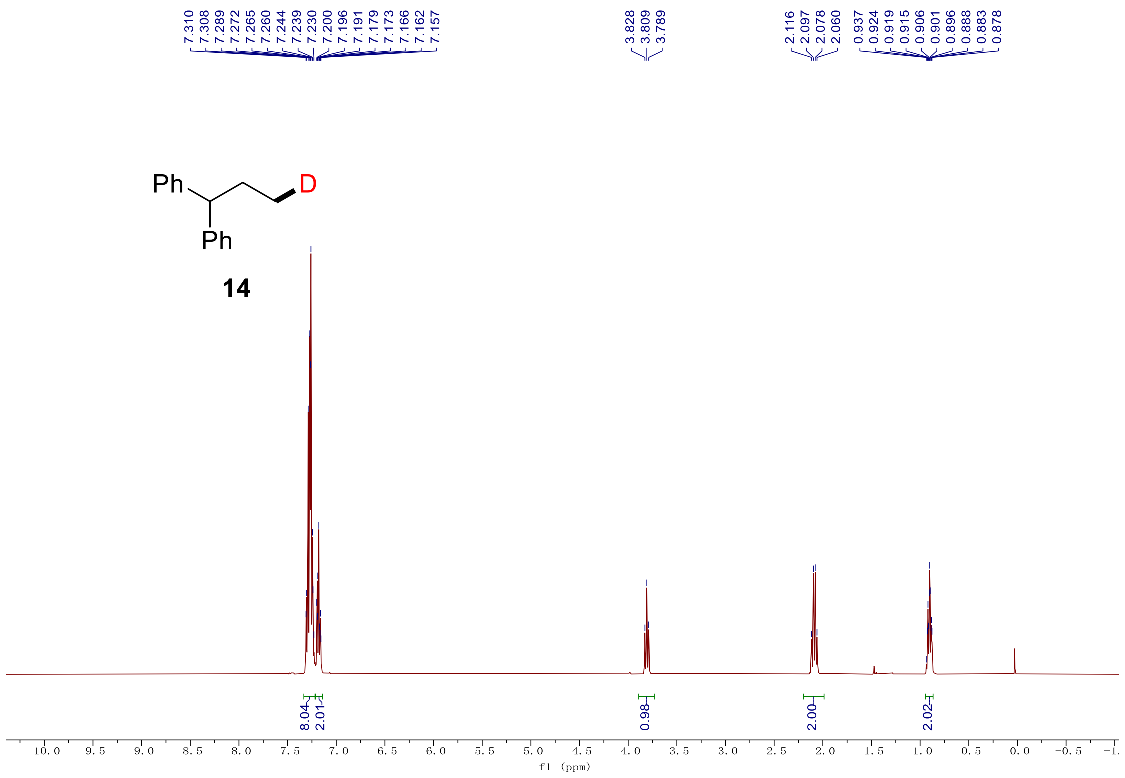

Supplementary Figure 40. ^{1}H NMR of compound **14** (400 MHz, Chloroform-*d*)

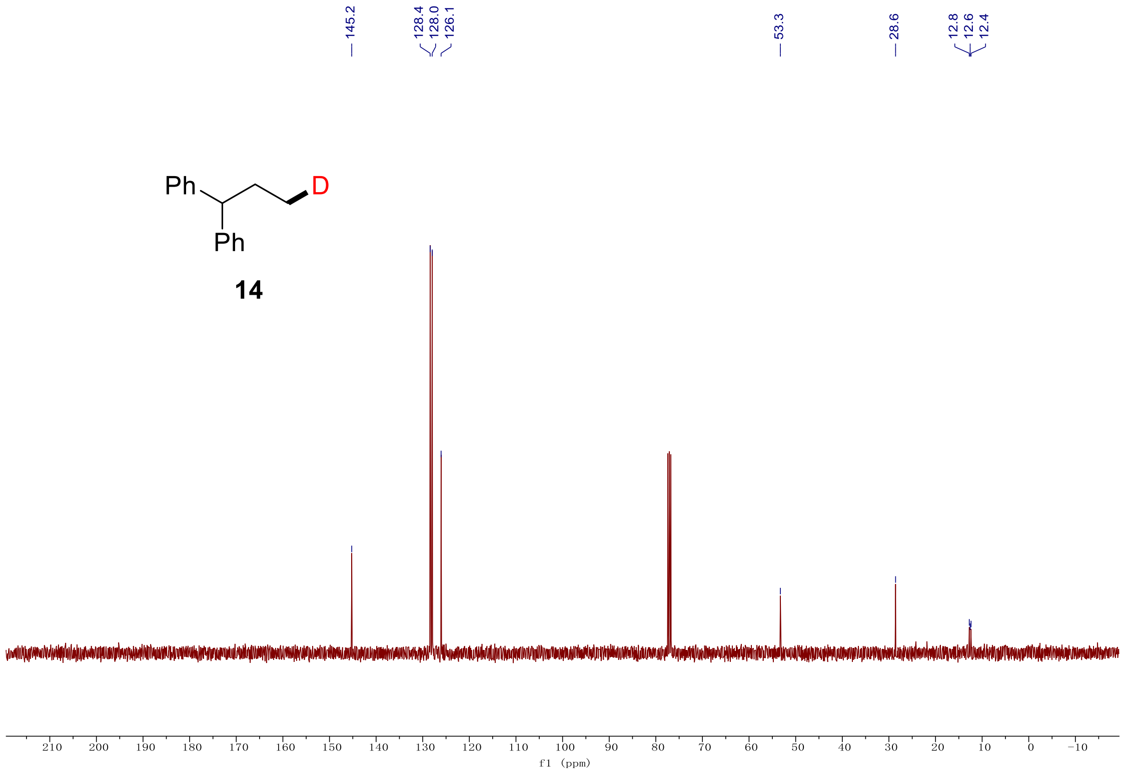

Supplementary Figure 41. ^{13}C NMR of compound **14** (100 MHz, Chloroform-*d*)

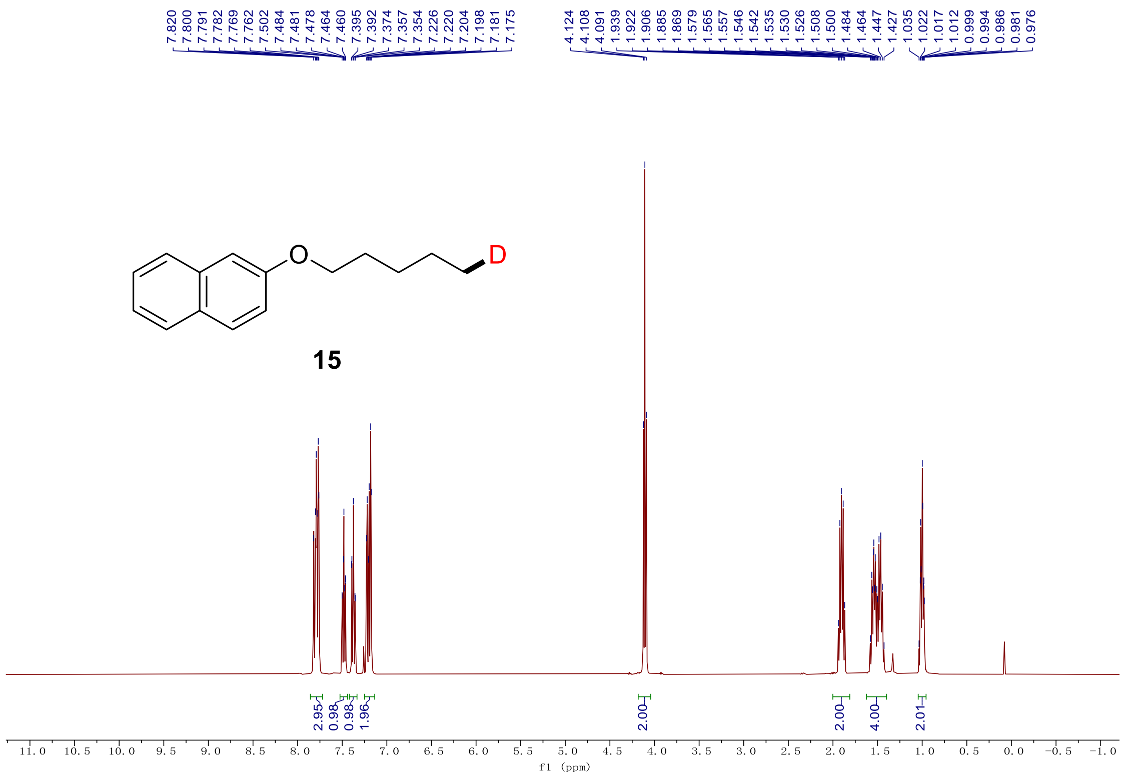

Supplementary Figure 42. ^{1}H NMR of compound **15** (400 MHz, Chloroform-*d*)

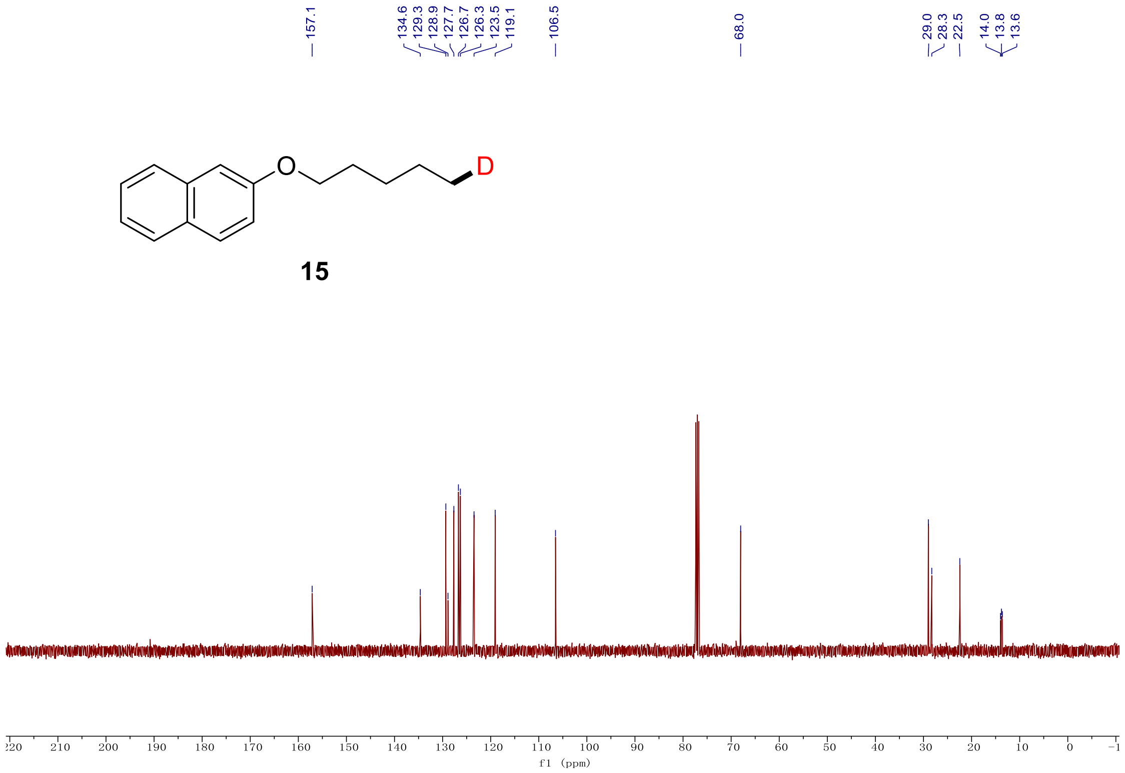

Supplementary Figure 43. ^{13}C NMR of compound **15** (100 MHz, Chloroform-*d*)

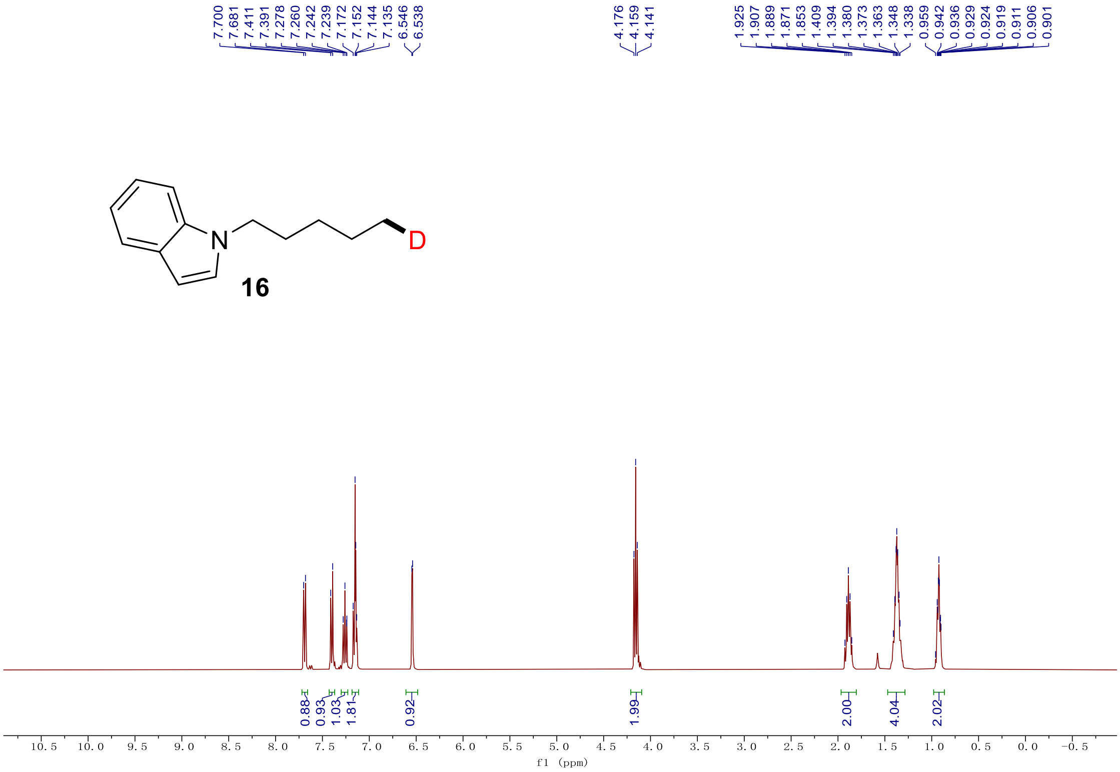

Supplementary Figure 44. ^{1}H NMR of compound **16** (400 MHz, Chloroform-*d*)

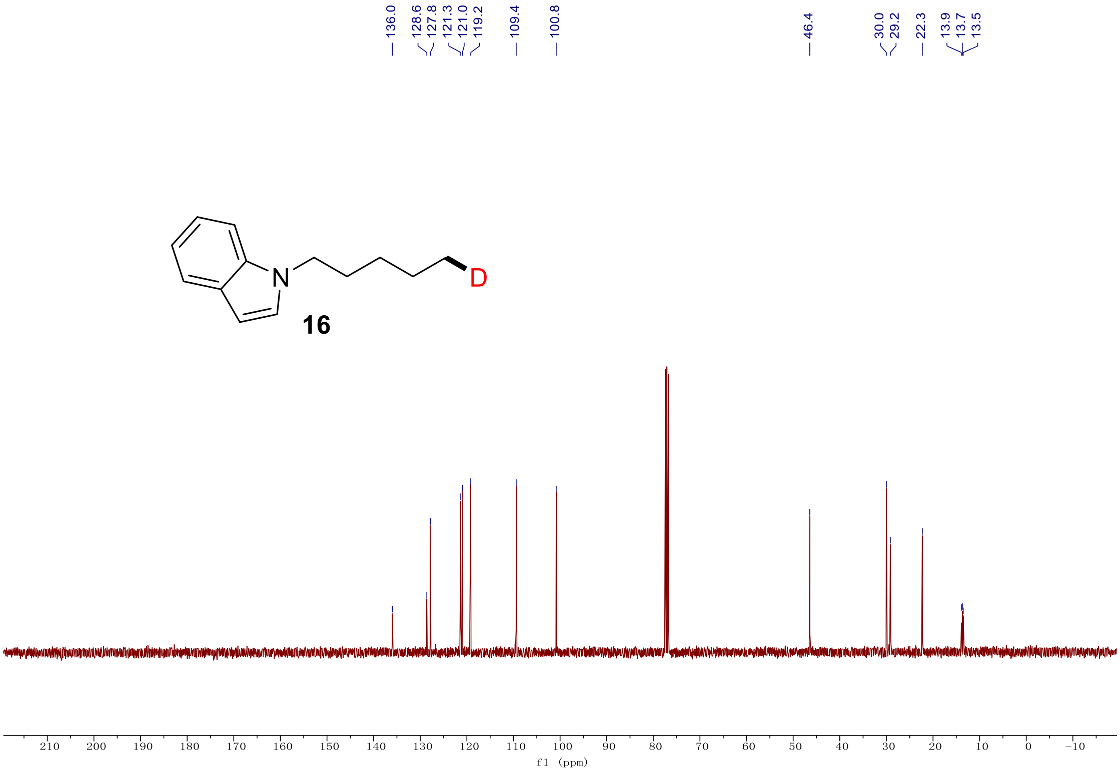

Supplementary Figure 45. ^{13}C NMR of compound **16** (100 MHz, Chloroform-*d*)

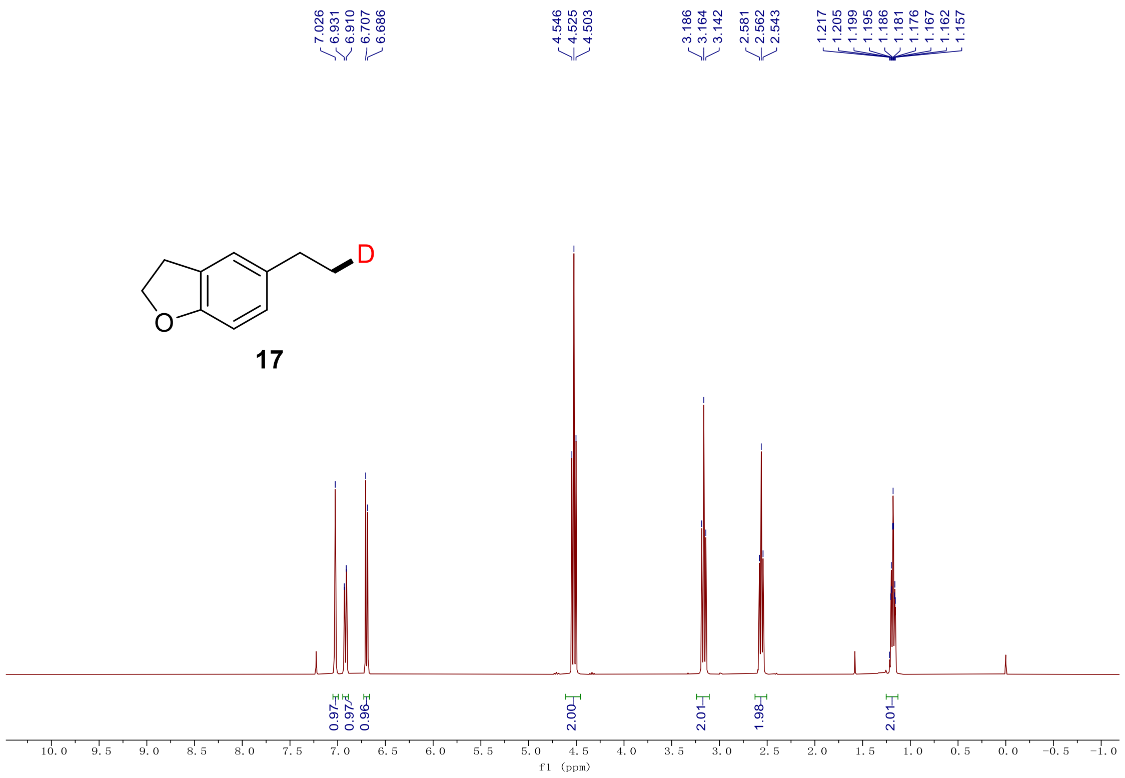

Supplementary Figure 46. ^{1}H NMR of compound **17** (400 MHz, Chloroform-*d*)

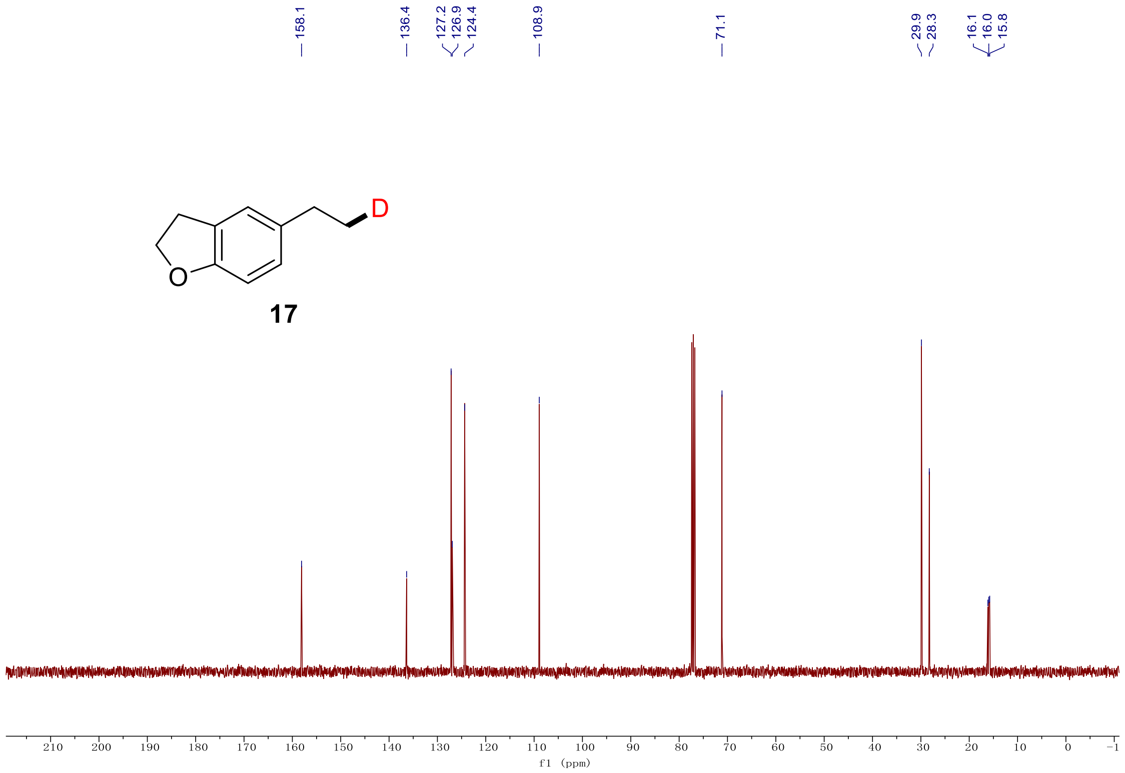

Supplementary Figure 47. ^{13}C NMR of compound **17** (100 MHz, Chloroform-*d*)

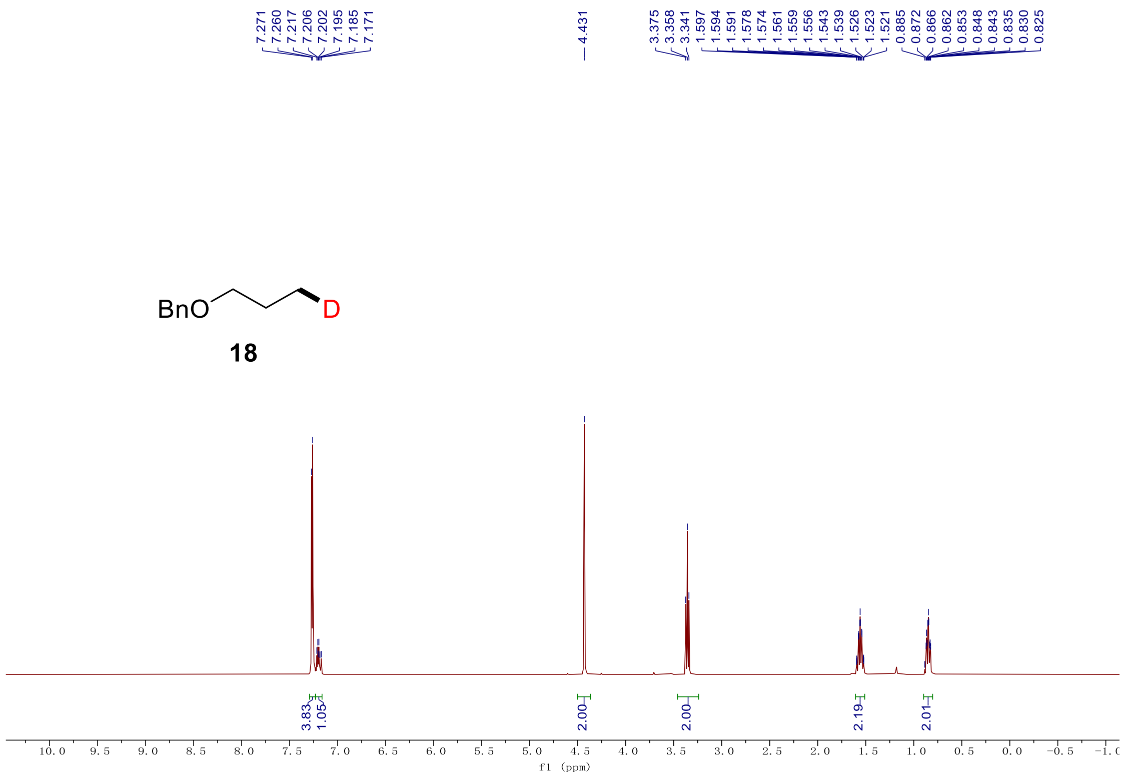

Supplementary Figure 48. ^{1}H NMR of compound **18** (400 MHz, Chloroform-*d*)

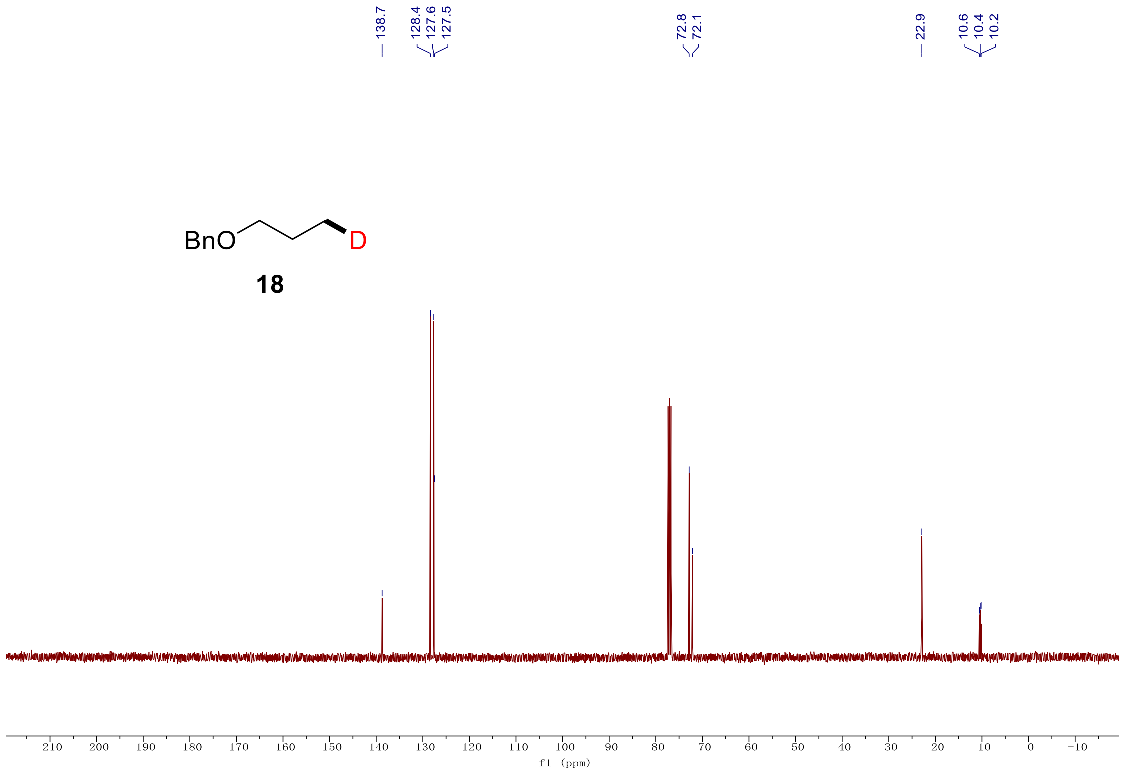

Supplementary Figure 49. ^{13}C NMR of compound **18** (100 MHz, Chloroform-*d*)

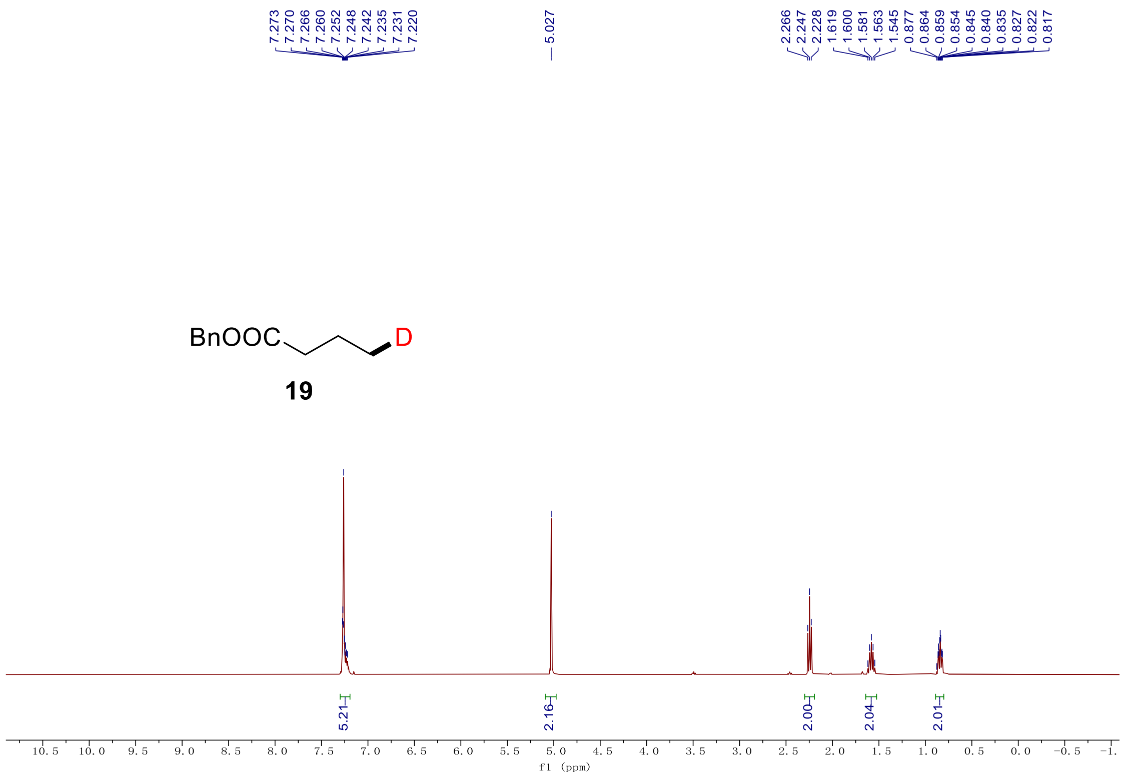

Supplementary Figure 50. ^{1}H NMR of compound **19** (400 MHz, Chloroform-*d*)

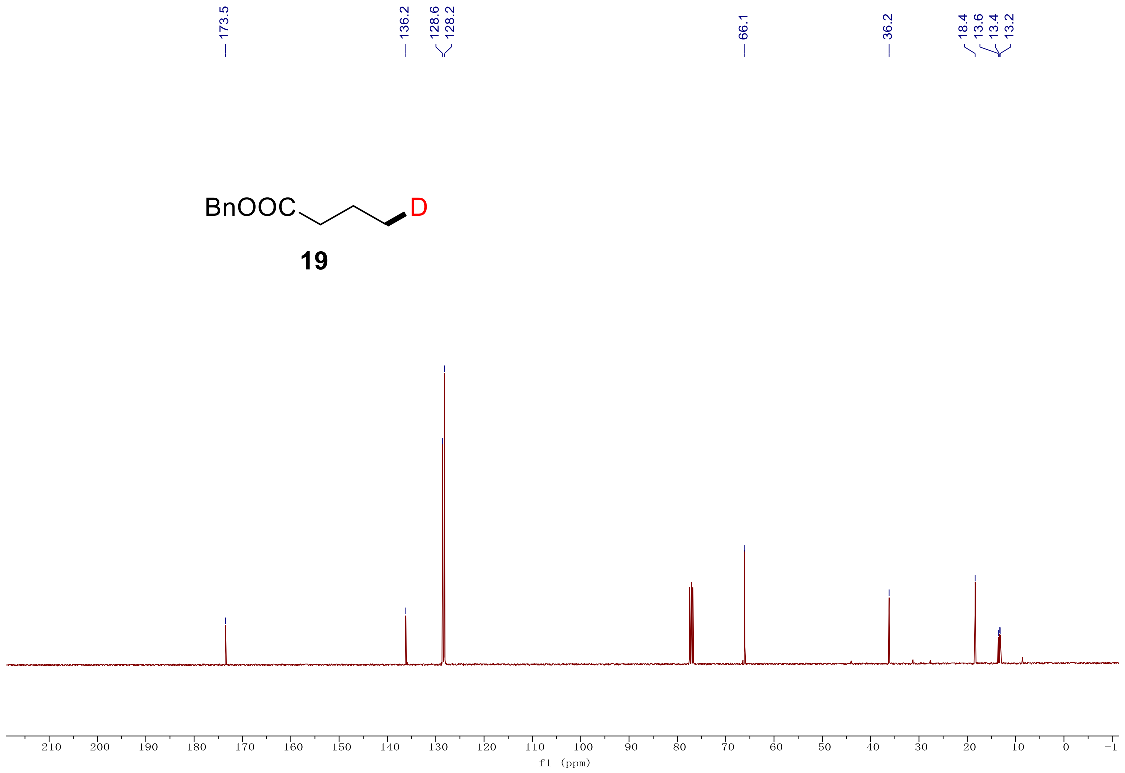

Supplementary Figure 51. ^{13}C NMR of compound **19** (100 MHz, Chloroform-*d*)

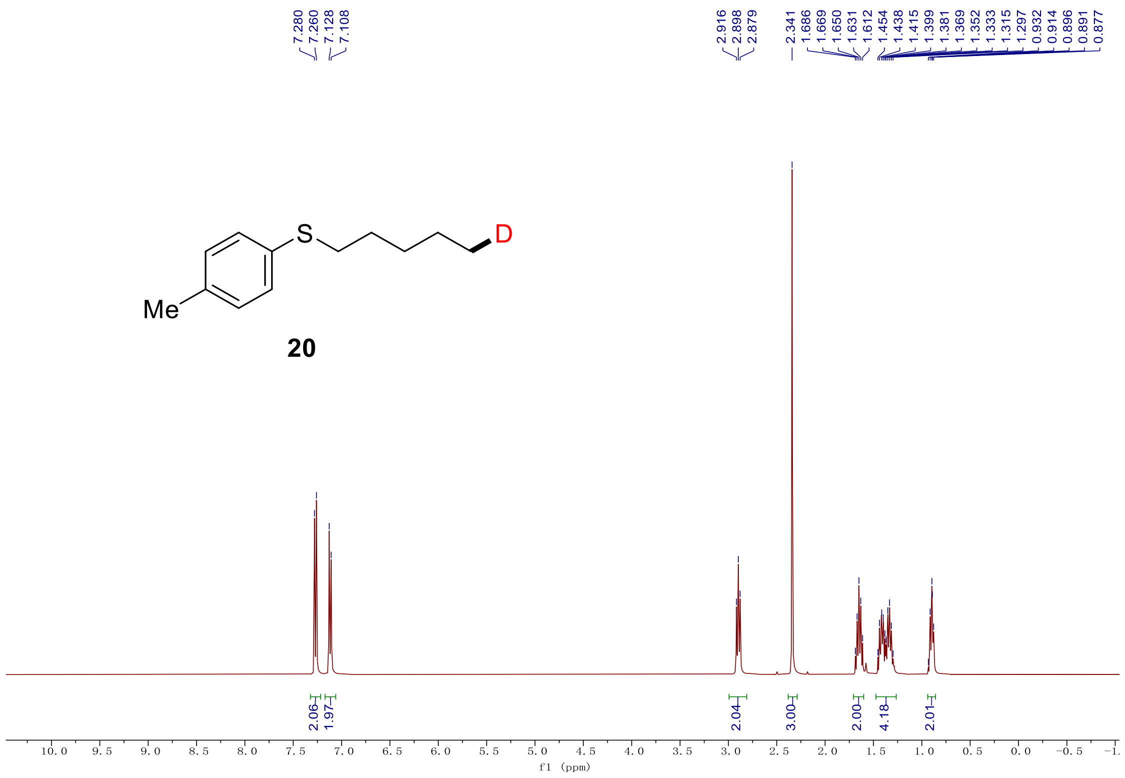

Supplementary Figure 52. ^{1}H NMR of compound **20** (400 MHz, Chloroform-*d*)

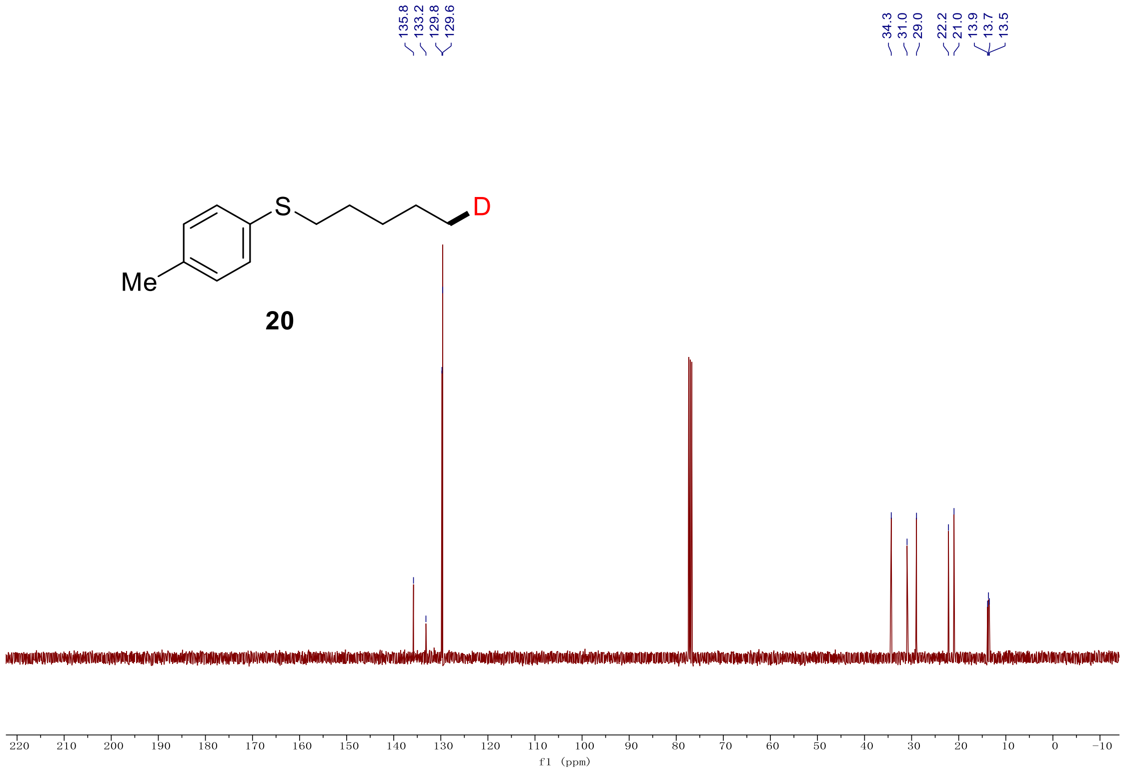

Supplementary Figure 53. ^{13}C NMR of compound **20** (100 MHz, Chloroform-*d*)

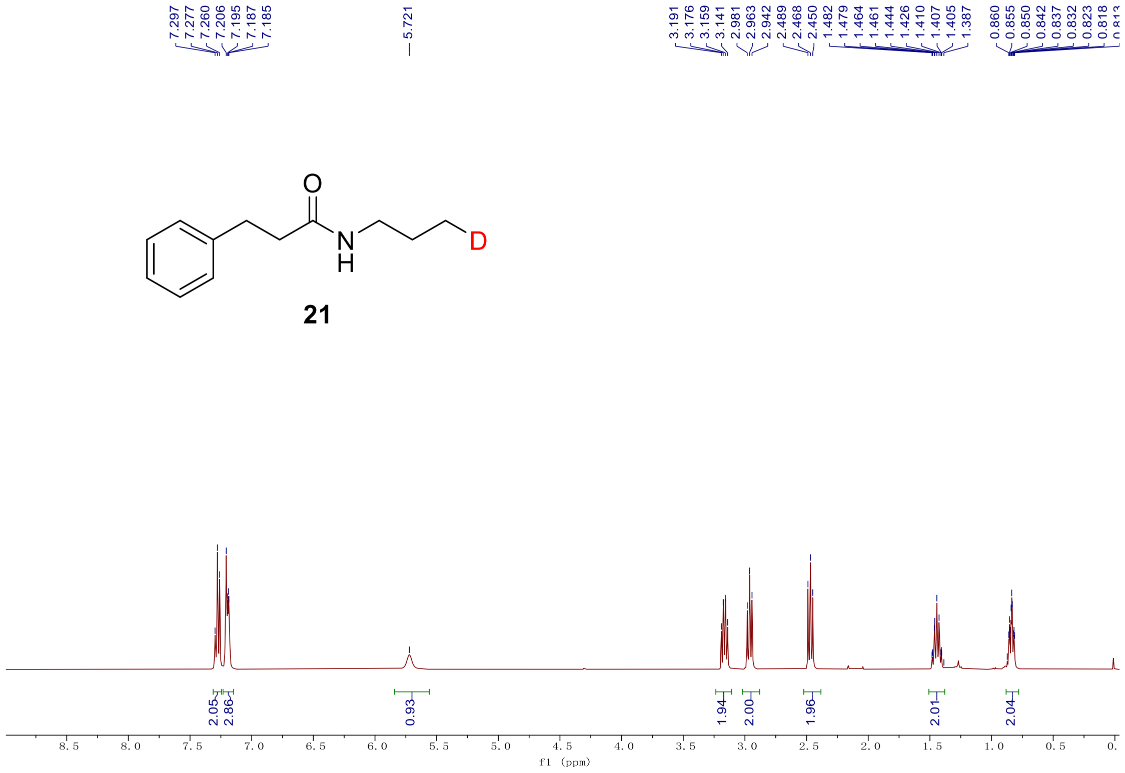

Supplementary Figure 54. ^{1}H NMR of compound **21** (400 MHz, Chloroform-*d*)

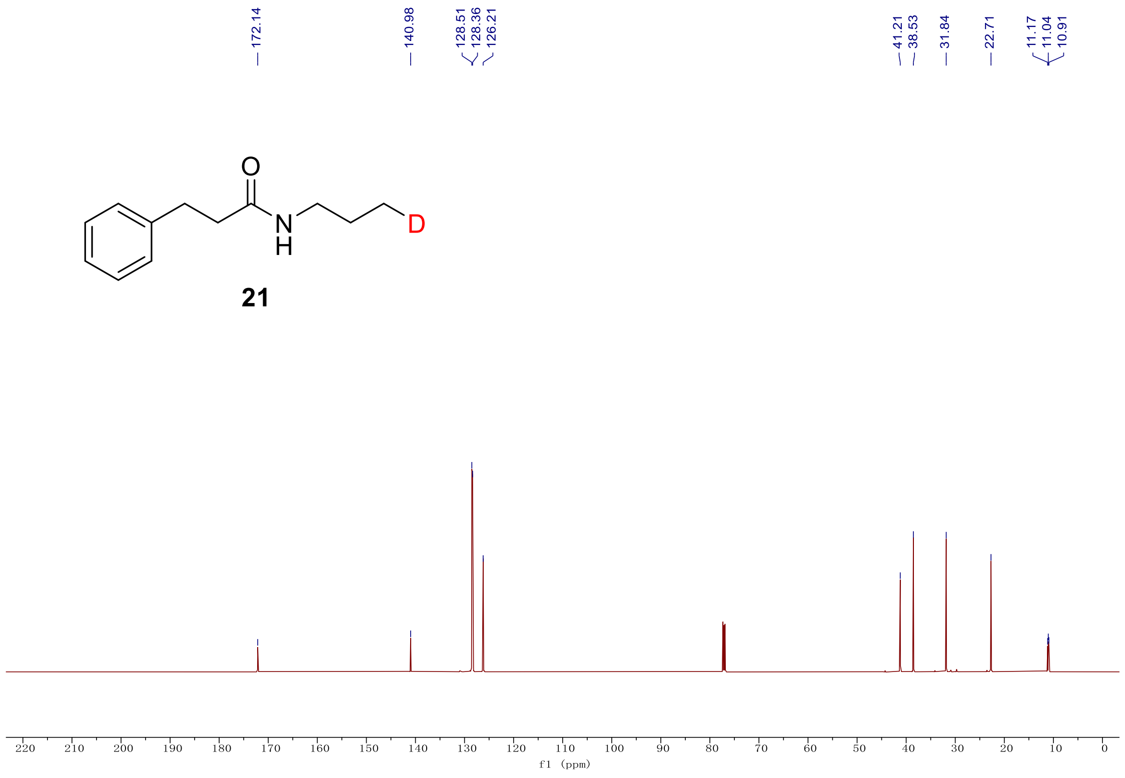

Supplementary Figure 55. ^{13}C NMR of compound **21** (100 MHz, Chloroform-*d*)

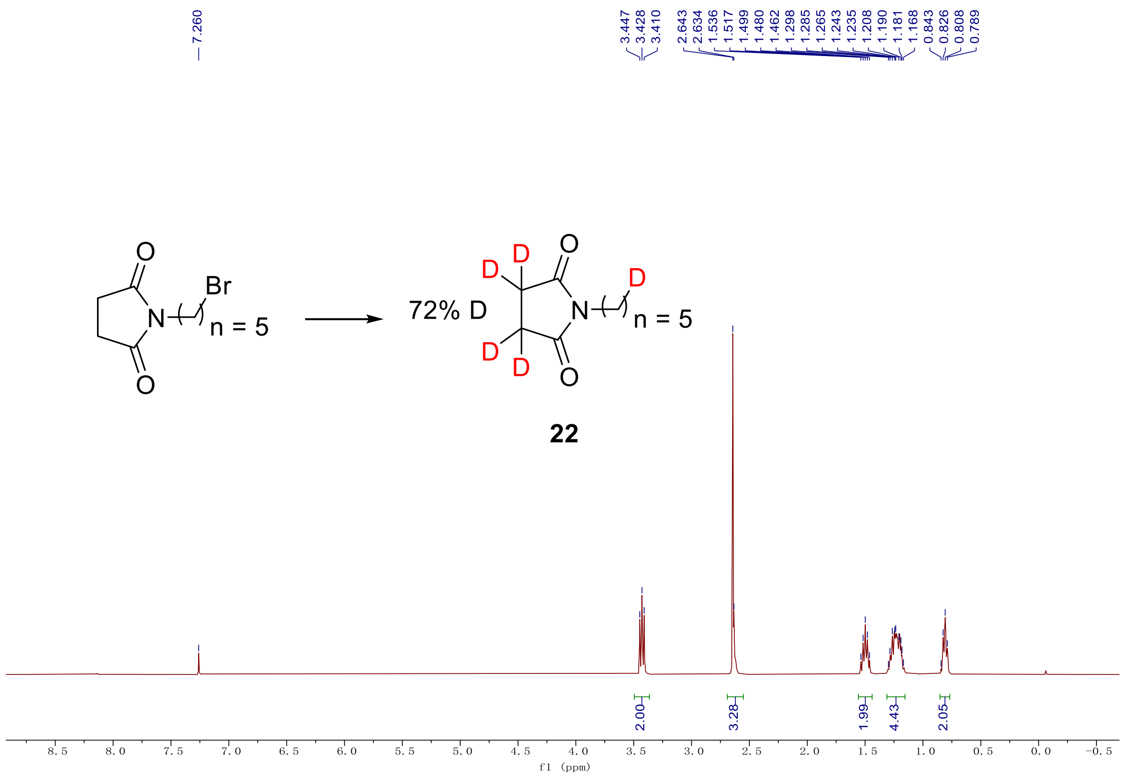

Supplementary Figure 56. ^{1}H NMR of compound **22** (400 MHz, Chloroform-*d*)

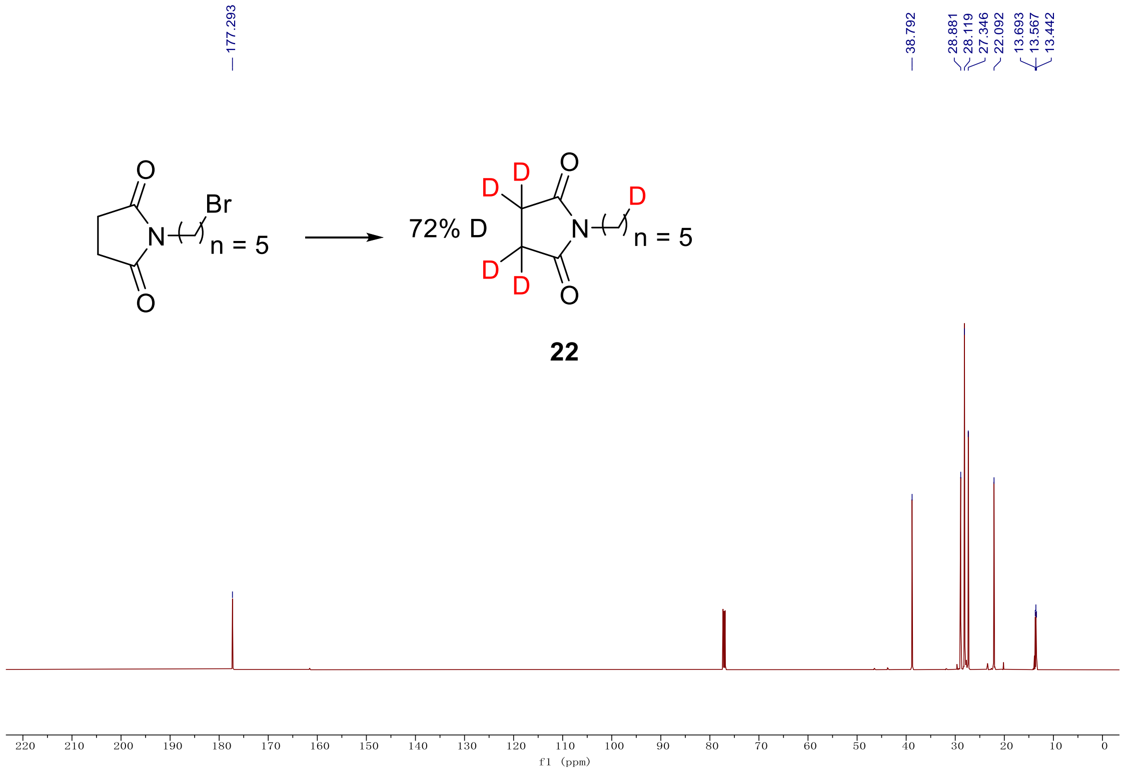

Supplementary Figure 57. ^{13}C NMR of compound **22** (100 MHz, Chloroform-*d*)

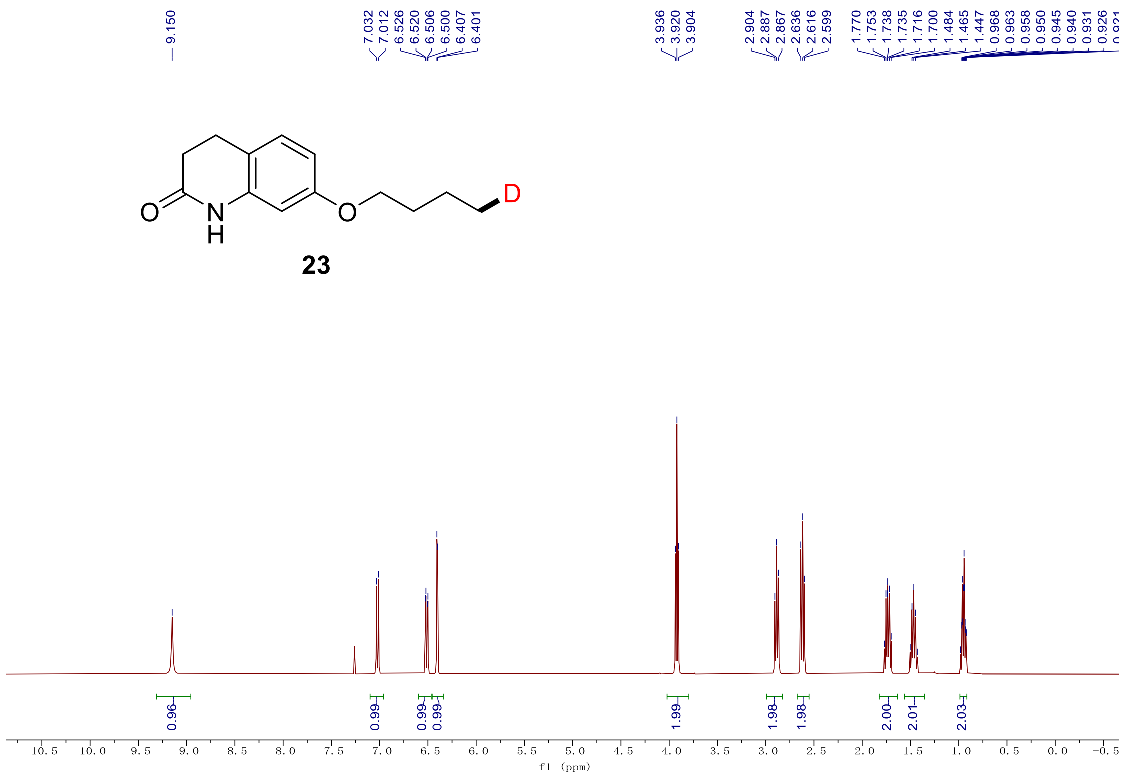

Supplementary Figure 58. ^{1}H NMR of compound 23 (400 MHz, Chloroform-*d*)

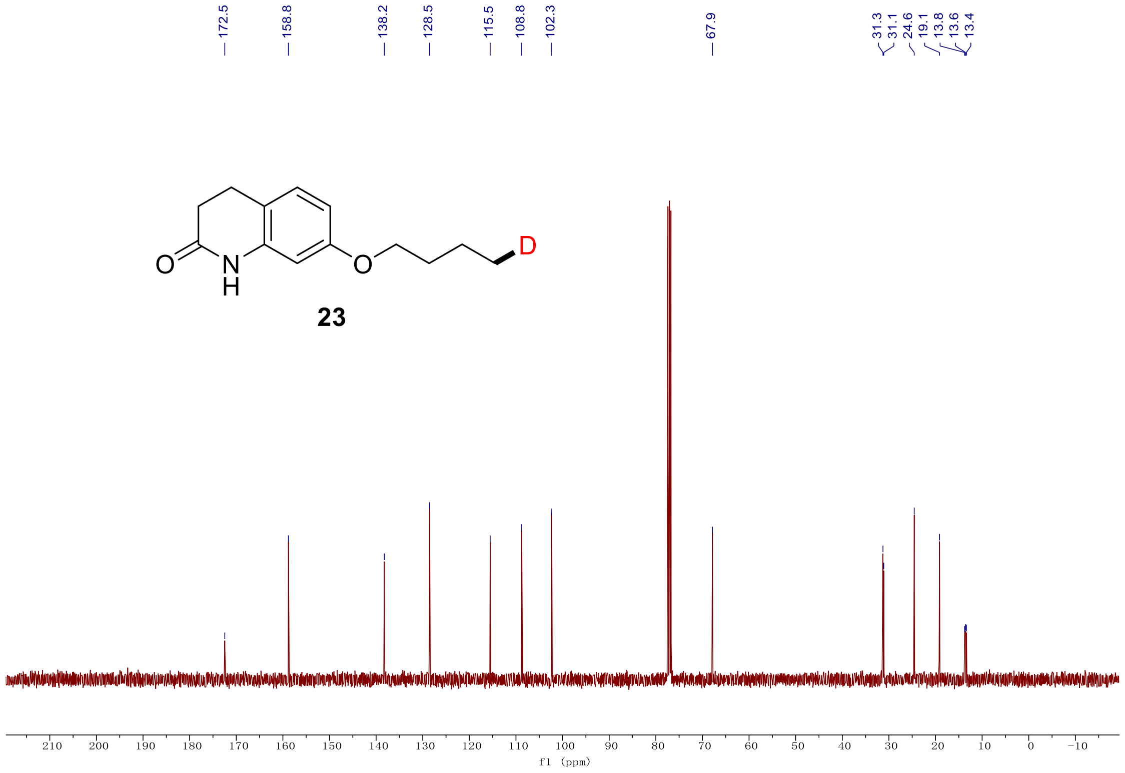

Supplementary Figure 59. ^{13}C NMR of compound **23** (100 MHz, Chloroform-*d*)

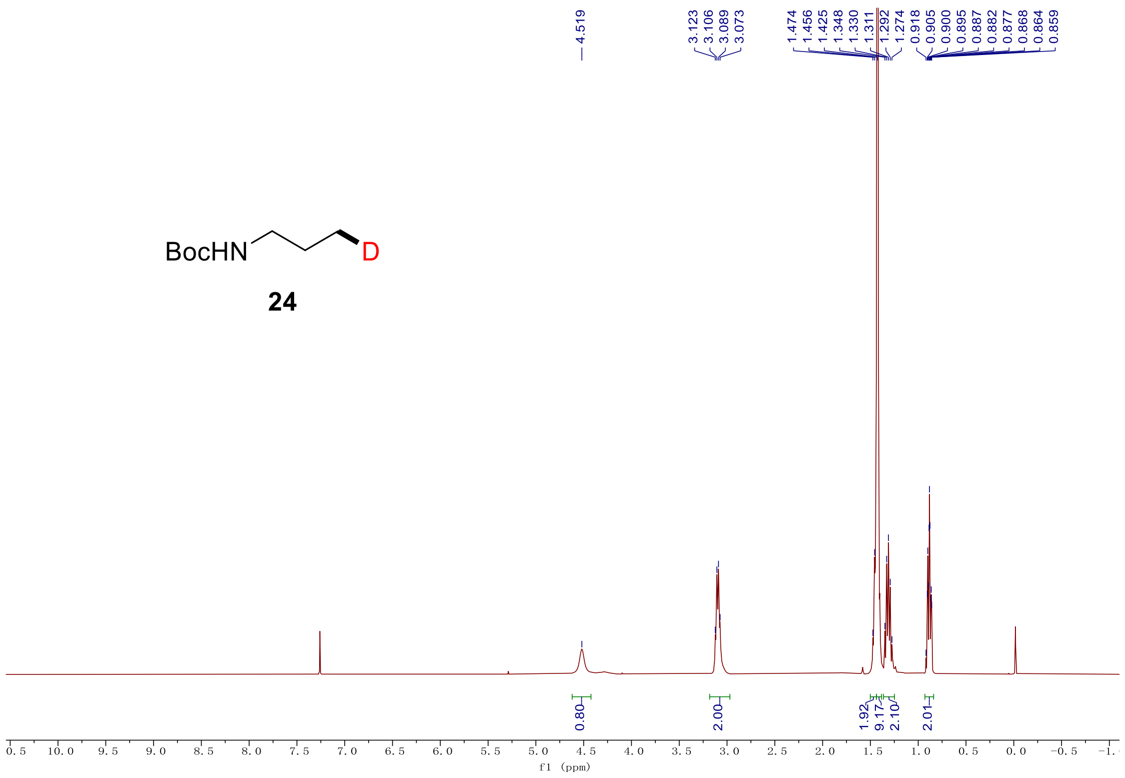

Supplementary Figure 60. ^{1}H NMR of compound **24** (400 MHz, Chloroform-*d*)

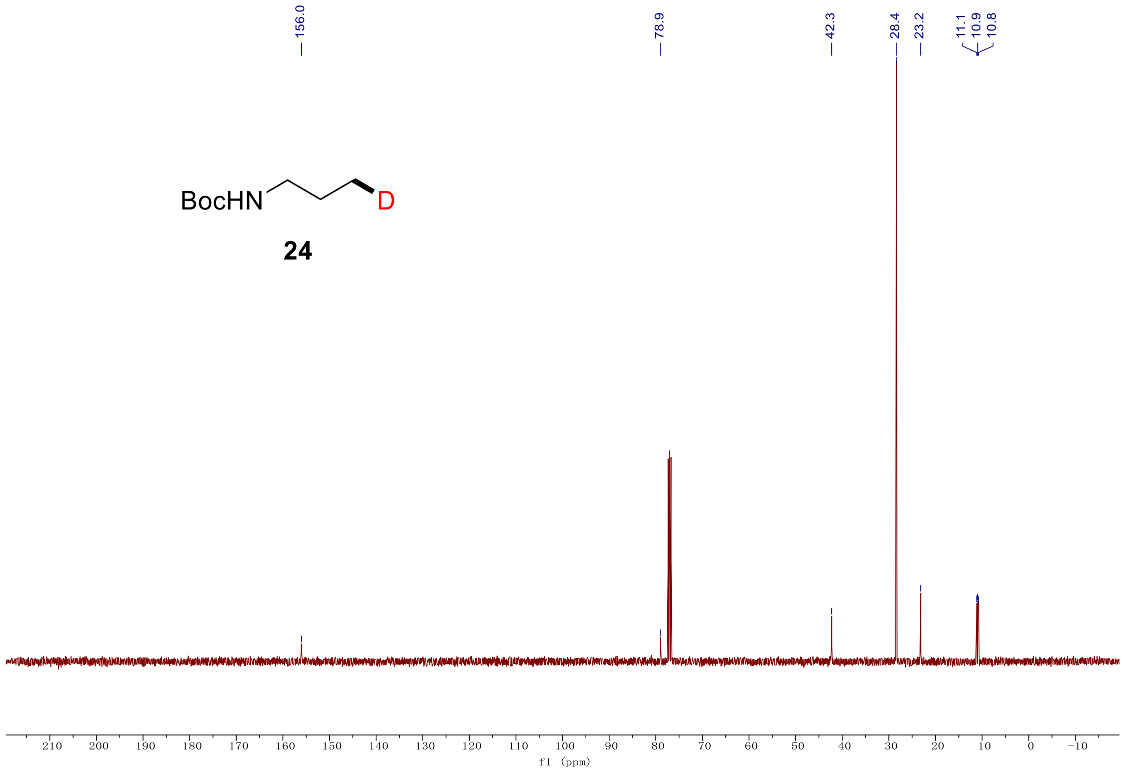

Supplementary Figure 61. ^{13}C NMR of compound **24** (100 MHz, Chloroform-*d*)

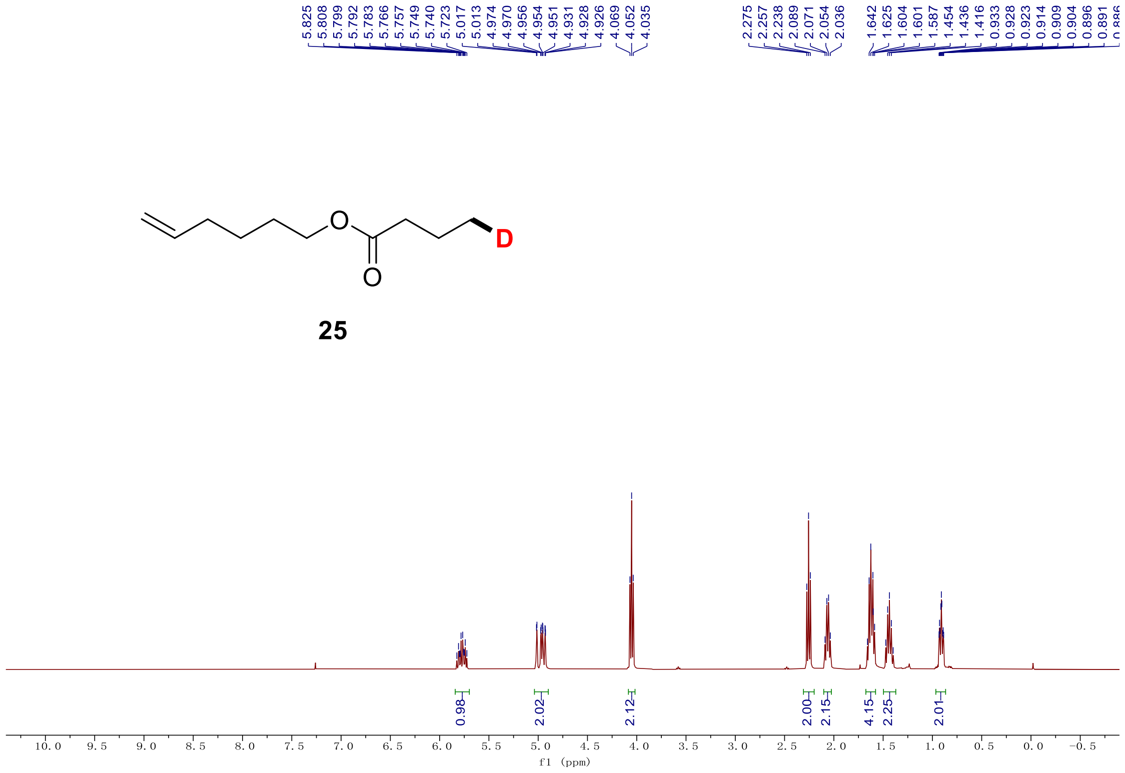

Supplementary Figure 62. ^{1}H NMR of compound **25** (400 MHz, Chloroform-*d*)

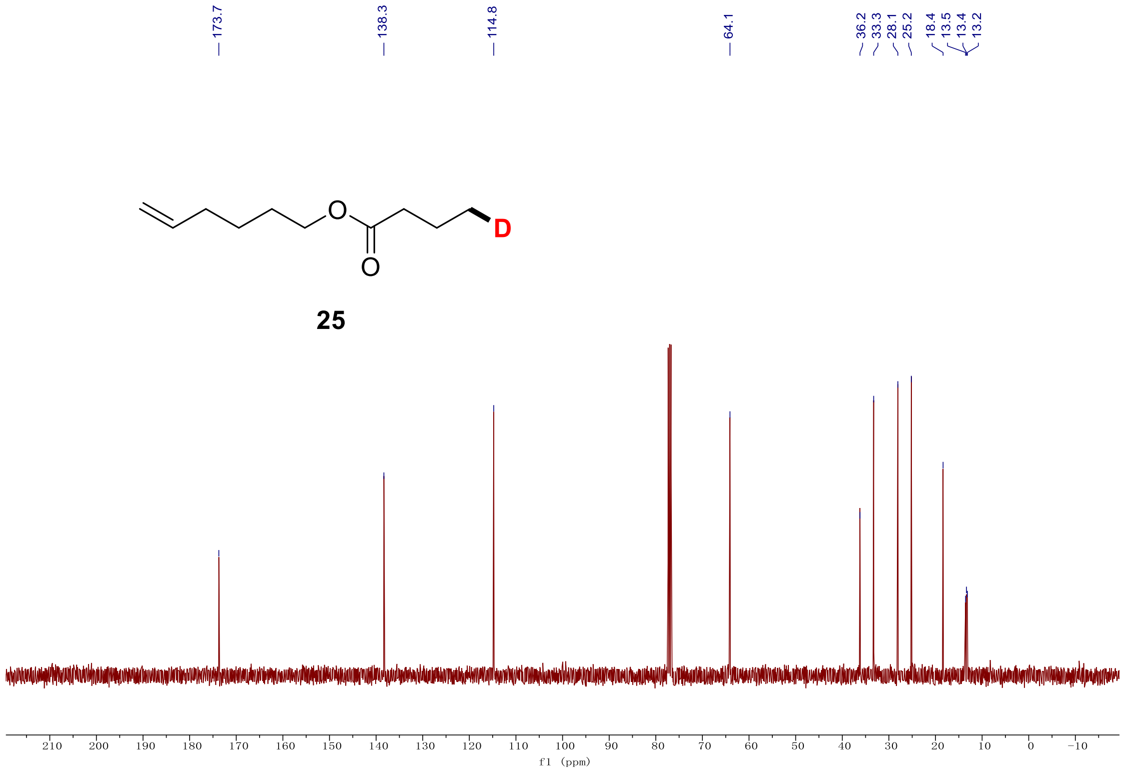

Supplementary Figure 63. ^{13}C NMR of compound **25** (100 MHz, Chloroform-*d*)

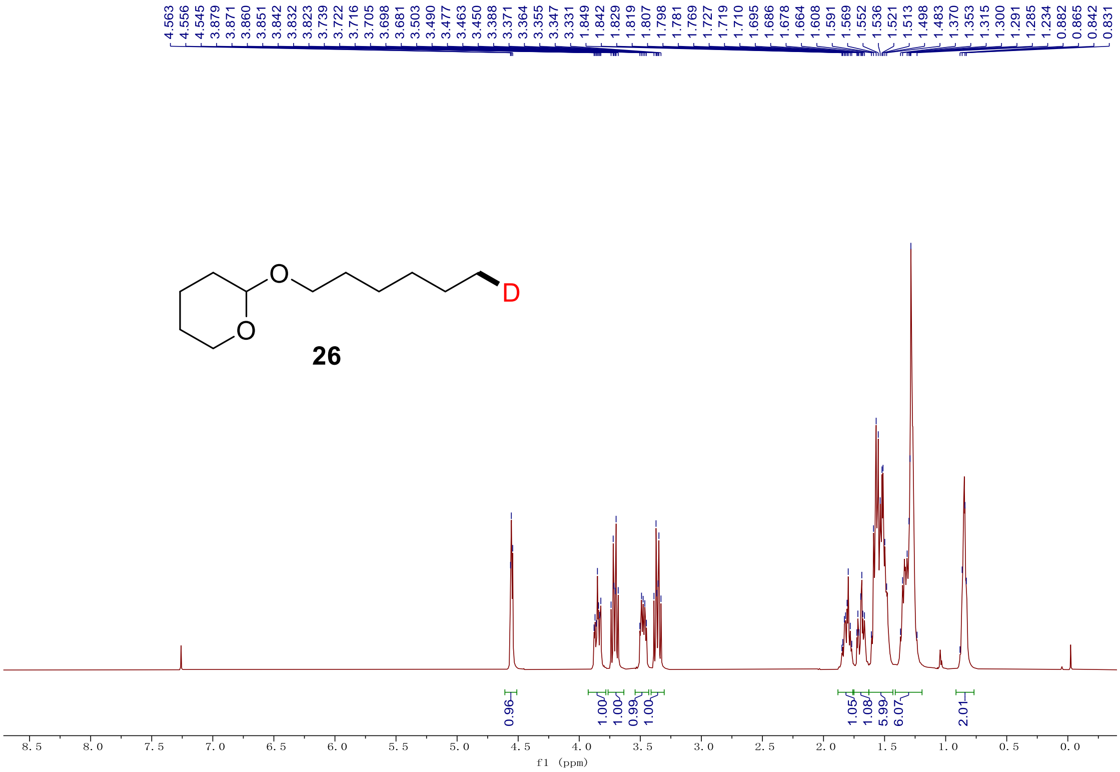

Supplementary Figure 64. ^{1}H NMR of compound **26** (400 MHz, Chloroform-*d*)

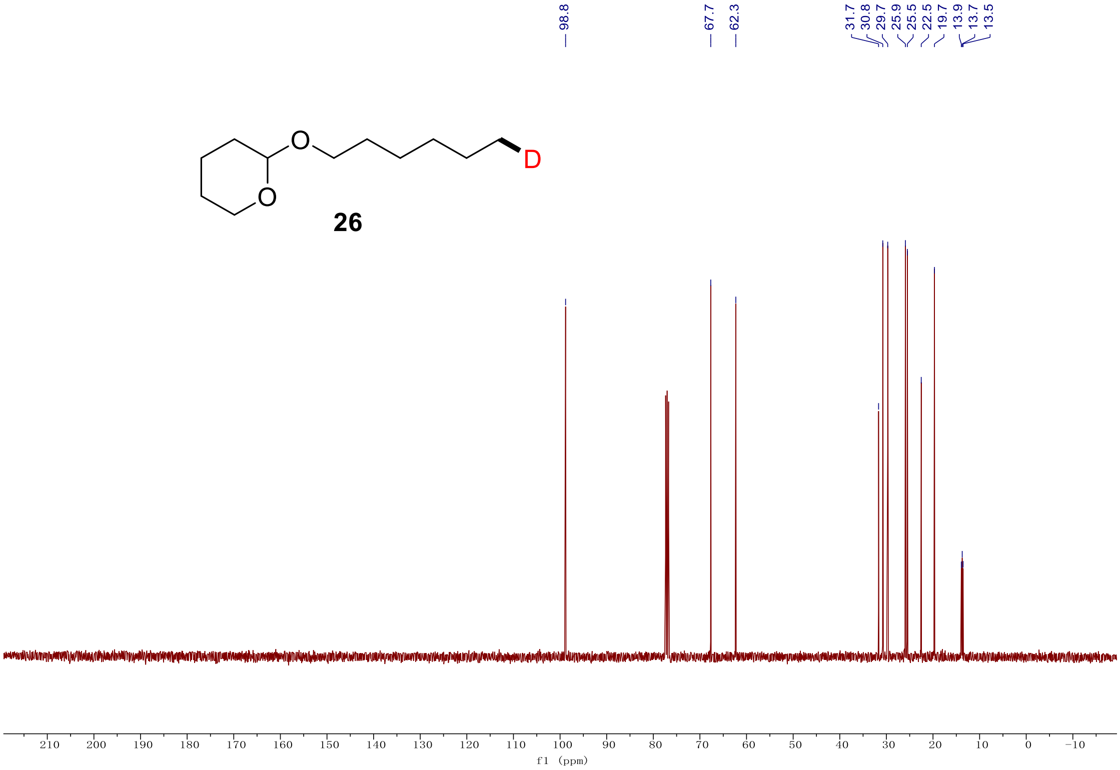

Supplementary Figure 65. ^{13}C NMR of compound **26** (100 MHz, Chloroform-*d*)

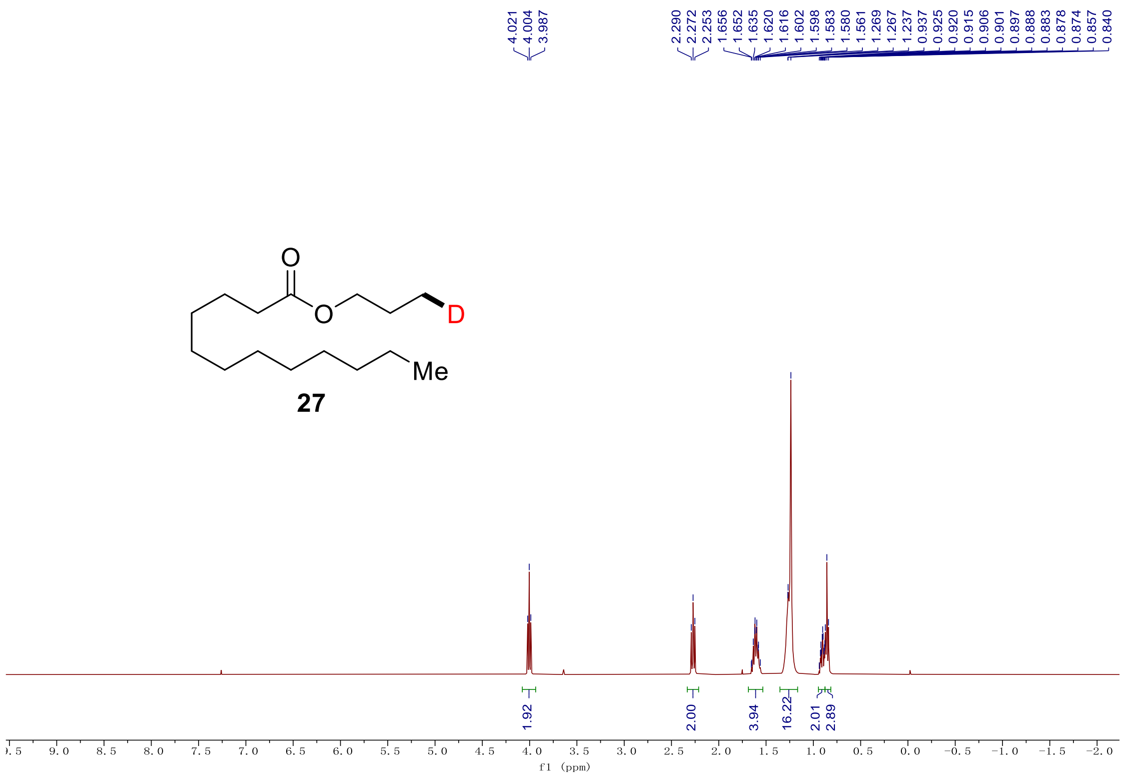

Supplementary Figure 66. ^{1}H NMR of compound **27** (400 MHz, Chloroform-*d*)

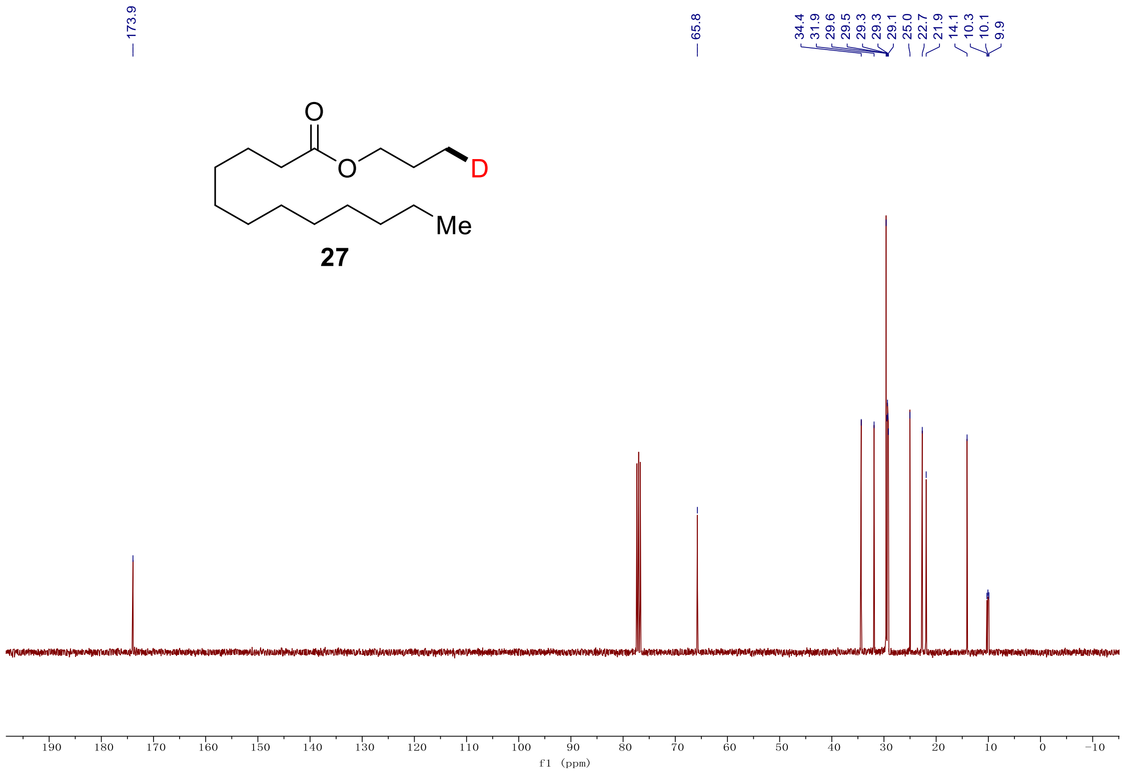

Supplementary Figure 67. ^{13}C NMR of compound **27** (100 MHz, Chloroform-*d*)

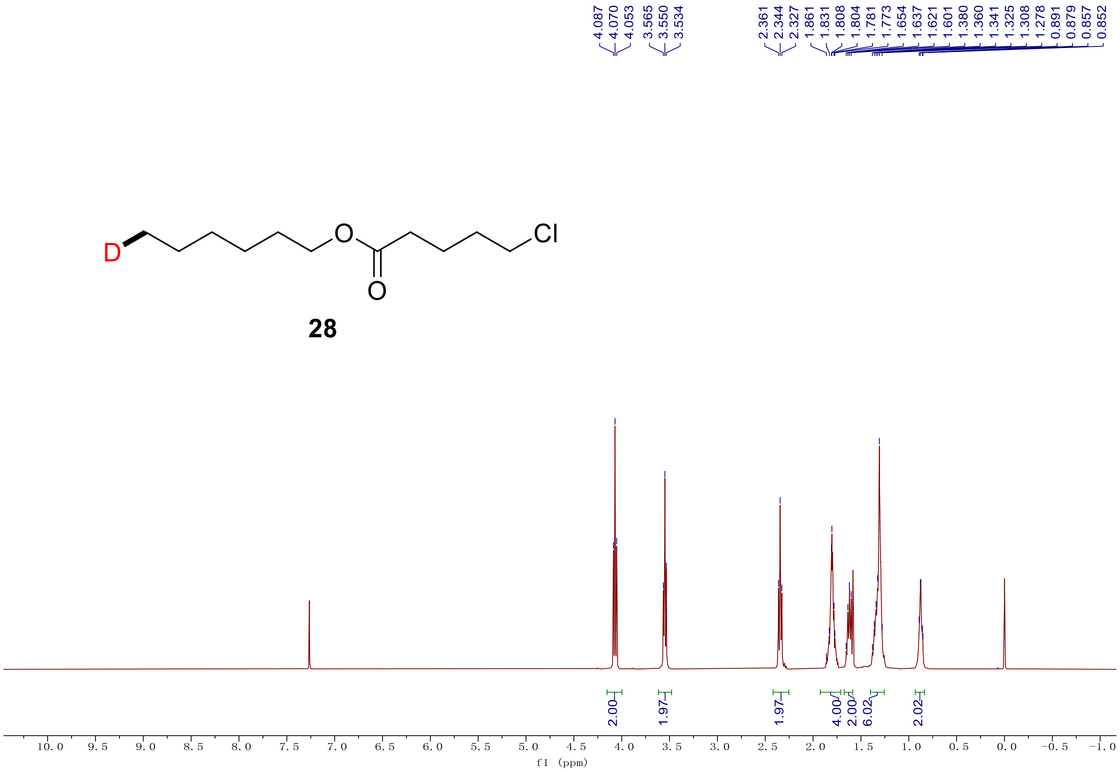

Supplementary Figure 68. ^{1}H NMR of compound **28** (400 MHz, Chloroform-*d*)

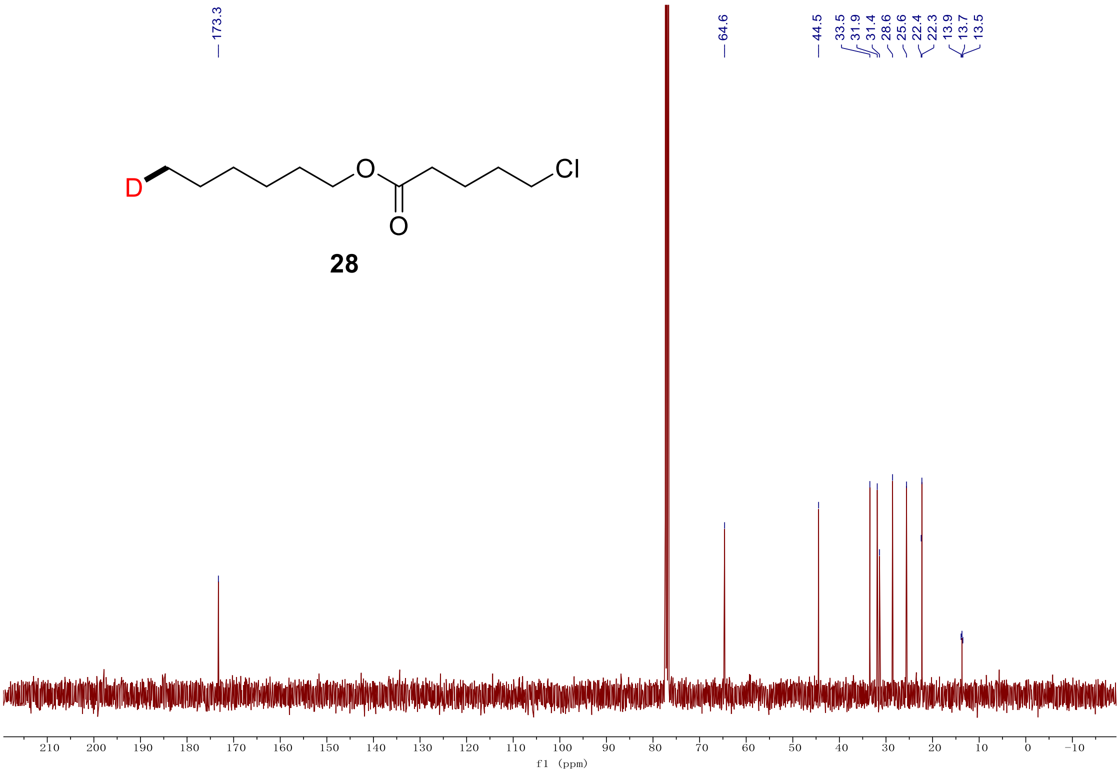

Supplementary Figure 69. ^{13}C NMR of compound **28** (100 MHz, Chloroform-*d*)

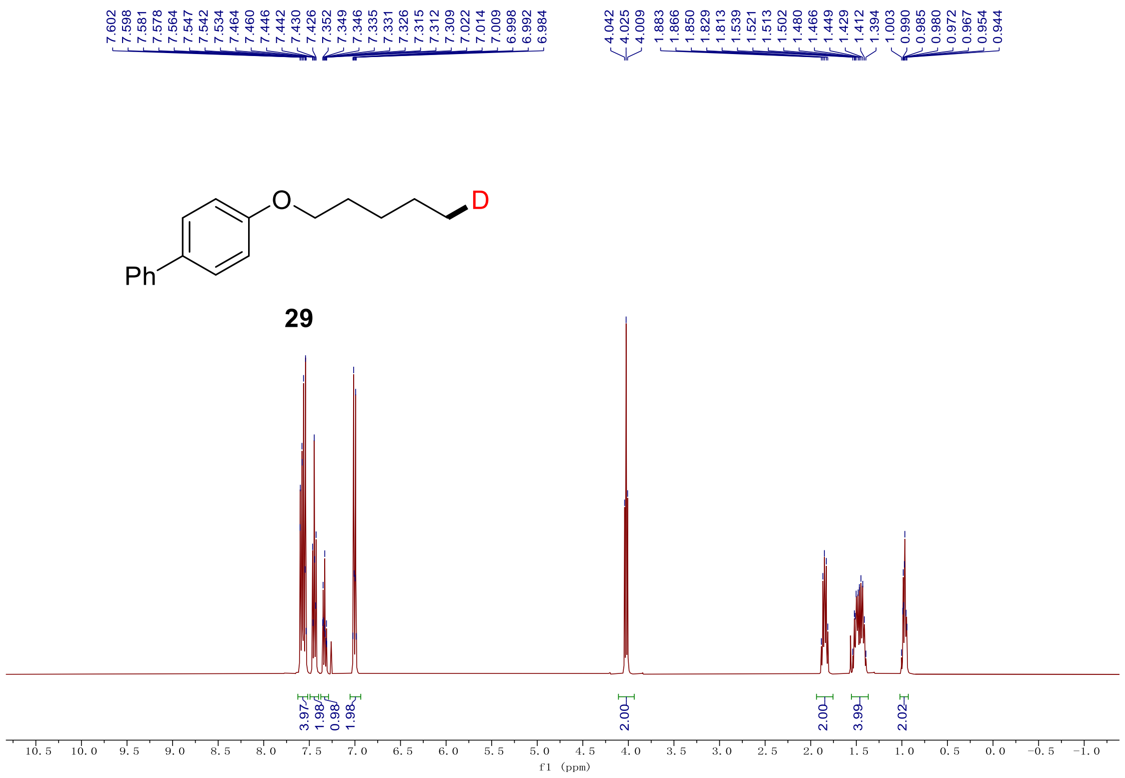

Supplementary Figure 70. ^{1}H NMR of compound **29** (400 MHz, Chloroform-*d*)

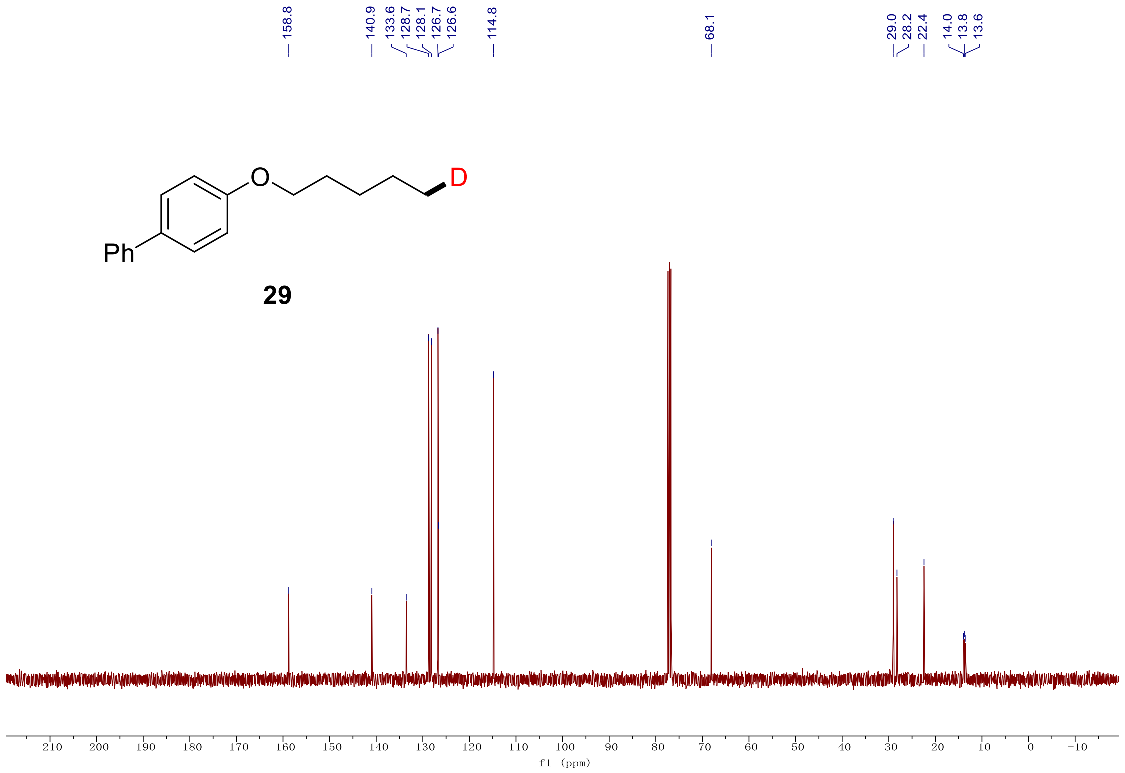

Supplementary Figure 71. ^{13}C NMR of compound **29** (100 MHz, Chloroform-*d*)

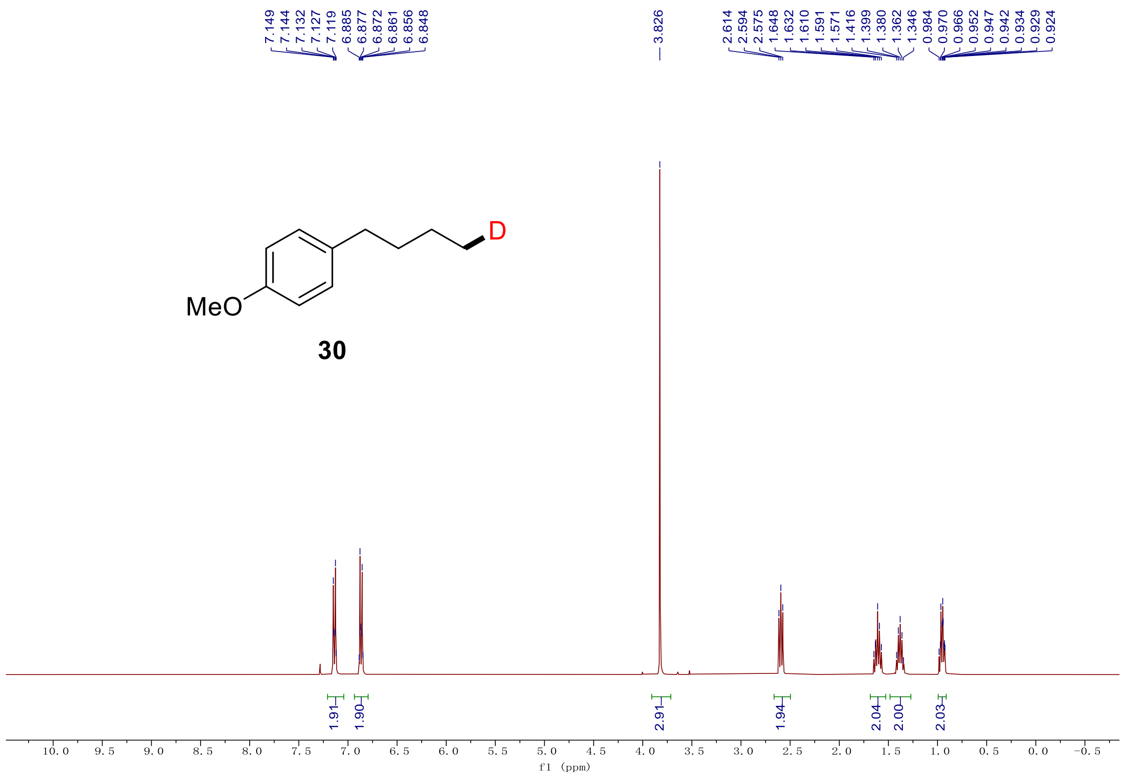

Supplementary Figure 72. ^{1}H NMR of compound **30** (400 MHz, Chloroform-*d*)

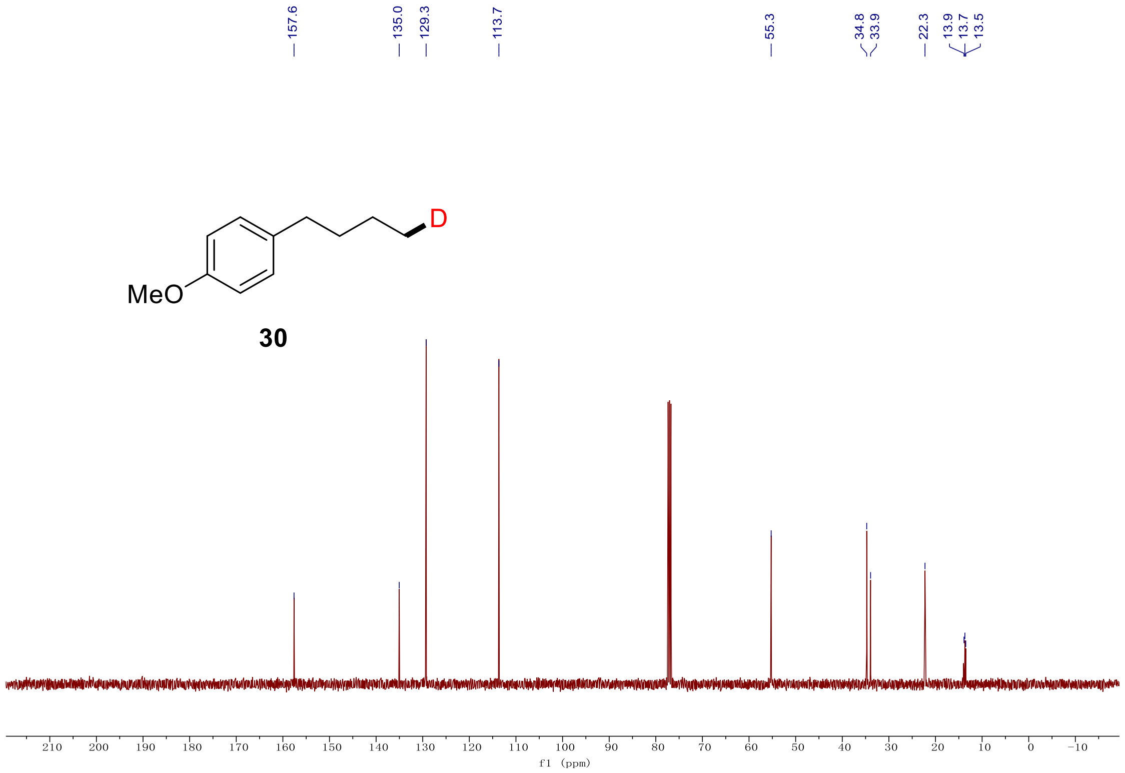

Supplementary Figure 73. ^{13}C NMR of compound **30** (100 MHz, Chloroform-*d*)

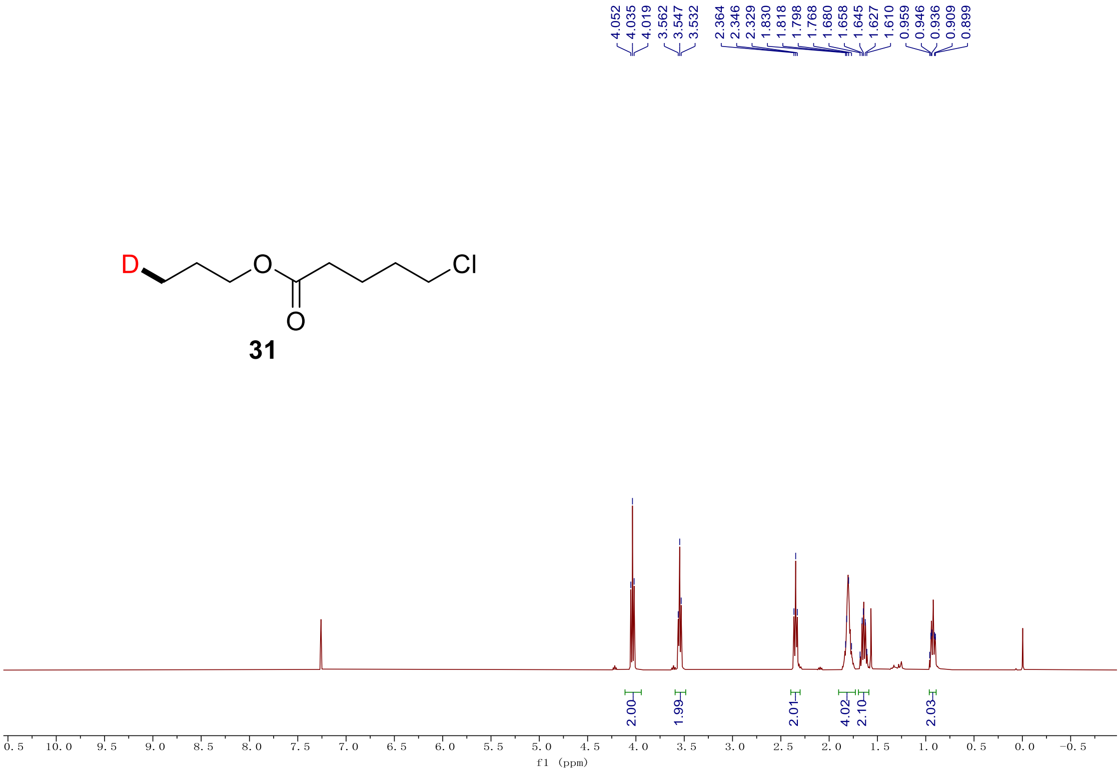

Supplementary Figure 74. ^{1}H NMR of compound **31** (400 MHz, Chloroform-*d*)

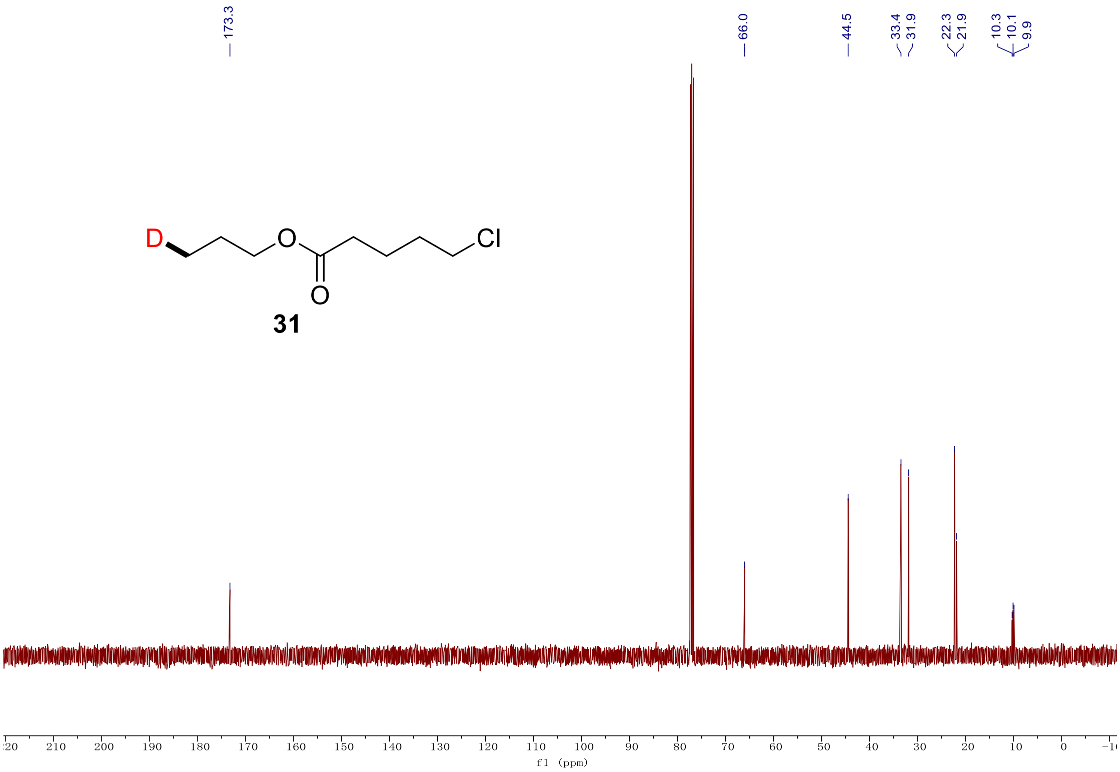

Supplementary Figure 75. ^{13}C NMR of compound **31** (100 MHz, Chloroform-*d*)

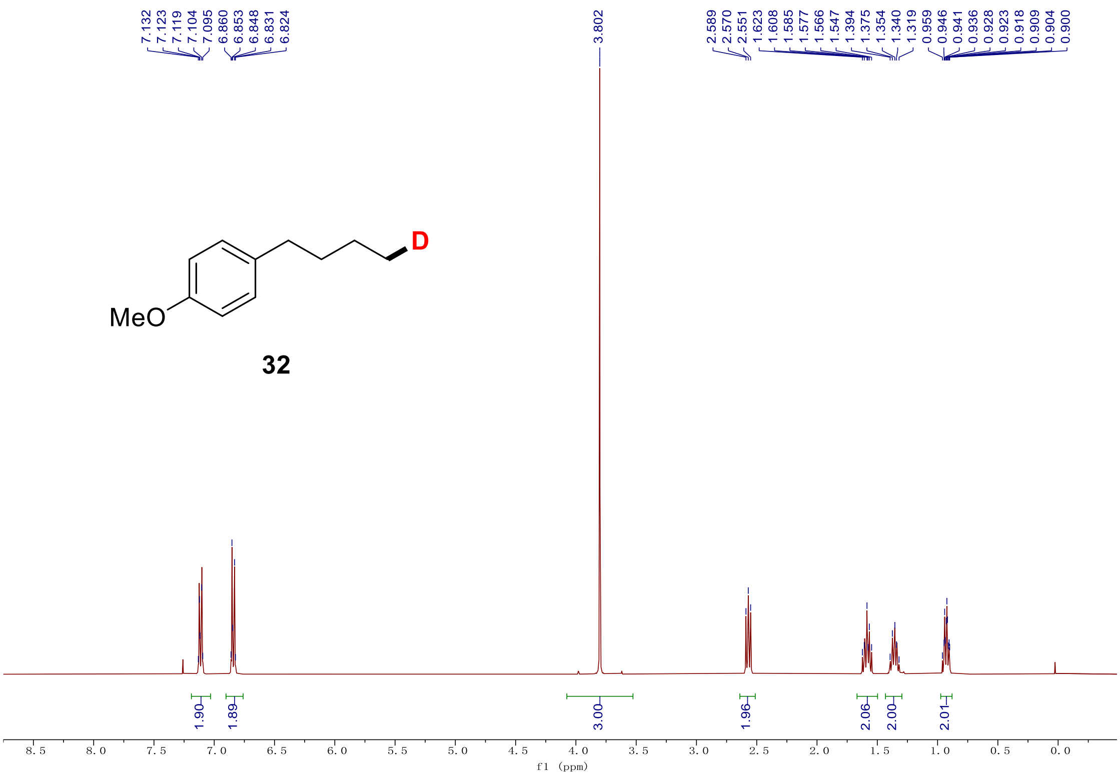

Supplementary Figure 76. ^{1}H NMR of compound **32** (400 MHz, Chloroform-*d*)

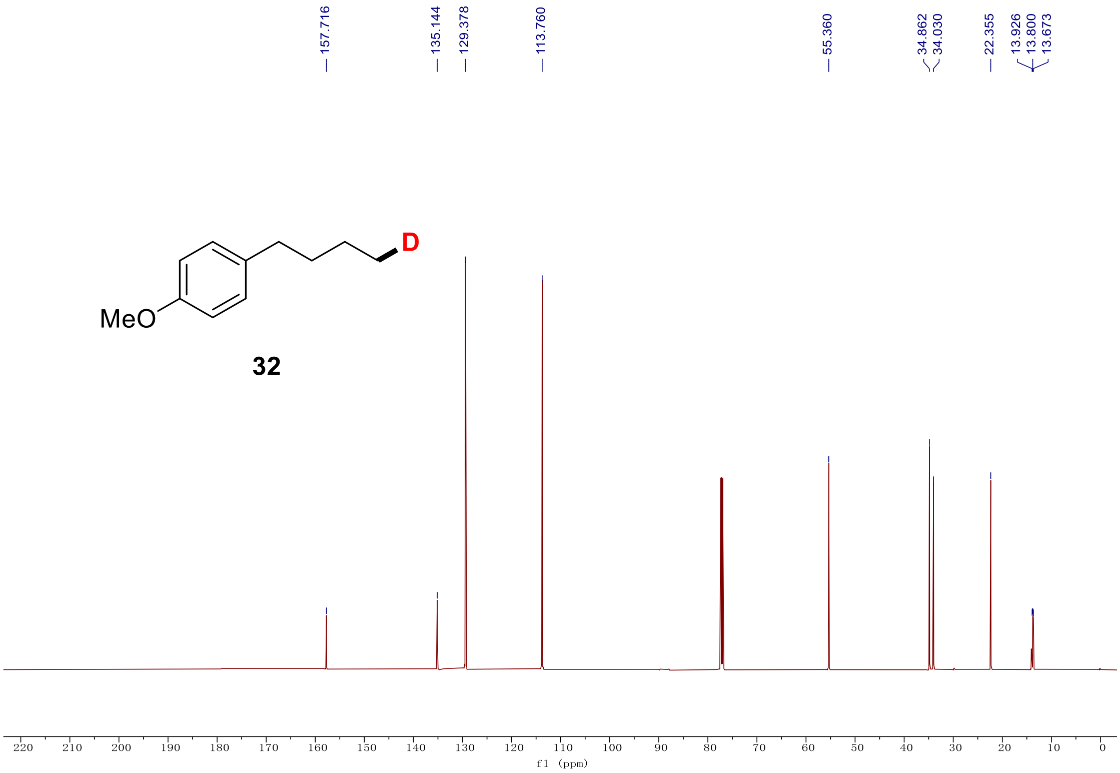

Supplementary Figure 77. ^{13}C NMR of compound 3**2** (100 MHz, Chloroform-*d*)

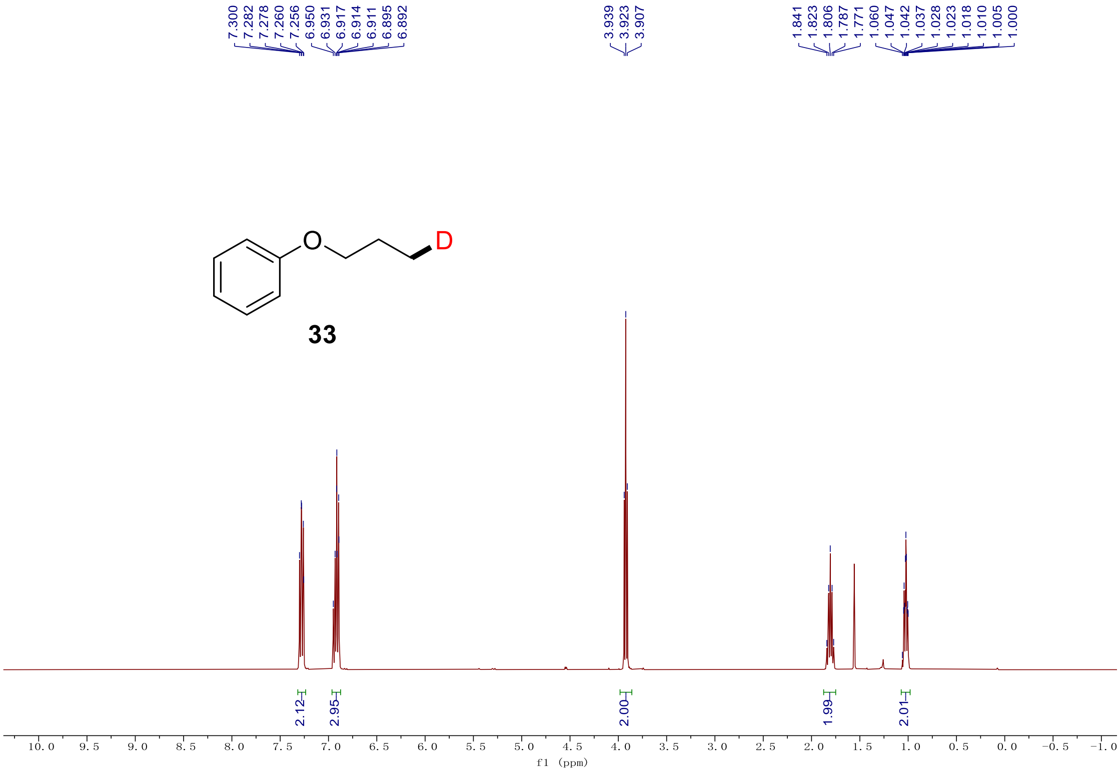

Supplementary Figure 78. ^{1}H NMR of compound **33** (400 MHz, Chloroform-*d*)

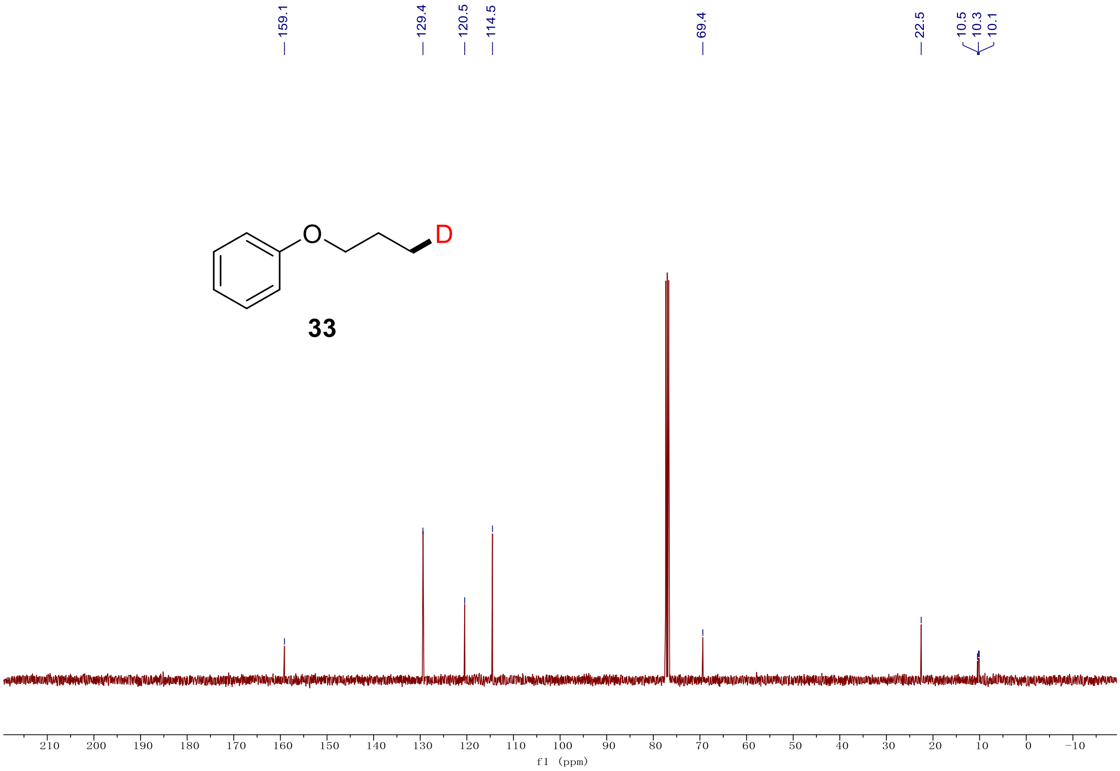

Supplementary Figure 79. ^{13}C NMR of compound **33** (100 MHz, Chloroform-*d*)

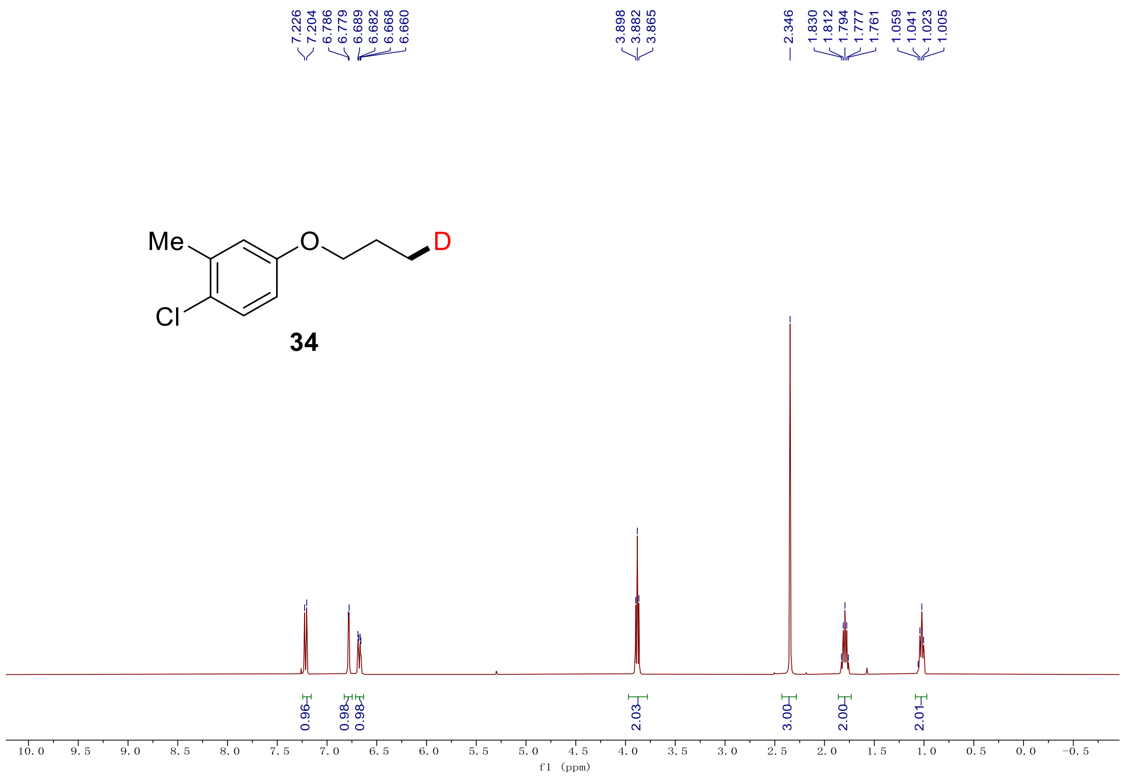

Supplementary Figure 80. ^{1}H NMR of compound **34** (400 MHz, Chloroform-*d*)

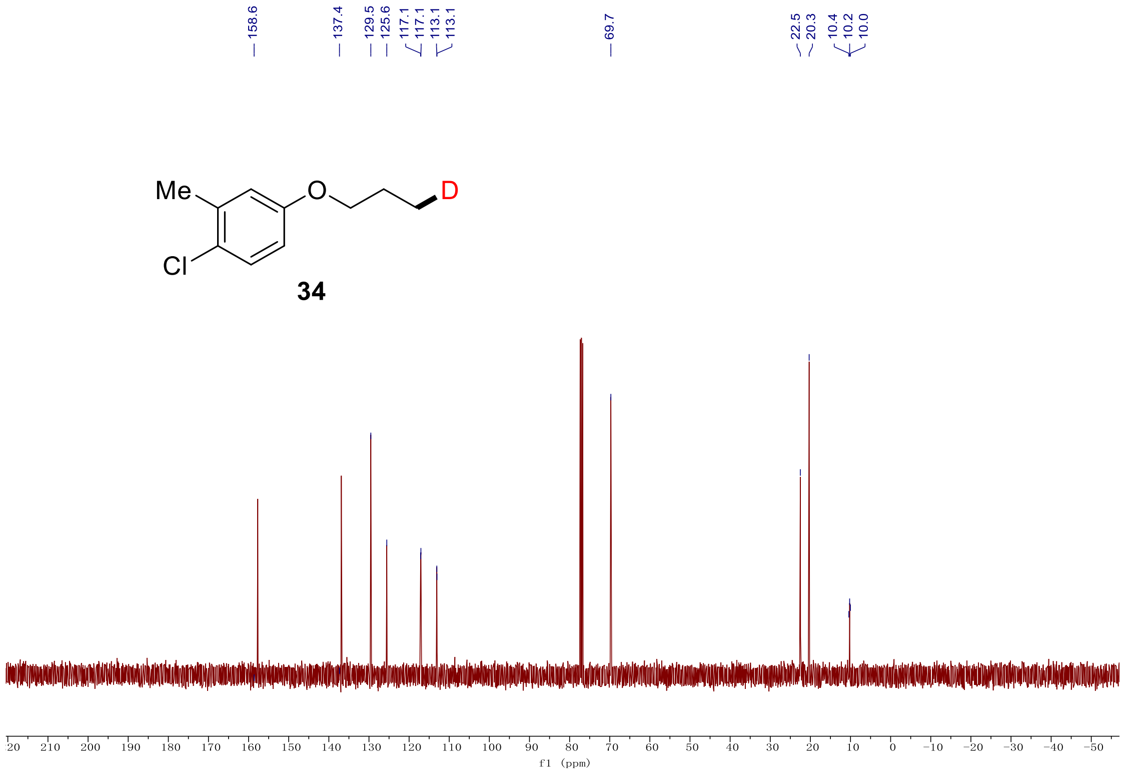

Supplementary Figure 81. ^{13}C NMR of compound **34** (100 MHz, Chloroform-*d*)

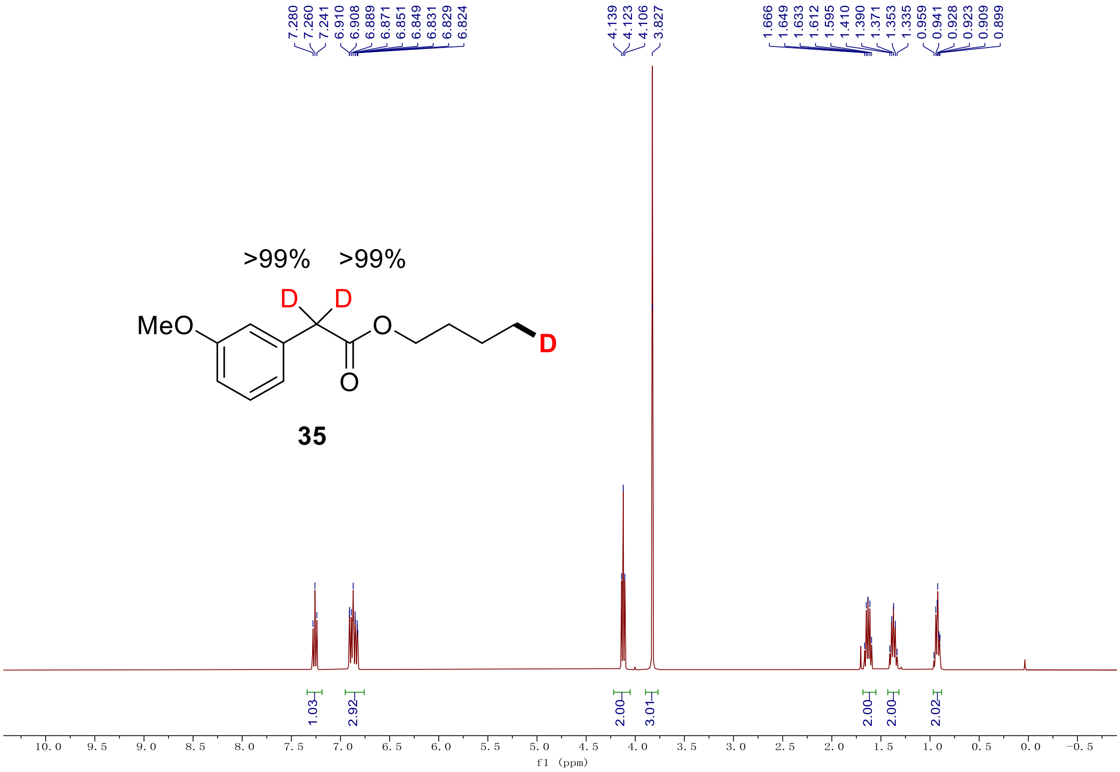

Supplementary Figure 82. ^{1}H NMR of compound **35** (400 MHz, Chloroform-*d*)

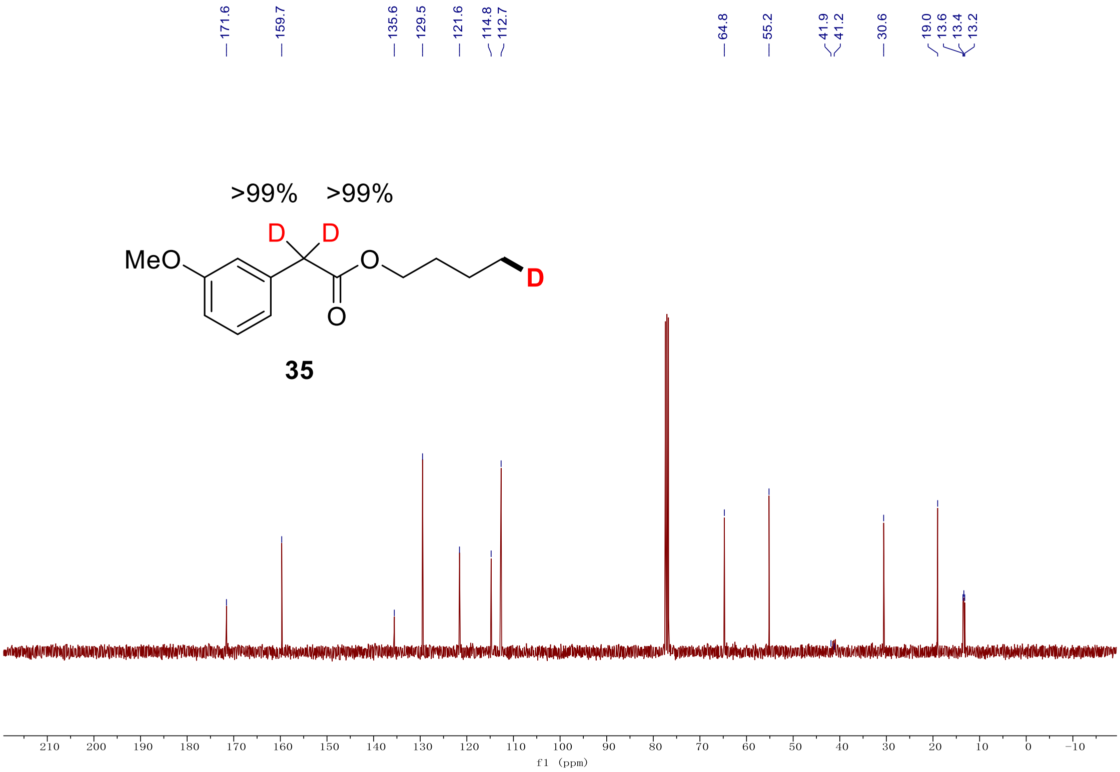

Supplementary Figure 83. ^{13}C NMR of compound **35** (100 MHz, Chloroform-*d*)

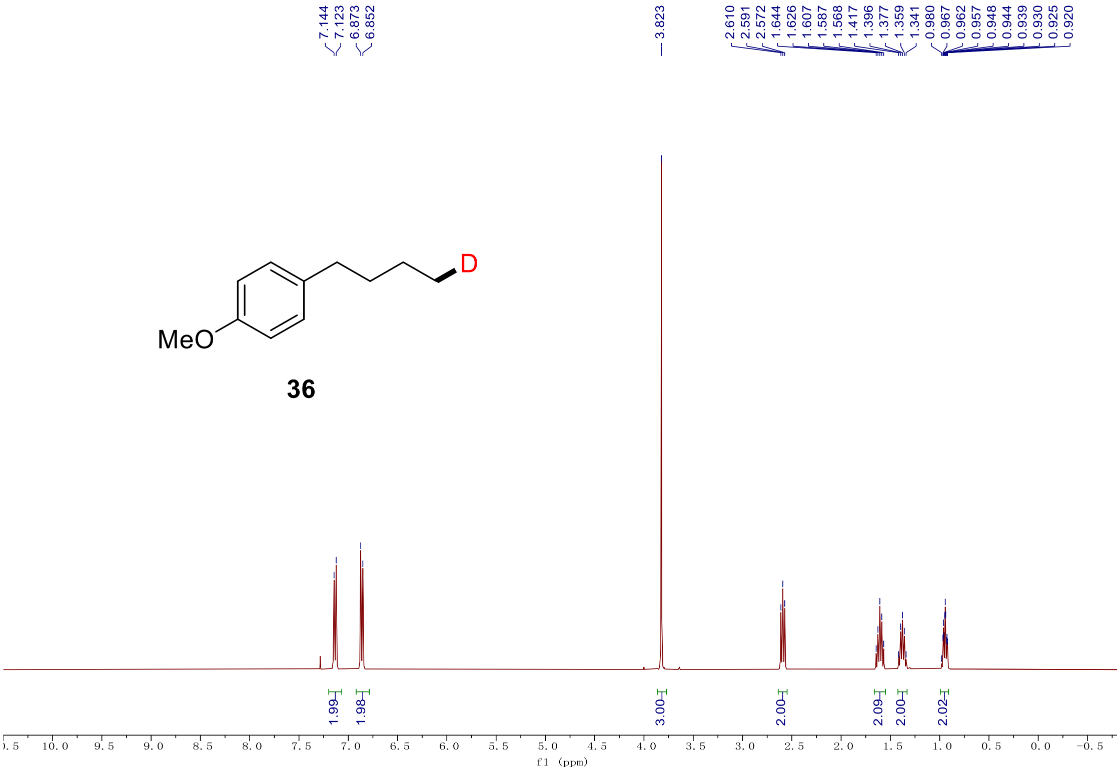

Supplementary Figure 84. ^{1}H NMR of compound **36** (400 MHz, Chloroform-*d*)

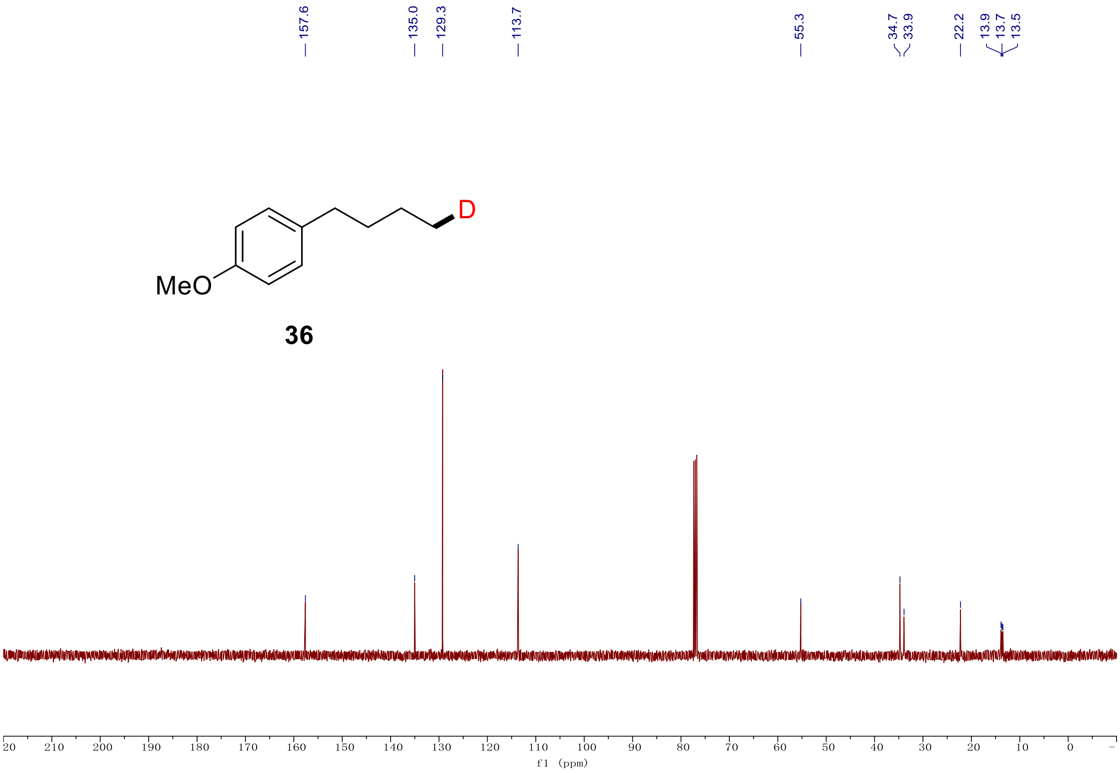

Supplementary Figure 85. ^{13}C NMR of compound **36** (100 MHz, Chloroform-*d*)

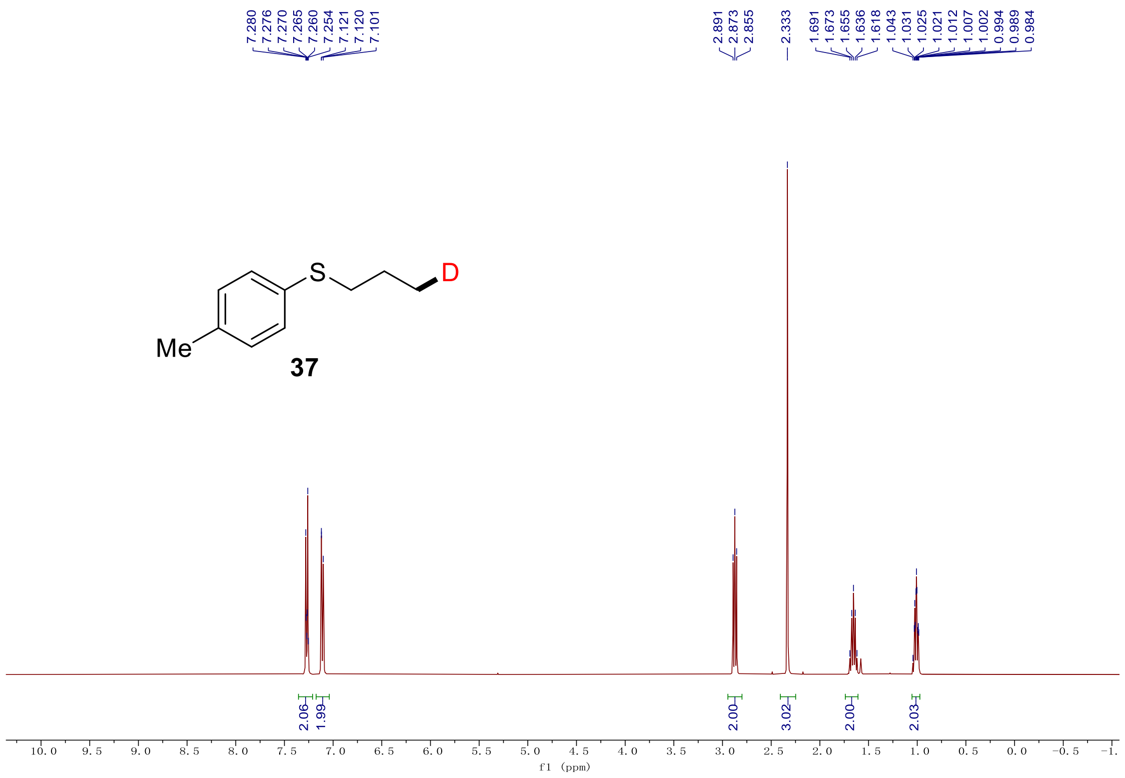

Supplementary Figure 86. ^{1}H NMR of compound **37** (400 MHz, Chloroform-*d*)

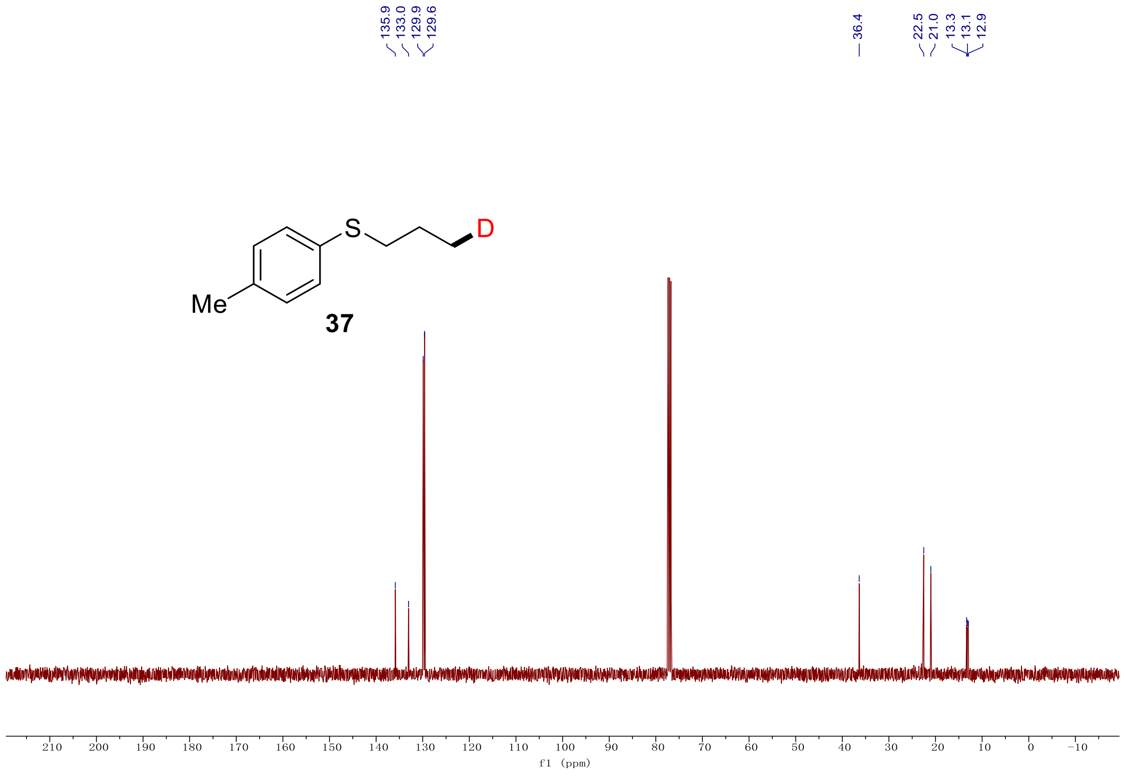

Supplementary Figure 87. ^{13}C NMR of compound **37** (100 MHz, Chloroform-*d*)

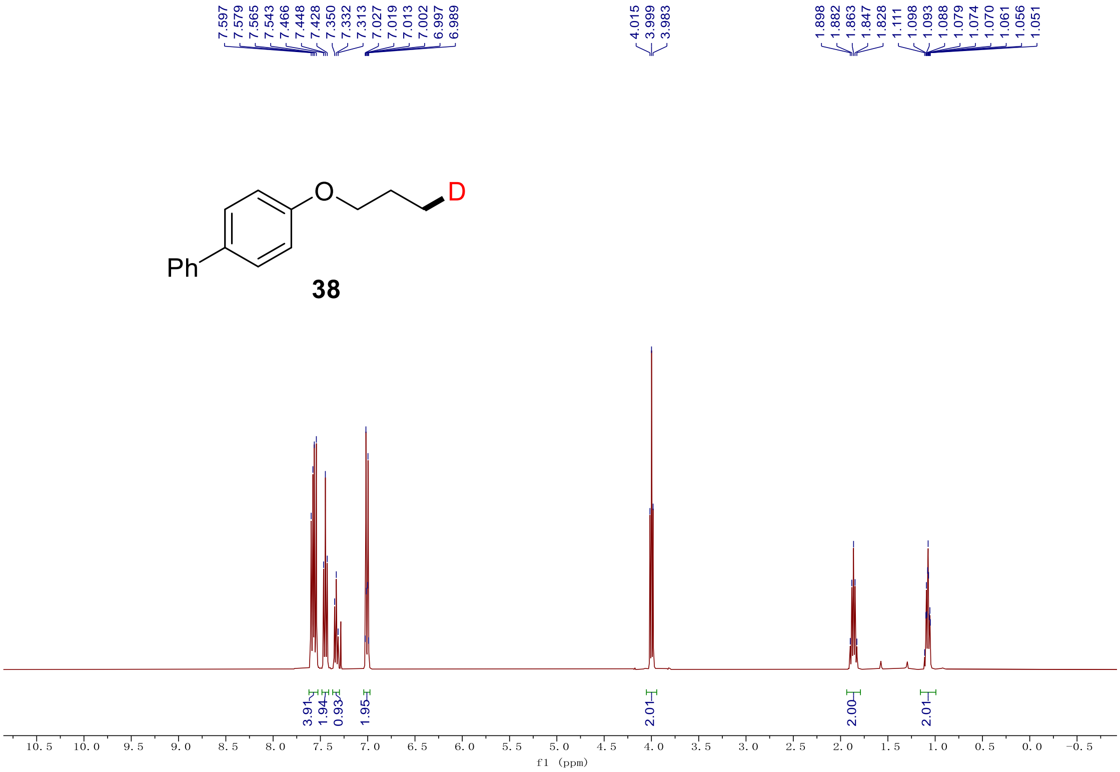

Supplementary Figure 88. ^{1}H NMR of compound **38** (400 MHz, Chloroform-*d*)

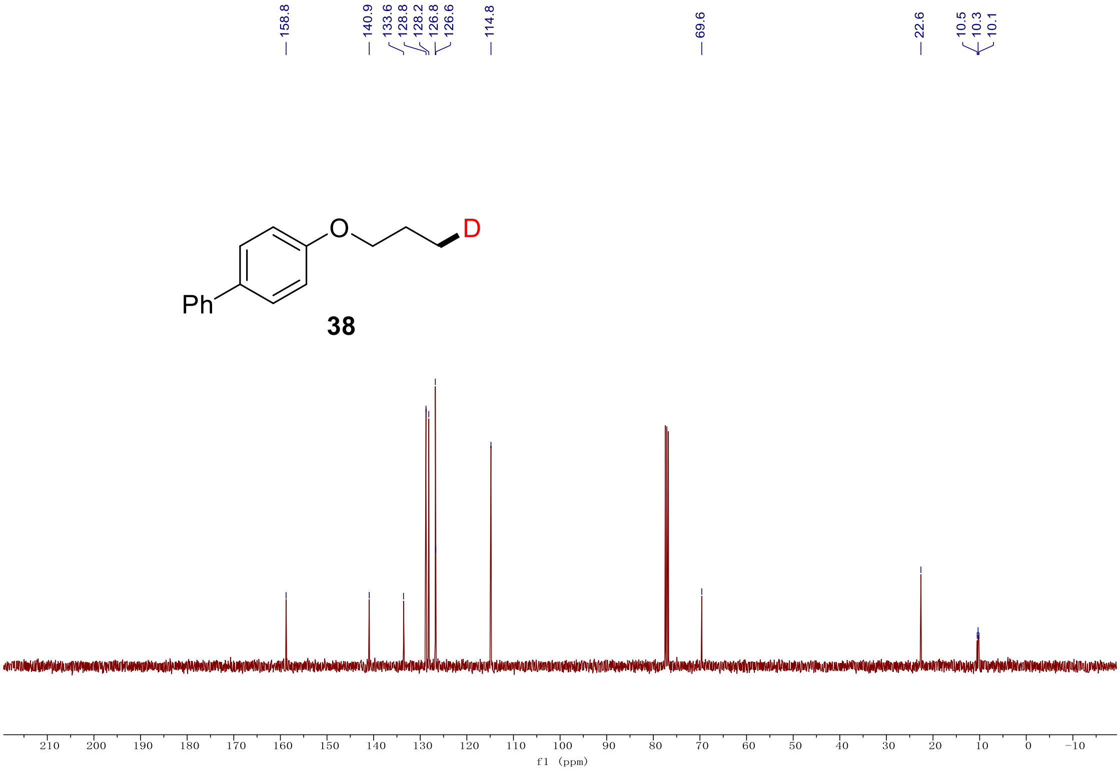

Supplementary Figure 89. ^{13}C NMR of compound **38** (100 MHz, Chloroform-*d*)

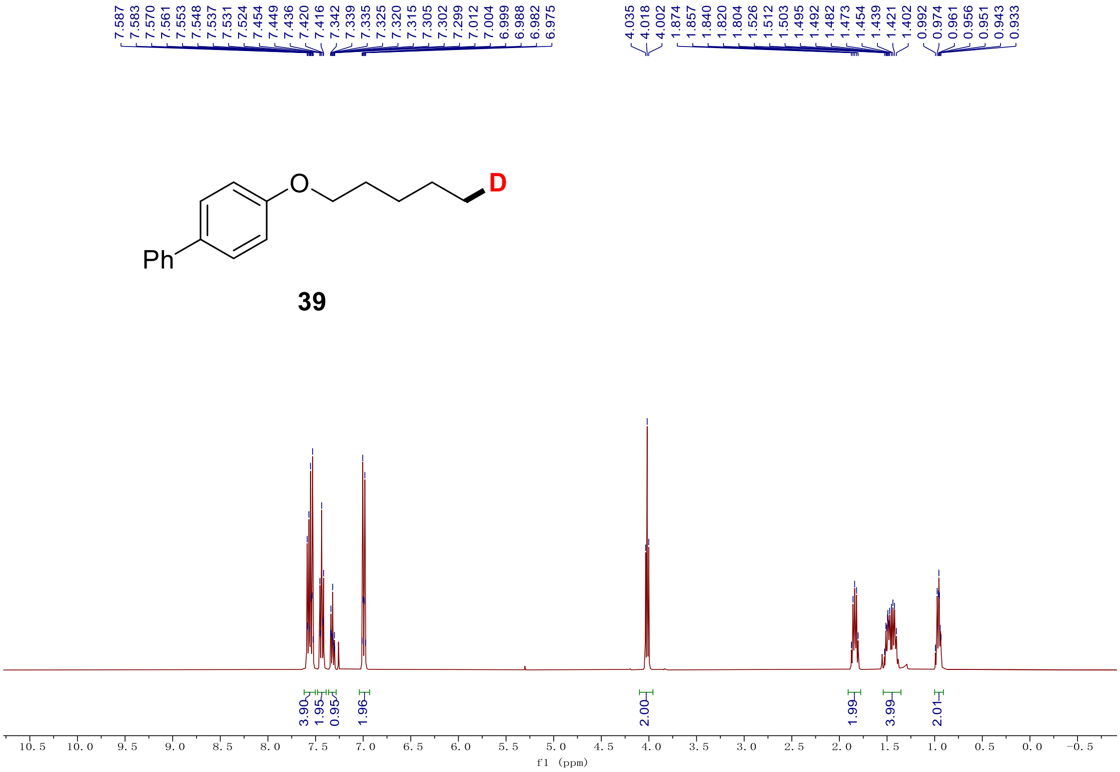

Supplementary Figure 90. ^{1}H NMR of compound **39** (400 MHz, Chloroform-*d*)

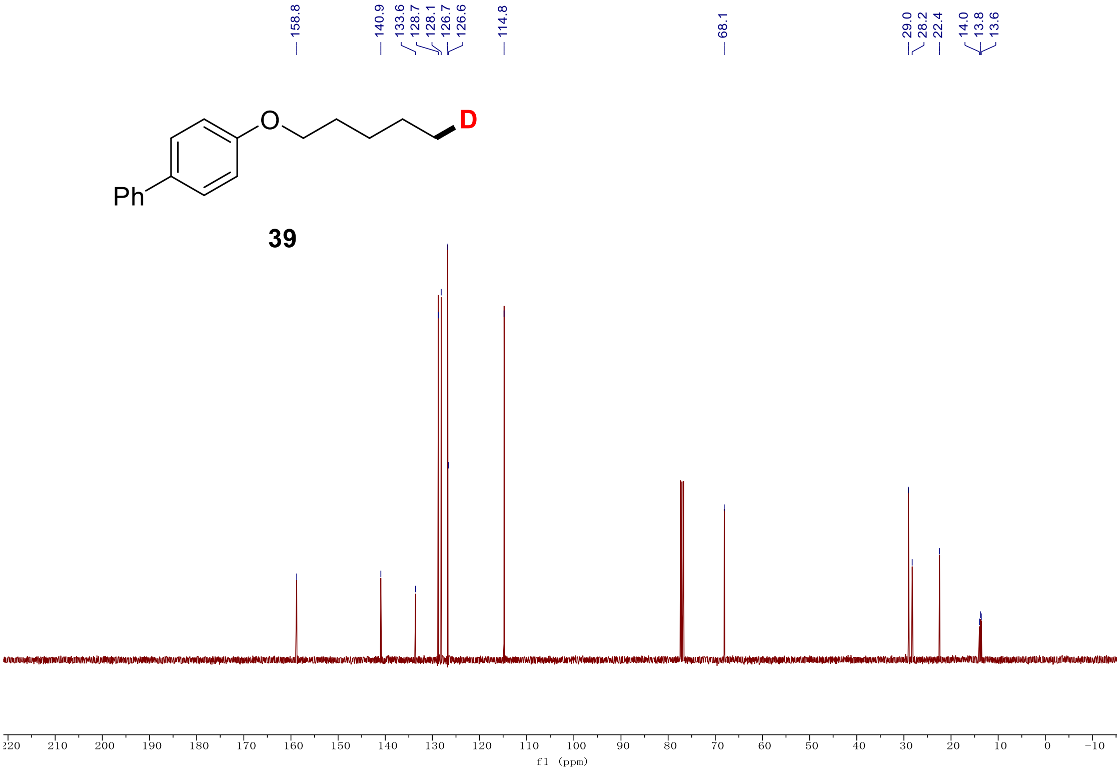

Supplementary Figure 91. ^{13}C NMR of compound **39** (100 MHz, Chloroform-*d*)

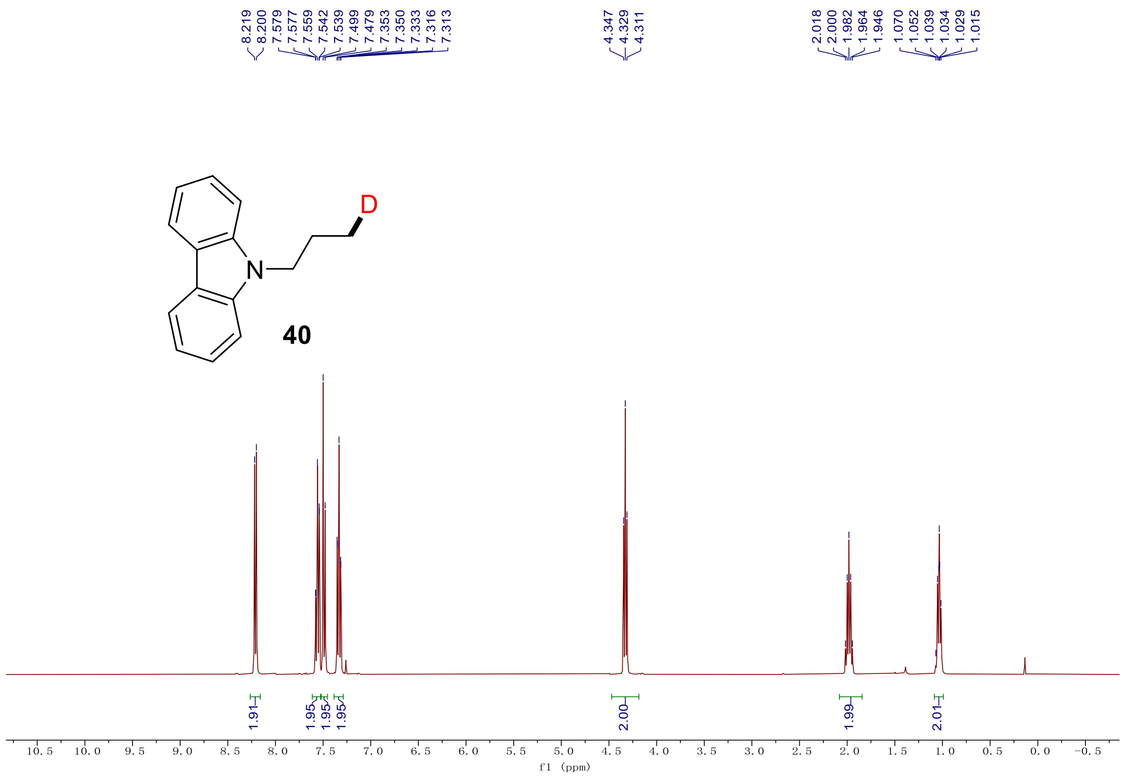

Supplementary Figure 92. ^{1}H NMR of compound **40** (400 MHz, Chloroform-*d*)

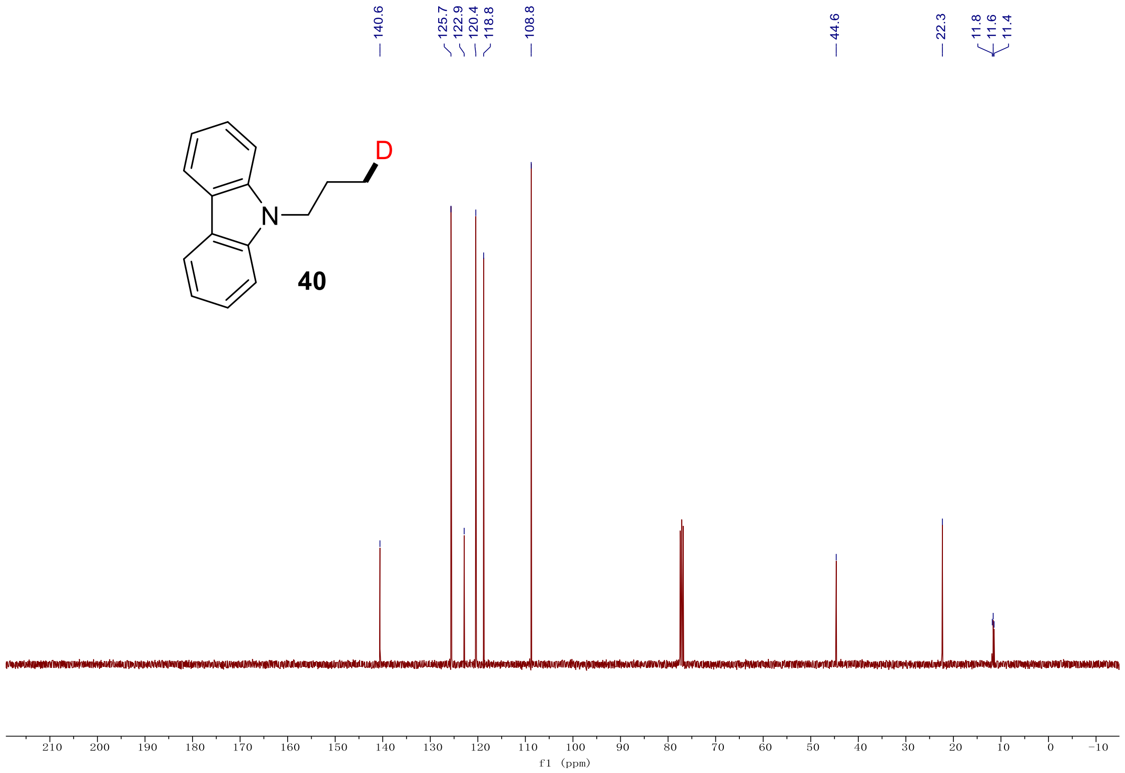

Supplementary Figure 93. ^{13}C NMR of compound **40** (100 MHz, Chloroform-*d*)

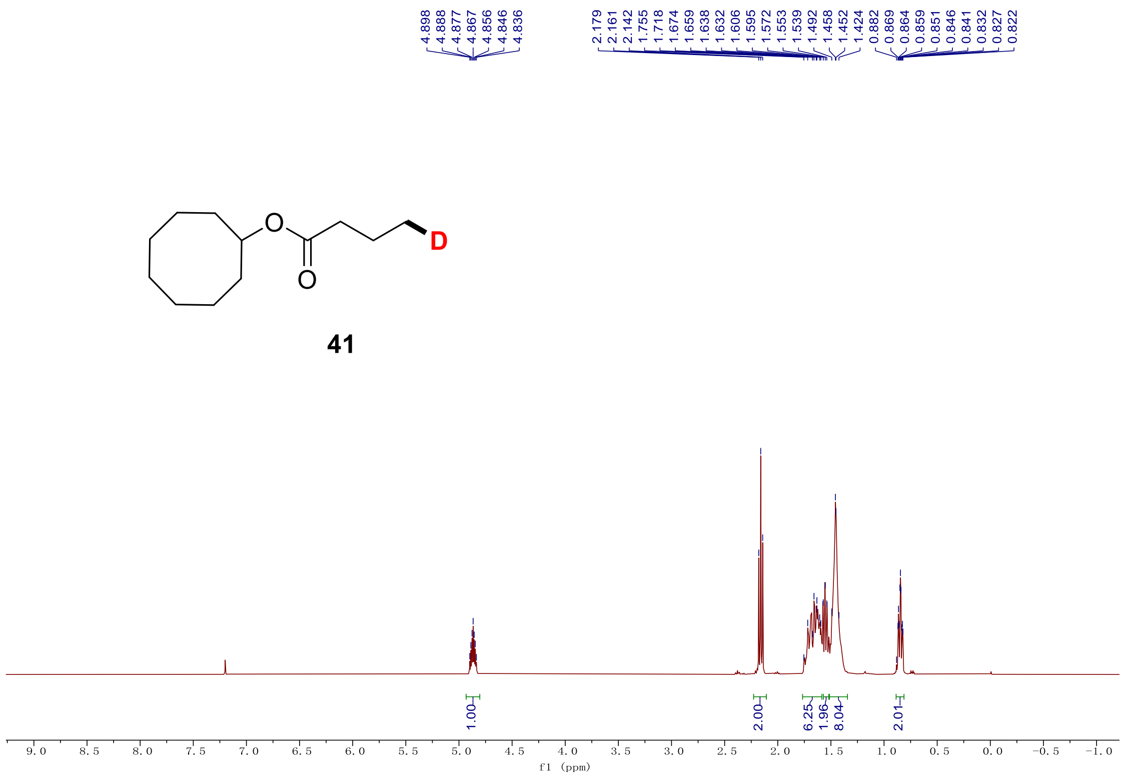

Supplementary Figure 94. ^{1}H NMR of compound **41** (400 MHz, Chloroform-*d*)

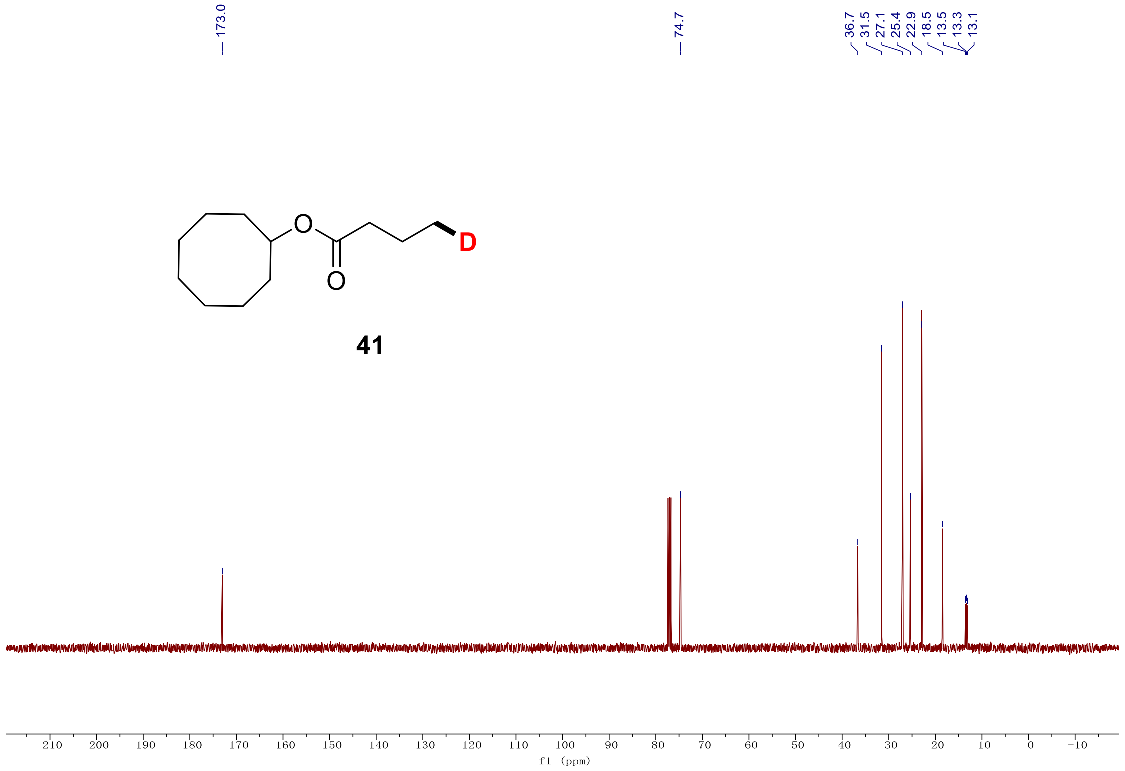

Supplementary Figure 95. ^{13}C NMR of compound **41** (100 MHz, Chloroform-*d*)

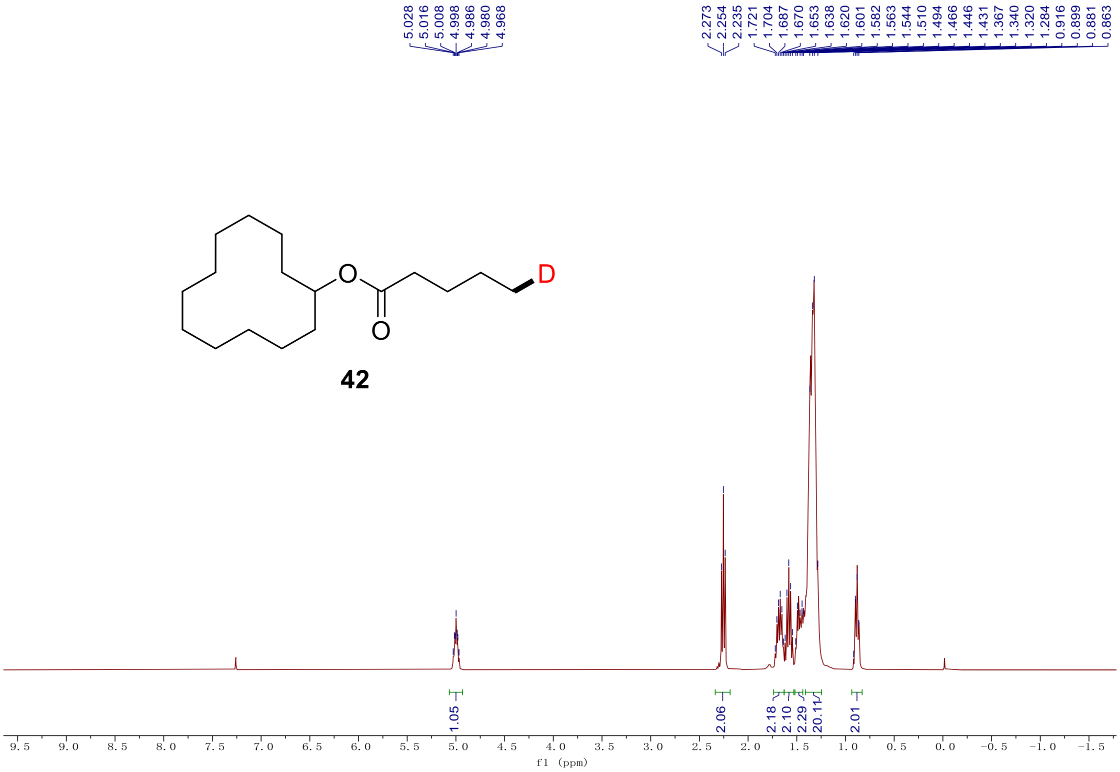

Supplementary Figure 96. ^{1}H NMR of compound **42** (400 MHz, Chloroform-*d*)

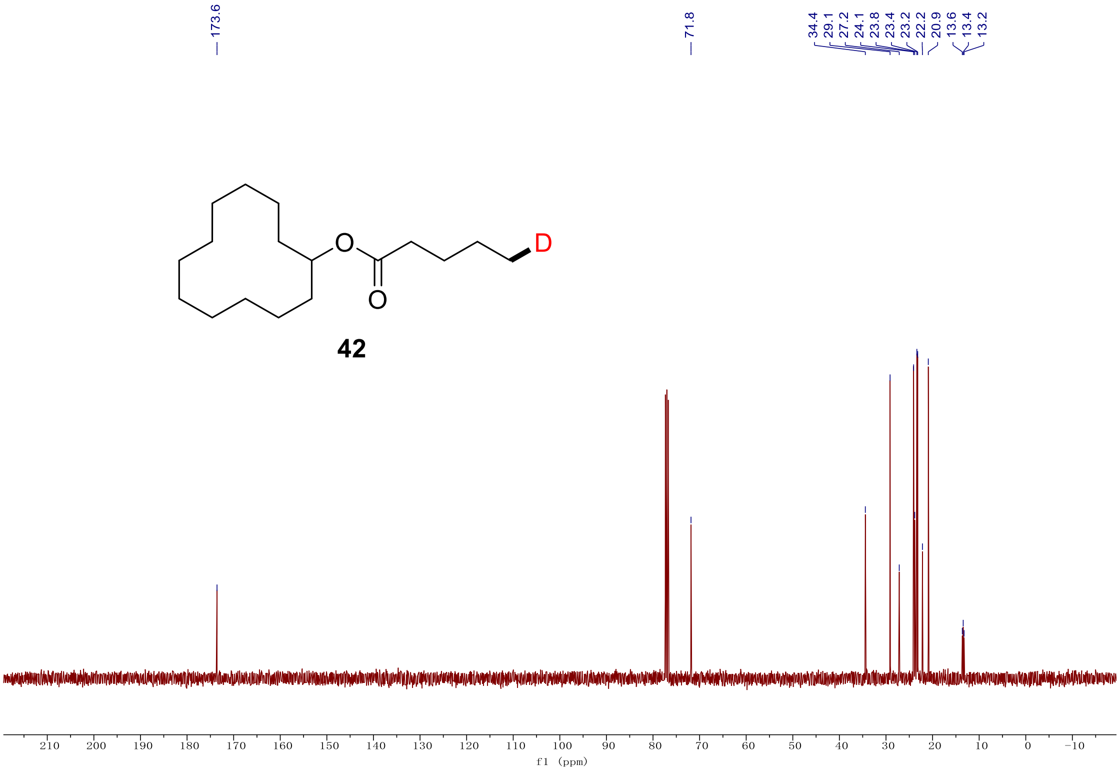

Supplementary Figure 97. ^{13}C NMR of compound **42** (100 MHz, Chloroform-*d*)

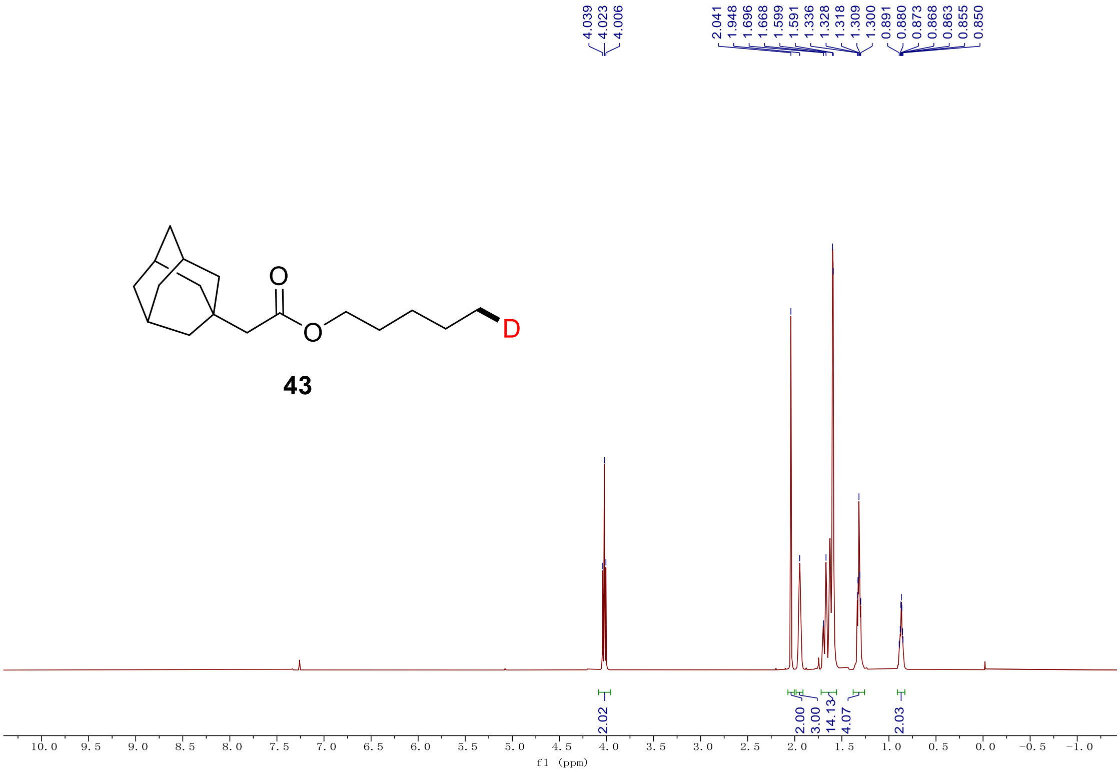

Supplementary Figure 98. ^{1}H NMR of compound **43** (400 MHz, Chloroform-*d*)

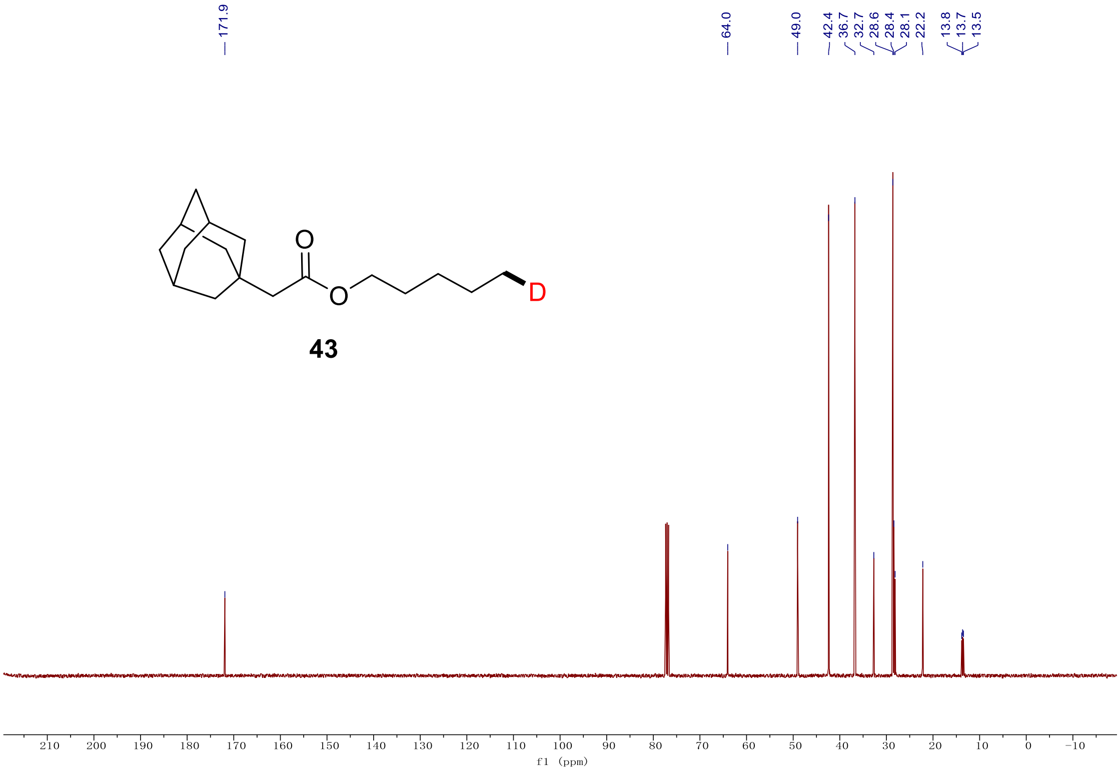

Supplementary Figure 99. ^{13}C NMR of compound **43** (100 MHz, Chloroform-*d*)

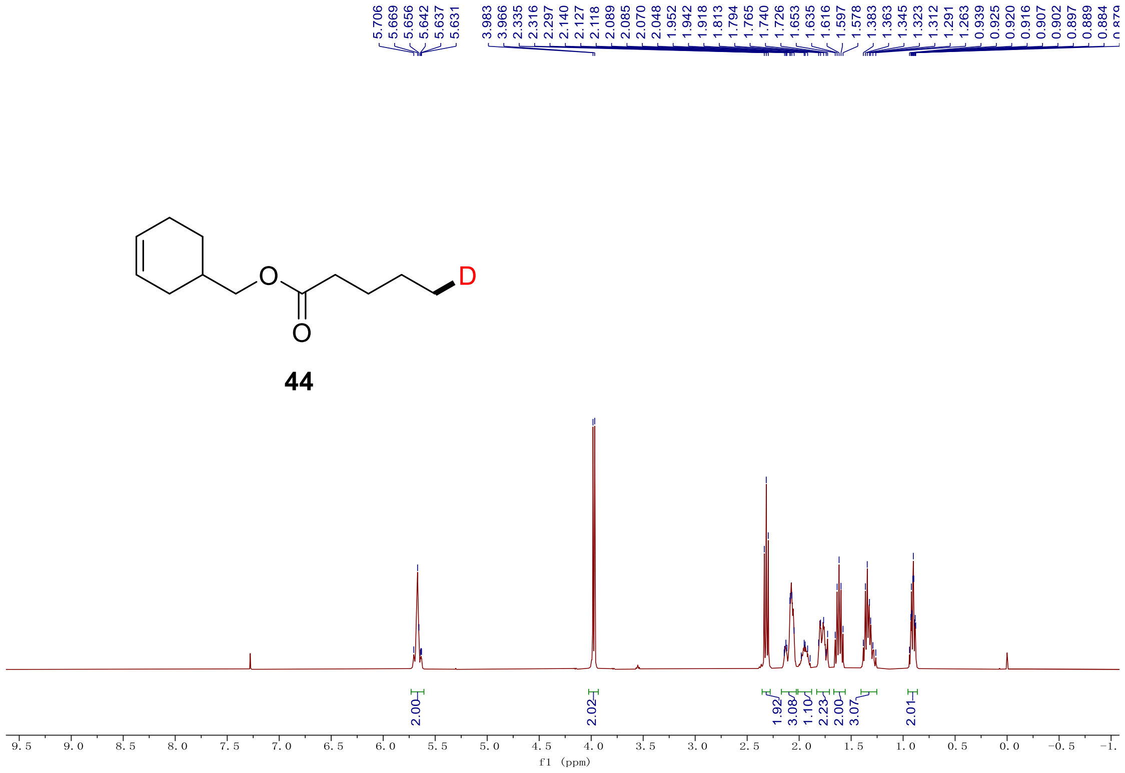

Supplementary Figure 100. ^{1}H NMR of compound **44** (400 MHz, Chloroform-*d*)

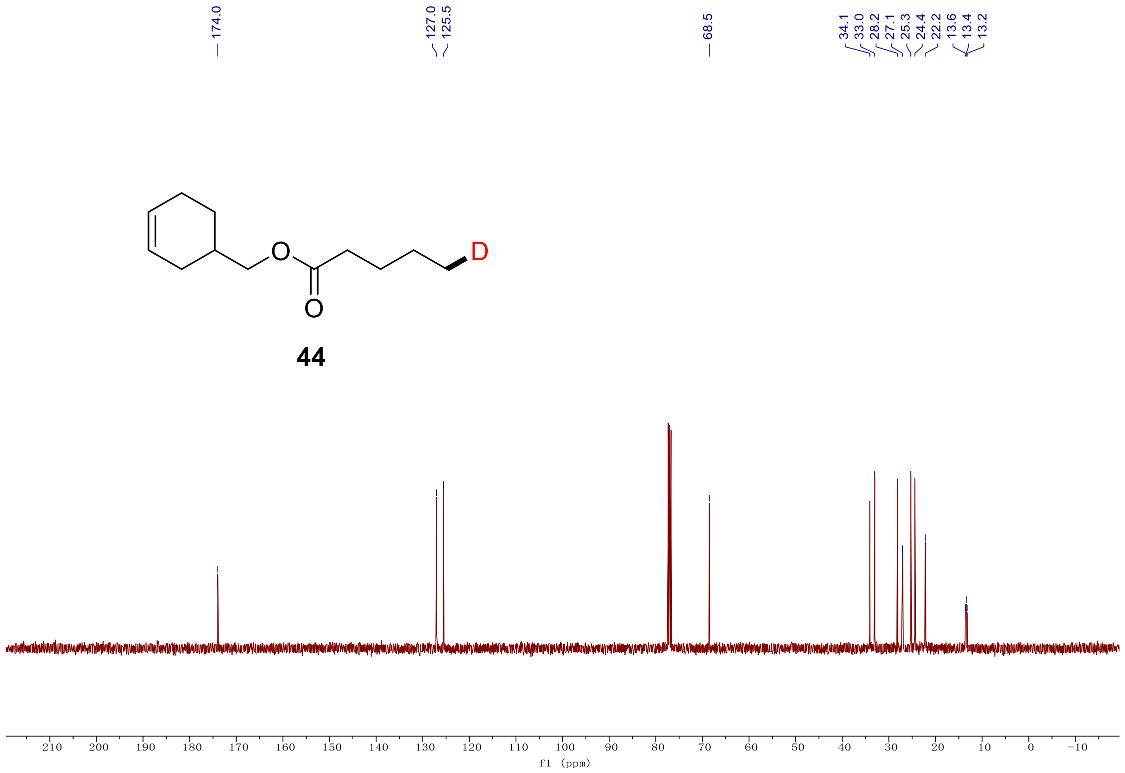

Supplementary Figure 101. ^{13}C NMR of compound **44** (100 MHz, Chloroform-*d*)

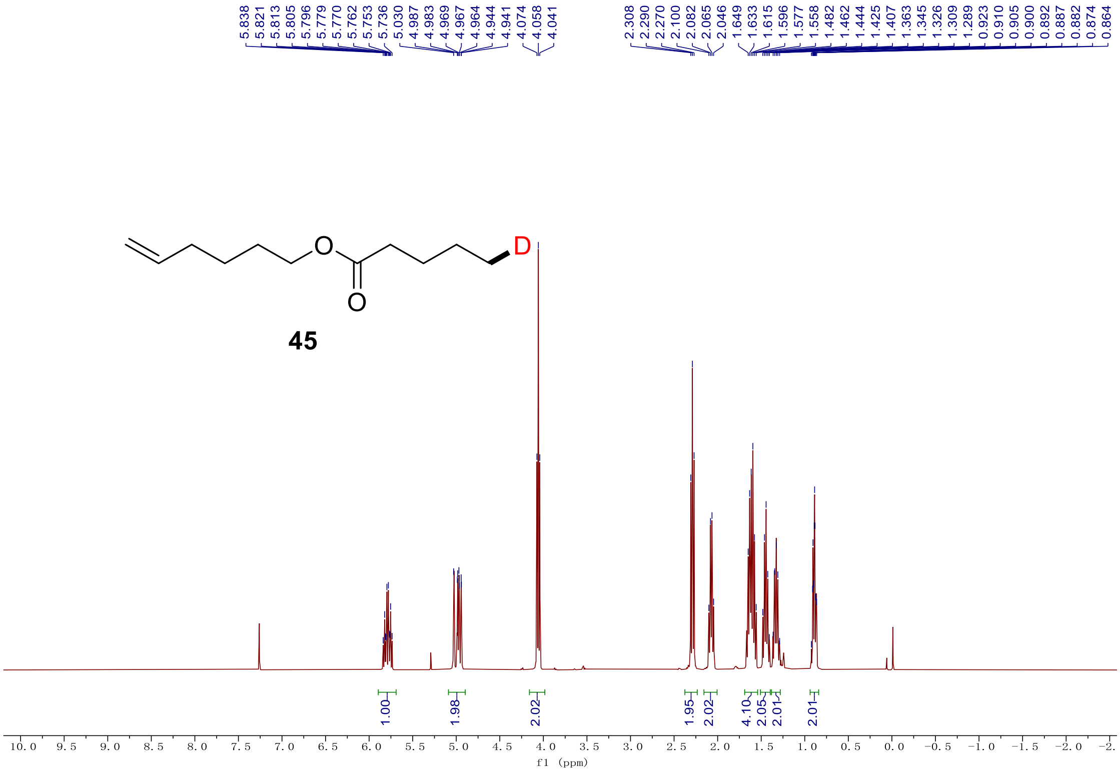

Supplementary Figure 102. ^{1}H NMR of compound **45** (400 MHz, Chloroform-*d*)

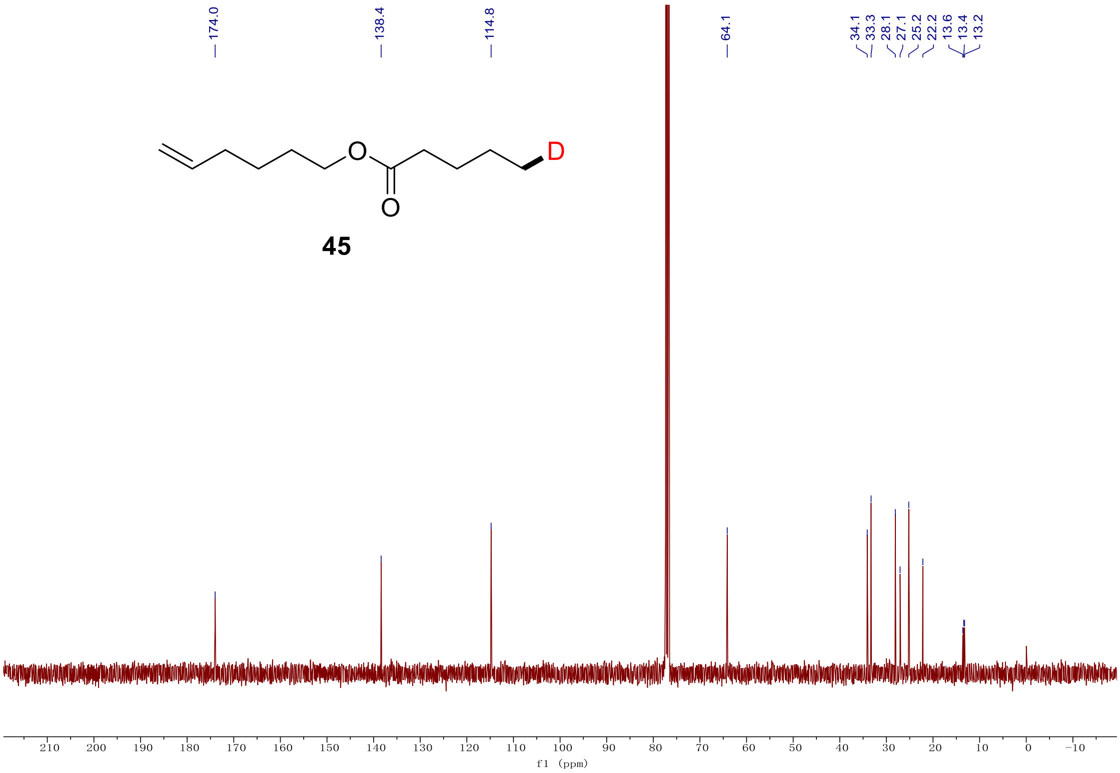

Supplementary Figure 103. ^{13}C NMR of compound **45** (100 MHz, Chloroform-*d*)

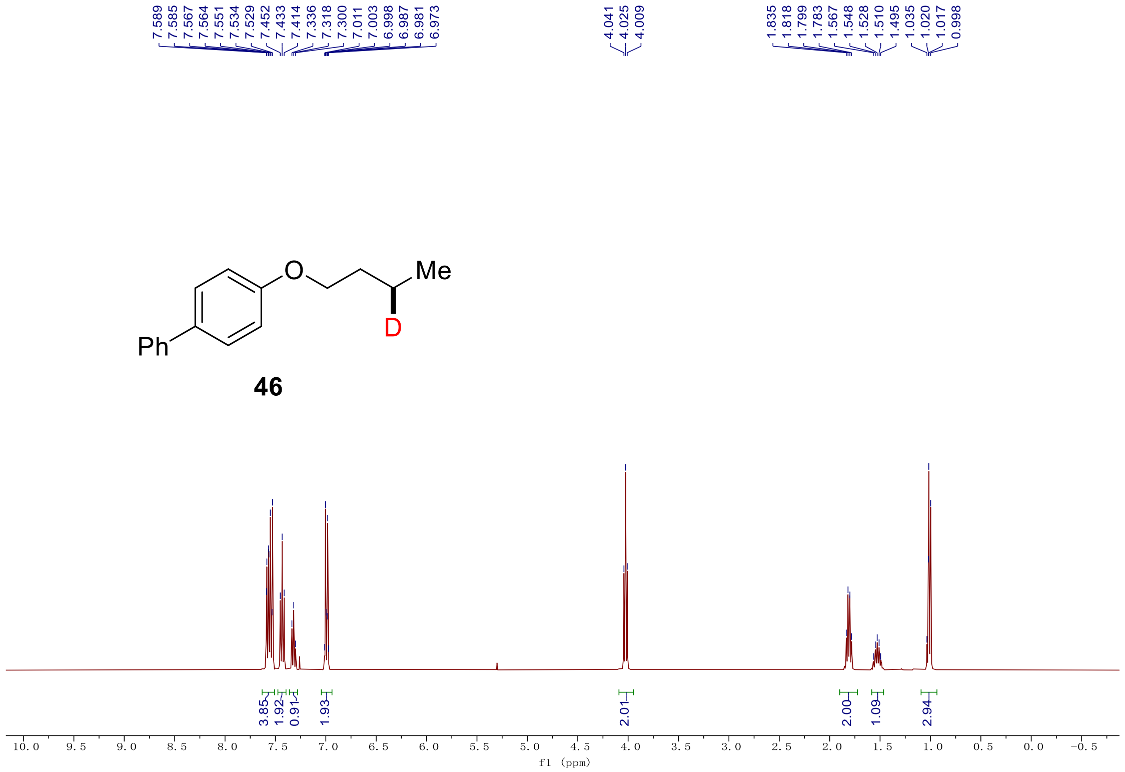

Supplementary Figure 104. ^{1}H NMR of compound **46** (400 MHz, Chloroform-*d*)

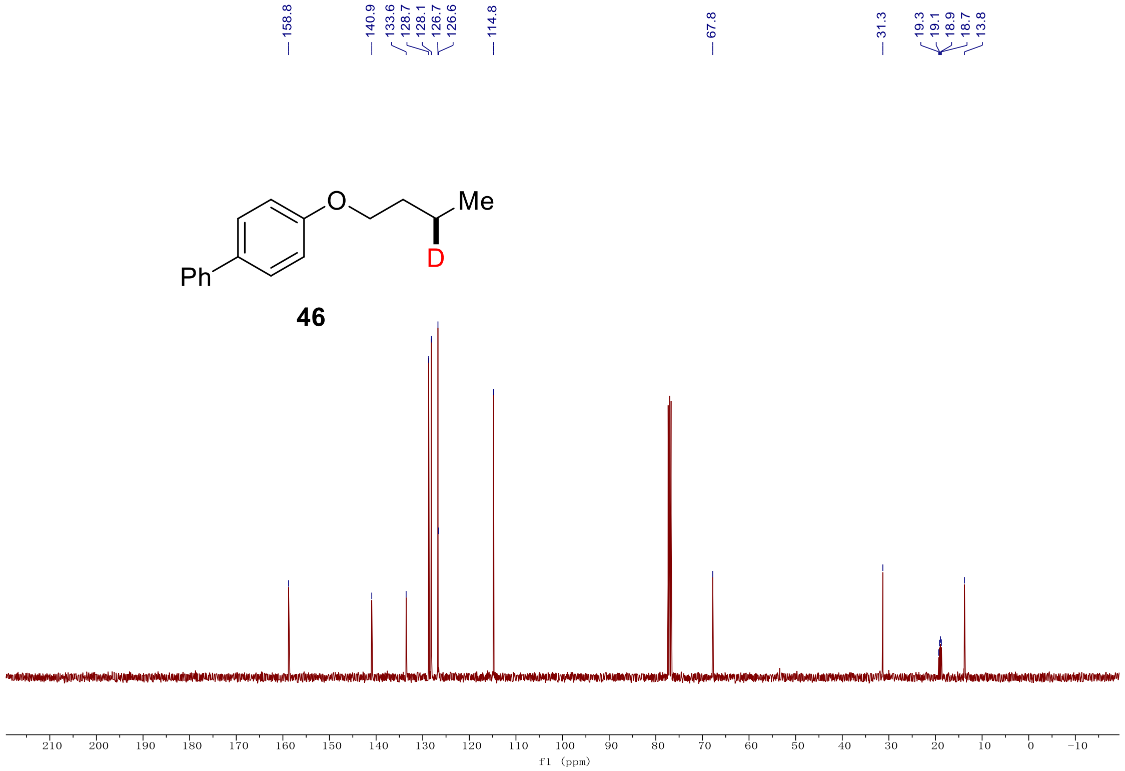

Supplementary Figure 105. ^{13}C NMR of compound **46** (100 MHz, Chloroform-*d*)

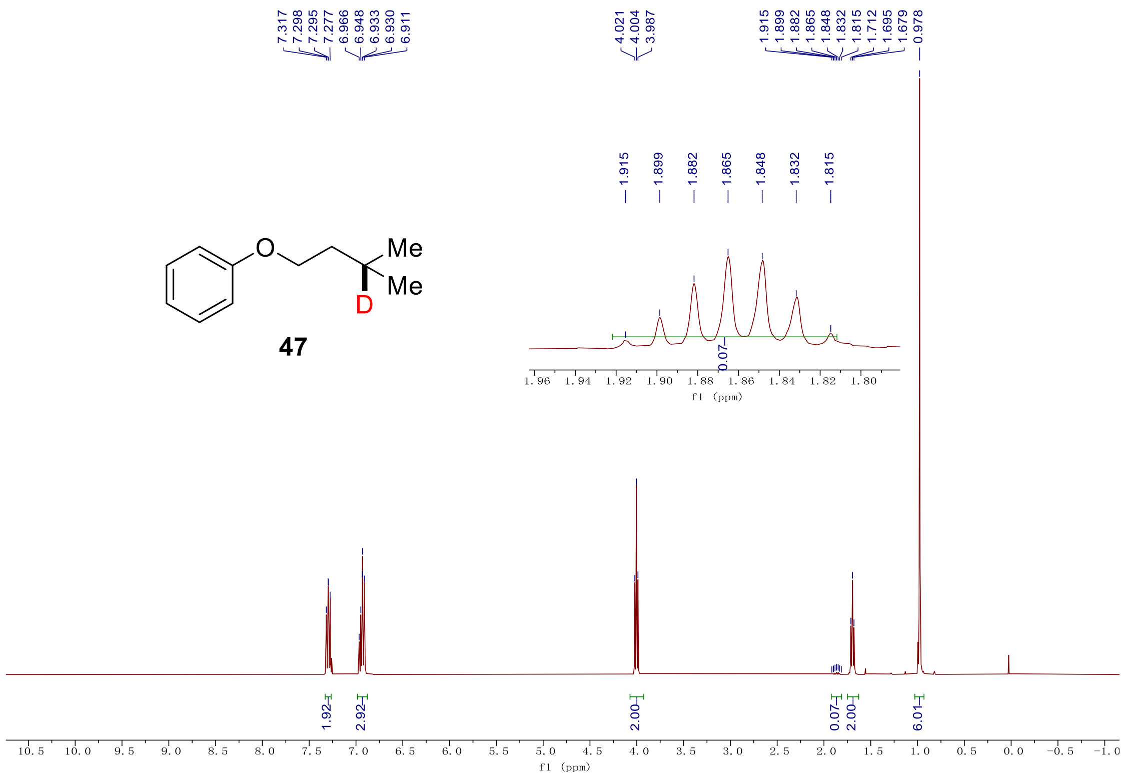

Supplementary Figure 106. ^{1}H NMR of compound **47** (400 MHz, Chloroform-*d*)

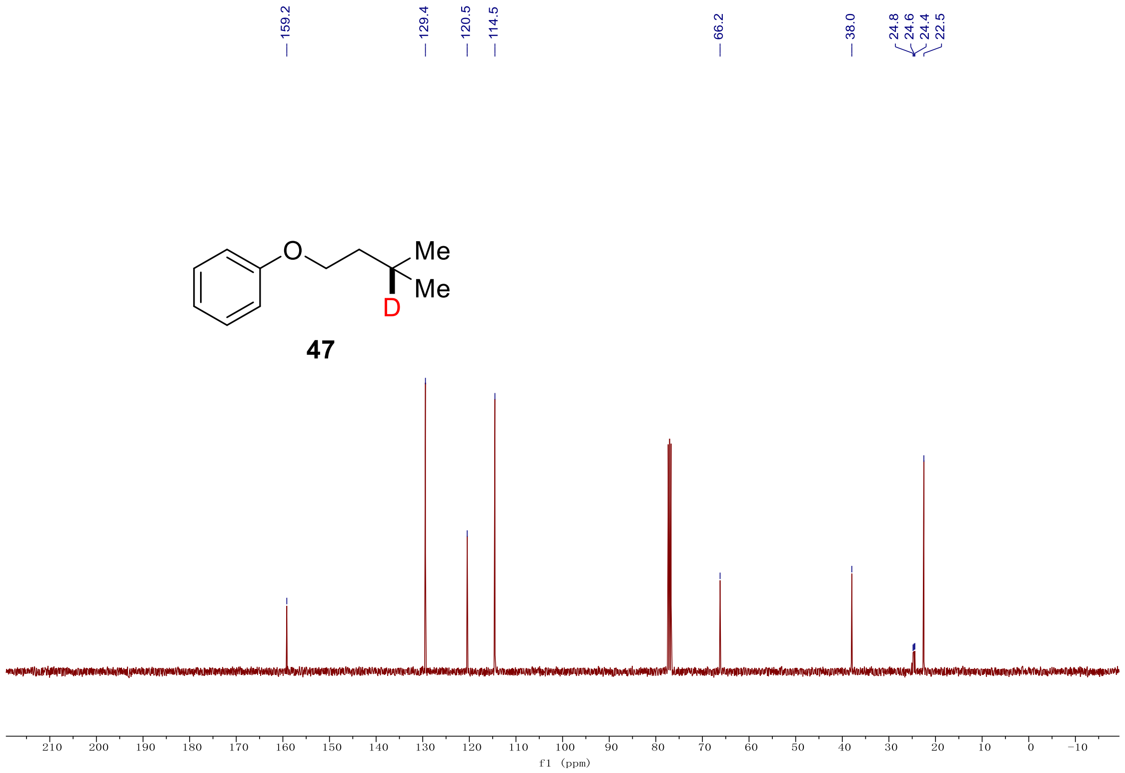

Supplementary Figure 107. ^{13}C NMR of compound **47** (100 MHz, Chloroform-*d*)

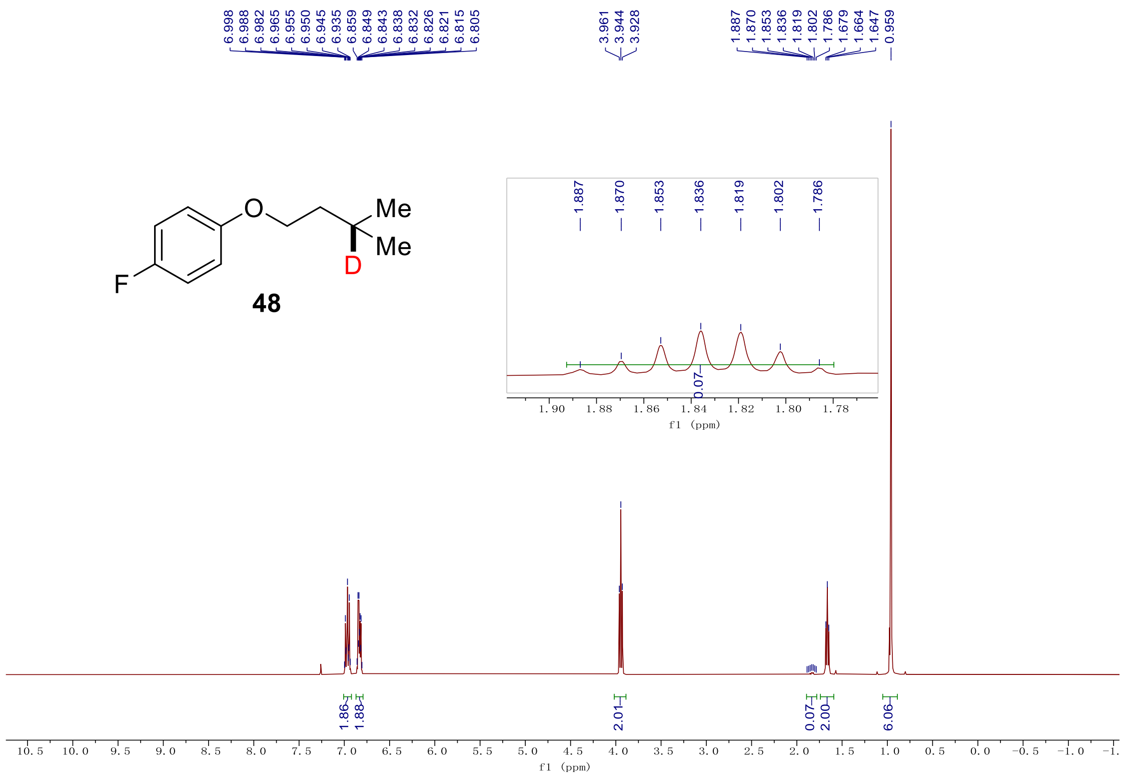

Supplementary Figure 108. ^{1}H NMR of compound **48** (400 MHz, Chloroform-*d*)

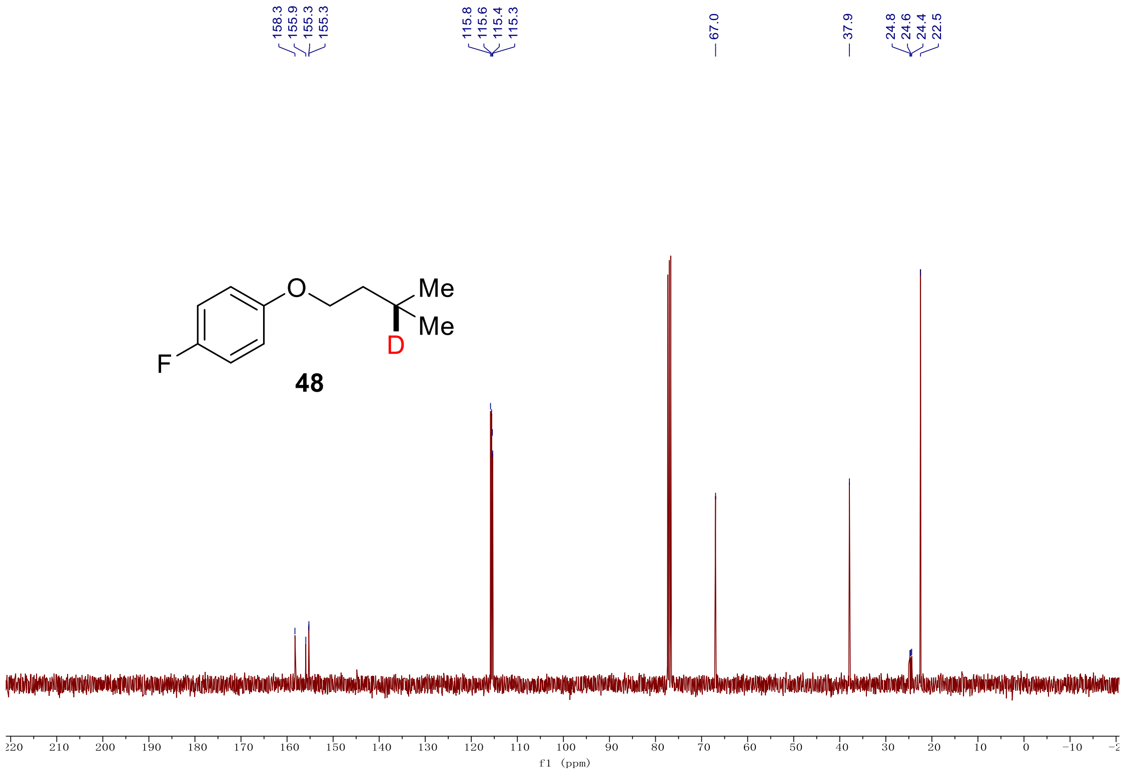

Supplementary Figure 109. ^{13}C NMR of compound **48** (100 MHz, Chloroform-*d*)

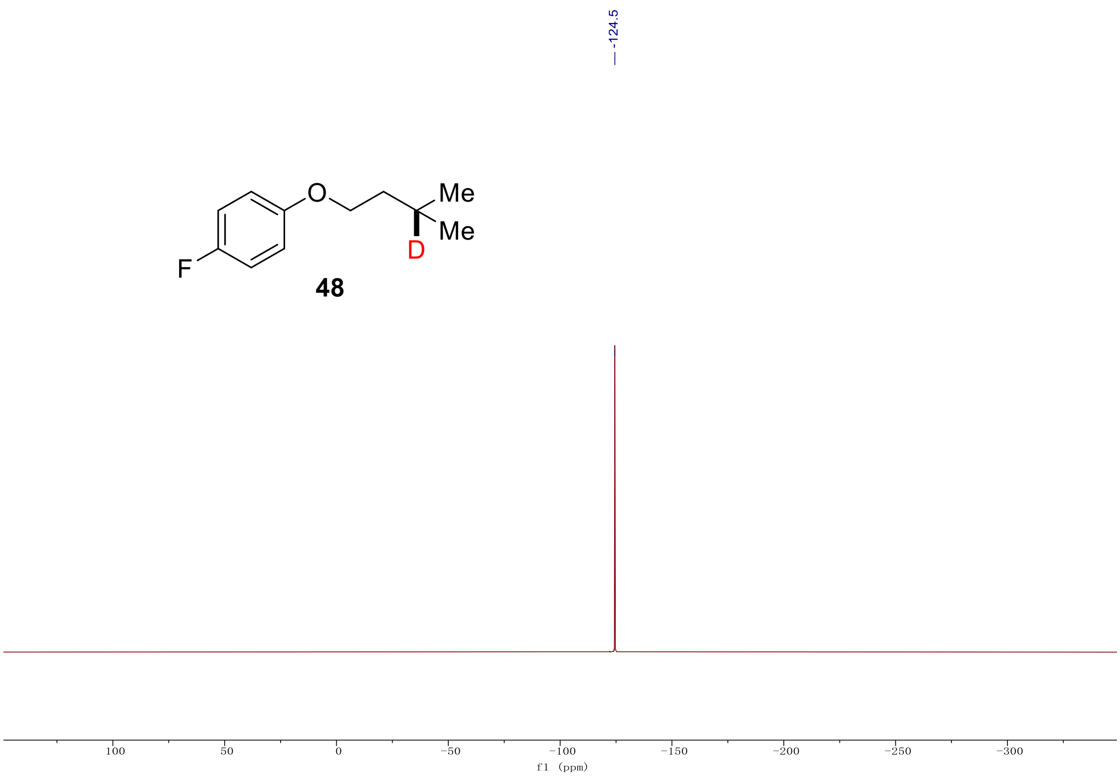

Supplementary Figure 110. ^{19}F NMR of compound **48** (375 MHz, Chloroform-*d*)

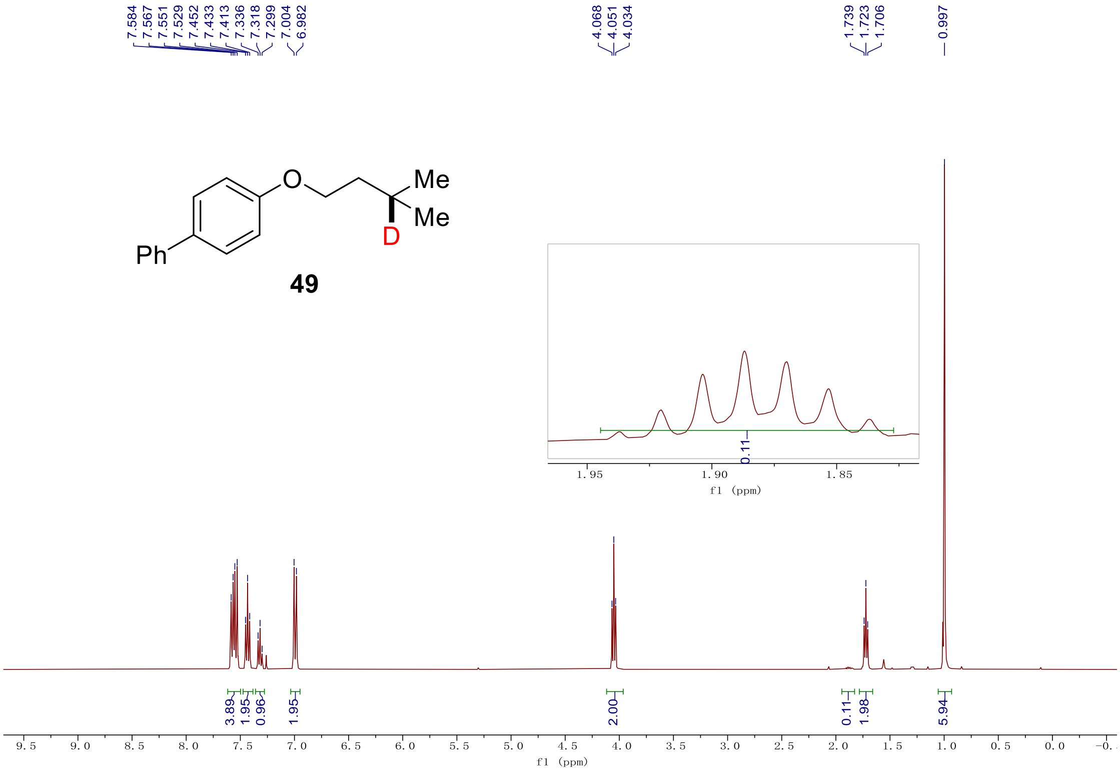

Supplementary Figure 111. ^{1}H NMR of compound **49** (400 MHz, Chloroform-*d*)

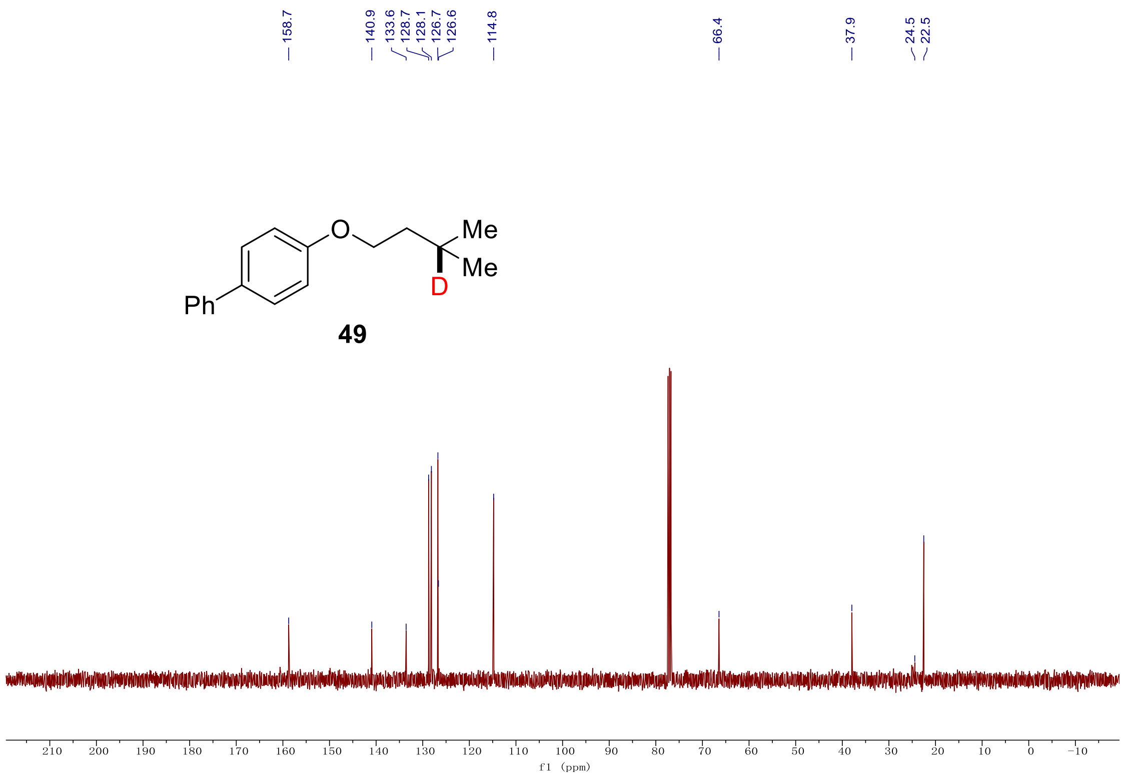

Supplementary Figure 112. ^{13}C NMR of compound **49** (100 MHz, Chloroform-*d*)

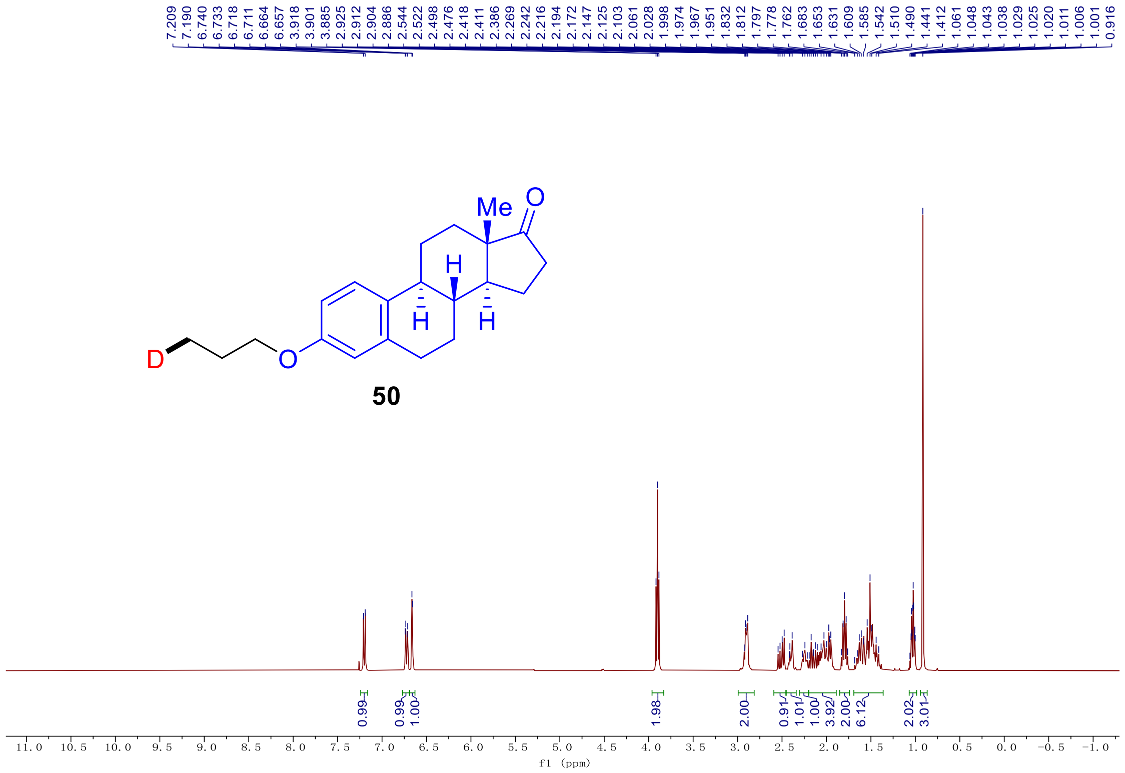

Supplementary Figure 113. ^{1}H NMR of compound **50** (400 MHz, Chloroform-*d*)

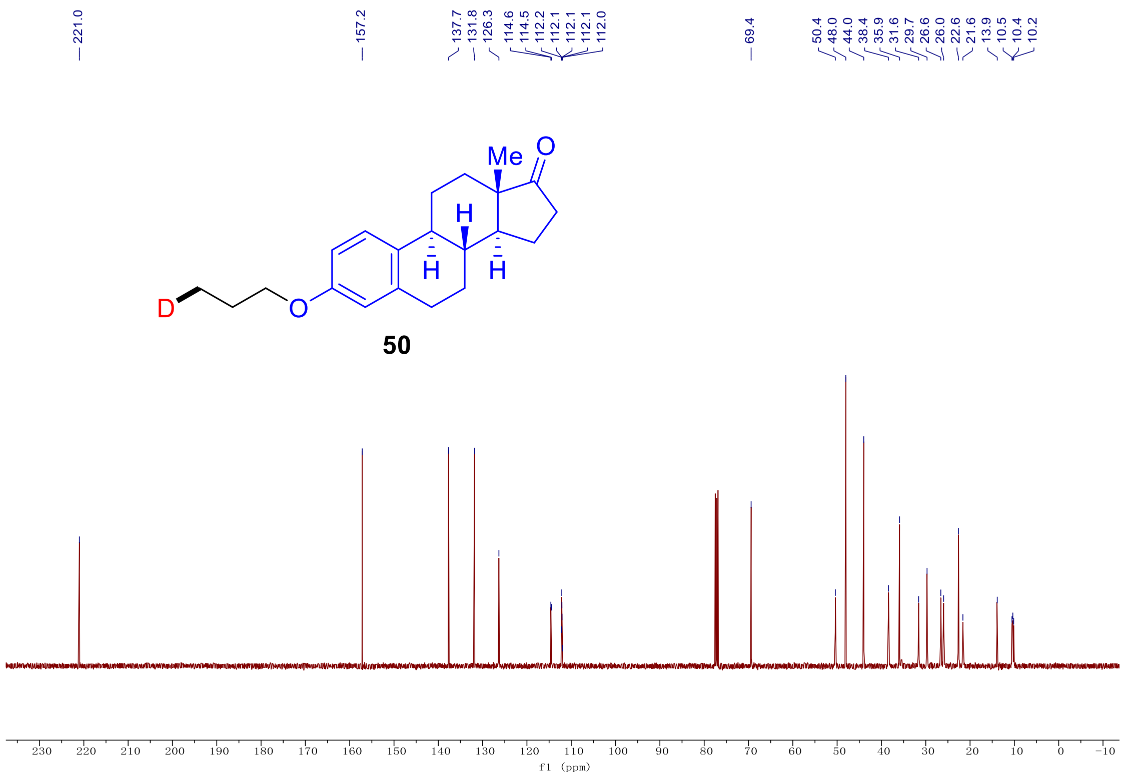

Supplementary Figure 114. ^{13}C NMR of compound **50** (100 MHz, Chloroform-*d*)

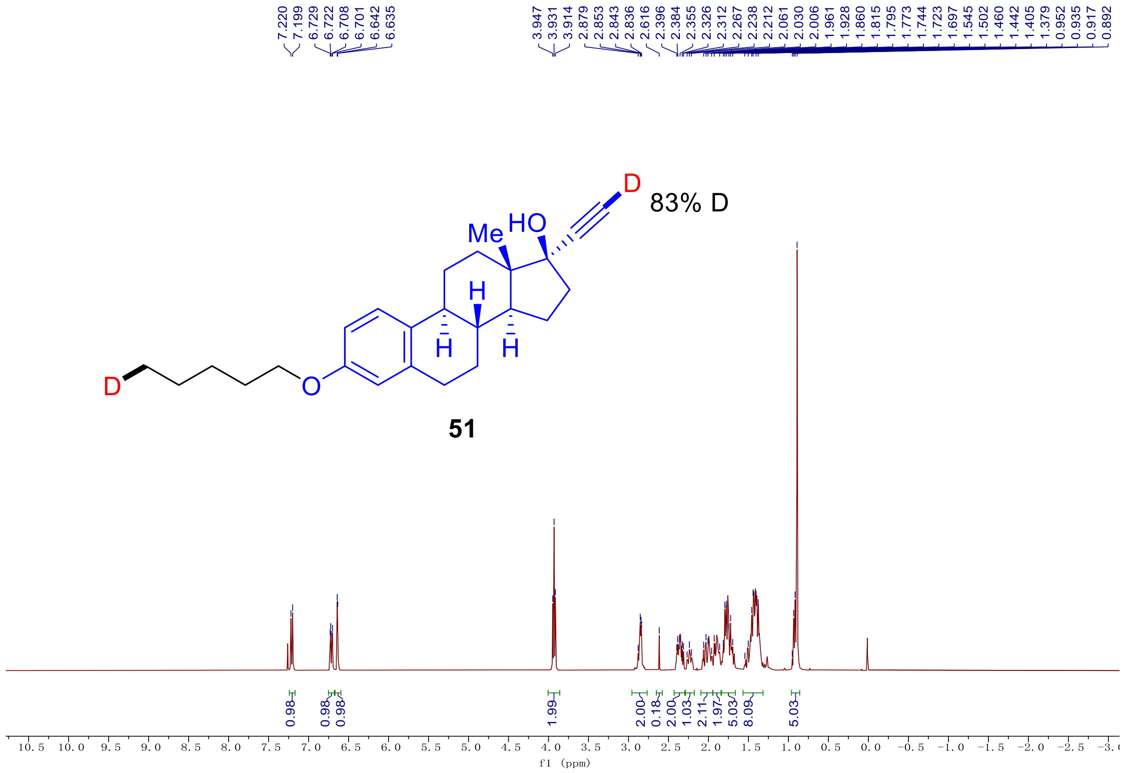

Supplementary Figure 115. ^{1}H NMR of compound **51** (400 MHz, Chloroform-*d*)

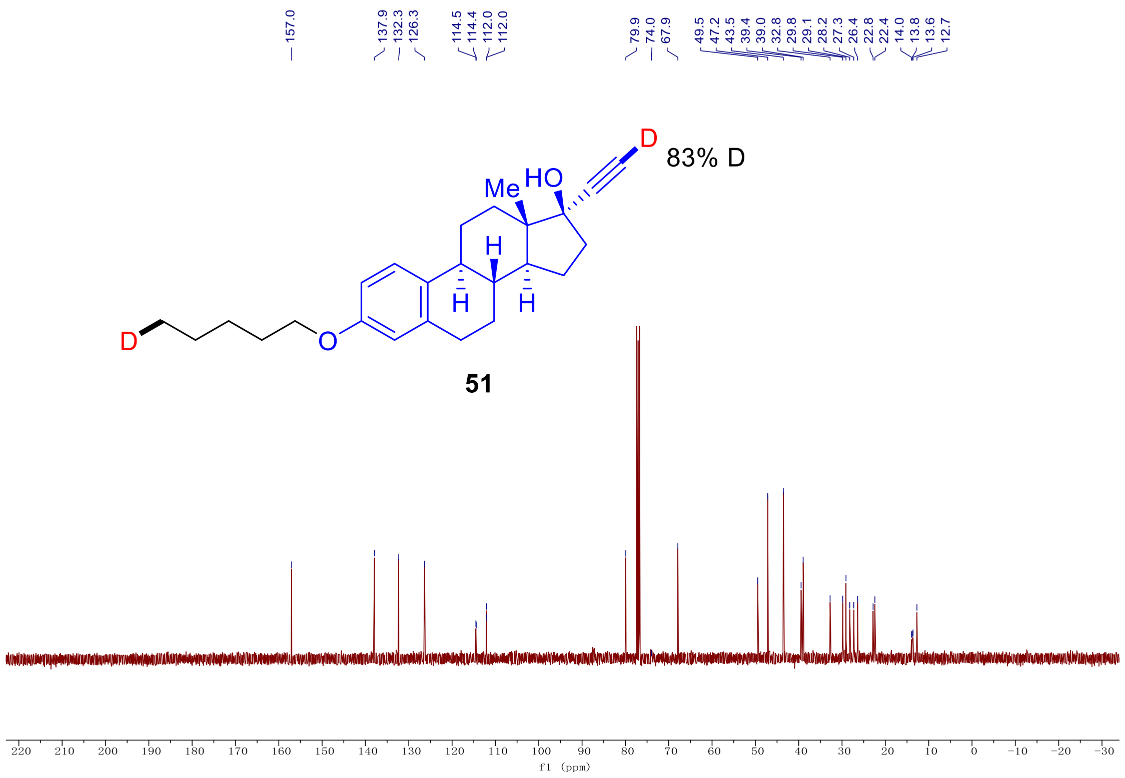

Supplementary Figure 116. ^{13}C NMR of compound **51** (100 MHz, Chloroform-*d*)

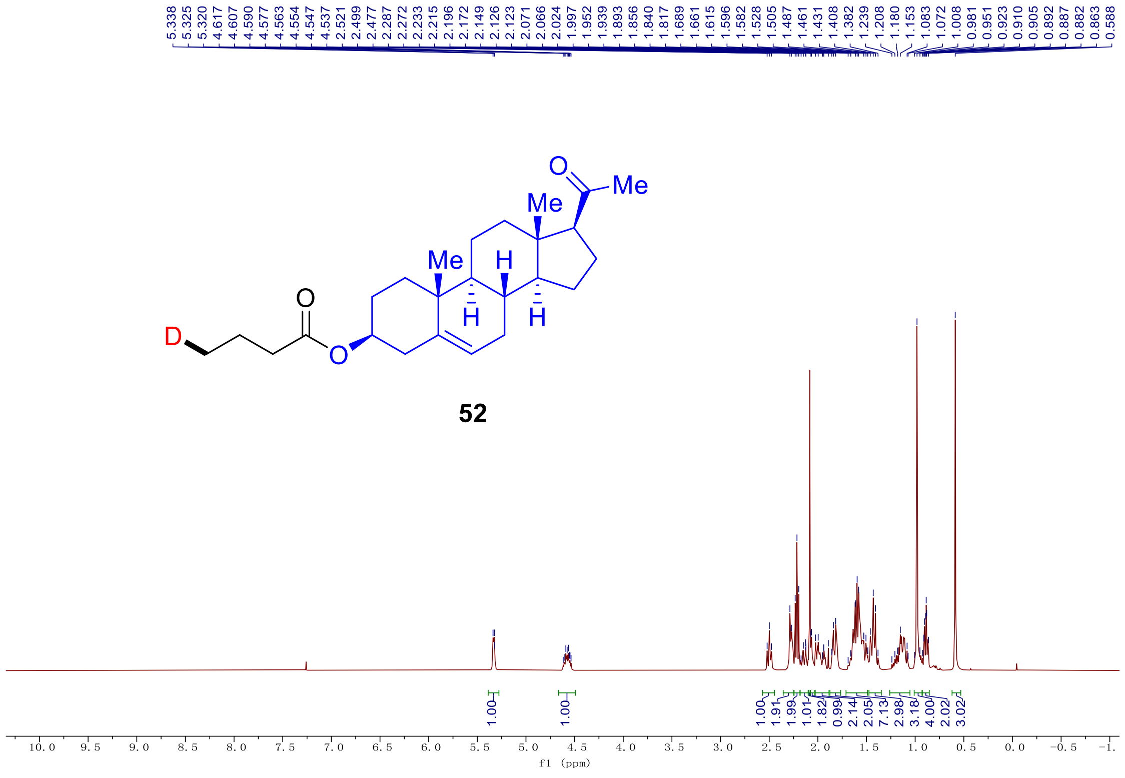

Supplementary Figure 117. ^{1}H NMR of compound **52** (400 MHz, Chloroform-*d*)

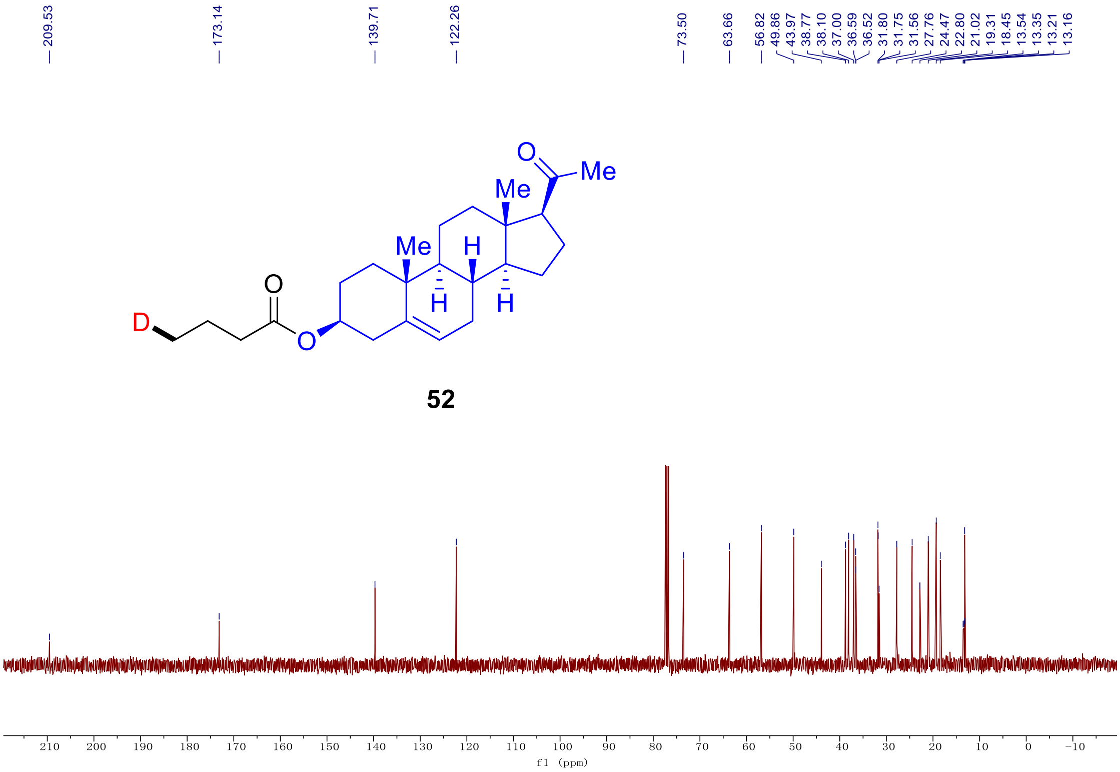

Supplementary Figure 118. ^{13}C NMR of compound 5**2** (100 MHz, Chloroform-*d*)

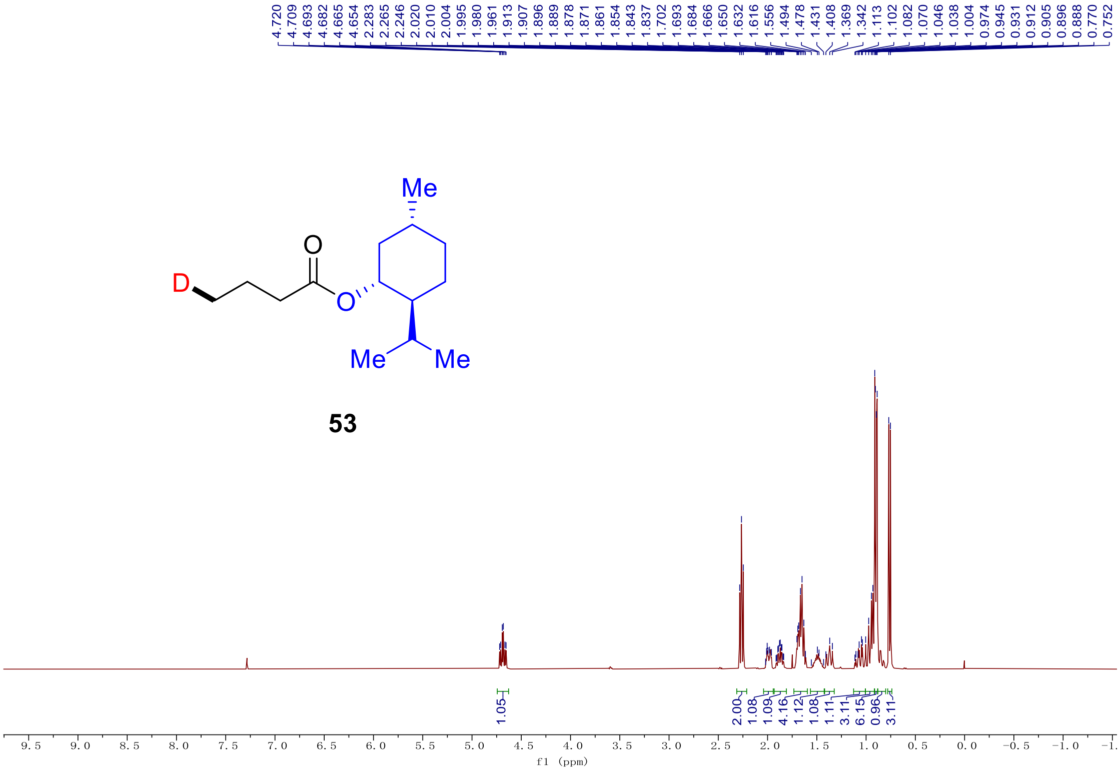

Supplementary Figure 119. ^{1}H NMR of compound **53** (400 MHz, Chloroform-*d*)

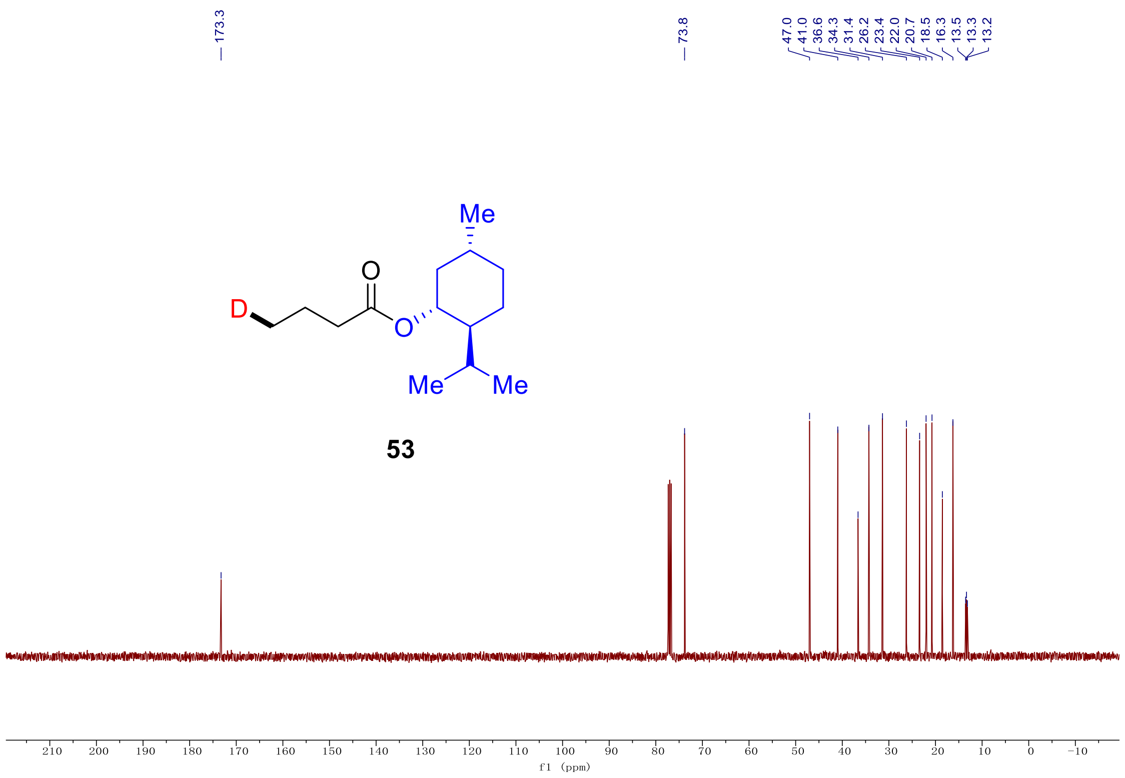

Supplementary Figure 120. ^{13}C NMR of compound **53** (100 MHz, Chloroform-*d*)

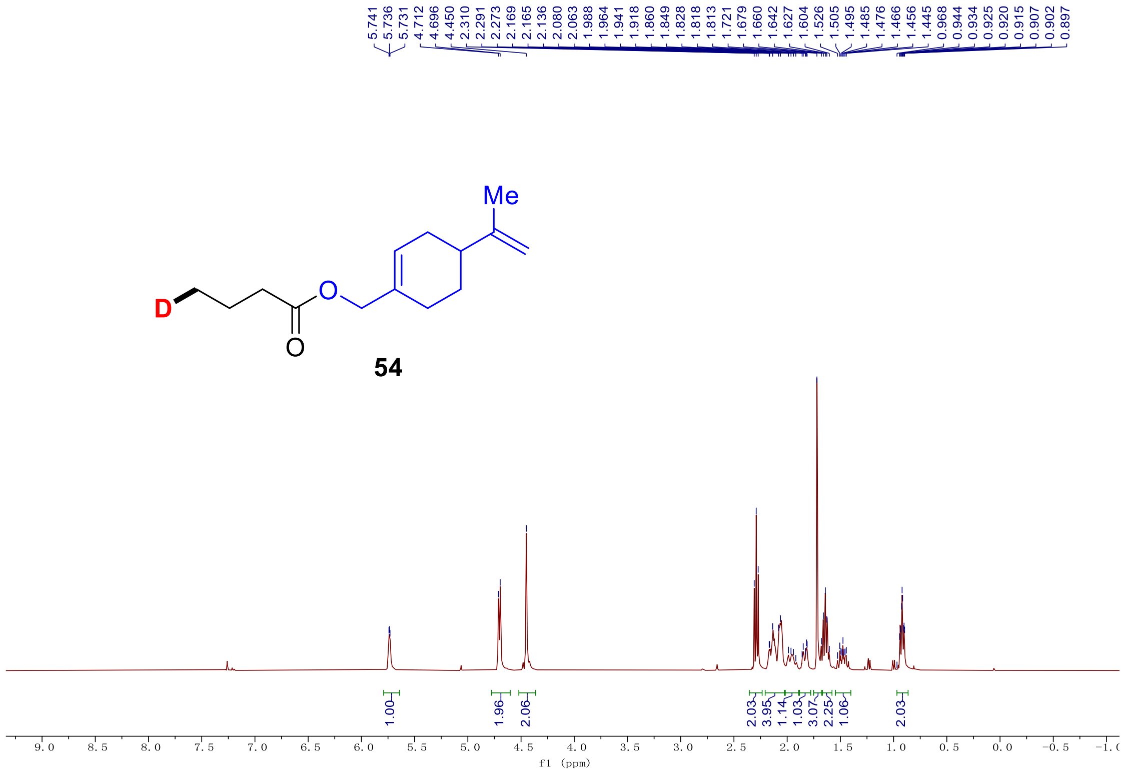

Supplementary Figure 121. ^{1}H NMR of compound **54** (400 MHz, Chloroform-*d*)

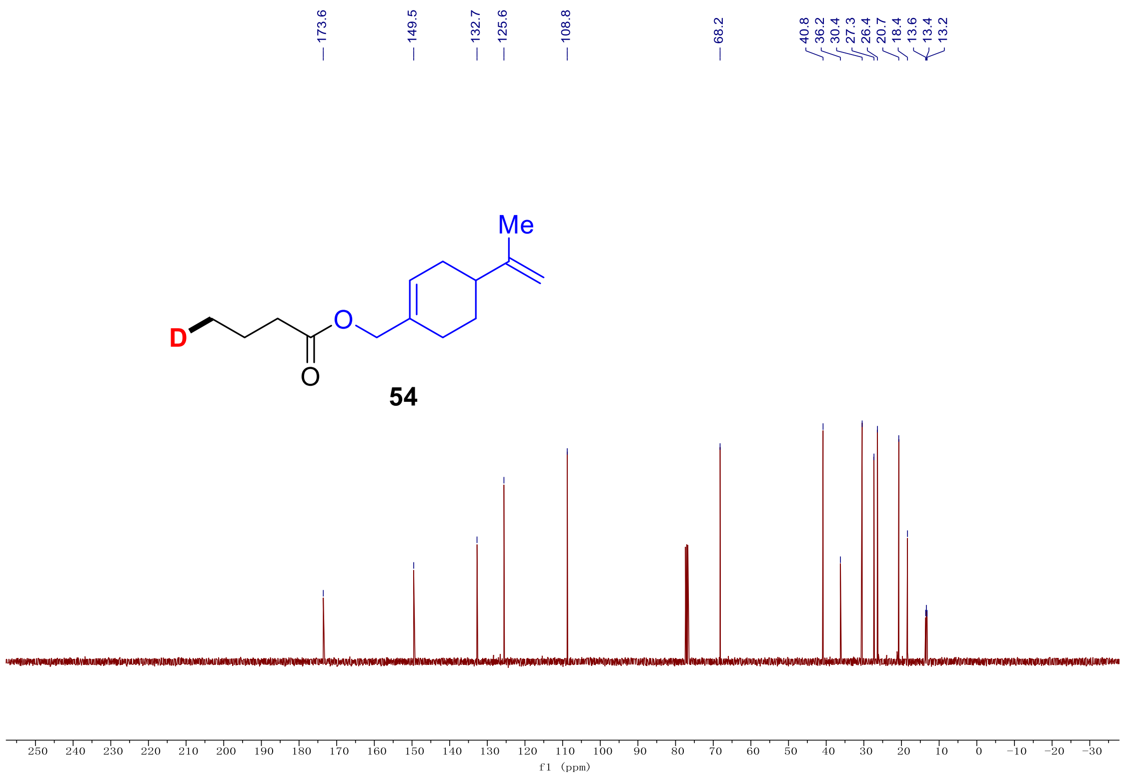

Supplementary Figure 122. ^{13}C NMR of compound **54** (100 MHz, Chloroform-*d*)

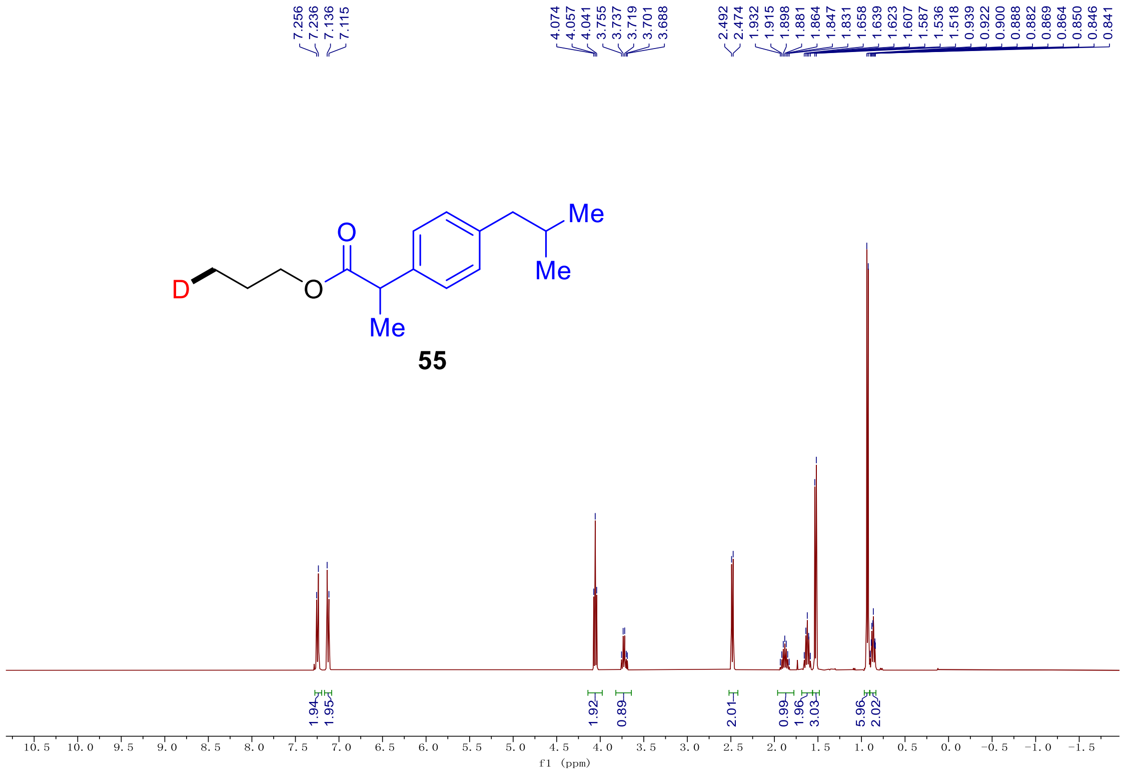

Supplementary Figure 123. ^{1}H NMR of compound **55** (400 MHz, Chloroform-*d*)

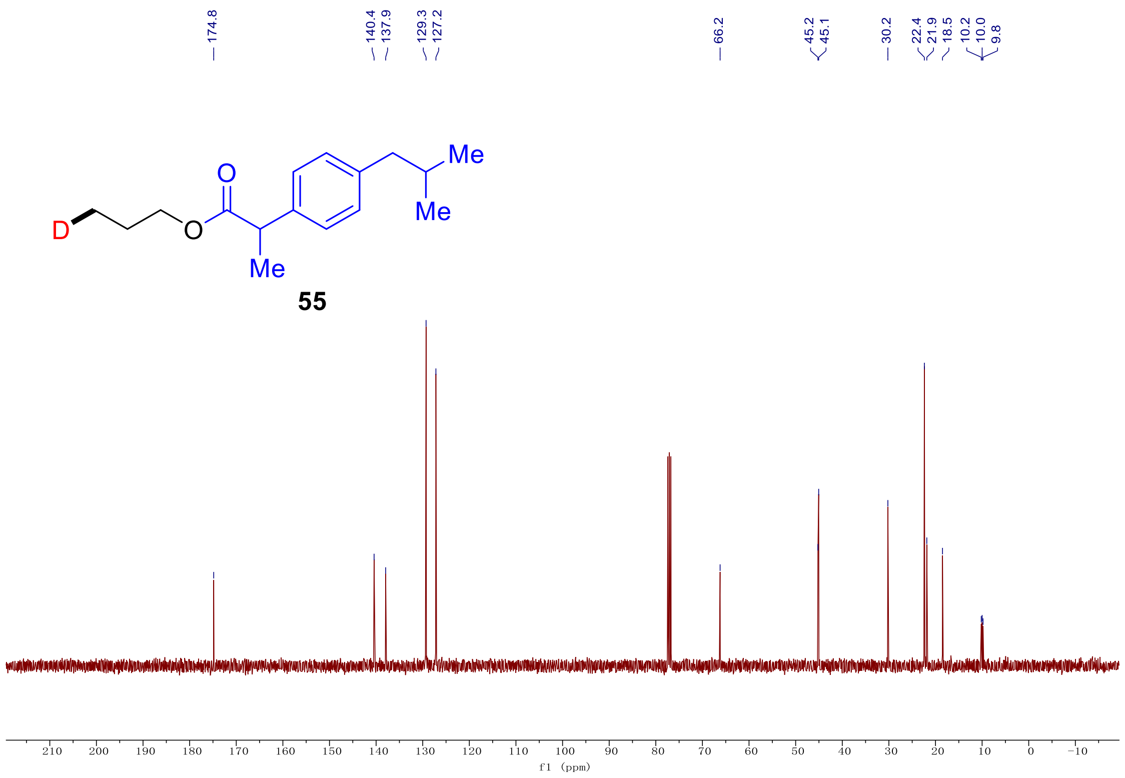

Supplementary Figure 124. ^{13}C NMR of compound **55** (100 MHz, Chloroform-*d*)

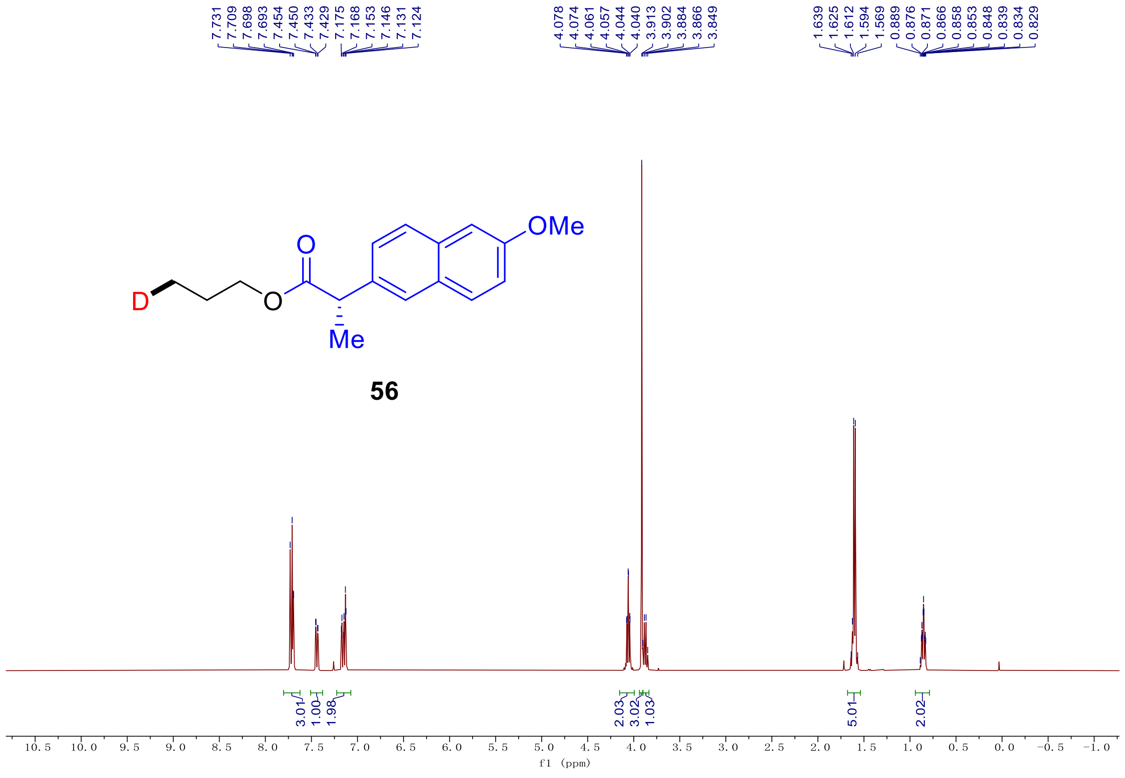

Supplementary Figure 125. ^{1}H NMR of compound **56** (400 MHz, Chloroform-*d*)

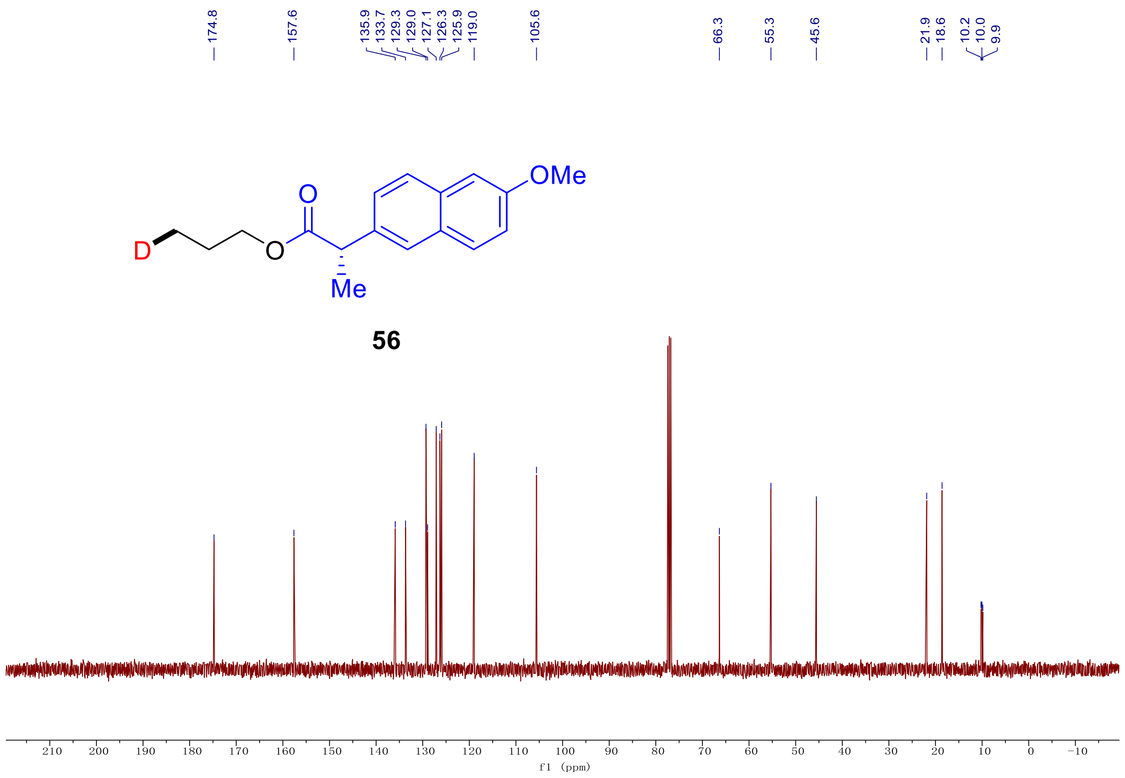

Supplementary Figure 126. ^{13}C NMR of compound **56** (100 MHz, Chloroform-*d*)

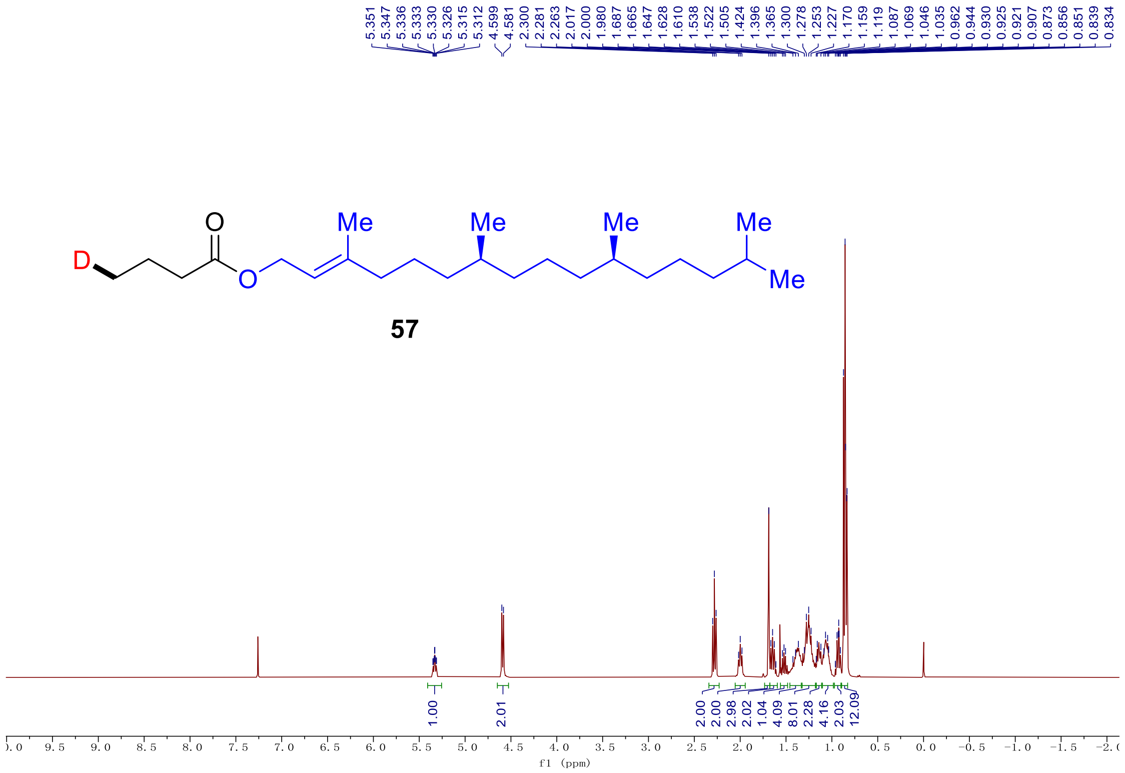

Supplementary Figure 127. ^{1}H NMR of compound **57** (400 MHz, Chloroform-*d*)

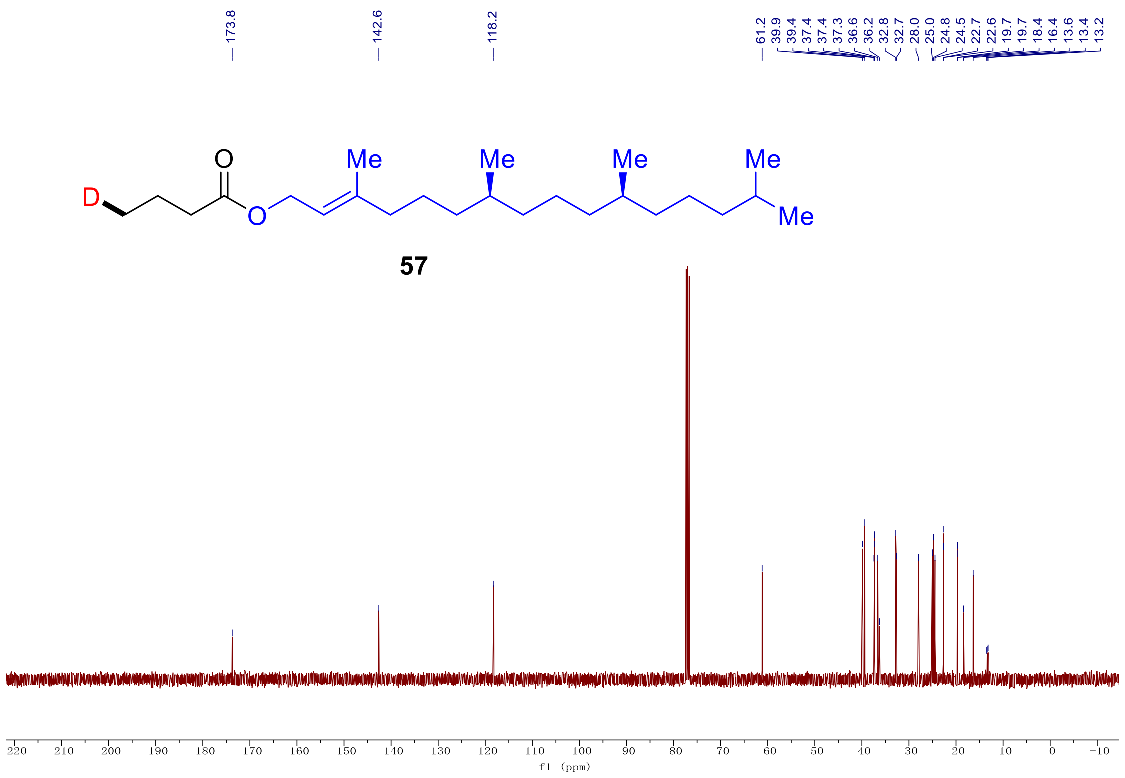

Supplementary Figure 128. ^{13}C NMR of compound **57** (100 MHz, Chloroform-*d*)

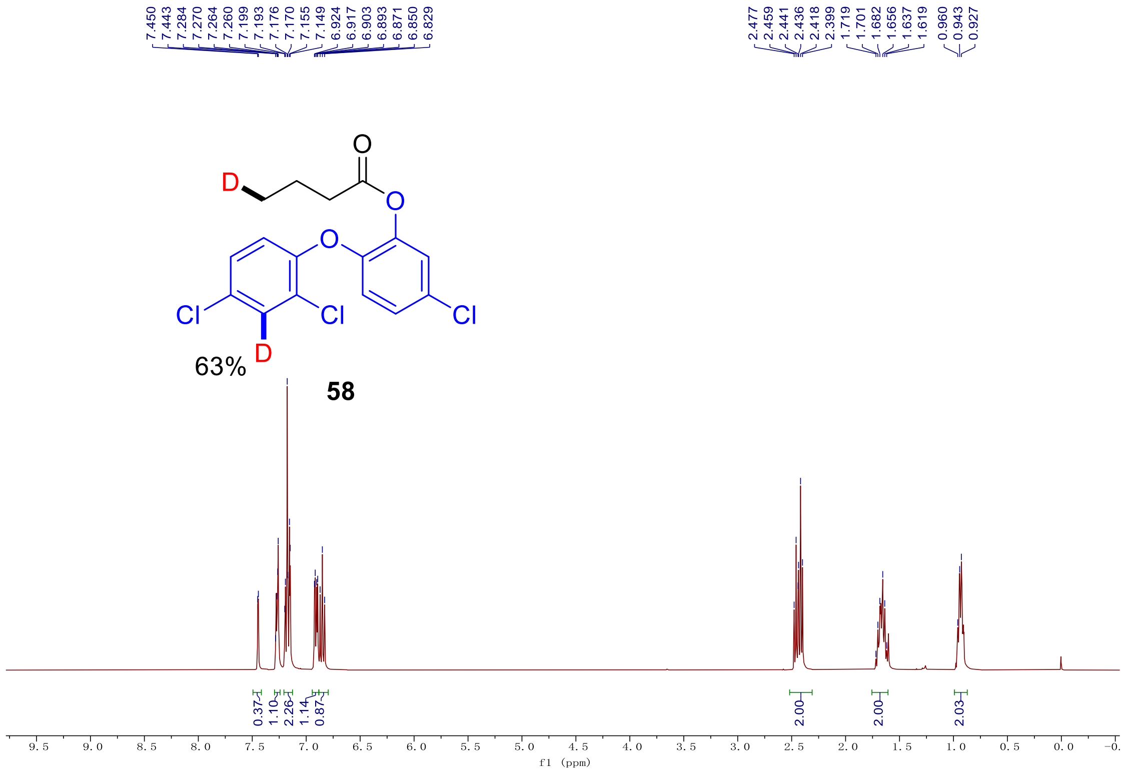

Supplementary Figure 129. ^{1}H NMR of compound **58** (400 MHz, Chloroform-*d*)

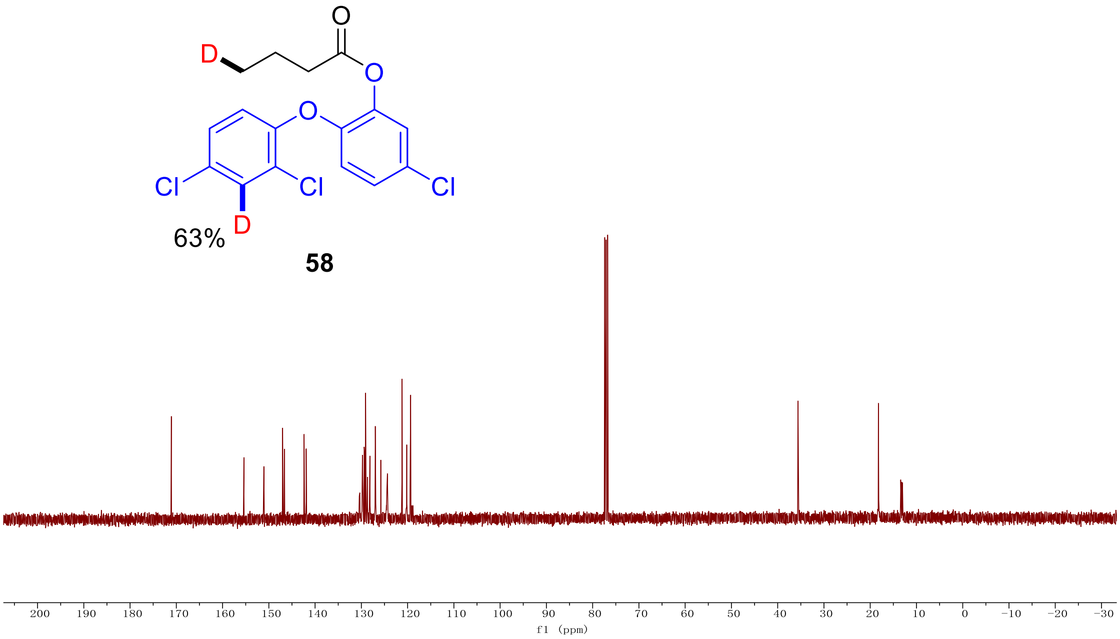

Supplementary Figure 130. ^{13}C NMR of compound **58** (100 MHz, Chloroform-*d*)

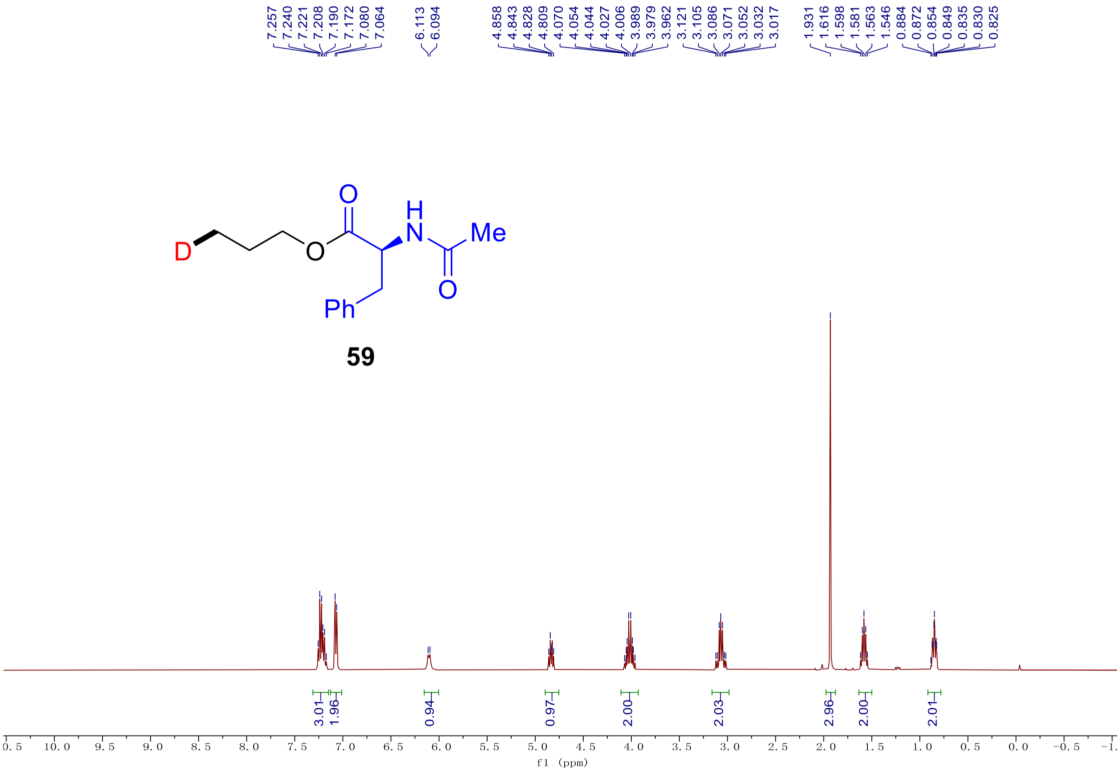

Supplementary Figure 131. ^{1}H NMR of compound **59** (400 MHz, Chloroform-*d*)

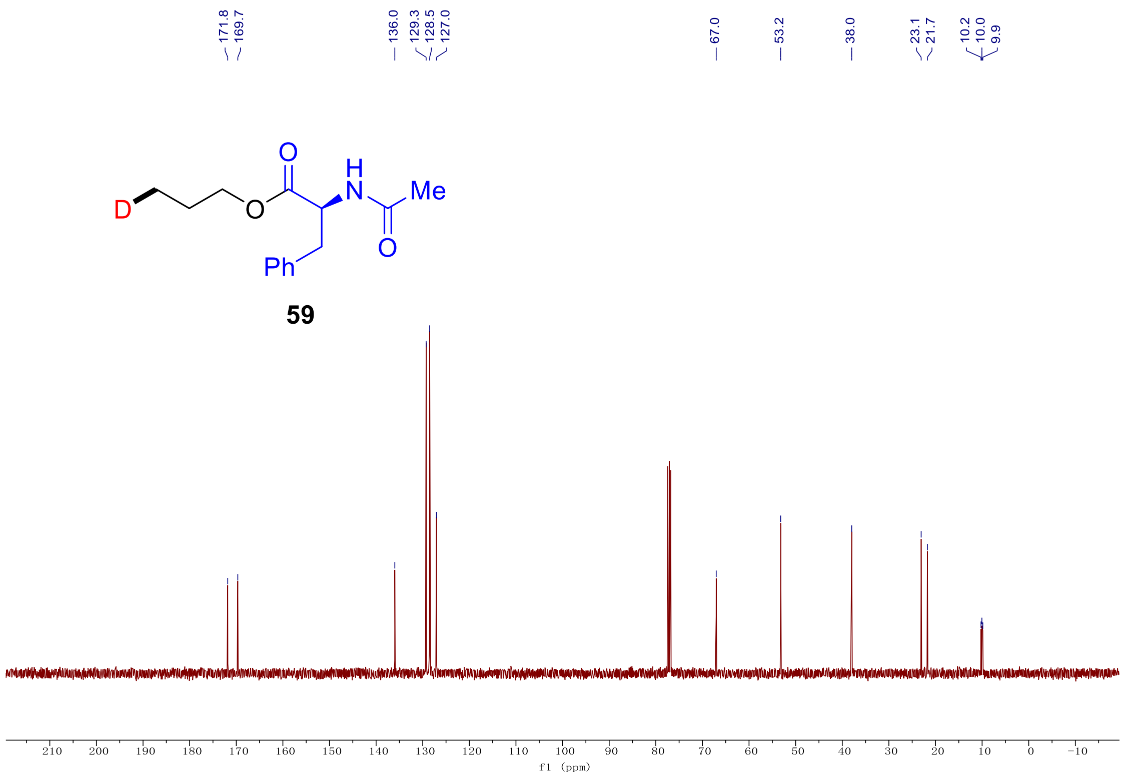

Supplementary Figure 132. ^{13}C NMR of compound **59** (100 MHz, Chloroform-*d*)

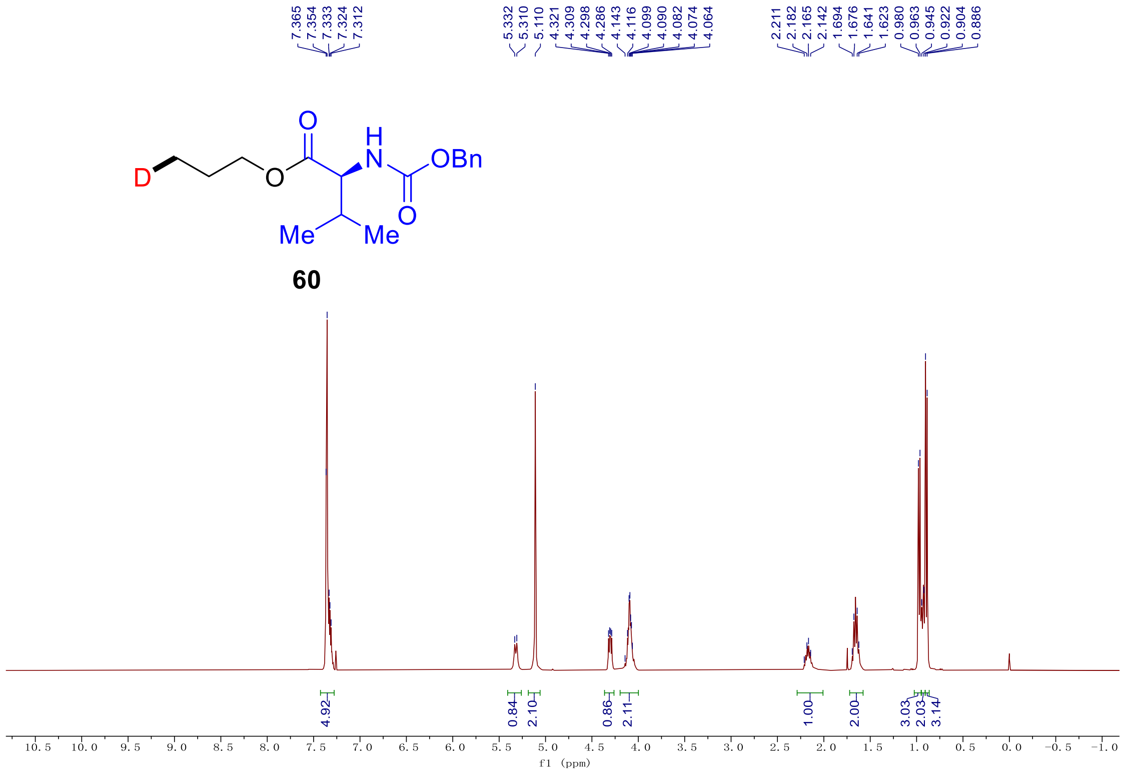

Supplementary Figure 133. ^{1}H NMR of compound **60** (400 MHz, Chloroform-*d*)

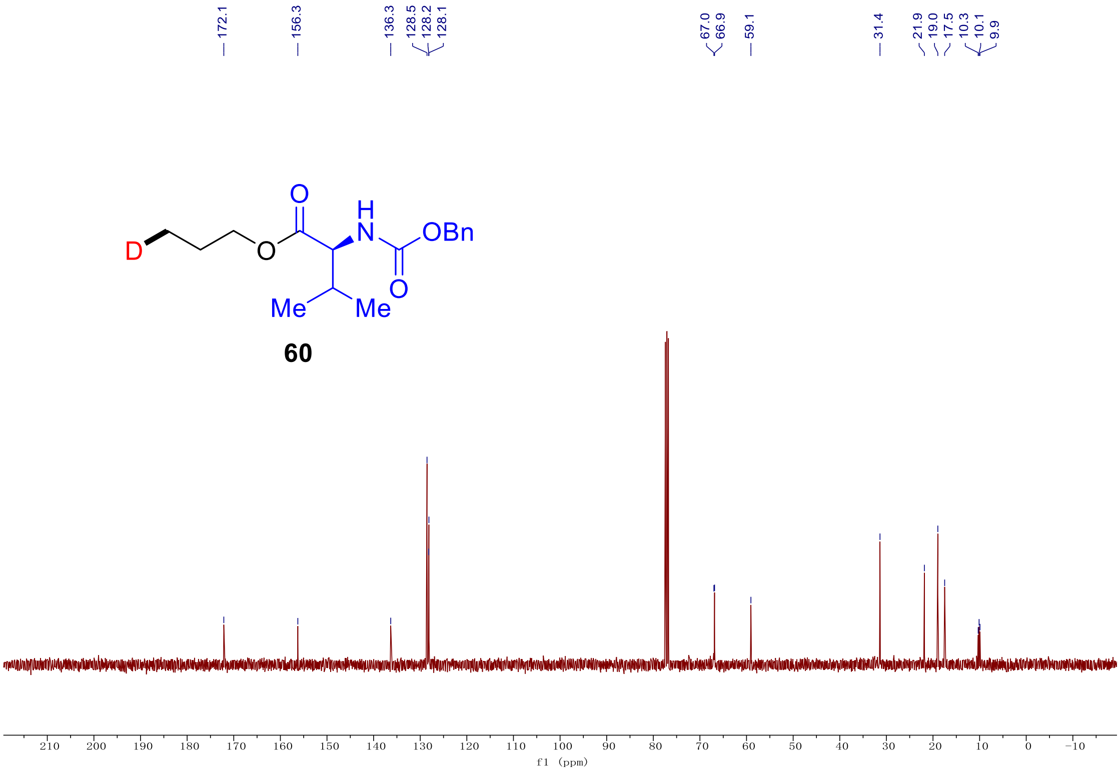

Supplementary Figure 134. ^{13}C NMR of compound **60** (100 MHz, Chloroform-*d*)

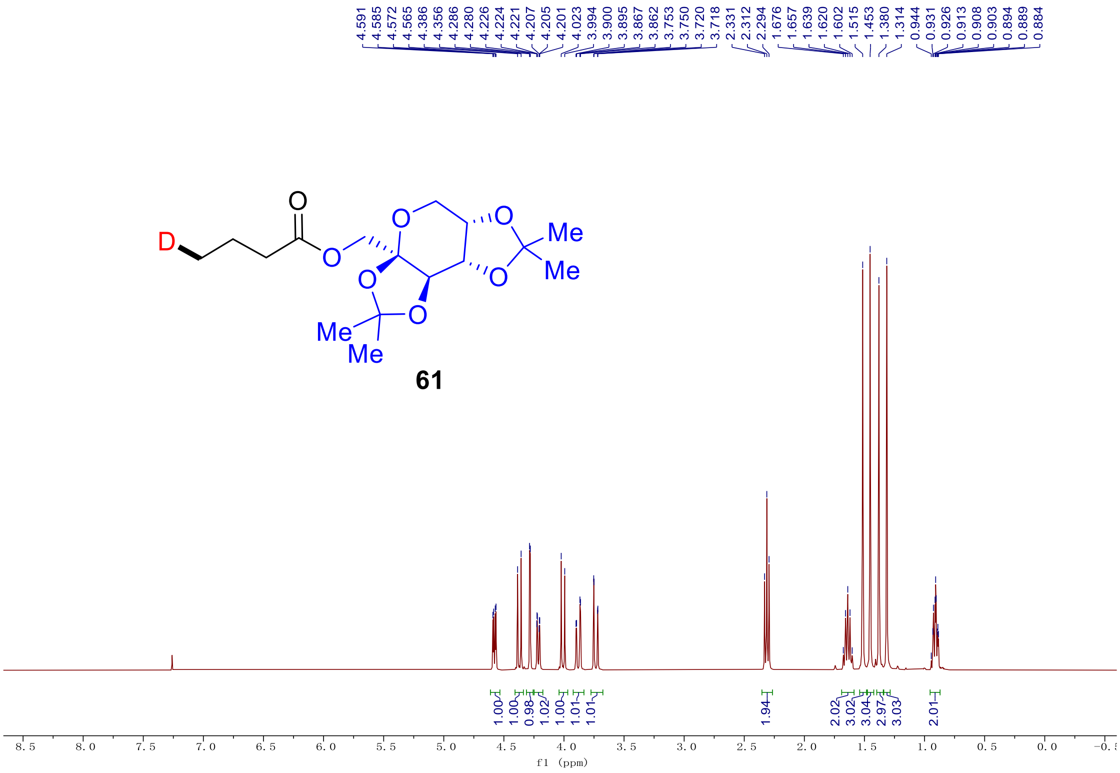

Supplementary Figure 135. ^{1}H NMR of compound **61** (400 MHz, Chloroform-*d*)

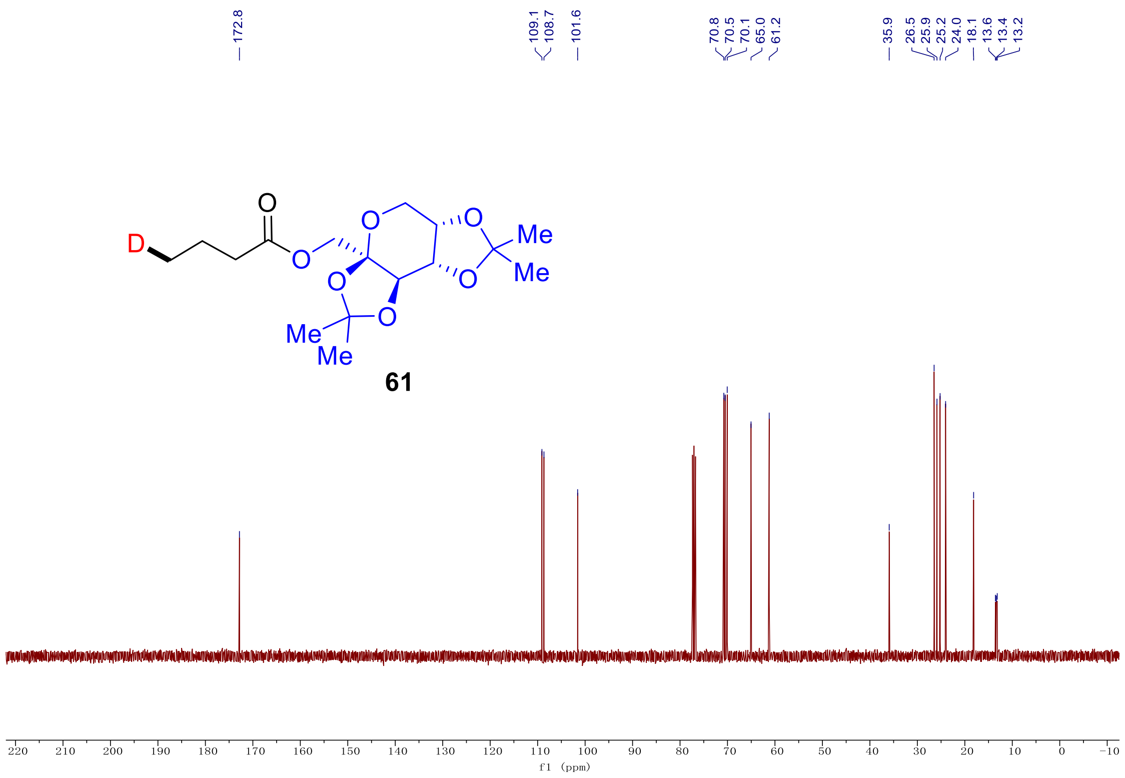

Supplementary Figure 136. ^{13}C NMR of compound **61** (100 MHz, Chloroform-*d*)

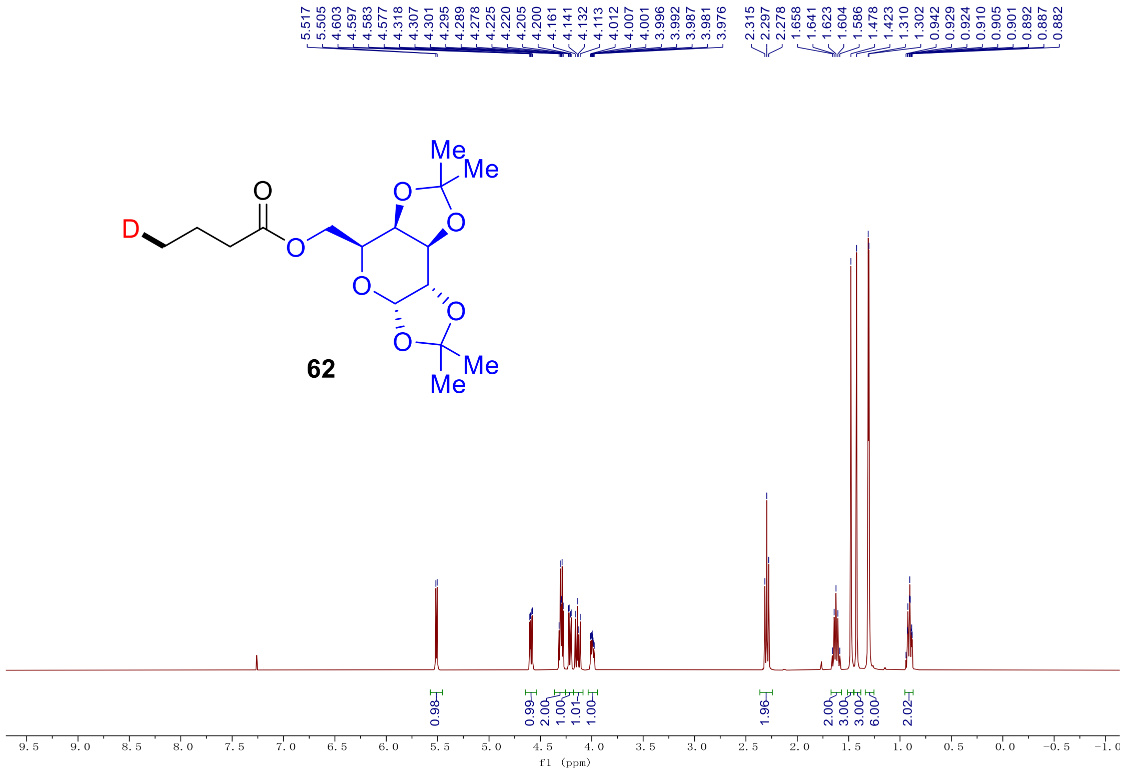

Supplementary Figure 137. ^{1}H NMR of compound **62** (400 MHz, Chloroform-*d*)

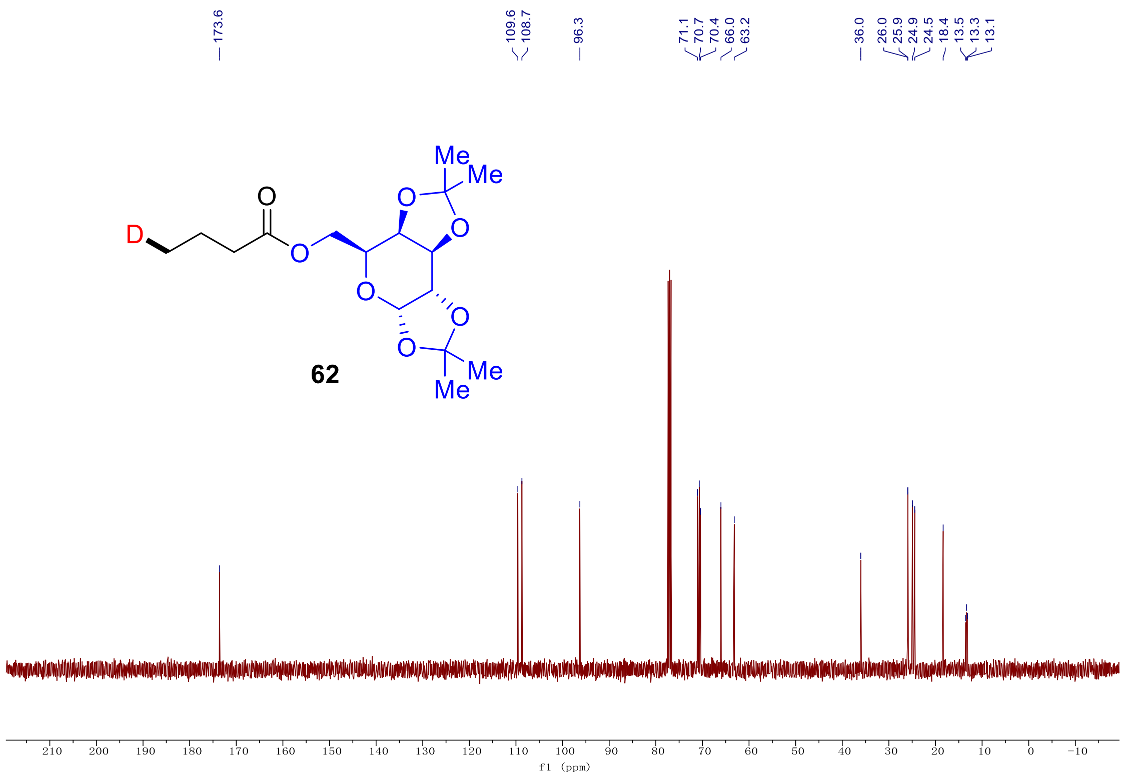

Supplementary Figure 138. ^{13}C NMR of compound **62** (100 MHz, Chloroform-*d*)

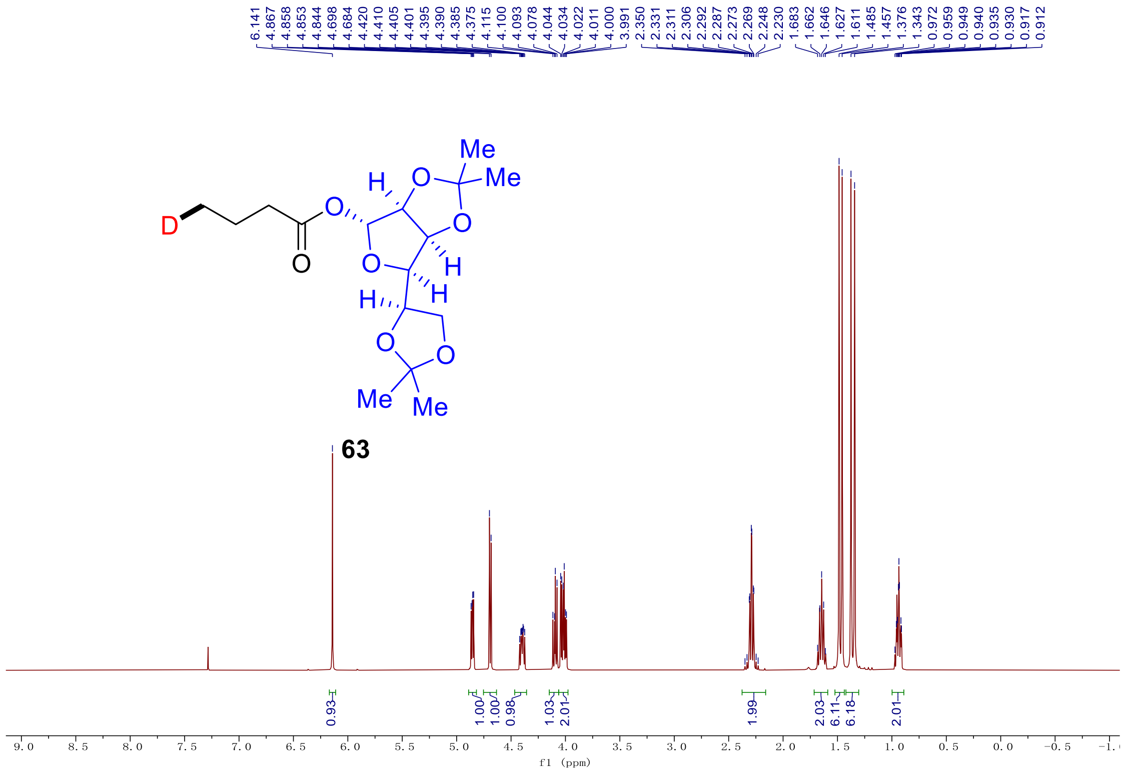

Supplementary Figure 139. ^{1}H NMR of compound **63** (400 MHz, Chloroform-*d*)

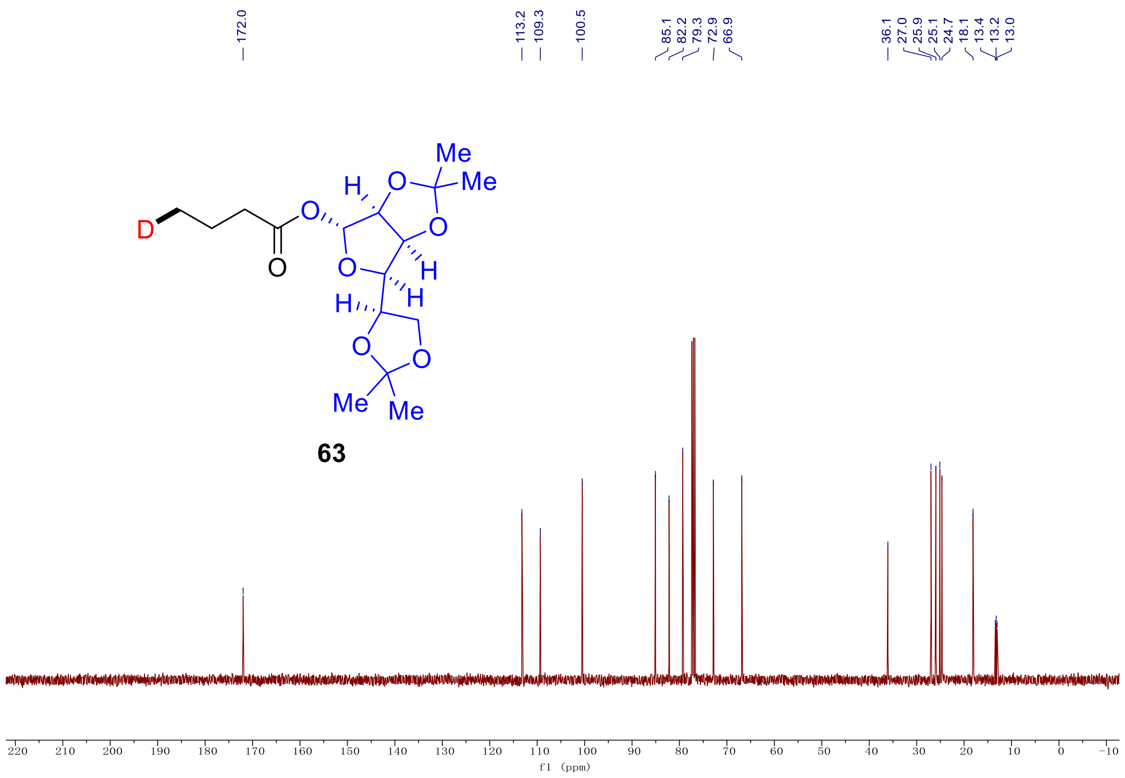

Supplementary Figure 140. ^{13}C NMR of compound **63** (100 MHz, Chloroform-*d*)

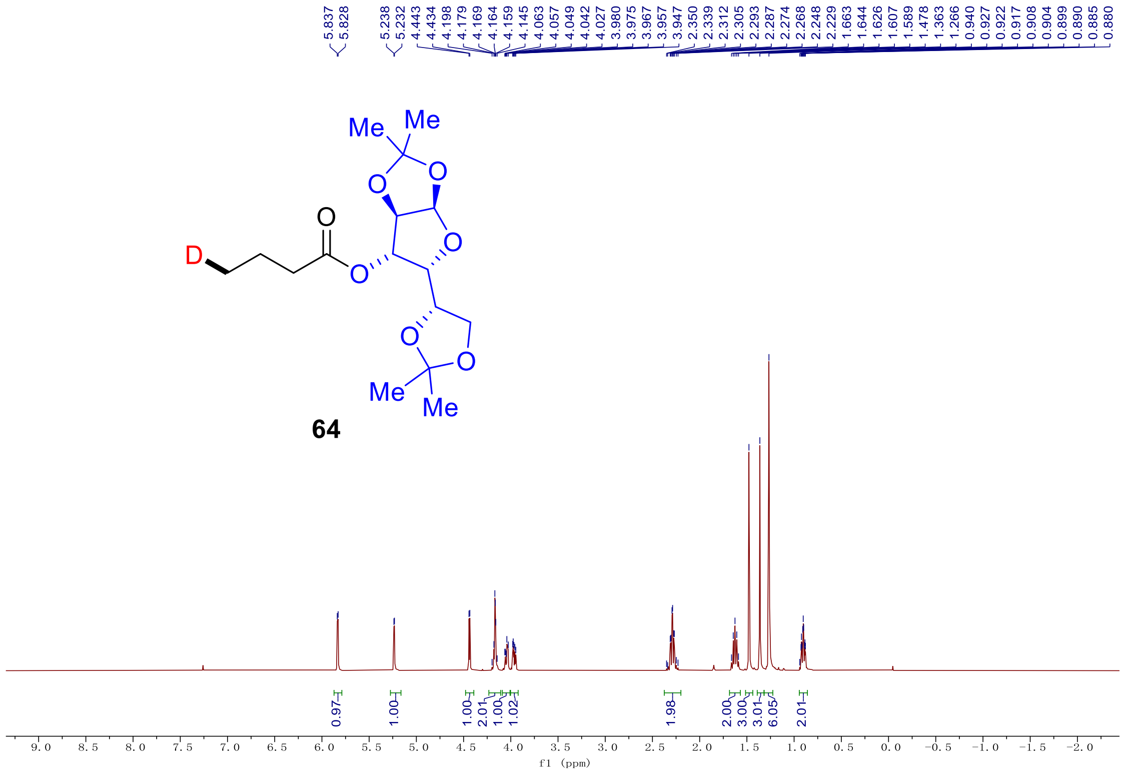

Supplementary Figure 141. ^{1}H NMR of compound **64** (400 MHz, Chloroform-*d*)

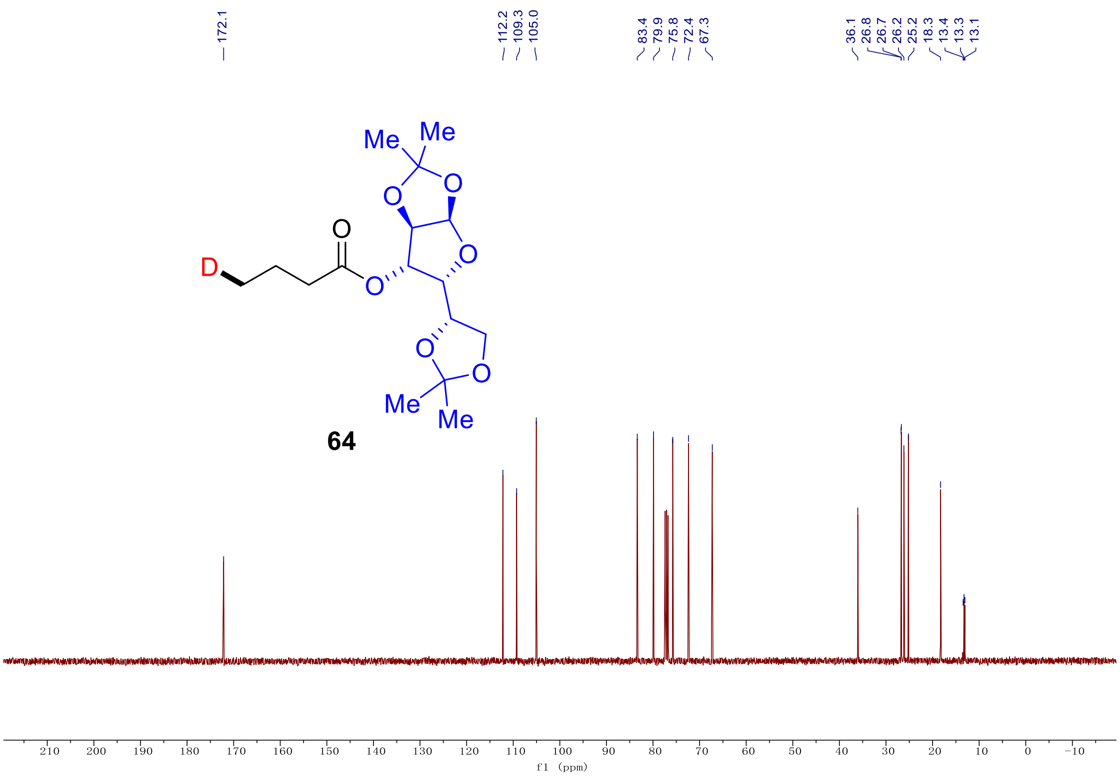

Supplementary Figure 142. ^{13}C NMR of compound **64** (100 MHz, Chloroform-*d*)

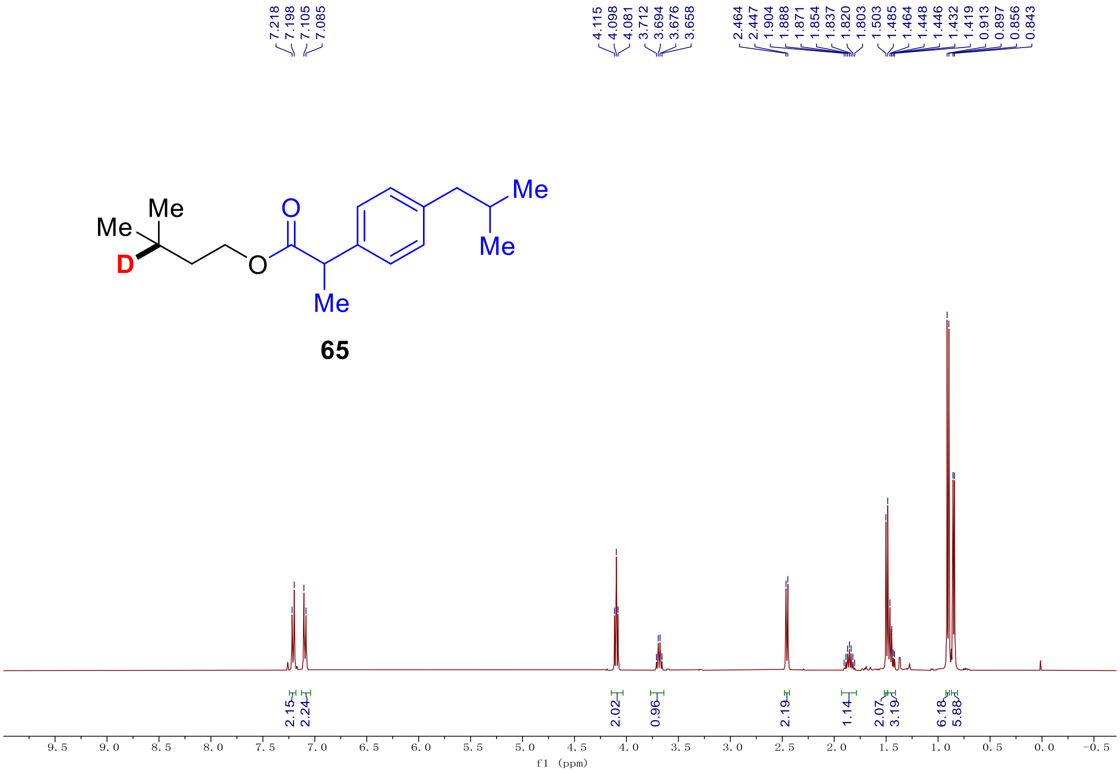

Supplementary Figure 143. ^{1}H NMR of compound **65** (400 MHz, Chloroform-*d*)

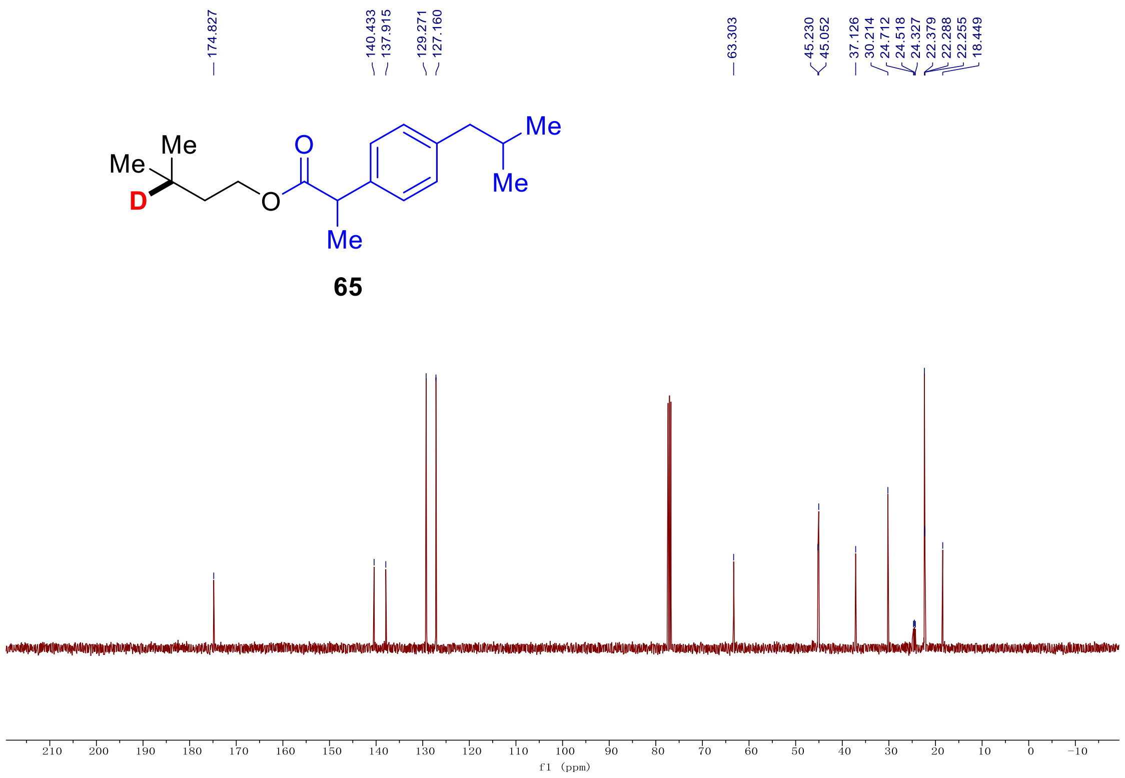

Supplementary Figure 144. ^{13}C NMR of compound **65** (100 MHz, Chloroform-*d*)

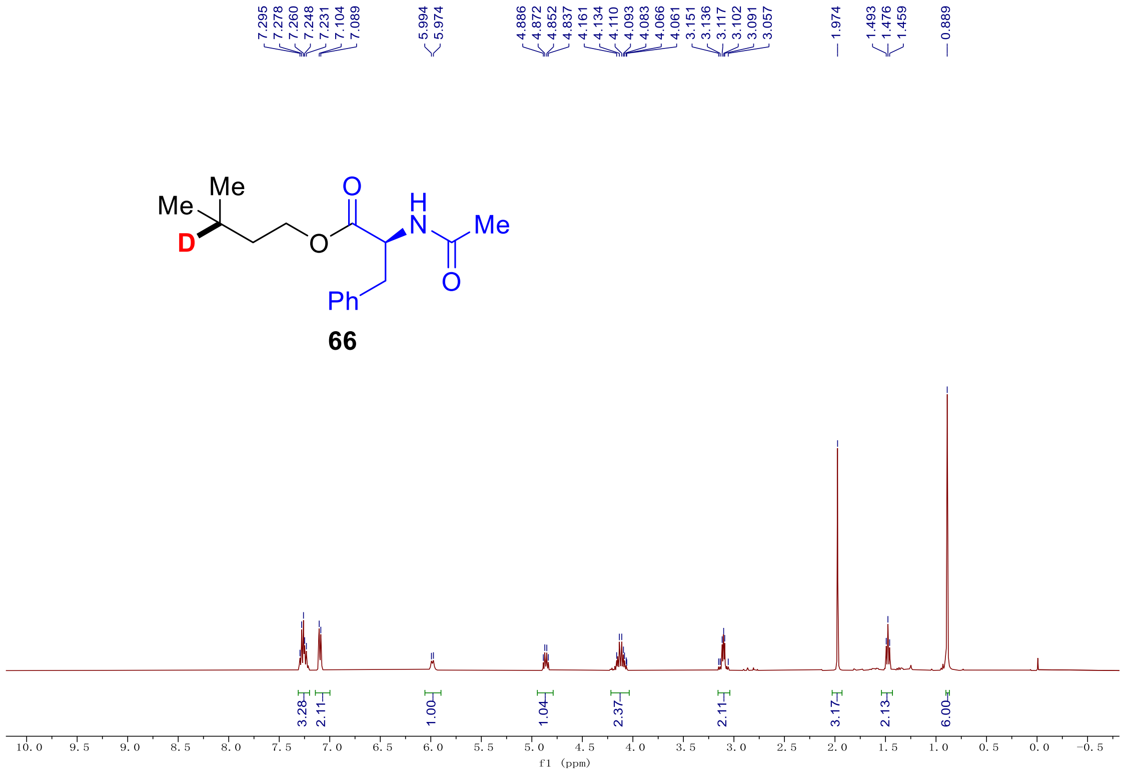

Supplementary Figure 145. ^{1}H NMR of compound **66** (400 MHz, Chloroform-*d*)

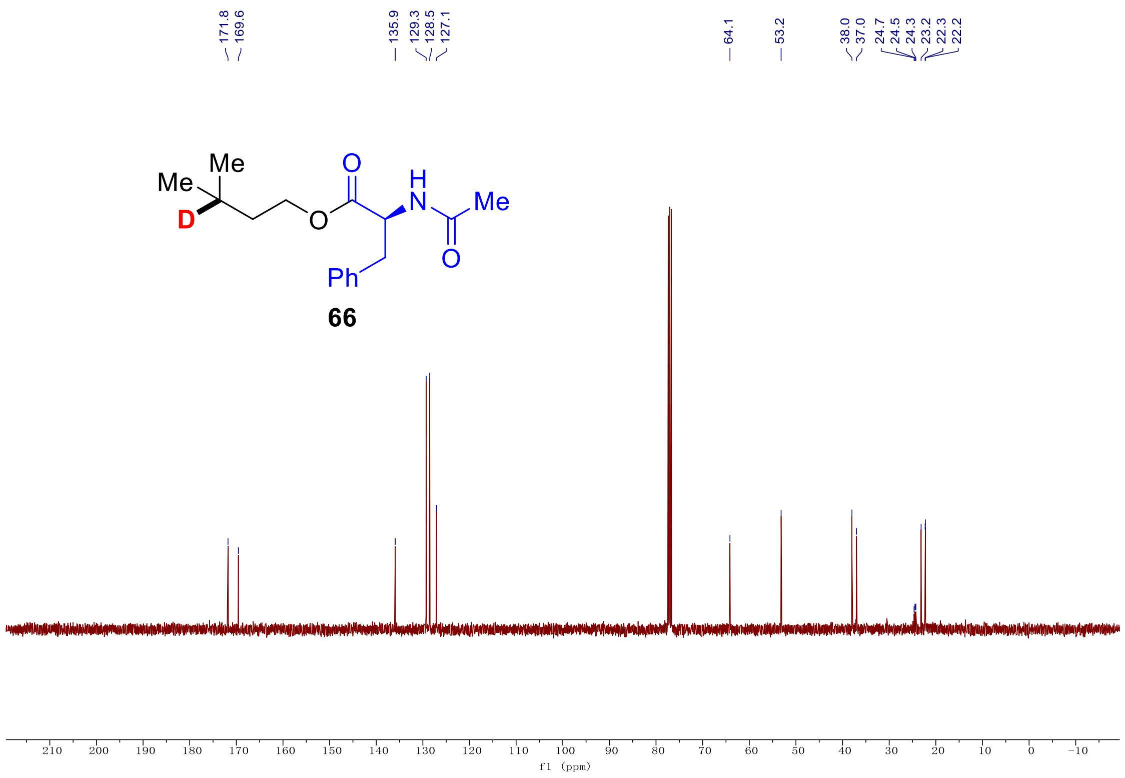

Supplementary Figure 146. ^{13}C NMR of compound **66** (100 MHz, Chloroform-*d*)

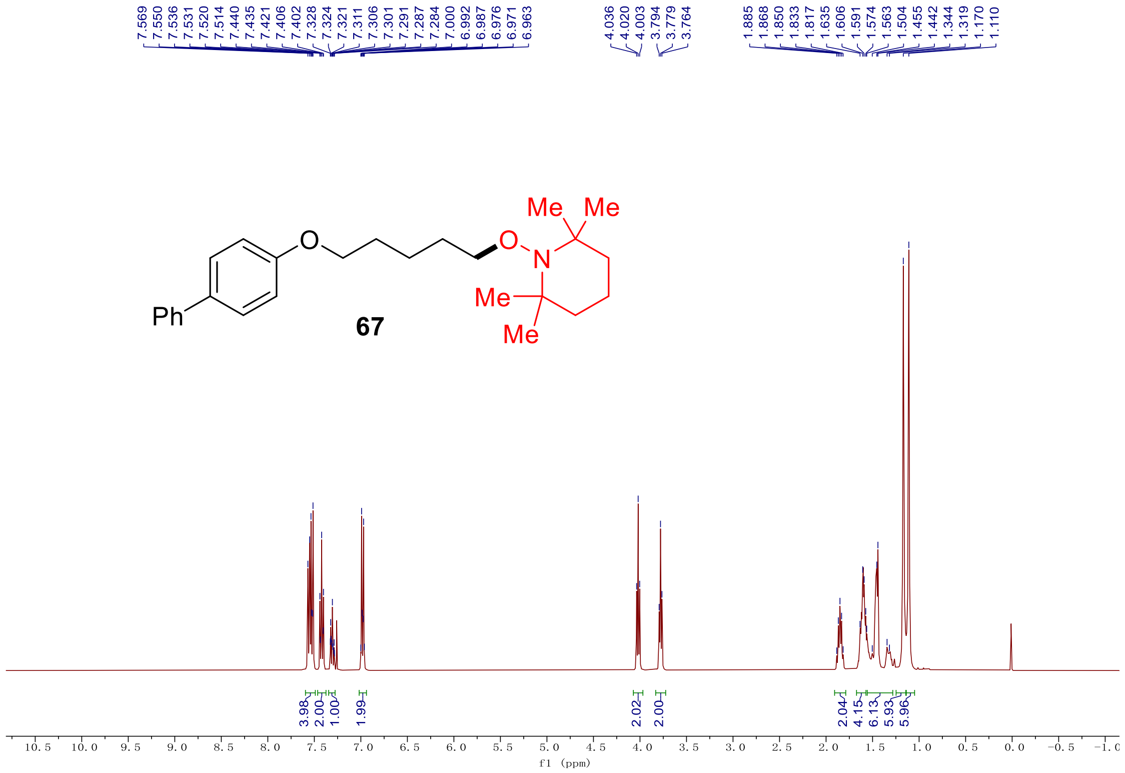

Supplementary Figure 147. ^{1}H NMR of compound **67** (400 MHz, Chloroform-*d*)

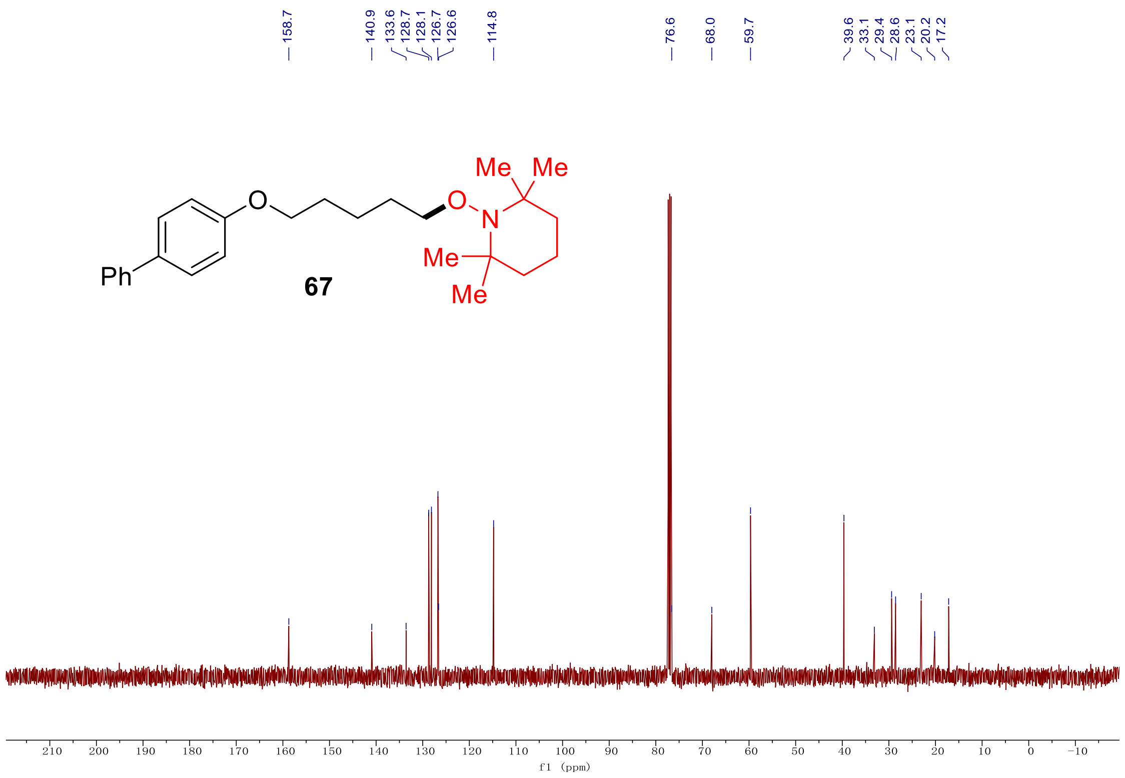

Supplementary Figure 148. ^{13}C NMR of compound **67** (100 MHz, Chloroform-*d*)

Supplementary References

1. Yi, J., Lu, X., Su, Y.-Y., Xiao, B., Liu, L. Nickel-Catalyzed Sonogashira Reactions of Non-activated Secondary Alkyl Bromides and Iodides. *Angew. Chem. Int. Ed.* **52**, 12635–12639 (2013).
2. Li, K., Zhang, K., Huang, H., Zhang, Q., Song, C. Direct Integration of Phthalazin one and Succinimide Scaffolds via Rh(III)-Catalyzed C−H Functionalization. *Asia n J. Org. Chem.* **10**, 1–9 (2021).
3. Li, Y., Ye, Z., Lin, Y.-M., Liu, Y., Zhang, Y., Gong, L. Organophotocatalytic selective deuterodehalogen ation of aryl or alkyl chlorides. *Nat. Commun.* **12**, 2894–2906 (2021).
4. Duan, J., Du, Y.-F., Pang, X., Shu, X.-Z. Ni-catalyzed cross-electrophile coupling between vinyl/aryl and alkyl sulfonates: synthesis of cycloalkenes and modification of peptides. *Chemical Science*, **10**, 8706-8712 (2019).
